# How CT happened: the early development of medical computed tomography

**DOI:** 10.1117/1.JMI.8.5.052110

**Published:** 2021-10-29

**Authors:** Raymond A. Schulz, Jay A. Stein, Norbert J. Pelc

**Affiliations:** a Varian, A Siemens Healthineers Company, Palo Alto, California, United States; b Hologic, Inc., Marlborough, Massachusetts, United States; c Stanford University, Department of Radiology, Stanford, California, United States

**Keywords:** computed tomography, early history, technical development

## Abstract

As we arrive at the 50th anniversary of the first computed tomography (CT) scan of a live patient, we take this opportunity to revisit the history of early CT development. It is not an exaggeration to say that the invention of CT may represent the greatest revolution in medical imaging since the discovery of x-rays. We cover events over a period of about two decades that started with the realization that accurate cross-sectional soft-tissue detail is possible and could be a significant advance. We describe in some detail the development of the first CT system and then the rapid technical advances during the following years that included the entry of many companies into the field and the circumstances that led many of those entrants to exit the field. Rather than focusing on the specific technical details (which can be found elsewhere), we include stories and events in the hope that broader lessons can be learned. As the first x-ray-based digital imaging modality, CT brought into common use an exceptional tool that benefits countless patients every day. It also introduced dramatic changes to biomedical imaging as a field that continues to influence progress to this day.

## 1 Introduction

**Fig. 1:**
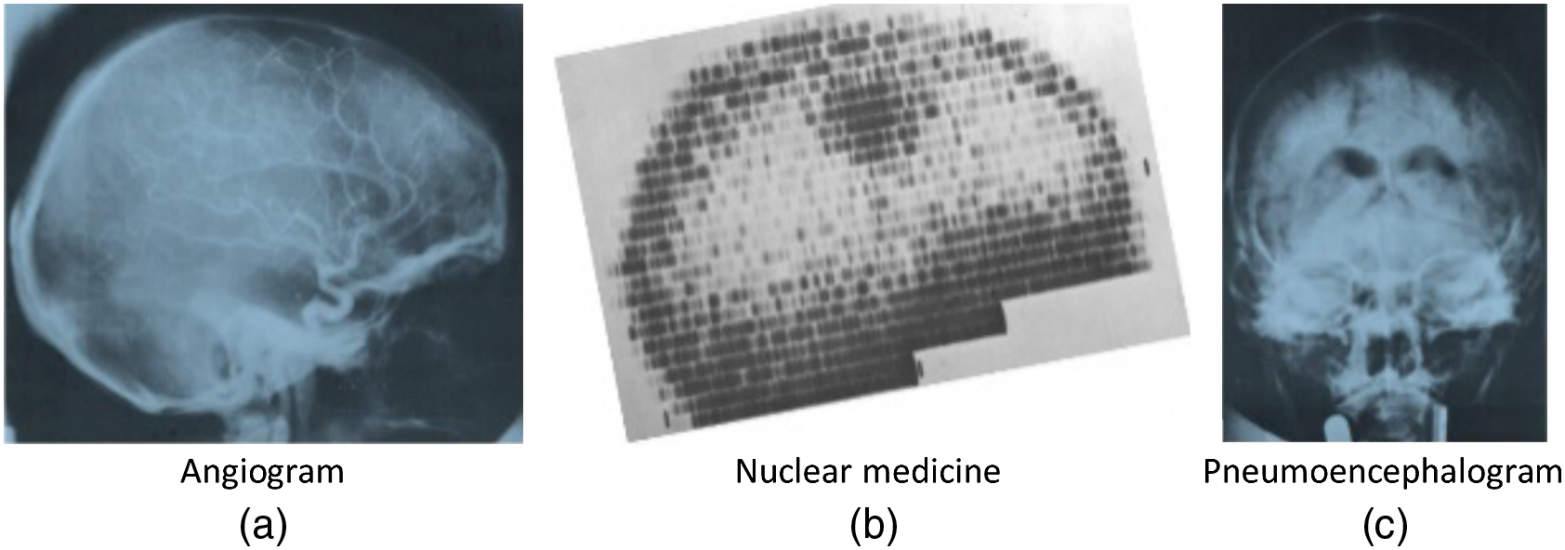
CT replaced a number of more invasive procedures. It completely replaced pneumoencephalograms, a very difficult procedure for patient and physician and reduced the use of catheter angiography and nuclear medicine.^2^ Images are from Ref. 3.

On Friday, October 1, 1971, a new procedure was performed to image a live patient’s brain. After a (lengthy) computer processing reconstruction delay, a remarkable image appeared on the screen of a monitor, sparking a revolution in medical imaging. Image “200.2A” was of a middle-aged female patient of Dr. James Ambrose at Atkinson Morley Hospital with a suspected tumor in the left frontal lobe, which was successfully excised and confirmed as a cystic astrocytoma.^1^ The scanning process was painfully slow. But since each new image was as revolutionary as the prior, it was worth the wait. So began a new chapter in medical imaging and diagnostic procedures that would quickly render obsolete painful, dangerous, time consuming, and often unhelpful pneumoencephalograms, ventriculograms, as well as many angiograms, and nuclear medicine procedures^2^ (Fig. 1). See Ref. 4 for a review of the clinical impact of CT.

Because of the importance of CT and its development, there have been many articles written about its history, including work in other fields. We will not cover all the history nor the technical details of the various advances. For those topics, we might suggest Refs. 5 to 12. Rather, we focus on efforts directly related to the introduction of diagnostic x-ray computed tomography (CT) and its early refinement, and specifically on some stories that may not be widely known. We begin with some efforts toward CT prior to the work of Godfrey Hounsfield. Although other researchers worked in areas related to CT (e.g., early attempts at axial tomography that did not include accurate reconstruction),^5^ we have restricted ourselves in this narrative to work directly related to medical x-ray CT, beginning with the contributions of Allan Cormack. We describe the development of the first CT system and what happened in the next decade or two. We intend to show the process by which CT became successful.

There is obviously more material than can be included in this paper. Some, including brochures for early CT scanners and other background material, are contained in Supplementary Material^13^ related to this paper.

## 2 CT Work Prior to Hounsfield

Allan M. Cormack studied physics at Cape Town University and Cambridge University. He returned to South Africa and worked at Groote Schuur Hospital in the mid 1950s, overseeing not only the safe handling of radioisotopes but also, first-hand, the use of x-rays for diagnosis and therapy. He noted that the methods used for dosimetry were crude compared with those in the physical sciences and that precise x-ray dosimetry could only be accomplished with knowledge of the distribution of attenuation coefficients between the source and the point of interest, and sought to determine this map from transmission measurements through the object.^14^ He found that, while the literature described the measurement of the attenuation coefficient of a uniform material, no method was available for inhomogeneous media, and so he sought to develop such a technique. While his main motivation was dosimetry, he recognized that these maps of attenuation would have other benefits, including eliminating the limitation in radiography due to the fact that images of a region of interest are superimposed on images of tissues above and below the structures of interest. His progress on this problem proceeded at a modest pace because it was a side interest, separate from his main work responsibilities. Cormack realized that the problem was a mathematical one, that of solving for a function from its line integrals. He searched the literature and consulted with mathematicians in Cape Town, found no solution, and first derived a solution for objects with circular symmetry. He had a phantom built from wood and aluminum, made transmission measurements along parallel lines using a Co60 source (only a single view was needed because of the circular symmetry), and was able to calculate attenuation coefficients for these materials that were in good agreement with independent measurements. The results were published in 1963.^15^ He then extended his theory to objects without circular symmetry. Implementation required knowledge of computer processing techniques, which was provided by a student, David Hennage. Cormack made measurements of a phantom composed of aluminum and Lucite and produced plots of attenuation coefficient along profiles through the object. This was arguably the first experimental demonstration of computer assisted tomography, and the work warranted subsequent recognition in sharing of the Nobel Prize with Hounsfield. The results were published in 1964.^16^ Years later he came across papers on reconstruction by Radon and others and cited them in a 1973 paper that also described application to imaging of positron emitters and also transmission measurements with heavy charged particles.^14^^,^^17^ With respect to his work in the 1960s, Cormack recounted, “Attempts were made to interest hospital physicists in the results, but to no avail.”^14^

A group at Siemens, led by Dr. Friedrich Gudden, also conducted work on what was eventually called CT, at about the same time as, and without knowledge of, the work of Hounsfield at EMI.^9^^,^^18^ Starting in 1967, they realized they could make cross-sectional images of attenuation coefficients from x-ray transmission measurements. These scientists were working within the radiology field, and, as such, were potentially subject to the opinions and biases of the field at the time. One of these was the importance of high spatial resolution. They understood basic requirements for tomographic reconstruction and realized that to achieve spatial resolution comparable to radiography they would need thousands of projections, each with thousands of samples, and they would have to produce images with very large matrix sizes. These computations were beyond the capabilities of computers at that time. Given these obstacles to being able to achieve spatial resolution of a fraction of a millimeter [and perhaps not fully appreciating the impact of low contrast detectability in three-dimensions (3D)], the project was terminated. Meanwhile, in the UK, unaware of the prior work of Cormack or Gudden when they started the project, Hounsfield and EMI were on their way to building a clinical prototype CT head scanner, as described in the next section.

## 3 Godfrey Hounsfield and the Development of the First Clinical CT System

Godfrey N. Hounsfield joined Electric and Musical Industries (EMI) in 1949 when he was 30. His only formal training was an Associates Certificate from the Faraday House.^19^ EMI at the time was a leading electronics conglomerate including music, film, electronics, military, and other businesses. He had a passion for understanding basic principles of technologies and worked on radar during the wartime effort prior to joining EMI. In his first years at EMI, he continued to work on radar systems and their displays.^19^^,^^20^ Years later, he led the design team that developed the UK’s first all transistor general purpose computer, the EMIDEC 1100. His engineering background would prove essential to his seminal contribution to medicine.

As his computer work wound down, Hounsfield was transferred to EMI’s Central Research Lab (CRL), which was famous for pioneering stereo recording, television broadcasting, and radar and communications work. Working within CRL on pattern recognition techniques, he was asked to think about projects that might be fruitful. He focused on that idea and asked himself the general question: could the unknown contents of a box be calculated by taking “readings” through the box?^21^ As it happened, in the late summer of 1967, during a vacation trip he met a physician who bemoaned the drawbacks of conventional radiography. This may have been his first introduction to human anatomy as an example of the problem of “reading through a box.” He pondered whether a system could theoretically recognize text in a closed book, page by page, “by shining a bright light across each page from various angles and measure what came out the other end.” He had simplified the volumetric “book-box” problem to a two-dimensional problem by breaking it down into a series of parallel slices. He believed that “given enough information you could calculate what was written on the page.”^20^

In late fall of 1967, Hounsfield showed he could iteratively compute a 3×3 grid of numbers from sums along horizontal, vertical, 45 deg, and 135 deg lines (projections) using manual arithmetic. He then approached Stephen Bates at CRL. They worked together and by December, using a link to a remote time-sharing computer, they created a program to compute the contents of an 8×8 matrix of numbers. He developed a solution to the CT reconstruction problem using the simplest possible arithmetic that was compatible with the available computers and without the knowledge of previous mathematical approaches.^20^

Funding for this work was always an issue in the early years. Hounsfield did unfunded work from November 1967 to July 1968, and it was during this period the idea for a “3D x-ray” was formed.^20^ By June, he had initiated a patent search and filed a patent disclosure. However, the director of CRL “would not allocate any of the small amount of available EMI funds to 3D x-ray unless there was external funding as well.”^20^ Hounsfield sought funding advice from the UK Department of Health and Social Security (DHSS) and submitted a proposal and funding request for £10K in August. While that request was rejected, he received advice for improvements and in October he submitted a funding application entitled “Proposed Project: An Improved Form of X-Radiography.”^13^ Its purpose was “to investigate the employment of a computer to make better use of the information when an object is examined by gamma rays or x-rays.”^20^^,^^22^

**Fig. 2:**
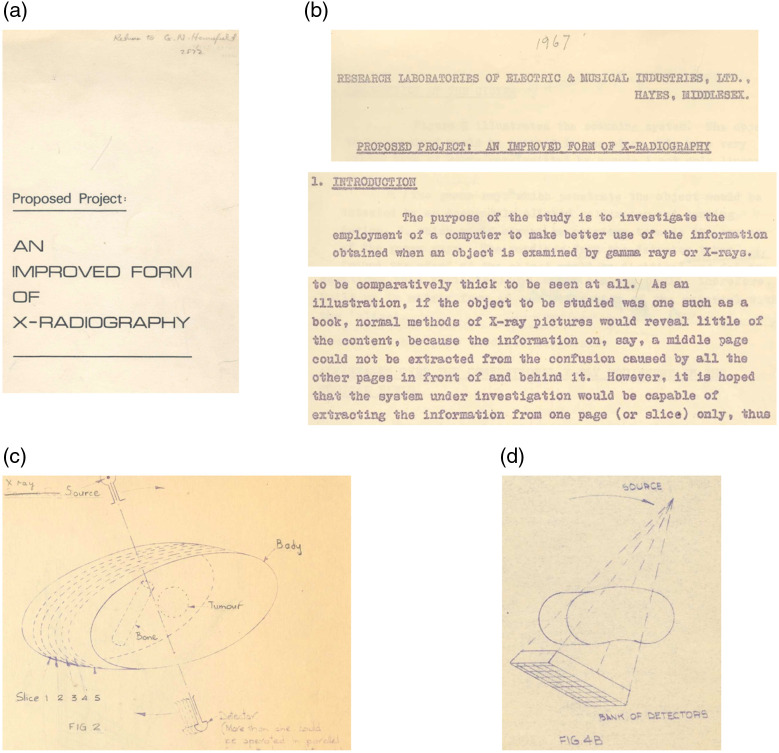
Portions of Hounsfield’s October 7, 1968, proposal. (a) Cover page, (b) introduction and book (object) and page (slice) analogy, (c) diagram of slicing patient anatomy, (d) diagram of bank of detectors to gather volume of anatomy. Images are from Ref. 20.

The proposal, portions of which are shown in Fig. 2, demonstrates that in 1968 Hounsfield had a clear mental model of CT without yet having seen a CT image or having done any practical tests. He foresaw the benefits of CT including high dose efficiency, sensitivity to <1% changes in attenuation, and lack of shadows from otherwise superimposed objects, and he also foresaw the need for windowing to handle the wide dynamic range in the image. The proposal explained the idea of tomographic slices using the “book” analogy: “if the object to be studied was one such as a book, normal methods of X-ray pictures would reveal little… (however) the system under investigation would be capable of extracting information from one page (or slice)…”^20^ [Figs. 2(b) and 2(c)]. He addressed the benefits compared with conventional tomography and described potential future roles in detecting tumors in patients. He discussed technical aspects, such as the choice of source and detector. He described how scatter and beam hardening affect CT, and the interactions between scan time and various system design choices. Amazingly, he anticipated volumetric cone-beam scanning, showing a diagram of a rectangular bank of detectors, to examine the entire anatomic volume [Fig. 2(dl)]. He laid the foundations of a completely new medical field and associated industry, including not only the key elements of the first CT scanner but also those of many systems to follow.^20^^,^^23^

The proposal initially received £2500 DHSS funding on October 28, 1968, plus equivalent meager funding by EMI; the total was half of what he had requested. He received some additional funding over time but, incredibly, total funding of just £69,000 resulted, in almost exactly three years, in the first CT image of a live patient.

**Fig. 3:**
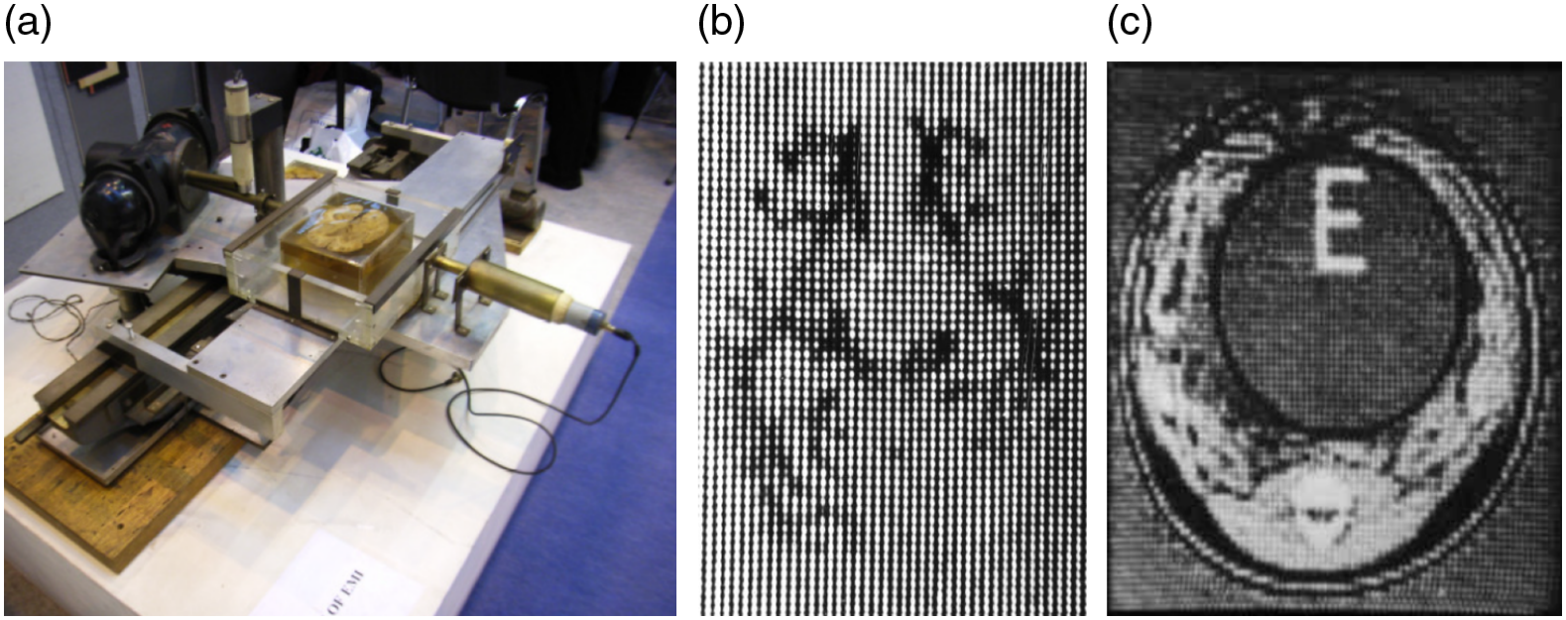
(a) Lathe scanner, (b) first brain specimen image, and (c) scan of piglet carcass. Images are from Refs. 22 and 24.

The first test bed scanner was built using parts salvaged from a lathe for the scanning mechanism and, initially, an Am241 source. It required nine days to complete a scan and 2.5 h of computer time. While this arrangement was useful for phantom tests, cadaveric scans were difficult because of cadaveric decomposition. Using an x-ray tube reduced the scan time to 9 h (Fig. 3), speeding up research. Samples were obtained from collaborating radiologists, Drs. Frank Doyle, James Ambrose, and Louis Kreel.^6^^,^^25^

The largest reconstruction matrix feasible on the timesharing system was 32×32. In 1969, the reconstruction was moved to the ICL 1905 mainframe, which, after optimization, was able to reconstruct 80×80 images in about 20 min. Images were stored on paper tape (one image took about 90 m of tape), which was fed to a viewer that could create photographic prints using a Polaroid camera coupled to an oscilloscope. Each printed image required an exposure taking 60 min.^20^ Changing window level and width took about an hour to rewind and reread the tape.^6^^,^^20^

Like other academic neuroradiologists, Dr. James Ambrose had spent much time seeking alternatives to the painful methods of examining the brain, which were in clinical use at the time but which were far from ideal. He was well-aware of the unmet need. Hounsfield, seeking collaborators, met Ambrose at Atkinson Morley Hospital in 1969. Ambrose showed Hounsfield his department and explained its activities. At the end of the tour Hounsfield stated, “I can do much better than that.” Ambrose challenged him to show what he could do with a brain specimen with a lesion. Hounsfield also received specimens from Drs. Frank Doyle and Louis Kreel. Five weeks after the tour, Hounsfield visited Ambrose again with a Polaroid photograph showing a CT scan of Ambrose’s brain specimen [Fig. 3(b)]. Ambrose saw the “electrifying” image and asked Hounsfield “Do you realize what you have done?” Hounsfield responded “Yes.” Radiology would never be the same again.^1^

Only 15 samples were scanned on the lathe bed scanner before the decision, in January, 1970, to build a clinical prototype for installation at Atkinson Morley Hospital. In February, Hounsfield visited leading radiologists in teaching hospitals with a business colleague from EMI to assess the likely market for CT scanners. The response was very discouraging. Since the discovery of x-rays in 1895, a central priority had been improving spatial resolution, and radiologists were concerned that CT would have much lower spatial resolution than film. Further, they were unaccustomed to using computers, and the system would be far more expensive than the equipment with which they were familiar. The doubters saw CT as an expensive way of getting worse images.^20^^,^^23^

By August, 1970, the specifications of the clinical prototype were finalized, and the system would be completed a little over a year later.^6^ In the meantime, Hounsfield gave talks showing results from the lathe-bed system. His presentations were greeted with limited enthusiasm. Most radiologists did not initially appreciate the benefits of eliminating tissue superimposition and achieving better than 0.5% density discrimination in 3D. This reaction, however, did not reduce Hounsfield’s determination.

**Fig. 4:**
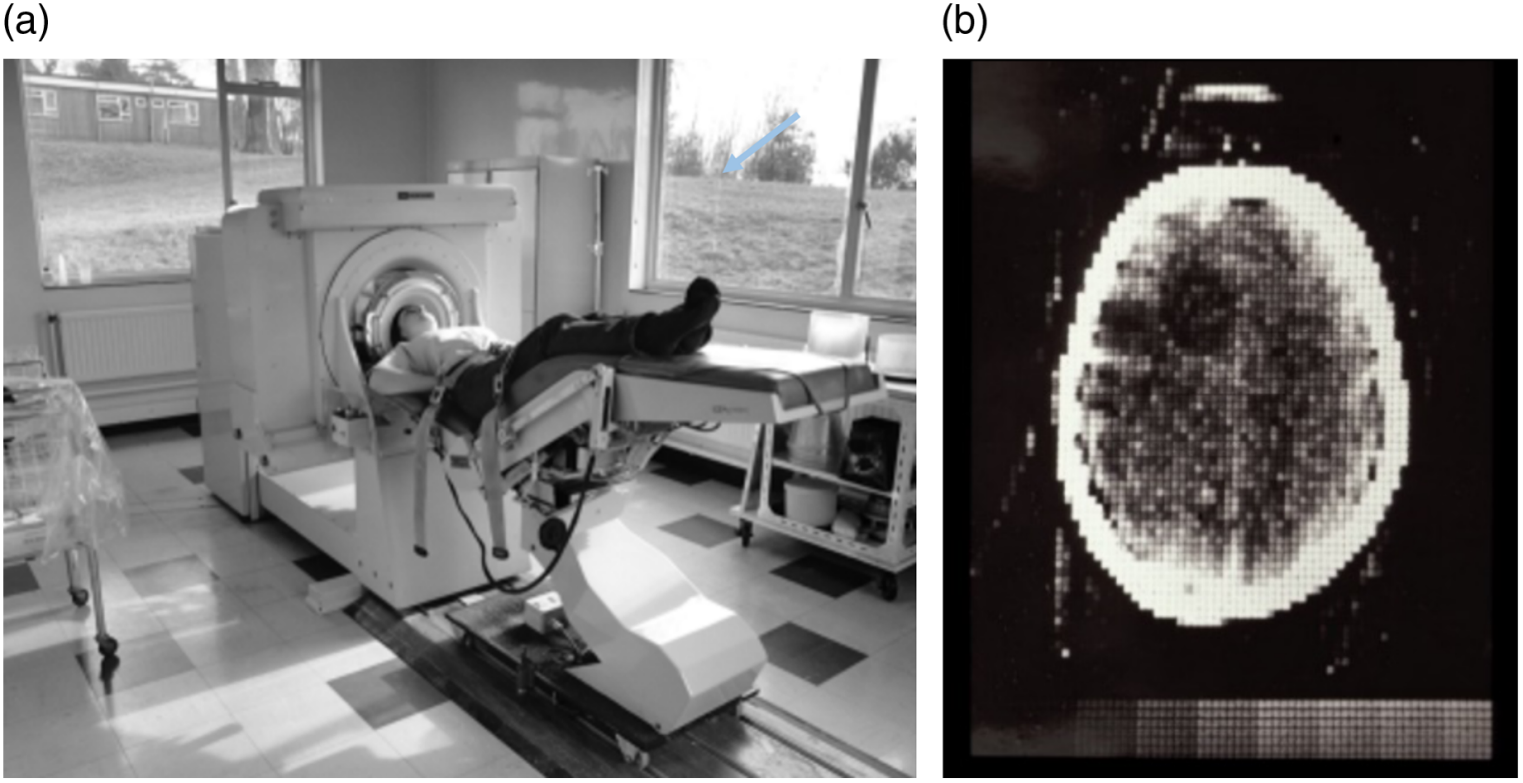
(a) The first EMI scanner at Atkinson Morley Hospital. (b) Image of first patient scan demonstrating the cystic astrocytoma (arrow added). Images from Refs. 22 and 26.

The first CT scan of a live patient took place on October 1, 1971, under the supervision of Dr. Ambrose, but two days passed before Dr. Ambrose would see the images because they were reconstructed on an off-site time-shared mainframe computer. (Minicomputers, which made CT practical, were just becoming available.) Ambrose notes that he and Hounsfield “jumped up and down like football players who had just scored a winning goal”^1^^,^^2^ when they first saw them. Ambrose said that the first clinical scan on October 1, 1971 (Fig. 4), showed a great deal more detail than he was expecting. He likely expected only the less impressive image quality produced by the lathe bed scanner, whereas the human scanner had further improvements developed in the interim.^20^^,^^23^

**Fig. 5:**
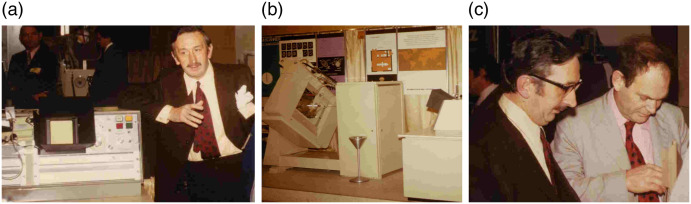
RSNA 1972. (a) Hounsfield next to a viewing unit (described below), (b) EMI-scanner on display, and (c) Hounsfield and Ambrose preparing the first RSNA lecture on computerized axial scanning. Images courtesy of Mac Gollifer and Richard Waltham.

The first presentation of this early clinical data was held at the British Institute of Radiology conference in April 20, 1972,^13^^,^^25^ by which time some 70 patients had been scanned. This resulted in a local story in *The Times*, including an image from the first patient scan and a picture of the machine.^8^ However, it was a presentation that took place in New York on Monday, May 15, 1972, that attracted the attention of leading neuroradiologists in the United States. Hounsfield and Dr. James Bull, a world-renowned British neuroradiologist, attended the annual 5-day Neuroradiology Postgraduate Course at Montefiore Hospital/Albert Einstein College of Medicine with other prominent faculty, including Drs. Juan Taveras from MGH and Ernest Wood from Columbia Presbyterian Medical Center. More than 400 other physicians also attended. Bull and Hounsfield were given the opportunity to make a 30-min presentation during a lunch hour.^13^ The combination of the specialized audience, with opinion-makers in attendance, and the revolutionary CT results, changed everything. The day following this talk, Hounsfield was featured in all three major television networks.^8^ Hounsfield visited major hospitals in the United States over the next week and other EMI representatives followed over the next months. One EMI manager noted, “Hospitals like the Mayo Clinic expect to examine 30 patients daily,”^20^ which means that “the Mayo Clinic will examine as many patients in one week as … had been examined by Atkinson Morley Hospital in Wimbledon during the whole year.”^20^ Dr. Wood advised EMI to book space at the upcoming annual meeting of the Radiological Society of North America (RSNA) in December, 1972, and to display its CT machine. EMI secured the last $10^{\prime }\times 20^{\prime }$ booth in the Palmer House Hotel in Chicago. At the RSNA meeting, Hounsfield and Ambrose were invited to give a talk in the Grand Ballroom immediately after the president’s address in a session called “New Techniques in Radiology”—the title of their talk was simply “Computerized Axial Tomography.” Their talk made quite a stir. RSNA 1972 was a major success for EMI and for CT (Fig. 5).^13^ At the trade show, EMI took multiple orders for the EMI-scanner, each requiring a nonrefundable deposit of $100,000.^9^^,^^27^

## 4 Minicomputer: An Enabling Technology

The initial images from the prototype CT scanner were reconstructed on a remote mainframe computer, requiring manual transfer of the raw data using magnetic tape and return of images on tape and on Polaroid photographs that were not available until the next day. This continued for approximately the first 6 months of scanning, limiting the number of patients scanned to only about 70. It also contributed to a low estimate of a worldwide market size of 12 units. Clinical CT could not have become widely practical without availability of more compact and lower cost computing. That was provided by minicomputers, which fortuitously developed at around the same time period.^13^ The first minicomputer, which sold for under $20,000, became available in 1965, a PDP-8, produced by Digital Equipment Corporation (DEC). Data General introduced the first 16-bit Nova in 1968 at $3999, one-fifth the price of a PDP-8, and followed with more advanced models including the Eclipse in 1974. DEC introduced the 16-bit PDP-11 in 1970. The Nova, Eclipse, and PDP-11 were good choices for early CT systems. However, image reconstruction could be a bottleneck to patient throughput, especially as more scans were acquired and matrix sizes increased. Physicians demanded faster reconstruction times and the computational power of minicomputers proved insufficient. To address this need, CT manufacturers employed array processors, such as those from Floating Point Systems or Analogic, Inc. Compared with minicomputers at the time, array processors were far faster for problems with a high degree of parallelism. Some were able to perform integer (e.g., array index) and floating point computations simultaneously. Some CT systems also employed special purpose hardware to accelerate computationally intensive operations, such as fan-beam backprojection. These computational systems allowed the general purpose minicomputer to manage the some other control and data handling processes.

Early CT systems also needed other computational functions. Data storage, which was also challenging, employed magnetic disks (which were physically large and had limited capacity) for short-term storage and magnetic tape for archiving. Data display functionality also required new subsystems, especially for image windowing. In addition, because radiologist’s workflow involved reading images on film-changers, multiformat cameras were developed that printed multiple CT images on sheets of photographic film. Film continued to be used for CT interpretation and also became part of the patient’s record.

## 5 EMI-Scanner, the First Commercial Scanner. How Did It Work? An Engineer’s Perspective

The patient was supine on a table with knees raised, and, with assistance, put his/her head inside a thick rubber sock attached to the inside of a five-sided $10^{\prime \prime }\times 10^{\prime \prime }$ (25 cm) plastic box (the sixth side was the rubber sock). The patient’s head was placed at an angle so that slices would be parallel to the orbitomeatal line (which contains the eye and ear canal). Water then filled the space between the sock and the plastic box. This “water bag” minimized the x-ray signal dynamic range, reduced x-ray beam hardening variation, and provided a water reference for CT number accuracy. It also provided a starting point for the iterative algorithm—uniform water attenuation throughout the scanned field of view.

The EMI-scanner had a Data General Nova 820 minicomputer with 32K of memory. It had a 2.5-MB two-sided hard drive, a reel-to-reel magnetic tape storage device, and a printer. The computer system and peripherals are estimated to have represented <10% of the approximate £100K cost of the system, which sold for $350 to 400K in the United States in the 1973 to 1975 timeframe ($2M in 2021 dollars).

The x-ray source was a fixed-anode oil-cooled tube that allowed a variety of settings, typically 100 kVp at 40 mA, 120 kVp at 32 mA, or 140 kVp at 27 mA. Most centers initially used the 120-kVp setting. The system scanned two adjacent 13-mm slices simultaneously using two sodium iodide (NaI) scintillators, each optically coupled to a photomultiplier tube (PMT), opposite the x-ray source. Imperfections in the detector, such as drift in gain and offset, were recalibrated at the start of each traverse, thus 180 times per scan. The system rotated one degree per linear traverse for a total 180 deg, gathering data points every 1.5 mm (160 samples) for each of two slices, storing that raw data on a magnetic disk. The scan time was 5 min for two slices.

In the early systems, the 28,800 data points were used to reconstruct an 80×80 image using Hounsfield’s iterative algorithm. The EMI image scale had water at 0 and air at −500, which established a relative attenuation scale. All other tissues had EMI numbers corresponding to their x-ray attenuation coefficients with dense bone around +500 EMI units. The early algebraic algorithm was later replaced with the faster convolution-backprojection method (with 1 mm sampling and 160×160 image matrix). The CT number scale was later expanded by a factor of 2 with air at −1000 and units of this expanded scale became a standard in the industry and were named for Hounsfield.

Reconstruction and display of two images took 5 min for two slices. One of the daily QA tests that was done from the outset was to scan the box of water and measure the noise (standard deviation around the water value), which measured the end-to-end noise in the system—including the photon noise and all secondary noise sources.

**Fig. 6:**
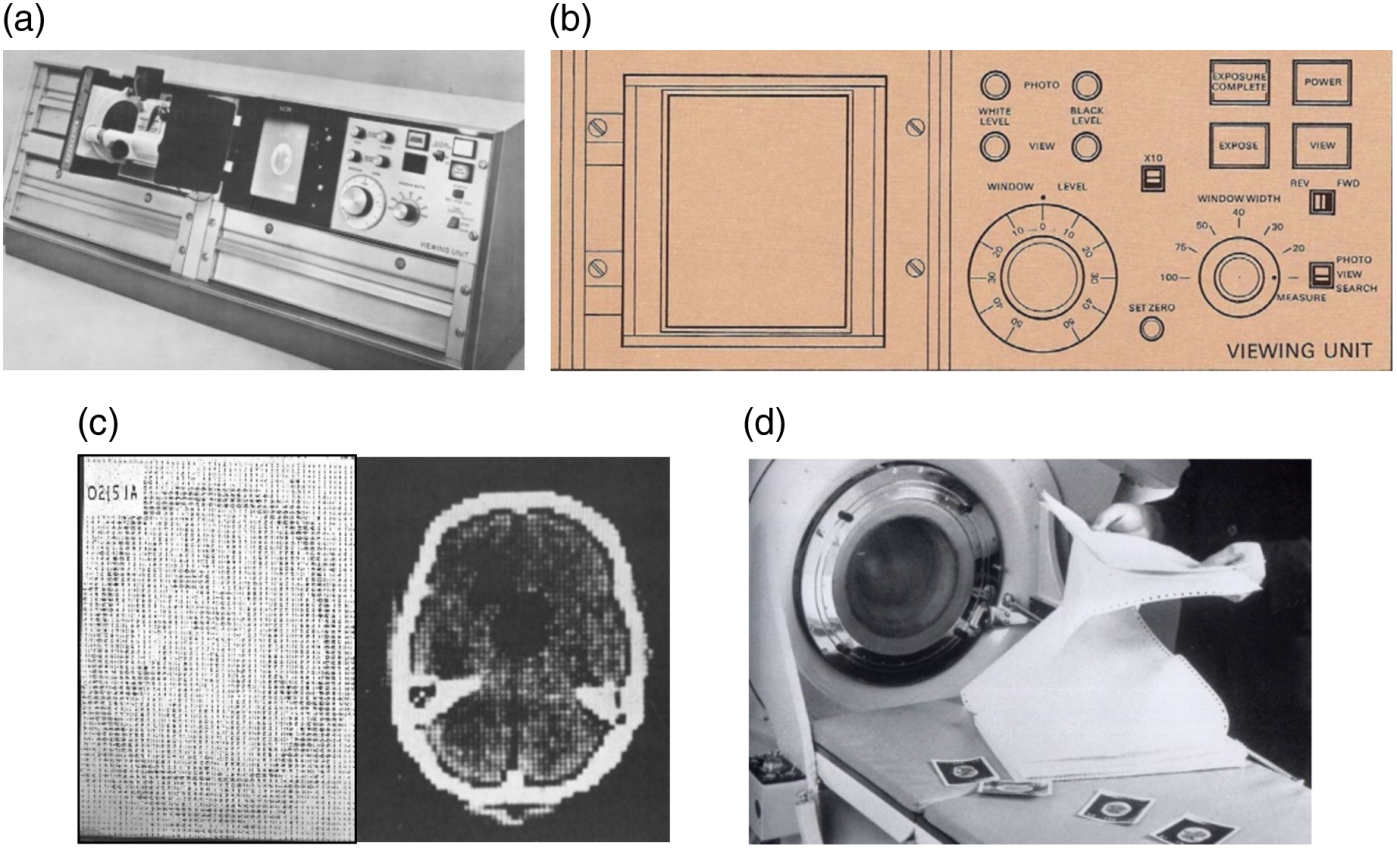
(a) The viewing unit of the EMI-scanner allowed setting of image window and level. The Polaroid camera hinge is mounted on a hinge to the left of the display screen. (b) A drawing of the controls. (c) Printout and gray scale image of the same slice. (d) Photo of researcher with printout and Polaroid images. Images from Refs. 26 and 28.

Because these images had such a wide dynamic range (−500 to +500) and the ability to measure subtle differences in attenuation, a technique had to be devised to select and map a portion of the dynamic range to the display. This was the first time a medical imaging system had such a wide range of discernable (i.e., statistically significant) image values and led to the introduction and first use of “windowing” and window width and window level controls. This required special-purpose hardware since computers and display devices did not have this capability at the time. The display console had dedicated knobs for this purpose (Fig 6). One allowed selection of seven discrete window width levels and the second selected level—the image value at the center of the window. The image values in the range level ±(width/2) were converted to 10 levels of gray on the display.

The EMI-scanner (retrospectively called the EMI Mark I) provided three types of image display: a numeric printout, a gray scale image on a display, and a hard copy, which was captured by a Polaroid camera that could be sealed over the display. The Polaroid images, chemically “fixed” with the Polaroid squeegee, were taken one at a time (Fig. 6). The patient’s record would consist of 8 to 12 Polaroid images (13 mm slices) taped into a folder. A typical scan would be scheduled for 60 to 120 min depending on the condition of the patient. For more detailed analysis, a printout of an image, with numbers from −500 to +500 could be included in the patient’s folder. Each would take ∼10  min to produce! To ensure the data was not lost, scans were transferred to magnetic tape. This was initially done at the end of each patient study.

## 6 Refinement of Translate Rotate Scanners, Second-Generation and the First Whole Body Scanners

By RSNA 1973, five scanners had been installed beyond Atkinson-Morley, three in the UK (Manchester, London’s Queen Square and Glasgow), and two in the United States. The Mayo Clinic EMI Mark-I system, delivered in early 1973, imaged the first patient outside the UK on June 18, 1973. About a month later, the MGH scanner was installed and operating. The first 10 EMI-scanners were partly hand built while manufacturing was ramping up.

**Fig. 7:**
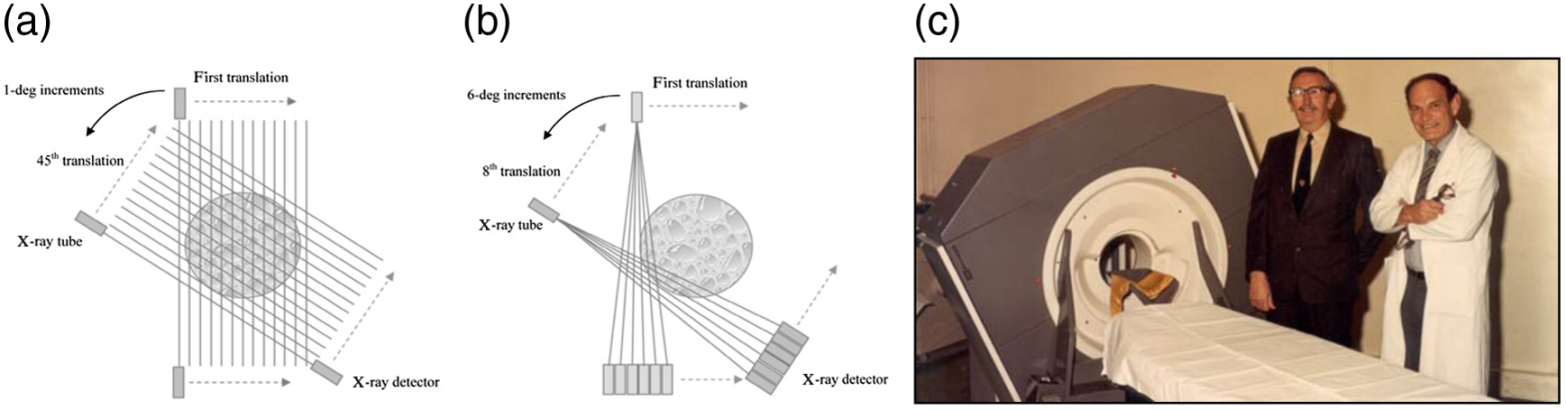
(a) First-generation CT geometry. In this example, the rotation increment is 1 deg. (b) Second-generation CT. In this example, six detectors span 6 deg, accelerating scanning by a factor of 6. (c) Hounsfield and Ambrose next to an EMI CT1010, an early second-generation head scanner. Graphic drawings are from Ref. 10. Image is from Ref. 26.

When the clinical power of CT became evident, demand increased as did efforts to improve the technology. In 1975, EMI introduced the CT1010 head scanner that replaced the water bag with a carbon prepatient attenuator. It still used a translate-rotate geometry with the capability to calibrate the detectors in each traverse, but it used eight detectors spanning 3 deg, allowing a 3-deg rotation increment and needing only 60 translations, reducing the scan time to 1 min. This design came to be known as second-generation CT (Fig. 7).

The growing market and enthusiasm also brought competition to EMI from companies adopting the first- or second-generation designs to quickly get to market. In 1974, Siemens and Hitachi introduced first-generation translate-rotate head scanners, with Siemens installing a Siretom at the University of Frankfurt, June 19, 1974. Ohio Nuclear, part of BCC (which became Technicare in 1973) and already successful in providing systems for the nuclear medicine imaging market, quickly developed the DeltaScan 50 whole body scanner (see below) and a less costly Delta 25 model, a head-only two-slice scanner, like the EMI system. A number of players new to diagnostic imaging, including pharmaceutical companies, entered the imaging field during the early years of CT. Syntex, a California drug company, introduced a head scanner. Neuroscan introduced a head-only scanner that GE licensed as a way to enter CT quickly. CGR, a major French medical imaging company, introduced an early head-only system. None of these were very successful in the marketplace.

Credit for building the first body scanner is often given to Robert Ledley, a Georgetown University dentist who, after traveling to the UK to look into buying an EMI scanner, returned to Georgetown and founded DISCO (Digital Information Science Corporation) to develop a first-generation body system (essentially a scaled version of the EMI-scanner) with a 5-min scan time, which was installed at Georgetown in 1974. Few were purchased and the design was sold to Pfizer. Ohio Nuclear installed a prototype of the Delta Scanner, a two-slice second-generation system (three detectors) body scanner in 1974 at the Cleveland Clinic.^29^ The DeltaScan 50 had a larger 256×256 image matrix and 2-min scan time.

As the number of detectors in the second-generation design increased, scan time decreased to the point that body imaging became feasible. Indeed, when Ledley visited, EMI was already far along developing a second-generation body scanner. The result of that work was the EMI 5000-series, a second-generation design with 30 detectors over 10 deg, with the translation and rotation of the heavy gantry reduced to slightly more than 1 s, achieving a scan time of 20-s to facilitate breath-hold imaging to reduce motion effects. In December, 1974, the EMI 5000 scanner was installed at Northwick Park Hospital, Middlesex, UK. The first two EMI 5005 scanners in the US were installed in October, 1975. Technicare continued to advance its designs and introduced a range of systems. In contrast to many of EMI’s competitors, the Delta Scan line of products was highly successful. They had more advanced software, user-friendly designs, faster scan times, and higher image resolution, and Technicare had a distribution agreement with Siemens. Demand was so high that, at the RSNA conference in 1975, customers lined up with $50K deposits to get in line for system delivery. After that year, RSNA forbade such transactions on the exhibit floor.

While these scan times were compatible with breath-hold imaging in many individuals, extracranial image quality was inconsistent due to random patient, organ, and peristaltic motion effects. The field needed even higher speed and the second-generation design could only go so far.

## 7 Quest for Speed, the NCI RFP, and “Third-Generation” Rotate-Rotate Scanners

With a wide enough array of closely spaced detector elements, CT scanning can be accomplished without any translation motion. Credit for this concept, later dubbed third-generation or rotate-rotate, is often given to Douglas Boyd, then at Stanford University.^30^^,^^31^ However, it was independently developed by others as well. Hounsfield had already conceived the idea, as shown in Fig 1(d) and detailed in his patents. David Chesler, a scientist at Massachusetts General Hospital, also had the design. Chesler and colleagues submitted a grant proposal to the National Institutes of Health to develop a CT body scanner. That proposal “Transverse Section X-ray Camera” was submitted in early 1973 and funded (CA015882) in November of that year.^32^^–^^34^ It included the design of a fan-beam body scanner with a 5-s scan time, filtered-backprojection reconstruction by rebinning to parallel rays, a prepatient attenuator to reduce dynamic range and beam-hardening artifacts (later called a bow-tie filter), and quarter-offset alignment to deal with the aliasing to which third-generation systems are prone.^13^

In late 1974, the National Institutes of Health/National Cancer Institute (NIH/NCI) issued a request for proposals (RFP) to spur the development of high-speed whole-body scanners. A number of collaborations between academia and industry responded to the RFP, and the competition resulted in an award to Columbia Presbyterian Medical Center/Neurological Institute of New York (CU/NINY) and two subcontractors including American Science and Engineering (AS&E),^27^ detailed in the next section. While the RFP resulted in a single award, it definitely accelerated the pace of CT development throughout the industry. Manufacturers who competed, as part of preparing their proposal, needed to create an R&D strategy. Then, the ones who were not funded knew that the field would advance, that the market was receptive, and that if they did not accelerate their R&D they would be left behind.

By the beginning of the second half of the 1970s, the third-generation design became the most popular strategy for fast body scanning. This included GE, Siemens, Varian, Searle, CGR, Artronix, Elscint, Philips, Toshiba, Hitachi, and Shimadzu.^13^ Other companies followed.

While we think of third-generation scanners as motivated by the goal of higher speed body imaging, some early systems were for anatomically specific applications. Artronix, which had its roots in computer systems for radiation therapy planning, introduced a head-only system (Neuro CAT)^13^ in late 1974 with a 12.8-s scan time that included a water bag. Artronix used their computer expertise to introduce a number of features: concurrent scanning, reconstruction and display, thinner (3 mm) slice capability, and volumetric reformatting.^35^^,^^36^ At around the same time, GE developed a scanner for breast imaging (CT/M), the first of which was installed at the Mayo Clinic in 1975. The patient was prone on the table with the breast immersed in a water bath; the system used a xenon ionization chamber with 127 channels and a 360-deg rotation time of 10 s. Among the clinical findings from the CT/M was the observation that the sensitivity for detecting breast malignancies was much higher after administration of contrast agents,^37^^,^^38^ setting the stage for much more recent experience with MRI and other modalities. It is ironic that, after so many years and granting that dedicated breast CT scanners have been developed, the only major body organ for which CT scanning is not in widespread use today is the breast. Both of those early third-generation systems used arrays of xenon ionization chambers (see below) for x-ray detection, and both companies went on to develop whole body systems.

At the time of the NCI RFP, GE initiated projects at its Corporate R&D Center to develop the CT/M breast scanner and a whole-body scanner (CT/T).^13^^,^^39^ After transfer of the project to GE Medical Systems, announced at the 1975 RSNA, the system acquired the name “7800.” The CT/T 7800 used a xenon ionization chamber with 289 signal channels plus two reference channels (details below), a pulsed x-ray source (at line frequency), and 360-deg rotation times of 4.8 or 9.6 s. The first prototype was installed at the University of California San Francisco (UCSF) in 1976. A modified version (CT/X) was installed later at UCSF. It had twice the rotation speed (360  deg/2.4  s), source pulsing (120 Hz rate), the ability to acquire data for a rotation of 525 deg, and a reconstruction algorithm that could produce an image from data over 212 deg, which was used for early work on dynamic scanning and cardiac imaging.^40^

**Fig. 8:**
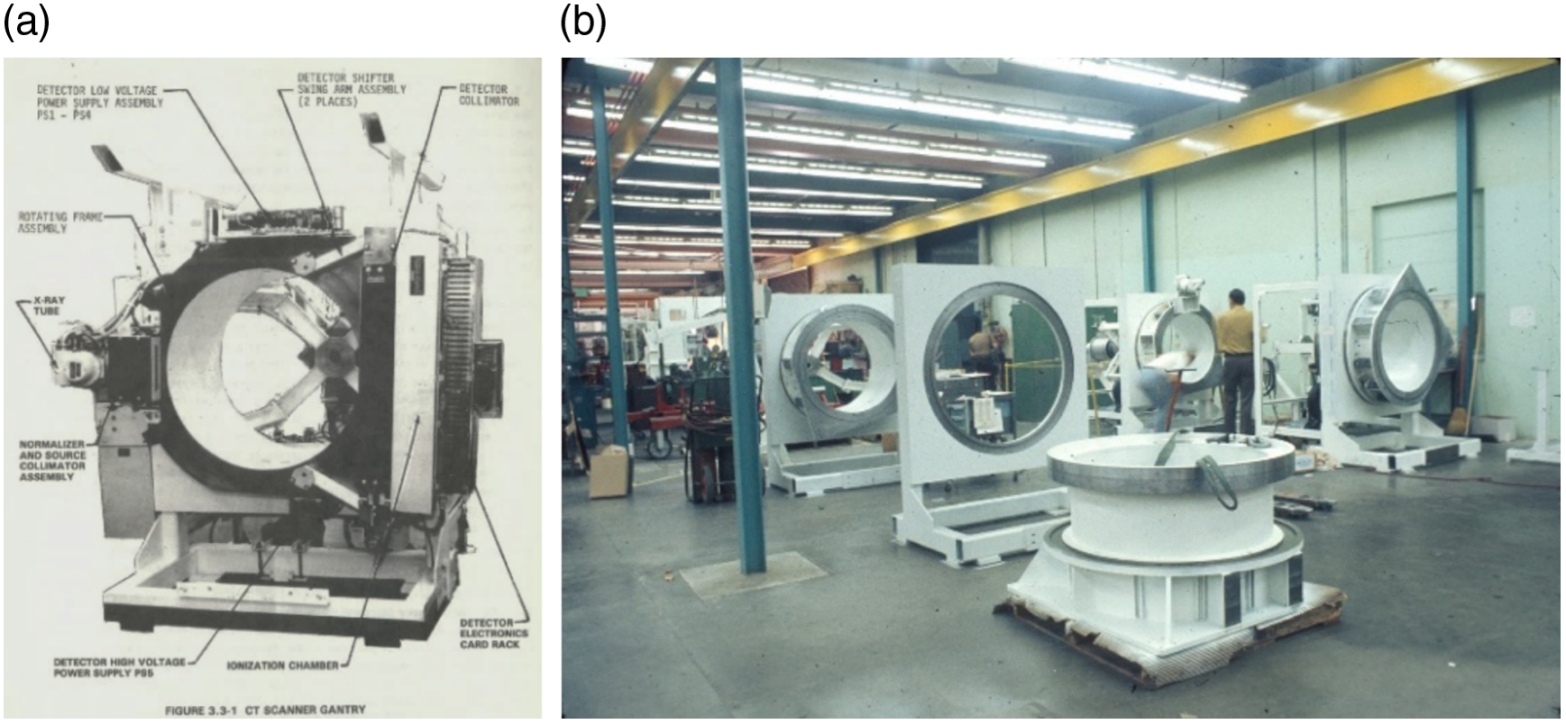
(a) Varian V-360-3 CT scanner with small high voltage slip allowing for continuous rotation, resulting in one side closed off. The high voltage power supply is below the rotating gantry. (b) Manufacturing floor at Varian Instruments. Images courtesy of Ed Shapiro.

Varian Associates developed a unique system, the first of which was installed at Stanford in late 1975.^13^^,^^41^ Early prototypes had a 6-s rotation time, but all production systems starting in 1976 were 3-s scanners. Like most of the other early third-generation scanners, it had a xenon ionization chamber (301 channels) and pulsed x-ray source, but unlike all the other systems of the time, it had a slip-ring that supported continuous gantry rotation. Unlike modern CT scanner slip rings that transfer power at low voltage and have a compact high voltage power supply on the gantry, the Varian system transferred high voltage power (Fig. 8). While the gantry had a wide opening, the slip ring had a rather small diameter, which required the system to have a tunnel into which the patient was placed. Constant rotation plus the ability to perform half-scan reconstructions enabled dynamic scanning with sub-3-s temporal resolution.^42^ The system also had shifter arms, shown in Fig. 8, that could move the detector during rotation to provide increased sampling, lower aliasing, and higher spatial resolution. The scanner enabled pioneering work on dual energy imaging.^43^

The Philips Tomoscan 300, introduced in 1977, also had a xenon detector but offered the unique feature of variable magnification. The source and detector, rigidly coupled to each other could be repositioned with respect to the center of rotation to use all the detectors for both small and large fields of view.^44^

Like other early third-generation body scanners, the Siemens Somatom, introduced in 1977,^45^ used a pulsed x-ray source and had a 4-s minimum rotation time. However, while all the other entrants used xenon ionization detectors, Siemens used an array of scintillators (CsI) coupled to photodiodes.^11^ One early Somatom model had two detector arrays with different cell spacings.^13^^,^^46^ Other unique features of the Siemens Somatom were its ability to deliver the reconstructed image immediately at the end of the scan and a very high heat capacity x-ray tube (1.3 M heat units), the combination of which produced a high-throughput machine.^13^^,^^45^^,^^46^ It used a special purpose array processor developed by Analogic, Inc., that could preprocess, filter, and backproject the detector data as it was acquired, thereby overlapping data acquisition and reconstruction, a feature termed “instantaneous image reconstruction.”^13^^,^^46^

In the following few years, the number of detector channels increased and the spatial resolution improved. For example, in 1977, GE introduced the CT/T 8800, which had 523 detector channels compared to 289 in the 7800 (over the same fan beam width). Calibration and reconstruction algorithms improved with important impact on image quality.

Besides faster imaging times, the development of CT systems with a fan beam wide enough to cover the entire field-of-view also enabled the production of radiograph-like images for localization and graphic scan prescription, often called scout images. These were facilitated also by collimators able to illuminate thin slices. To produce these, the gantry is held stationary while the patient is advanced through the beam.^47^^,^^48^ Scout scanning quickly became an essential feature of every CT system.

**Fig. 9:**
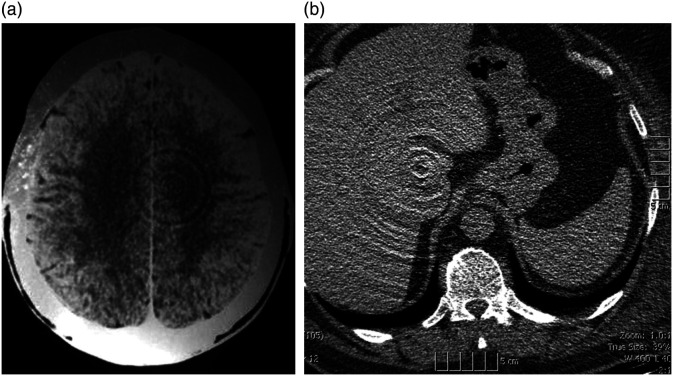
Initially, all third-generation scanners suffered from ring artifacts such as these. Head image courtesy of Dr. Laughlin Dawes, Radiopaedia.org, rID: 35901. Body image courtesy of Dr. Omar Giyab, Radiopaedia.org, rID: 22694. GE was the first to find a solution to ring artifacts.

While the third-generation architecture offered many advantages compared to earlier systems, it also presented important challenges. Principal among these is the tendency of these systems to produce ring artifacts (Fig. 9). In contrast to first- and second- generation systems in which each detector can be calibrated in each traverse, the detectors in third-generation scanners can only be calibrated when the patient is not in the beam. Errors in a single detector channel add constructively (to produce a full or partial ring) and the required detector linearity and stability is extremely tight.^41^^,^^49^ This problem was understood early on and caused concern among system designers.^50^ Another fundamental problem with third-generation systems in which the detector channels have little or no crosstalk is that the acquired projections can suffer from aliasing^51^ when the projections have spatial frequency content above the Nyquist frequency. Both of these problems motivated the search for a fast scanning architecture that could avoid these problems.

## 8 Avoiding Rings: “Fourth-Generation” Rotate-Stationary Systems

As mentioned above, the 1974 NIH/NCI RFP to build a high-speed whole-body CT scanner led to an award of approximately $1M (later increased to ∼$1.6M) to a group including the Neurological Institute at Columbia–Presbyterian Medical Center and American Science and Engineering (AS&E), with Sadek Hilal, MD, PhD, as a principal investigator. The team included Jay Stein, PhD, from AS&E who brought x-ray and detector experience and Larry Shepp, PhD, from Bell Labs who brought expertise in mathematics and algorithms. The proposal submitted to NIH^13^ included plans to build a third-generation scanner, but Stein and Shepp understood that such a system would be prone to ring artifacts from imperfect detector channels.^50^ Another reason AS&E chose to avoid the third-generation design was the difficulty of packing many large scintillator-photomultiplier detectors, a technology familiar to AS&E, into the small space required for good imaging optics and spatial resolution.

**Fig. 10:**
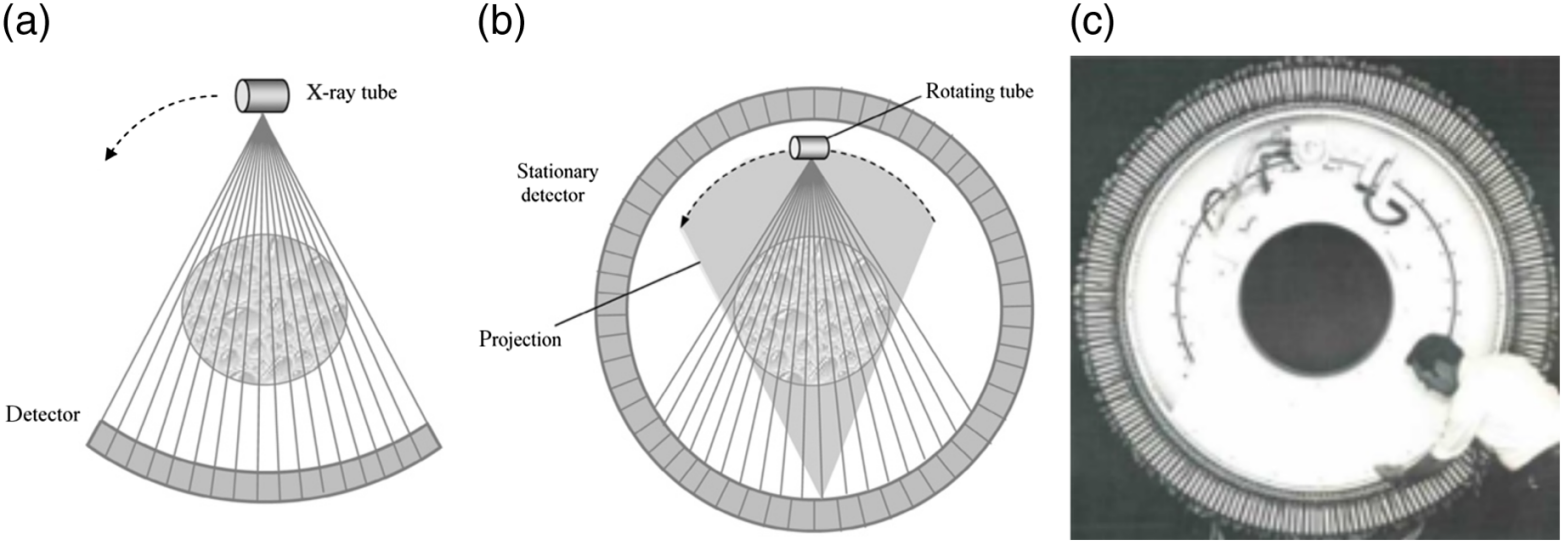
(a) Third-generation CT acquires data by rotation of a fan beam source rigidly coupled to a detector array. (b) In a fourth-generation scanner, the fan beam source rotates inside a stationary detector ring. A detector cell measures an inverse fan of data as the source rotates (shaded). (c) Image of the AS&E gantry. Graphic drawings are from Ref. 10. Image is from AS&E brochure.

Stein and Shepp, turned the problem on its head by breaking the rigid coupling of the tube from the detectors, creating a CT system with a stationary ring of detectors around the patient with a tube that rotates in the space between the patient and the detector ring (Fig. 10), termed fourth generation.^52^^,^^53^ In this schema, each detector could collect a continuous inverse fan of data as the tube rotates. Each detector could be calibrated at the beginning and/or end of each inverse fan view, and the design completely avoided ring artifacts. Also unlike in third-generation scanners, where the number of samples in a divergent projection was fixed, one could sample the inverse attenuation data in as fine increments as desired.^52^ While at the time third-generation scanners can collect an arbitrary number of views, the number of rays in each was limited by the number of detector cells in the array. By contrast, in the fourth-generation design the number of views is limited by the number of detectors but the number of rays in each inverse fan is flexibly controlled, an advantage for achieving high spatial resolution.

The fourth-generation design is not without challenges. For the same detector spacing, the fourth-generation design needs far more detectors compared to third-generation, only a fraction of which are used at any time. Because the beam enters a detector cell from many directions, the system cannot reject in-plane scatter. Also, the x-ray tube is closer to the patient, resulting in higher skin dose.

AS&E launched their scanner concept in March, 1976,^13^ and shipped the first unit in 1977 to Columbia University. It had 600 bismuth germanate (BGO)-PMT detectors, collimated to achieve higher spatial resolution (at reduced dose efficiency). It met the design goals of the NCI/NIH RFP and more, with 5 s scanning, sub-mm in-plane resolution, 512×512 image matrix, and slices as thin as 2 mm. Users of the scanner who were accustomed to the much larger voxel volume of the original EMI-Scanner were amazed: “this decrease in volume from 117 to 1.13 cu mm represents approximately a 100-fold increase in resolution.”^54^ Shortly after commercial introduction of the system, AS&E sold the design to Pfizer.

The development of the fourth-generation design with NIH funding led to an interesting intellectual property dispute. Patent applications were filed but at the time the federal government would take title to sponsored inventions. AS&E was advised to petition for an exclusive license, a request that generated controversy about whether doing so would be in the public’s interest. In the end, because of this and other patenting issues, no patents ever issued.

Even before the dust settled on the patent questions, some competitors adopted the fourth-generation design. Technicare launched the DeltaScan 2000 series and Picker International launched the Synerview scanners.^13^ Scan times were reduced to as low as 1 s for 360-deg rotations. While some had complete detector rings, the DeltaScan 2010 and the Synerview 300 had detectors that spanned 180-deg plus the fan angle to reduce cost.

## 9 Hybrid

While “conventional” third- and fourth-generation scanners were competing in the marketplace and were being further improved, several hybrid designs were developed. Based in part on the original architectures, these systems had modifications to mitigate weaknesses or achieve unique capabilities.

**Fig. 11:**
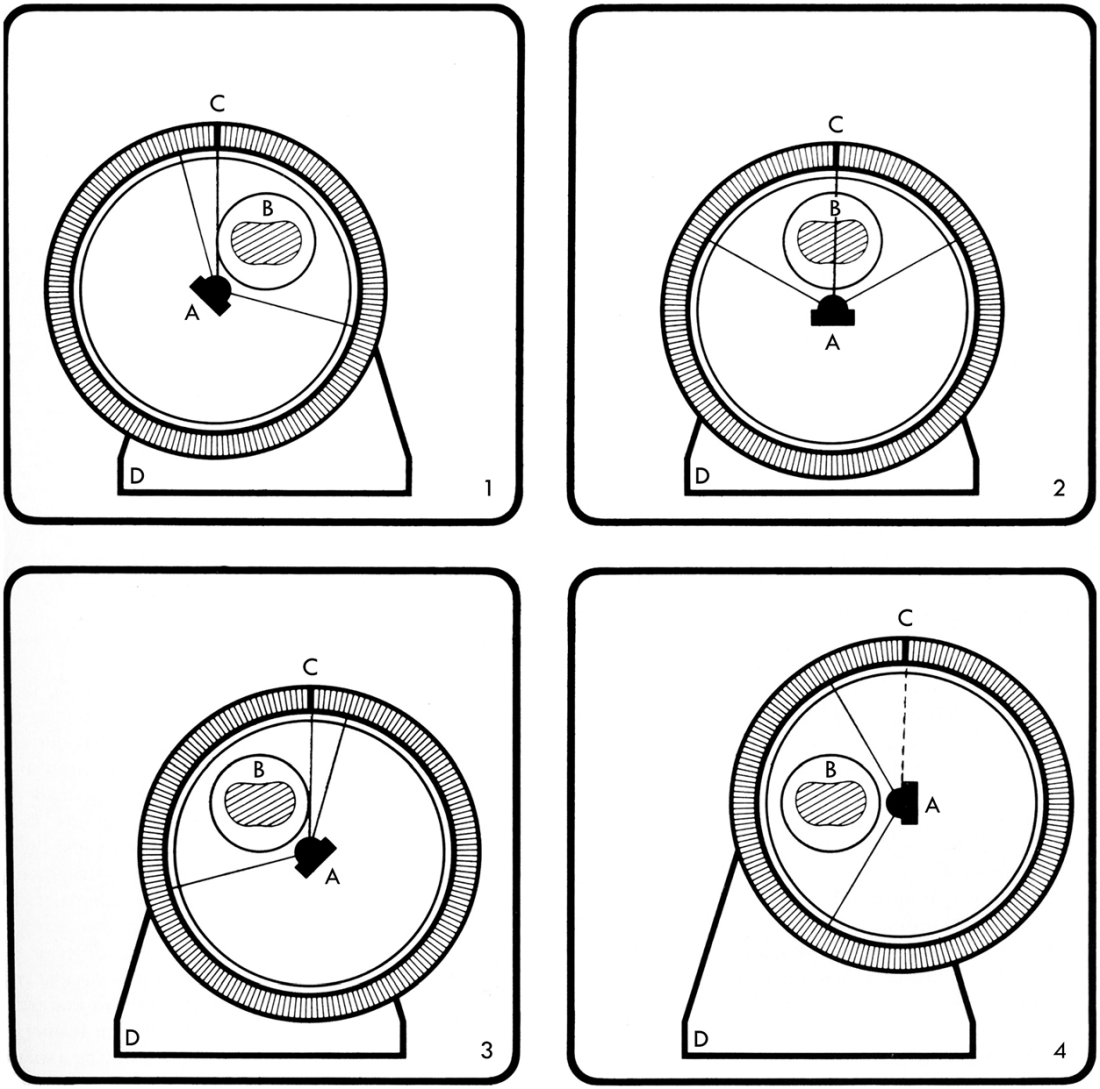
In the Artronix hybrid system, in the reference frame of the detector ring, the x-ray source (A) is at the center of the ring and rotates about that point. In the laboratory (and patient) frame of reference, the gantry rotates about the stationary patient (B). As the scan proceeds, each detector element (C) measures a parallel ray projection. Graphic from Ref. 55.

Artronix had developed a whole-body third-generation scanner that used a xenon detector but suffered from ring artifacts. They wanted to use a ring of detectors but sought to continue to use an ionization chamber and to retain the ability to reject patient scatter. In their hybrid design (Fig. 11) that used a “concentric x-ray source and eccentric detector ring,”^56^ the detector ring has cells that are focused at the center of the ring. The patient is located inside the ring but not at the center. The source focal spot is at the center of the ring and rotates in place, with the focal spot position staying in place but the direction of the fan beam rotating. At the same time, the entire assembly rotates in the laboratory frame, so the patient is stationary and the beam and detectors revolve around the subject. The design, which also allowed for continuous scanning, was creative but not successful commercially, perhaps due in part to detector cost and patient dose issues.

**Fig. 12:**
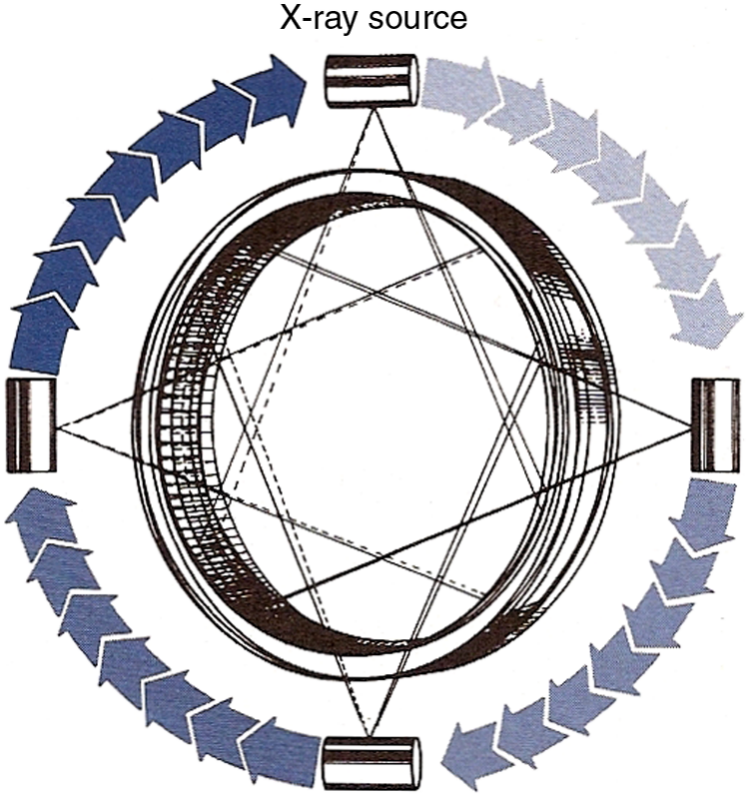
In the rotate-nutate design, the source rotates around the detector ring. As it does so, the side of the ring near the source tilts away from the scan plane, creating a nutating motion of the ring. Drawing from Ref. 7.

Based on phantom simulation work^52^ comparing third- to fourth-generations, the EMI group in Northbrook, Illinois, convinced management that the fourth-generation design was preferred because of ring artifacts in early third-generation systems. They sought a way to avoid some of the problems mentioned above and came up with the rotate/nutate concept. In this hybrid fourth-generation design, the x-ray tube is outside of the ring of detectors.^57^ This immediately solved many problems. Since the detectors are closer to the patient, it allows for both smaller detectors and a relatively smaller number of detectors for cost reduction. The reduced geometric magnification reduces focal spot blurring, which, combined with the smaller detector cells, leads to higher spatial resolution. In addition, the skin dose problem was reduced because the tube was no longer close to the patient. However, the detector ring cannot be in the way between the source and the patient. In the nutating design, the ring is tilted 2 deg so the detector region near the source is not in the way of the beam (Fig. 12). The first EMI 7070, which used 1088 CsI-photodiode detectors,^7^^,^^13^ was installed at Mallinckrodt Institute of Radiology in March 1979, but only 43 systems were sold before EMI exited the CT business (see below). The 7070 rotate/nutate concept re-emerged, first briefly as the Quad I from Omni Medical and more successfully later as the Toshiba TCT-900, of which more than 400 were installed.

Topaz was a hybrid third-generation system^20^^,^^22^ designed at EMI’s Central Research Laboratory. Hounsfield forecast ring artifacts early on and sought to avoid them. The Topaz used an electronically steered, flying spot x-ray source so that, as the patient was scanned, the focal spot motion was such that a ray through the patient was measured by two detector cells a small fraction of a second apart. The detectors could be continuously compared and calibrated. The system had 612 CsI-photodiode detectors including a central zoom region. While volumetric 1200×1200×270 images were achieved in 1980, the system was not commercialized by EMI before it closed its CT business.

**Fig. 13:**
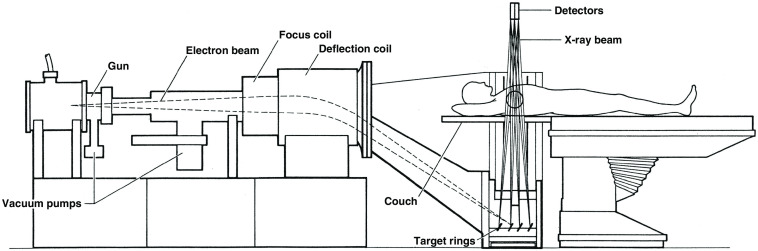
The electron beam is accelerated and steered to scan along one of four 210-deg tungsten anode targets, creating an x-ray source that rapidly rotates about the patient. The x-ray beam transmitted through the patient is measured by two 210-deg stationary rows of detectors. Reproduced from Ref. 58.

The Imatron C-100 Ultrafast CT (a.k.a. Cine CT or Electron Beam CT, Fig. 13) was another hybrid fourth-generation system and a major innovation. The commercial system, the result of many years of research at UCSF^59^ and introduced in 1981,^13^ offered unprecedented temporal resolution and required no motion at all to produce a set of parallel slices. The patient was essentially inside a large x-ray tube that had a 6’ semicircular anode and an electron beam that was electronically scanned in 33 to 100 ms over one of four anode tracks to produce a focal spot that rotated 210 deg. The other half of the system was two rows of 432 scintillator-photodiode detectors over 210 deg. The combination of four anode tracks and two detector rows could produce eight slices, two at a time.^13^ The design was intended to facilitate cardiac scanning and the system dominated that field for decades, until conventional CT scanners were able to image the heart reliably. It was also attractive for pediatric patients. Pioneering research with this system paved the way for coronary calcium scoring,^60^ among other applications. There were later versions, C-150 and C-300, with twice the number of detectors (864 per detector ring). In addition, in the C-300 all 1728 detector electronic channels can be used with a single detector ring that has finer pitch scintillators to offer a single-slice high-resolution mode with 100 ms scan time.^61^

## 10 Detector Technology

At the time that CT technology was developed, detectors using scintillators and photodetectors or high-pressure gas to convert high energy photons to a measurable signal were well-known and understood. Counting photons was out of the question for the CT application. The energy of many photons would be integrated in each measurement interval.

A scintillator (e.g., sodium iodide or calcium fluoride) coupled to a PMT was a very reasonable choice. The solid scintillators provide high quantum detection efficiency (QDE) and PMT’s can provide high noise-free gain. However, they are bulky and not inexpensive, and their gain can drift. These properties were compatible with translate-rotate scanners and these detectors were used in all first- and second-generation systems. These detectors were also used in early fourth-generation systems (see below).

**Fig. 14:**
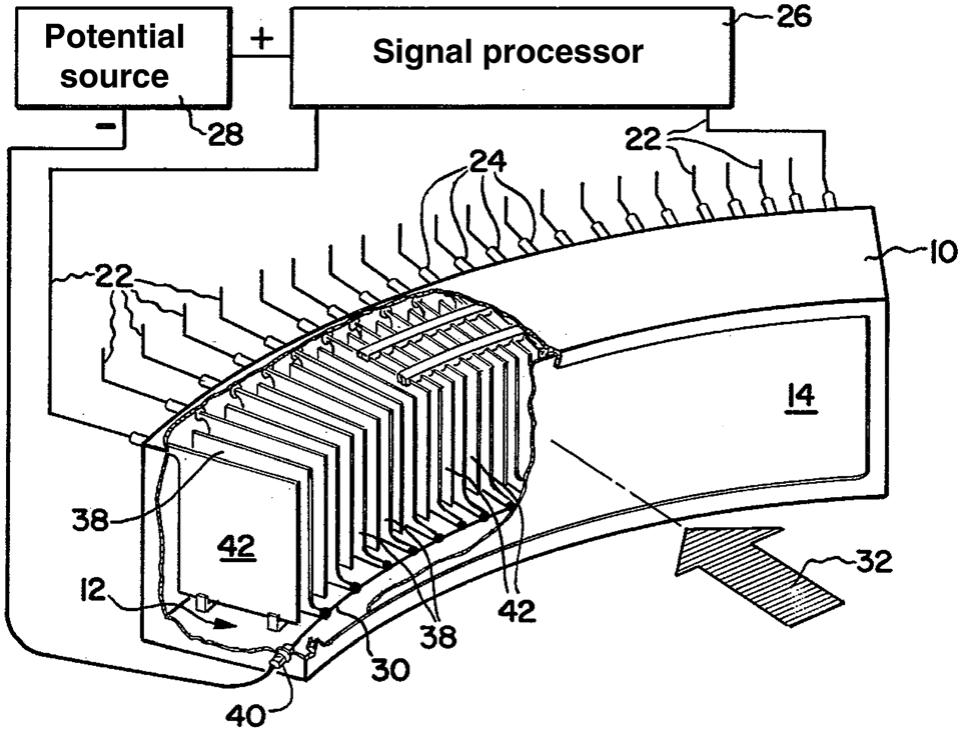
Drawing of a xenon detector array with tungsten plates, alternating anode signal plates and cathode bias plates. Reproduced from Ref. 62.

Ionization chambers can be quite stable and linear. If the electric field is high enough, the signal can be relatively insensitive to the bias voltage. To have reasonable detection efficiency, the ionization chamber must use a high atomic number gas at high pressure and be relatively thick (compared to a solid scintillator). Xenon at 20-30 atmospheres with cell depth of several centimeters can provide QDE on the order of 50% for diagnostic x-ray spectra. Pioneering work on these detectors was done by Whetten and Houston at GE.^62^ An important choice in the design of a multichannel ionization chamber for CT is the direction of the electric field compared with the fan beam and x-ray path. Having the electrodes above and below the fan-beam or front-to-back in the array would seem to be good choices but that approach presents problems. Designs eventually settled on systems with plates parallel to the direction of x-ray travel. This design easily achieves high field for efficient collection of ions and electrons and prevents cross-talk by stopping migration of characteristic x-rays produced by the xenon gas. In addition, the plates act like an antiscatter grid. The GE design had alternating signal and high voltage plates, with each detector cell comprising a signal electrode and the two bias electrodes on either side (Fig. 14).

As mentioned above, while the vast majority of early third-generation CT systems used xenon detectors, Siemens pioneered the use of scintillators coupled to photodiodes for third-generation scanners. PMTs were out of the question for high-resolution fan-beam detectors. Photodiodes are an alternative but, with the loss of the gain provided by PMTs, careful attention had to be given to the brightness of the scintillator (i.e., efficiency of conversion of x-ray energy to light) and the efficiency of light collection. The initial Siemens detector used cesium iodide (CsI) as the scintillator. One advantage of CsI is its brightness. Unfortunately, it suffers from afterglow and other problems. EMI was the first manufacturer to use scintillator-photodiode detectors in a fourth-generation design. BGO and cadmium tungstate were used in some CT systems as alternatives to CsI. These scintillators avoid some of the problems of CsI but are less efficient at converting x-ray energy to light, decreasing the detector gain. Eventually, scintillators with high speed and high brightness were developed and scintillator-photodiode detectors were used by every system manufacturer.

Neither xenon ionization chambers nor scintillator-photodiode detectors have inherent amplification. In general, they require tens of eV of x-ray energy to produce one measurable electron. As a result, the data acquisition system (DAS), with signal amplifiers and analog-to-digital converters, can be a challenge to ensure that the system has high dose efficiency and wide dynamic range. The DAS design was even more challenging for third-generation scanners since signal channels must have exceptional linearity and stability. Many system manufacturers reached out to Analogic Corporation, which had a reputation for high-quality signal digitization. For a number of years, Analogic produced DAS subsystems for virtually every CT scanner.

## 11 Competition, Cost Containment, and Consolidation

The early years of CT attracted numerous companies into the field, including new start-ups and companies that were not previously engaged in x-ray imaging. These players in turn brought innovative ideas that quickly advanced the field. The traditional imaging companies may not have been at the forefront early on, but success in CT became an existential issue to them,—they either succeeded or their business would falter—and all of them decided to invest in CT technology and entered the market. The authors on this paper were fortunate to be involved in CT in the early years and in the “Generations Battle.” More information on all the scanners and vendors discussed in this paper can be found in Table 1.^63^

**Table 1:** Evolution and consolidation of 20 scanner companies over a ∼10-year period. Companies are shown in groups: nonmedical, those in pharma or nuclear medicine before CT, and those with medical imaging businesses prior to CT. Number of units sold included only for the first two groups. H3^rd^ and H4^th^ reflect hybrid third- and fourth-generation systems (covered in Sec. 9) with significant modifications of the original system designs. Photos: https://www.radhis.nl/ct-generaties.html.

Twenty scanner companies in the first 10 years: 1971 to 1981								
Type-Date/								
**Nonmedical**	EMI (72-80)	DISCO (73-75)	AS&E (76-79)	Artronix (75-78)	NS (75-80)	Varian (75-80)	OmniMed. (77-84)	Imatron (81-01)
1971 to 1980/Type	1^st^, 2^nd^, H4^th^	1^st^	4^th^	3^rd^, H4^th^	1^st^ or 2^nd^	H3^rd^	2^nd^, 3^rd^, H4^th^	H4^th^
Model	Mk-I, 1010 (1^st^ and 2^nd^)	ACTA 0100	AS&E 500	Neuro CAT 1100	Neuroscan CT/N	Varian V360-3	CT 4001 (2^nd^)	Imatron C-100
	5000, 7070 (2^nd^ and 4^th^)			Torso CAT 1120			Quad-1 (4^th^)	Imatron C-150
							6000M (4^th^)	Imatron C-300
Highlight feature	Invented CT scanner	First commercial body	First 4^th^ gen.	First 3^rd^ gen.	—	First slip ring	—	First stationary
	First patent: 1 Oct. 1971		First 5-s scan			First 3-s scan		First subsec
	Nobel for Invention							33 to 100 ms
Number delivered	~1000 (~50 are 4^th^)	<5	5	31	<10	45	<20	75
Comment	Mark-1: Mayo, MGH: 1973	Georgetown, 1975	Columbia, 1977	—	—	—	—	—
	7070: Mallinckrodt: 1979							
Resolution/sale	IP+service: GE	IP+service: Pfizer	IP+Mkt.: Pfizer	Out of Mkt.	IP+service: GE	IP+service: GE	Out of Mkt.	IP+ Service: GE
**NucMed/Pharma**	Ohio Nuclear (74-77)	J&J/TC (77-86)	Syntex (75-78)	Searle (76-79)	Pfizer (79-82)	—	—	—
1971 to 1980s/Type	1^st^, 2^nd^	4^th^	2^nd^	3^rd^	1^st^, 2^nd^, 4^th^	—	—	—
Models	Δ50 Δ25 (1^st^	Δ2020, 2010, 2005	SS 60 & 90	Pho/Trax 4000 (3^rd^)	0150; 0200FS (1^st^, 2^nd^)	—	—	—
	Δ50, FS (2^nd^)	Δ2020 HR, Δ2060			AS&E450, Pz2400 (4^th^)			
Highlight feature	First 2-s scan	—	—	—	—	—	—	—
Number Delivered	~600	~350	20	20	<20	—	—	—
Comment	Δ50 highly successful	First 2020	—	—	—	—	—	—
	Took deposits at RSNA‘75	Cleveland Clinic, 1977						
Resolution/Sale	Technicare, J & J ‘79	IP+Service: GE’86	Roche: Bought ‘94	EMI Service ‘78	MktSer: Philips ‘80	—	—	—
**Medical Imaging**	Siemens (74-…)	GE (75-…)	Picker (75-01)	Philips (78-…)	Hitachi (76-…)	Toshiba (78-18)	Elscint (77-98)	CGR (76-87)
1971 to 2010s/Type	1^st^, 2^nd^, 3^rd^	1^st^, 3^rd^	2^nd^, 4^th^	2^nd^, 3^rd^	2^nd^	1^st^, 3^rd^, 4^th^	2^nd^, 3^rd^	1^st^, 2^nd^, 3^rd^
Models	Siretom 1, 2000 (1^st^, 2^nd^)	CT/N (1^st^)	Synerview 12 (2^nd^)	Tomoscan 100, 200 (2^nd^)	H-250 (1^st^)	TCT-20A, 300S (3^rd^)	Scan-Ex (2^nd^)	Densitome (2^nd^)
	Somatom 1, 2, (3^rd^)	CT/M (3^rd^)	120, 300, 600 (4^th^)	Tomoscan 300 (H3^rd^)		TCT-80A (H3^rd^)	Exel 905 (2^nd^)	CE10000 (3^rd^)
	Somatom DR, Plus4 (3^rd^)	CT/T 6800, 7800 (3^rd^)	Synerview 1200 (4^th^)	Tomoscan 500 (3^rd^)		TCT-400 (H4^th^)	Exel 2000 Sprint (3^rd^)	
		CT/T 8800, 9800 (3^rd^)	MX 8000 (4^th^)	CT Aura (3^rd^)		TCT-900S (H4^th^)	Exel CT Twin (3^rd^)	
Highlight feature	Instant reconstruction	First to solve ring	First 1-s scan	—	—	—	First multi-detector, plus 3^rd^ gen.	—
	1 M HU tube	artifact problem						
							plus 3^rd^ gen.	
Comment	Distributed Ohio Nuclear Δ50 & Δ25: 1974-1975. Analogic A/D in Somatom	First 7800: UCSF 1977	First 600: Cleveland, Univ. Hosp.: 1978	—	—	—	—	—
		First 8800: UCSF 1979						
Resolution/sale	Ongoing	Ongoing	SalesMkg: Philips	Ongoing	Ongoing	SalesMkg: Canon	SalesMkg: Picker	Merged w/ GE

Note: NS, Neuroscan; TC, Technicare.

74-89: 15-year patent battle between EMI, Technicare, AS&E, and GE; 1978: CON $100K law enforcement & SSA reimbursement restriction huge hit to all companies. Of the 20 companies in the CT business in the period 1971 to 1981, only four remain in that field: Siemens, GE, Philips, and Hitachi.

The successful development of fourth-generation CT by AS&E was a significant advance. The system had scan times far shorter than second-generation scanners. In response, Technicare, Picker, and EMI adopted some form of fourth-generation design. Third-generation systems were competitive in scan time, but all early third-generation systems had problems with ring artifacts. The origin of the rings was understood, and many predicted they would never be solved. The fourth-generation systems avoided these errors. They also could achieve high spatial resolution by collimating the detectors. To compete, third-generation scanners needed to solve the ring artifact problem and also decrease the detector pitch. The companies with third-generation designs were under tremendous pressure to quickly make technical improvements or pivot their strategy. Artronix moved to a hybrid fourth-generation scanner. Walter Robb, the CEO of GE Medical Systems, with advice from scientists in his business and at GE’s Corporate R&D Center, doubled down on the third-generation design, betting that the problems would be solved and probably risking his career and perhaps even the future of GE’s medical imaging business on the outcome.^64^^–^^67^ His bet paid off. With the introduction of the 8800, the improvements in detector quality and the implementation of sophisticated calibration, correction, and signal processing algorithms, GE solved the ring artifact problem and was able to achieve consistently high image quality. Siemens, Philips, and other third-generation CT vendors followed. The aliasing problem was also solved, first by quarter-offset alignment and later by focal spot wobble. Credit for quarter-offset is sometimes attributed to Brooks et al.,^51^ but it was suggested earlier by Peters and independently by Chesler.^13^^,^^32^ With these problems under control, the cost and other performance advantages of third-generation designs put pressure on companies that had selected other architectures.

Within a few years, the interest of the large x-ray imaging companies to invest in CT technology led to competitive advantages for them. Their long relationships with their customers, also took hold since many customers were generally more comfortable with commercial partners they had dealt with in the past, and from whom they could have broader business partnerships.

As the clinical impact of CT became evident, the market for CT systems increased and government entities and third-party payers pressed for limiting the number of systems sold. Joseph Califano, the US Secretary of Health, Education, and Welfare stated in 1977 before a Senate subcommittee, “We do not need a CT scanner in every hospital in this country….Every child does not need a Rolls Royce; every teenager does not need a Cadillac; every hospital does not need a CT scanner.” Fortunately, many others, including medical professionals, who had seen the impact CT could have in patient care disagreed with Mr. Califano. CT was eventually shown to be one of the most important medical advances.^68^ The US federal government already required hospitals to obtain a Certificate of Need for any large capital investment, and in 1978, it issued guidelines for the purchase of a new CT systems, causing a decline in the US market,^13^ which accounted for two-third of global sales. From a patient care perspective, the constraints led to long wait times for CT exams and had negative impact on the many patients who would have benefitted from CT studies. From a business perspective, they had a major impact on all vendors. They led to a rise in sale of systems to free-standing imaging centers and increases in the mobile system market, but these did not make up for the drop in sales to hospitals.^13^ While the tightened market led to increased share for some of the traditional imaging companies (e.g., GE and Siemens), it was especially hard on companies that had small share and in turn led to the departure of many of them from the market.

Early on, there were 20 companies in the CT business, beginning with “startup companies” who had little to no knowledge of the diagnostic imaging field. Next came companies that were in the medical or pharma field (Table 1). By the late 1970s, the number of players decreased and consolidation began.^69^ Varian exited the field in 1977 and sold its CT business and IP portfolio to GE. In 1978, Syntex and Artronix left the CT market, CGR did so in 1979 (CGR merged with GE in 1988), and Searle sold its CT business to EMI in 1978. None of these players had more than 2% share of the US market. Technicare, initially very successful with the second-generation Delta 50, was acquired by Johnson and Johnson in 1978 to provide financial stability during the CON downturn. As GE’s image quality improved, Technicare and GE “fought each other for every order.”^9^ Technicare faced losses due, at least in part, to the high cost of the 2000 series scanners. Their MRI business remained competitive but in 1986 Technicare was sold to GE. EMI merged with Thorn Electrical Industries, Ltd., in 1979 to become Thorn EMI, Ltd. Because of rapidly changing market conditions, product delays, and management changes, the EMI CT business was under stress and less than a year later (April 30, 1980) the company was sold. GE acquired the US CT parts and service business and some patents and most of the EMI Medical businesses outside the US, with the exception of Topaz, which was sold to Philips. Thorn EMI still held the substantial CT patent portfolio that was eventually licensed to all CT manufacturers, earning Thorn EMI many tens of millions of pounds sterling.^70^ By the early-to-mid-1980s, the CT market was largely in the hands of the traditional imaging companies. While the products from these companies had differences and business competition remained strong, many felt that the years of CT innovation were past. This opinion was supported by the concurrent large investments and advances in MRI. However, the predictions that CT innovation were over were clearly wrong. Indeed, CT was poised for dramatic advances that continue to this day. Newer technologies that drove these advances, such as spiral or helical, multislice, wide-cone, dual-energy, and photon counting CT, are discussed in a companion paper in this JMI Special Section.^71^

## 12 Concluding Comments

On October 11, 1979, almost exactly 8 years after the first patient’s CT scan at Atkinson-Morley Hospital, it was announced that the Nobel Prize in Physiology or Medicine would be jointly awarded to Allan Cormack and Godfrey Hounsfield for the “development of computer-assisted tomography.”^72^ The announcement reported: “It is no exaggeration to state that no other method within x-ray diagnostics within such a short period of time has led to such remarkable advances in research and in a multitude of applications.”

It is remarkable that neither Hounsfield, an engineer, nor Cormack, a physicist, the two recipients of the 1979 Nobel Prize in Physiology and Medicine, had a doctorate in any field of medicine or science, or really a background in physiology and medicine. The development of CT also led to a new unit of measure, the Hounsfield unit (HU). The disruptive initial innovation that led to the tremendous advance that is CT was the work of individuals, especially Hounsfield, who came from outside radiology. While modern CT scanners are getting closer to the in-plane spatial resolution of radiography and fluoroscopy, that level of spatial detail was simply not possible in the early 1970s. Computed tomographic imaging therefore required one to be willing to accept far lower spatial resolution (e.g., 3 mm as opposed to 0.3 mm) for the potential benefits of solving the superimposition problem and for the sensitivity to small density differences. It seems that those in the diagnostic imaging field at the time were unwilling to make this compromise. The temporary abandonment of CT work at Siemens in the late 1960s is one example, but those individuals were not alone. Many throughout the radiology field were of the opinion that such low spatial resolution was a nonstarter. Hounsfield (and Cormack) was not limited by the same preconceived ideas and faced skeptics from experts in conventional radiology. The work that Hounsfield triggered led to a dramatic change in our field. Since the initial disruptive innovation, progress was made by many, driven by scientific creativity and industrial competition, each leading to significant incremental improvements that have accumulated to enable amazingly sophisticated images in 2021.

The development and adoption of CT arguably marked the beginning of a major transformation of diagnostic imaging and of radiology as a field, from analog imaging largely involving film, to digital imaging in which pixel values are calculated by algorithms, and the beginning of imaging in which computers play a major role. Certainly, computers had started to be used in radiology and nuclear medicine prior to CT (e.g., Ref. 73) and ultrasonography was generating images algorithmically, but those were exceptions. Radiologists were not familiar nor comfortable with computers. Even CT (and MRI) images were, for many years, printed on photographic film and read using film-changers. The field has changed dramatically. In hindsight, given the total conversion of radiological imaging from film to digital, it is remarkable that the first digital x-ray imaging technology that came into widespread use was an entirely new modality, CT. That said, the significant film budgets for film use by CT and MRI undoubtedly contributed to the adoption of Picture Archiving and Communication Systems (PACS).

The rapid development and deployment of CT would not have progressed as quickly without forums at which technical and medical practitioners could exchange ideas and report their advances. One was the International Symposium and Course on Computerized Tomography, organized annually starting in 1975 by Drs. Juan Taveras and Paul New. Another was the *Journal of Computer Assisted Tomography*, established in 1977. Both included technical content contributed by physicists and engineers with contributions on new applications and clinical use provided by medical specialists. In some respects, this approach was followed by MRI some years later and continues to this day at the ISMRM.

It can be argued that the success of CT paved the way for MRI and other complex and expensive medical technologies. While some individual companies were not successful in CT, the industry as a whole was. Further, many academic careers were advanced, and most of all, patients greatly benefitted. This was a roadmap for future opportunities, like MRI.

The very early rapid development of CT would not have happened without government funding of research in industry labs—private–public partnerships. Two significant examples are the limited funding provided to EMI by DHSS in the UK and the NIH RFP for the development of fast body CT. In both cases, the relatively modest funding by the government led to much, much larger investment by companies, both those that received the initial funding and also their competitors, spurring competition and innovation. By contrast, the brakes applied to the growth of the CT market by governments in the United States and elsewhere (e.g., CON) while understandably motivated by the desire to ensure that health expenditures are well-spent, slowed down the growth of technology and led to the departure of companies from the field. That said, it is likely that the consolidation of the CT market would have happened at some point anyway.

We expect that readers of this paper may have their own stories and memories they would like to share. Readers interested in doing so can submit their contributions in an email to spiejournals@spie.org, with “CT@50” in the subject line. They will be collected and published in this journal.

The work and experiences described in this paper as well as in companion papers in this JMI Special Section led to the development of a new imaging technology. In 2020, in the United States alone, there were ~65,000,000 CT procedures performed on 14,000 units in 9700 centers,^74^ positively impacting the health of many, many patients.
