# Supplementary material for: How CT happened: the early development of medical computed tomography
Source: J Med Imaging (Bellingham). 2021 Oct 29;8(5):052110. doi: 10.1117/1.JMI.8.5.052110 (PMC8555965; doi:10.1117/1.JMI.8.5.052110)
Supplement: Supplementary file 1 [file JMI_008_052110_SD001.pdf]

Supplemental Material for

"How CT Happened – the early development of medical computed tomography"

Raymond A. Schulz[a], Norbert J Pelc[b], and Jay A. Stein[c]
[a] Varian, A Siemens Healtineers Company, Palo Alto CA 94304, USA
[b] Department of Radiology, Stanford University, Stanford CA 94305, USA
[c] Hologic, Inc., Marlborough MA 01752, USA

**Content**	**Page**
Hounsfield 1968 proposal "An Improved Form of X-Radiography	2
Programs from 1972 presentations at BIR and Montefiore	18
Hounsfield report on visit to New York and Boston May 1972	19
Minicomputers - critical component for success	23
Early work at MGH	24
New EMI machine brochure, May 1972	25
EMI New Perspectives brochure, RSNA, Nov 1972	29
GE CT/T brochure (cover only)	45
AS&E CT brochure 1976	46
Varian Associates Inc-1976	61
Pfizer AS&E CT Ad 1978	62
Picker Synerview 1sec Ad 1978	63
Siemens Somatom Ad 1978	64
EMI CT 7070 brochure 1978	65
Technicare Deltascan 2020 brochure 1979	72
Elscint CT Twin brochure 1993	84
Hounsfield speech at Magnus school 1983	91
Some drivers of CT market changes	96
CT U.S. Sales 1973-1988	97

Return to G. N. Hounsfield
2872

Proposed Project:

AN
IMPROVED FORM
OF
X-RADIOGRAPHY

Proposed Project:

AN
IMPROVED FORM
OF
X-RADIOGRAPHY

RESEARCH LABORATORIES

Electric & Musical Industries Ltd
Hayes · Middlesex

1967

RESEARCH LABORATORIES OF ELECTRIC & MUSICAL INDUSTRIES, LTD., HAYES, MIDDLESEX.

PROPOSED PROJECT: AN IMPROVED FORM OF X-RADIOGRAPHY

1. INTRODUCTION

The purpose of the study is to investigate the employment of a computer to make better use of the information obtained when an object is examined by gamma rays or X-rays.

It is well known that when an X-ray picture is taken through an object the three dimensional interior must be shown as a two dimentional picture. Hence all details from front to rear appear superimposed one upon another and a confused picture results; indeed, any 'submerged' object usually has to be comparatively thick to be seen at all. As an illustration, if the object to be studied was one such as a book, normal methods of X-ray pictures would reveal little of the content, because the information on, say, a middle page could not be extracted from the confusion caused by all the other pages in front of and behind it. However, it is hoped that the system under investigation would be capable of extracting the information from one page (or slice) only, thus presenting a map of all the information contained in that slice only, irrespective of that on the pages on either side of it. This concept is illustrated in Figures 1 and 2 on page .

In Figure 1 the normal X-ray technique is shown producing a confused and fuzzy picture of all the objects in the path of the X-ray beam AB, whereas in Figure 2 the proposed system produces a clear outline of all submerged objects within the body.

If the slice is divided into a mesh of say 20 thousand 1 mm cubes.

2. DESCRIPTION OF THE SYSTEM

Figure 3 illustrates the scanning system. The object to be examined would be scanned in one plane only by a very narrow beam of ~~gamma rays~~* emitted by source A not only linearly across the plane in a direction X, but at all angles as illustrated by the 2nd, 3rd, etc. scans.

The ~~gamma rays~~* which penetrate the object ~~would~~ be detected by an accurately aligned collimator and sensing device, B, which ~~would~~ always be pointing towards the source of the gamma rays. The readings from the detector taken "round the edge" of the object ~~would be~~ digitised and fed to a computer for processing. If sufficient scans ~~and angles of scan~~ are made there should be enough information from the '~~edge~~' readings ~~in~~ the detector to produce sufficient equations to calculate by computer the value of ~~transmission~~ of each cubic millimetre of material within the slice (i.e. there would be more equations than variables). A ~~crude~~ picture could, therefore, be built up in matrix form ~~of the absorption~~ of the material within the 'slice'.

* or any other rays used for diagnosis.

3. ADVANTAGES OVER THE CONVENTIONAL X-RAY EQUIPMENT IN THE MEDICAL FIELD

The principal application would be for detecting tumours and suchlike tissues which are likely to vary from a minimum of 1 cubic centimetre to a maximum of 60 cubic centimetres in volume. A high definition would therefore not be called for and it is accordingly possible to concentrate on accurate absorption readings.

The importance of this new system lies in the fact that the calculated absorption values are 100% due to the material constituting the tumour, whereas in the conventional X-ray picture they represent the mean absorption of all the material along the line of the penetrating rays (line AB in Figure 1) of which only a very small percentage will be due

to the tumour. This is very important since tumours may only absorb 5% more gamma rays or X-rays than the normal healthy tissue round them, and therefore higher accuracies of detection are very much in demand.

~~Further~~ advantages of such a system would be:

(1) In general the system makes better use of available information which is presented in the form of ~~more~~ accurate absorption readings, and an ~~increased number~~ of pictures for the same dose of radiation to the patient. The tones and detail of the picture would not be obscured as in normal X-ray pictures by other confusing information being printed on top (equivalent to, say, 40 superimposed pictures).

(2) Absolute values of absorption could be plotted accurately for each cubic centimetre of material within the slice (if necessary these could be plotted as numbers for comparison with each other). The 'contrast' of the picture could be arranged so that the full black to white range represents a window of very small ranges of absorption.

4. COMPARISON WITH TOMOGRAPHY

The following paragraphs describe how a simple comparison can be drawn between the proposed system and tomography, and indicate that it must be possible to obtain more information and better accuracy, for a given dosage, by means of the system proposed.

Figure 4A illustrates the usual movement of the plate and source in tomography. The line A B is on a row of elements through the slice to be viewed, which would be perpendicular to the paper. The shadow of these elements would be kept stationary on the photographic plate whereas areas at O and P would move and blur; and information concerning these areas is therefore lost. For convenience, the beam from the X-ray source is shown as a multiplicity of beams 1 cm in diameter and the elements to be measured 1 cm^3. If the body

is 40 cm thick, then 1 cm^3 of material in the slice would influence only a small proportion of the X-rays arriving at the plate - approximately 3%; the rest of the rays arriving produce fogging or blurred images superimposed upon the picture.

It is possible to replace the plate with a series of detectors (at a, b, c ...) approximately 1 cm apart and to take readings at a number of angles (representing say 1 cm of movement of the source) as the source is rotated around the body. The sum of the readings on each detector during one partial rotation of the source would then produce a tomograph of AB equivalent to the normal method, and with no extra radiation required. The two systems are therefore comparable. However, it can be seen in Fig. 4A that there is a whole series of lines above and below the line AB, e.g. CD and EF, which, if the readings from the detectors are chosen in a certain order and added, would produce a different section through the body. The whole body therefore can be covered by a series of calculated tomograms with the dose required only for one tomogram produced in the normal way. A possible method of removing blur might be as follows -

(a) Produce tomogram of line AB.

(b) Calculate the blurring of this tomogram from the information contained in all the other tomograms on either side of it, i.e. areas O and P.

(c) Subtract (b) ~~and~~ from (a).

This method of producing a more accurate tomogram is however impractical. It only serves to show in a simple way that information is avalable for obtaining:-

(1) Considerably more pictures for the same dose.

(2) More accurate absorption readings with "blurring" and "fogging" removed.

In practice, it is easier and more accurate to calculate the picture from the readings as described in paragraph 2 above , as it can be seen that Fig. 4A is only a special case of Fig. 3, the scan in the latter being shown as parallel lines rotated through 180° whereas the former scans over approximately 90° with slightly converging lines. The proposed system is therefore a method of producing an idealised type of tomogram.

Note

Methods of producing a number of pictures at the same time in tomography by using more than one plate must result in an overall reduction of information on each plate as the available photons must be shared by the plates.

5. TECHNICAL CONSIDERATIONS

The method described at the beginning of this paper illustrates a simple system. A study has been made of the practical problems of this system and the following conclusions have been drawn.

5.1 Choice of Source

(1) Low energy gamma sources (such as Americium 60 keV) produce an ideal single line spectrum at the correct energy level, but have insufficient intensity of radiation. It would take many hours to produce a picture from these sources.

(2) Higher energy sources (such as Caesium 137 - 600 keV) are a considerable improvement; the production of a picture would take less than one hour but the separation and detection of the various tissues of the body is not as good as the low energy X-rays or gamma rays.

(3) X-rays. There is theoretically sufficient intensity to produce a series of 40 pictures in less than one minute, but to handle this rate of information a special array of linear detectors would have to be developed (described later). However, using a bank of 10 scintillation counters at present obtainable, a useful picture could be obtained in 3 minutes. A picture with the maximum possible information would take 1 hour. or using a linear detector 3 minutes.

It is well known that X-ray sources have considerable spread over the energy spectrum and this could complicate the computer program. It may be possible that the source would have to be calibrated by means of a "phantom" wedge and the results fed into the computer for reference. Experiments would have to be conducted to deduce the limits of accuracy of such a system.

5.2 Detectors

For most laboratory and prototype machines scintillation counters would be adequate. However, their use is restricted by an upper limit of the rate of counting; they are costly and the number used per equipment must therefore be restricted. Both these factors must increase the time taken to obtain a picture. It can be shown that the accuracy of the detector need be no greater than 1 part in 1000 to produce approximately 2% accuracy of absorption reading on the picture. The range of accuracy need cover only 1/3 of the full range of the detector. It may be possible therefore in the future to use analogue methods of proportional detection from a bank of semiconductors*(see Fig. 4B). The output of each detector could be integrated and electronically scanned sequentially to reduce the complexity of equipment (variations of d.c. levels and gain could be corrected by the computer).

One half rotation of 180° would be required (Fig. 4B) to produce 40 "slices" in considerably less than 1 minute. Such a device must be considered as a possible future solution to the problem of excessive time to obtain a picture.

Picture time approx 3 mins.

* As an interim measure one scintillator and photomultiplier could be used in a linear mode of operation but later semiconductors may be developed to fit this application.

5.3 Definition

If a one centimetre wide beam is chosen, a resolution better than 1 cm could be obtained; for example, if the shape of the cross-section of the beam is known, at least three extra values may be computed from this information. Hence it may be possible to build up a picture with a 120 x 120 matrix instead of 40 x 40. However, the most accurate readings would only be produced in areas greater than 1 cm^2.

5.4 Compton Scatter

Compton scatter presents no problem with a simple system using a single detector and collimator. However, should multiple detectors be used (above 10) it would be necessary to correct for this effect, and a system to achieve this has been worked out.

6. RESEARCH PROPOSALS

A simple test jig could be made in which the object is rotated in front of a fixed radioactive source and collimator, and detected by a fixed scintillation counter and collimator. Blocks of material of known absorption could be arranged in various patterns within the beam and readings taken through them at known angles. These could be compared with the calculated values of absorption and accuracy assessed of the picture that would have been obtained. If the assessments are favourable, readings could be fed into a paper tape punch. A program for accepting the information and reconstructing the picture could be written and used on the 1905 computer. This being only a practical experiment, the time taken to form a picture would be very much slower than would be the case in normal use. Phantoms of tumours of varying size and density could be presented to the machine and the pictures produced compared with normal X-ray photographs and tomograms of the same phantom.

A theoretical study to overcome the computing problems associated with the broad spectrum produced by an X-ray tube could also be included.

7. APPLICATIONS

There are two main applications for the equipment:

(1) In Hospitals, Clinics and Medical Centres for the early detection of tumours when symptoms indicate that such may be present.

(2) For fitting in static and mobile Mass Radiography Units used for "screening". In this case a bank of detectors used side by side so that many picture slices may be taken through the patient at the same time would be essential. The digital information presented is in a form such as can be compared in a computer with other pictures by pattern recognition techniques, and by this means it could deal with the large amount of information that would come from Mass Radiography Units of this type.

8. CONCLUSIONS

The theoretical studies conducted so far have indicated that present methods of tomography do not use the majority of information available to the detector (film). It is theoretically possible to improve the accuracy of detection of absorption within the body by at least an order. At the same time at least 20 times as many pictures for the same amount of radiation through the body could be obtained.

Although scintillation counters are very time consuming they would be quite suitable for accurate systems where the 'rate' of taking a picture is of low importance. They could also be used in cases where a minimum radiation dose is necessary, when a smaller number of counts would be taken for each reading (which would also increase the picture rate). This would reduce the accuracy of detection but it could still be kept as good as a conventional tomogram. However, scintillation counters are not suitable for faster systems in which 40 "slices" would be required in one minute. In this case it is hoped that in the future a bank of linear detectors could be developed to fit this requirement.

At the moment it is necessary to prove that the theory works in practice with currently available components, irrespective of how long it takes to produce a picture.

If the proposed system can be proved successfully, it would be a very considerable incentive for the component manufacturers to produce components capable of speeding up the process.

9. ESTIMATED COST

It is estimated that £10,000 would be required to cover the work involved in proving the system.

B.653/2
08314/R/GNH/JPC
7.10.68.

APPENDIX Table of Comparisons between existing Systems and Proposed System of Radiography

	Normal X-Ray Picture	Tomography	Proposed System
Accuracy of absorption reading 1 cu cm of material	High accuracies of detection impossible. 1 cubic cm of material controls only 3% of radiation received through the body. A random distribution of material in line with it varying ±1% could obliterate all possibility of detection.	Picture obscurred by "blurring" and fog, e.g. if readings through the body vary randomly by 5% then the "blurring" produced by this would allow the tumour to be detected to an accuracy of 30%.	Accuracy of detection is theoretically better than 1%. The accuracy of these readings is practically independent of the nature of the material which surrounds a given element.
Definition minimum size of picture elements.	Better than 0.1 mm	Approx. 1 sq. cm.	1 sq. cm detected accurately. 1/9th sq. cm would be defined less accurately. Picture matrix 120 x 120
Ability to deduce absolute values of absorption.	Impossible to deduce absolute values.	Vague comparison of absorption with the mean value of the rest of the body.	Absolute values of absorption can be plotted for each cubic cm of material.
Number of pictures produced for a given radiation dose.	One	One (or 3 less accurate pictures).	Theoretically between 20 and 40 pictures.

APPENDIX Table of Comparisons between existing Systems and Proposed System of Radiography

	Normal X-Ray Picture	Tomography	Proposed System
Accuracy of absorption reading 1 cu cm of material	High accuracies of detection impossible. 1 cubic cm of material controls only 3% of radiation received through the body. A random distribution of material in line with it varying ±1% could obliterate all possibility of detection.	Picture obscurred by "blurring" and fog, e.g. if readings through the body vary randomly by 5% then the "blurring" produced by this would allow the tumour to be detected to an accuracy of 30%.	Accuracy of detection is theoretically better than 1%. The accuracy of these readings is practically independent of the nature of the material which surrounds a given element.
Definition minimum size of picture elements.	Better than 0.1 mm	Approx. 1 sq. cm.	1 sq. cm detected accurately. 1/9th sq. cm would be defined less accurately. Picture matrix 120 x 120
Ability to deduce absolute values of absorption.	Impossible to deduce absolute values.	Vague comparison of absorption with the mean value of the rest of the body.	Absolute values of absorption can be plotted for each cubic cm of material.
Number of pictures produced for a given radiation dose.	One	One (or 3 less accurate pictures).	Theoretically between 20 and 40 pictures.

NORMAL X-RAY TECHNIQUE.

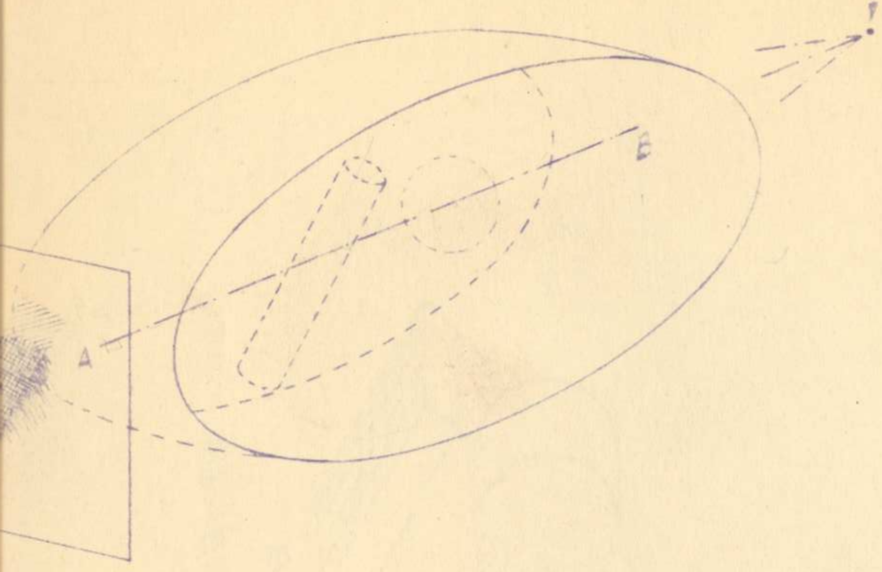

FIG. 1.

~~PROPOSED~~ SYSTEM

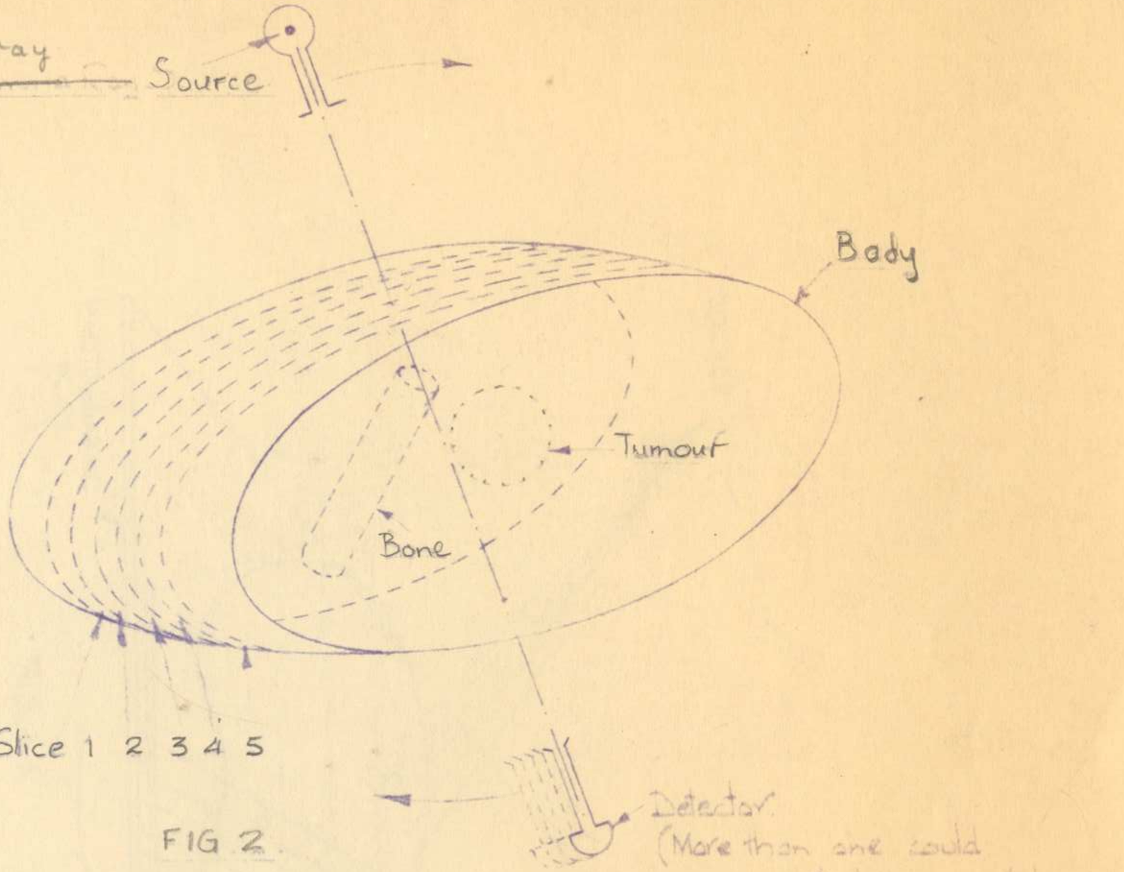

FIG 2.

Full range of tones 5% of total range of readings

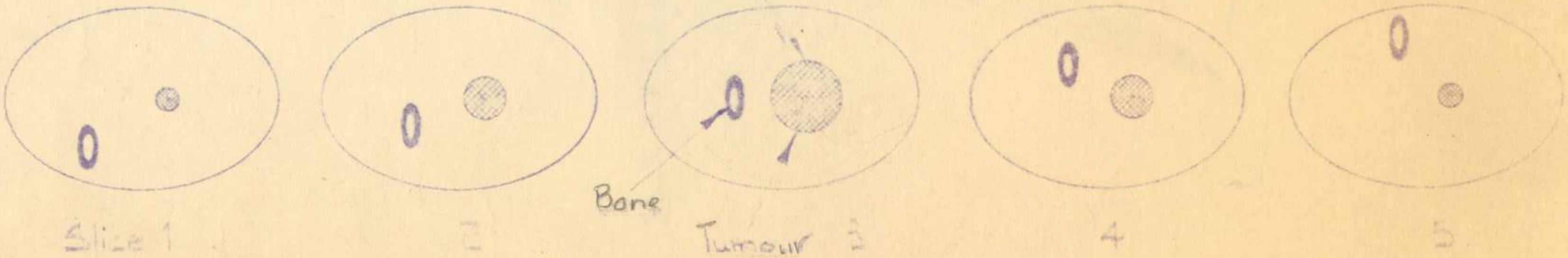

Pictures reconstructed by ~~proposed~~ system.

ICTURE

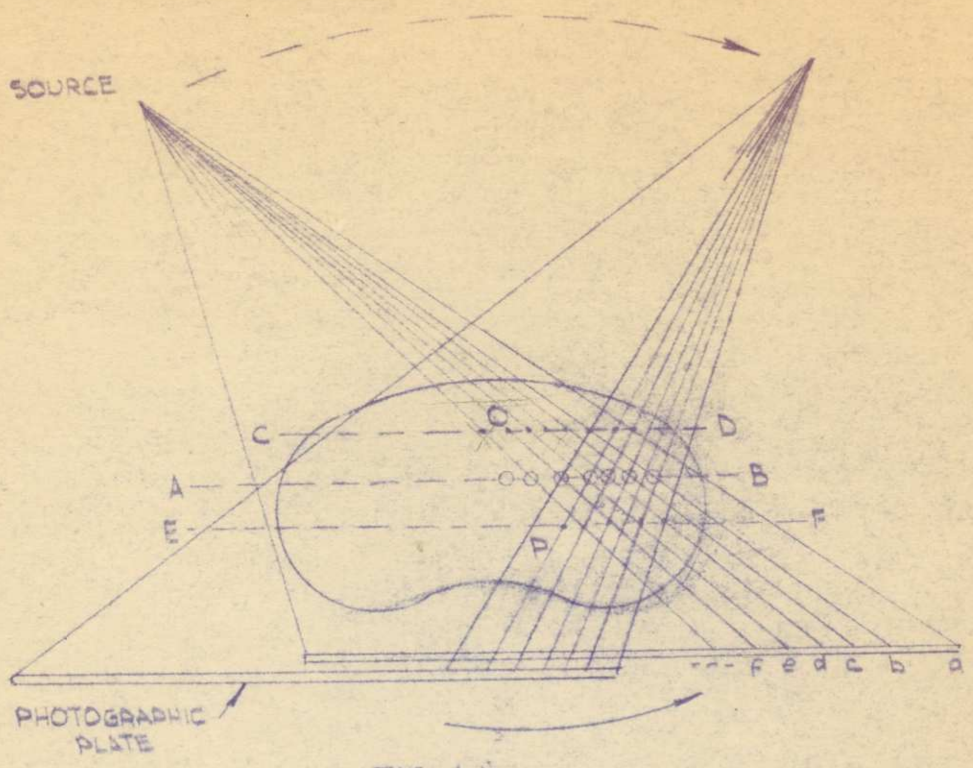
SOURCE
C
O
D
A
B
E
F
P
f e d c b a
PHOTOGRAPHIC PLATE

FIG.4A

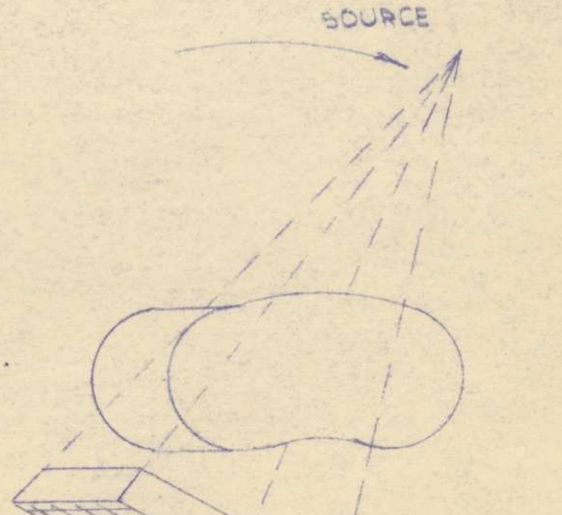
SOURCE
BANK OF DETECTORS

FIG.4B

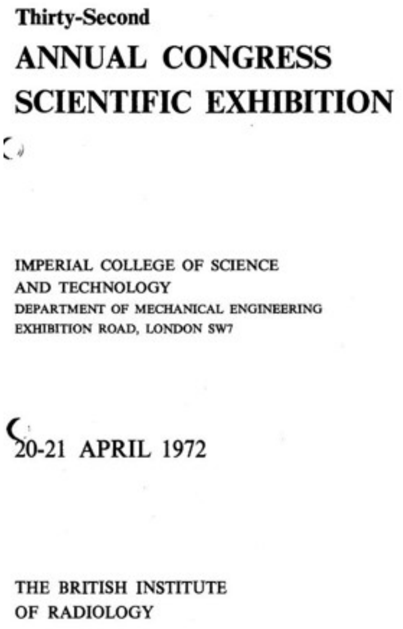

Thirty-Second

ANNUAL CONGRESS

SCIENTIFIC EXHIBITION

IMPERIAL COLLEGE OF SCIENCE
AND TECHNOLOGY
DEPARTMENT OF MECHANICAL ENGINEERING
EXHIBITION ROAD, LONDON SW7

20-21 APRIL 1972

THE BRITISH INSTITUTE
OF RADIOLOGY

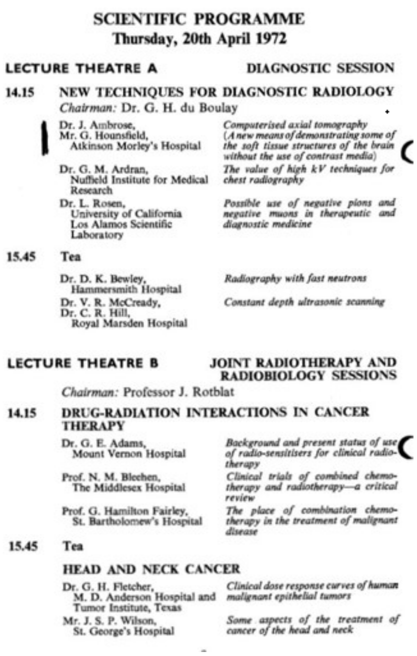

SCIENTIFIC PROGRAMME
Thursday, 20th April 1972

LECTURE THEATRE A — DIAGNOSTIC SESSION

14.15 NEW TECHNIQUES FOR DIAGNOSTIC RADIOLOGY
Chairman: Dr. G. H. du Boulay

Dr. J. Ambrose, Mr. G. Hounsfield, Atkinson Morley's Hospital	*Computerised axial tomography (A new means of demonstrating some of the soft tissue structures of the brain without the use of contrast media)*
Dr. G. M. Ardran, Nuffield Institute for Medical Research	*The value of high kV techniques for chest radiography*
Dr. L. Rosen, University of California Los Alamos Scientific Laboratory	*Possible use of negative pions and negative muons in therapeutic and diagnostic medicine*

15.45 Tea

Dr. D. K. Bewley, Hammersmith Hospital	*Radiography with fast neutrons*
Dr. V. R. McCready, Dr. C. R. Hill, Royal Marsden Hospital	*Constant depth ultrasonic scanning*

LECTURE THEATRE B — JOINT RADIOTHERAPY AND RADIOBIOLOGY SESSIONS

Chairman: Professor J. Rotblat

14.15 DRUG-RADIATION INTERACTIONS IN CANCER THERAPY

Dr. G. E. Adams, Mount Vernon Hospital	*Background and present status of use of radio-sensitisers for clinical radio-therapy*
Prof. N. M. Bleehen, The Middlesex Hospital	*Clinical trials of combined chemo-therapy and radiotherapy—a critical review*
Prof. G. Hamilton Fairley, St. Bartholomew's Hospital	*The place of combination chemo-therapy in the treatment of malignant disease*

15.45 Tea

HEAD AND NECK CANCER

Dr. G. H. Fletcher, M. D. Anderson Hospital and Tumor Institute, Texas	*Clinical dose response curves of human malignant epithelial tumors*
Mr. J. S. P. Wilson, St. George's Hospital	*Some aspects of the treatment of cancer of the head and neck*

Scientific Program
– 32nd *British Institute of Radiology Meeting* –
Imperial College London: Thursday, April 20, 1972

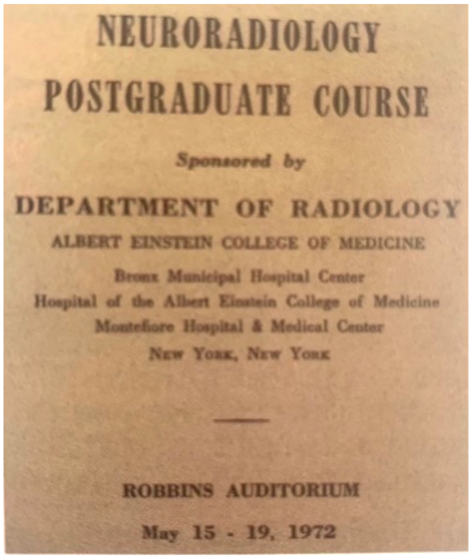

NEURORADIOLOGY
POSTGRADUATE COURSE

Sponsored by

DEPARTMENT OF RADIOLOGY
ALBERT EINSTEIN COLLEGE OF MEDICINE
Bronx Municipal Hospital Center
Hospital of the Albert Einstein College of Medicine
Montefiore Hospital & Medical Center
New York, New York

ROBBINS AUDITORIUM
May 15 - 19, 1972

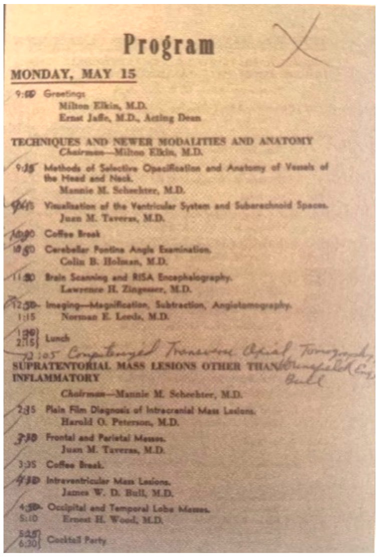

Program

MONDAY, MAY 15

9:00 Greetings
Milton Elkin, M.D.
Ernst Jaffe, M.D., Acting Dean

TECHNIQUES AND NEWER MODALITIES AND ANATOMY
Chairman—Milton Elkin, M.D.

9:15 Methods of Selective Opacification and Anatomy of Vessels of the Head and Neck.
Mannie M. Schechter, M.D.

9:45 Visualization of the Ventricular System and Subarachnoid Spaces.
Juan M. Taveras, M.D.

10:30 Coffee Break

10:50 Cerebellar Pontine Angle Examination.
Colin B. Holman, M.D.

11:30 Brain Scanning and RISA Encephalography.
Lawrence H. Zingesser, M.D.

12:30- Imaging—Magnification, Subtraction, Angiotomography.
1:15 Norman E. Leeds, M.D.

1:30
2:15 Lunch

2:05 Computerized Transverse Axial Tomography Hounsfield (Eng) Bull

SUPRATENTORIAL MASS LESIONS OTHER THAN INFLAMMATORY
Chairman—Mannie M. Schechter, M.D.

2:35 Plain Film Diagnosis of Intracranial Mass Lesions.
Harold O. Peterson, M.D.

3:10 Frontal and Parietal Masses.
Juan M. Taveras, M.D.

3:35 Coffee Break.

4:10 Intraventricular Mass Lesions.
James W. D. Bull, M.D.

4:50- Occipital and Temporal Lobe Masses.
5:10 Ernest H. Wood, M.D.

5:25
6:30 Cocktail Party

Program of the first day of the Neuroradiology Postgraduate Course
Montefiore Medical Center, New York, NY: May 15, 1972

114 Copies to: Mr. W. Ingham. ✓
Mr. E. Gowler.
Mr. R. Froggatt.

file 3D x-Ray

VISIT TO NEW YORK AND BOSTON

I get the impression that many important English Doctors have moved to America and most have some connections with Dr. Bull and DHSS and the Atkinson Morley Hospital. This is greatly in our favour. Dr. Bull is known very well over there, many doctors being ex-pupils of his.

Dr. Wood of Columbia Institute suggested that there would be an evaluation period of at least a year, as in this country, and said that in the first year we would sell a minimum of three and a maximum of six in New York and about the same over the whole country. But in the five years he predicted 500 machines could be sold, i.e. every town with a population over half a million would have one (this may be an 'on the spot' exaggeration - considering the number of towns of this size, 100-200 machines is a more likely figure.)

The Chicago Exhibition is very important and he suggests we book space immediately, as it gets taken up very quickly. He says that a machine should be sent over for the exhibition, as people in America like to see what they are buying. He suggested that he bought it after the exhibition for evaluation and to help us with sales.

He stressed the point that the after-sales attention is all important. The resident "picker" engineer is continually modifying the machines installed to the requirements of the Doctors and each machine is individually made for each hospital. This cross checks with New York University Medical Centre, where it was stated that the maintenance bill worked out to be 10% annually of the total cost of equipment and apparently is good business for the companies concerned.

I saw a newly installed X-ray room, which cost a quarter million dollars and took six months to install the equip. The total equipment is no more complex than the EMISCANNER and the engineering is of an average standard - there have been two resident Picker engineers continually making on the spot modifications and attending to every whim of the doctors in charge.

There are three resident Picker engineers to attend to the 160 X-ray machines in the hospital.

G. N. HOUNSFIELD

RECEIVED
25 MAY 1972
MR. [illegible] OFFICE

24th May, 1972

about 30 [illegible]

Visited (read paper)

** Albert Einstein Clinic,
185 Eastchester Road,
Bronx NY,
430 2000

Dr. Lawrence H. Zingesser, AL Ei
Col. of Med.
Radiology Dept. NY Bronx 10461

Require Literature

Dr. M.M. Schechter

Albert Einstein Col. of Medicine Bronx NY
4302575 (director of the symposium)

Visited

** Columbia Neurological Institute
of New York,
710 West 168 Street, Broadway,
N.Y. NY 10032
212 579 3051

Contact

Dr. Earnest H. Wood (201) 652-6876

Friend of Dr. Bull. Very helpful - suggests tie up as in "Atkinson Morley" - hopes to buy machine and help to sell for us - may require resident engineer. (Note - there are three resident Picker Engineers for 160 X-ray machines. It is a very large hospital, more than 24 neurological tests a day. A figure of 6,000 neurological in-patients was given.

** **Visited**

N.Y. Hospital

Cornell U.V. Medical Centre,
1300 York Street,
Avenue of Americas

Dr. John Evans - Chairman of Radiological Dept.

Very interested, hopes to buy machine.

Contact

Dr. Gordon Potts, engaged in research
(Ex Atkinson Morley - knows Dr. Ambrose and Dr. Bull)

Dept. of radiology, N.Y. Hospital
525E 68th Street, N.Y.10021

Very helpful - very large hospital

? Memorial Hospital
over Road

Dr. Robins requires literature - very interested

(Unable to visit)

** Mayo Graduate School of Medicine
Mayo Foundation & University of Minnesota,
Rochester, Minnesota 55901
Area Code (507) 282 2511

Dr. Colin B. Holman
Consultant in Radiology
Mayo Clinic - Large Hospital

(friend of Dr. Bull)

Requires film (could we post)

Visited

** N.Y. University Medical Centre
E 31st St.

Chief Physicist Dr. Colin Orton
(was out)

Dr. Chase(very impressed but hypercritical) I have reason to believe that he has close connections with Phillips, enthusiastically talked of buying, he has a team of English doctors and physicists working for him - one - Dr. C.H. Marshall, whom I know personally.

2,000 angiograms done a year
5,000 brain scans

Bellvue Hospital
Neuro Radiology Dept.
550 1st Avenue

One of the largest hospitals situated next to N.Y. Medical Centre (above), works with it, has 2,000 neuro-patients/year. (suggest send literature to Chief Neuro Radiologist)

Visited

Bronx Vetrans Administration Hospital
Bronx N.Y. 10468

Dr. F.K. Chan - connected with Columbia Hosp. (i.e. Dr. Wood). At moment have only 15 brain patients/week. They are expanding their premises and are well off financially (and have a large grant for new equipment). May consider machine after trials at Columbia (Although they are expanding, their need appears to be not high)

Physicst MR. CYPRIAN REID

Visited

Massacheusetts General Hospital
Boston Massachusetts 02114
Area Code 617 726 8344

Dr. Ivan Taveras
Radiologist in Chief

Very helpful - knows Dr. Bull

Good selling prospects after assessment in N.Y.

REQUIRING LITERATURE

Yun Peng Huang, M.D.,
Dept. of Radiology,
Mount Sinai Hospital,
New York City, N.Y.10029

Bernard S. Epstein, M.D.,
The Long Island Jewish-Hillside Medical Centre
New Hyde Park,
N.Y. 10042.

PAL VARGA, M.D.,
V.A. Hospital Manhattan,
24th Street and First Avenue,
New York, N.Y. 10010

Dr. Edward Pollock,
Dept. Radiology,
Presbyterian Hospital,
622 W. 168th Street,
New York, N.Y.10032

M.L. Rezmik,
P.O. Box 1616,
Durban,
Natal, South Africa

P.N. Goodwin,
Radiology Dept.,
Albert Einstein College,
Bronx, N.Y. 10641.

E.S. Himelstein,
Radiology - Jacobi Hospital,
Bronx Municipal Hospital Centre,
Bronx, N.Y.10641

John Olsen, M.D.,
University of Chicago Hospital,
950E. 59th Street,
Chicago.

Samuel M. Wolpert, M.D.,
Tufts-New England Medical Centre,
171, Harrison Avenue,
Boston, Mass. 02111

Dr. Heywood Epstein,
Dept. of Radiology,
Nassau Hospital,
Mineola, L.I.,
New York, U.S.A.

Dr. Robert Epstein,
Dept. of Radiology,
Mount Sanai Hospital,
Miami Beach, Florida, U.S.A.

Dr. Forrest Clore,
4016 S.W. 38th Street,
Gainesville, Florida 32601

Barry T. Katzen, M.D.,
435 E. 70th Street,
New York, N.Y.10021

Rushton Capers,
ET Du Pont
De Nemours,
380 All Wood Road,
Allwood Street,
Clifton, N. Jersey 07012

Dr. Octavio Cortef,
St. Barnabus Hospital of Cronic. Desease,
Arthur Avenue, Bronx, N.Y.

Warren W. Church,
U.S. Public Health Service,
109, Holton Street,
Winchester, Mass.

Roberto B. Jimenez, M.D.,
Mass. General Hospital,
Boston, Mass. 02114

Melvin L. Haas, M.D.,
1467 Harper Street,
Augusta, Georgia 30904, U.S.A.

E.W. Emery,
Dept. of Medical Physics,
Royal Post Graduent Medicine School,
Hammersmith Hospital, London, W 12 OHS

Dr. Edward Webster,
Mass General Hospital,
Boston, Mass.

J.B. Stull, M.D.
Hale Hospital,
4A Mill Street,
Haverhill, Mass 01830

27

Mini-Computers: The critical component for success

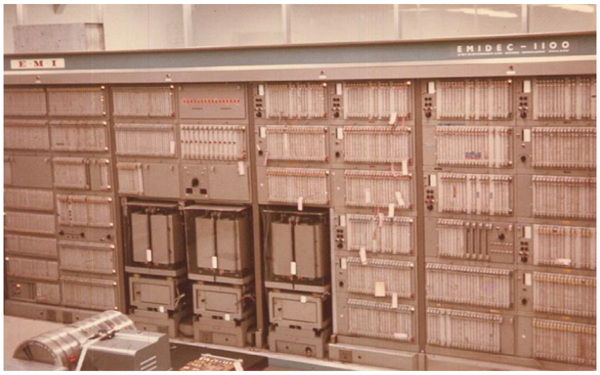

EMIDEC 1100

Hounsfield worked on the design of the EMIDEC 1100 mainframe computer and knew its capabilities. It was fast for its time, but that was all relative. It had 1024 words of memory and three magnetic drums that stored less than a 3.5" floppy of the 90s. It could add 2 numbers in 140 msec.

In the prototype EMI Mark the projection data was collected by a reel-to-reel tape drive, sent across town for overnight reconstruction on an ICL1905 mainframe, and returned the next day for display, analysis and Polaroid image capture and medical record archiving.

On March 22, 1965, the PDP-8, a 12-bit (two 6-bit words) minicomputer produced by Digital Equipment Corporation (DEC) was launched. It was the first commercially successful minicomputer, with over 50,000 units being sold over the model's lifetime. [https://en.wikipedia.org/wiki/PDP-8] At $18,500, it was the first computer to be sold for under $20,000 with a smaller subsequent PDP-8/S being the first computer to be sold for under $10,000!. In 1967, the new ASCII character convention dictated 8-bit words (& multiples thereof), to accommodate all 128 characters. That changed everything. The Nova family was a series of 16-bit minicomputers released by Data General (DG) in 1969 by the designer of the PDP-8 and other DEC colleagues. It differed from the PDP-8, by adopting the new standard with 32K 16-bit words. Advertised as "the best small computer in the world", https://en.wikipedia.org/wiki/Data_General_Nova#History NOVA quickly gained a following, especially in scientific and educational markets. It outperformed and cost less than half the equivalent from DEC at a base price of US$3,995. In a simulated test at EMI CRL labs, a Nova 820, recently acquired by the Cognitive Systems Group, beat out the PDP-8 and became the device of choice to launch into the world's hospital market.

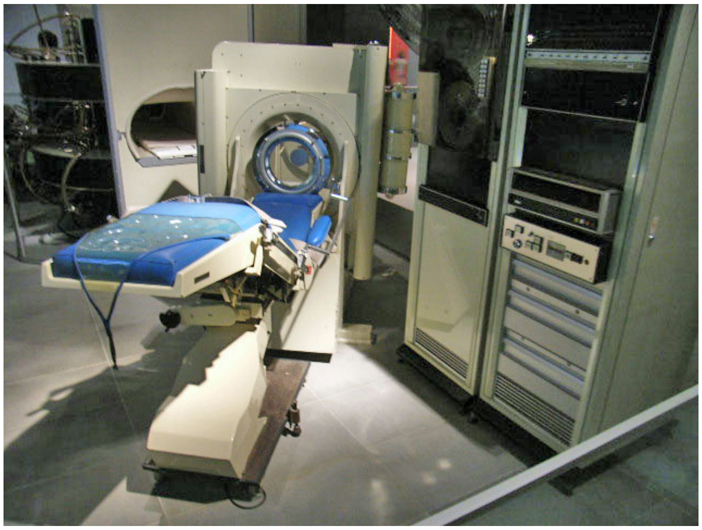

Data General Nova next to EMI Mark I. Left rack contains tape drive. Right rack has the computer and other hardware

Prior to the availability of the minicomputer the market was estimated to be 20 units because of the challenge of reconstructing the data at an offline mainframe. The minicomputer was the final key *critical component* in a self-contained scanner system that could reside in a hospital radiology department (see below on the world's first mini-computers.).

Material on the early CT research at MGH starting in 1973, courtesy of David Laning, Ph.D.

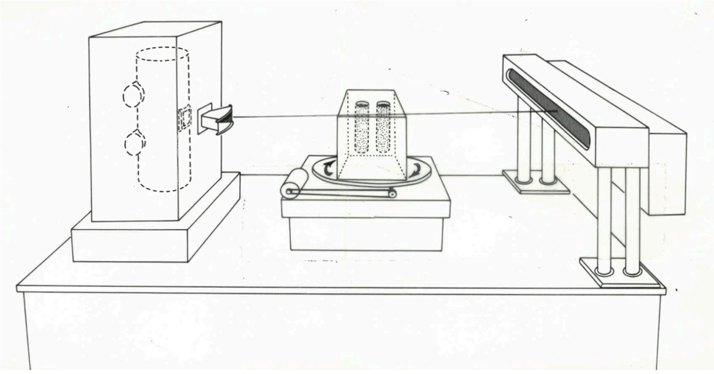

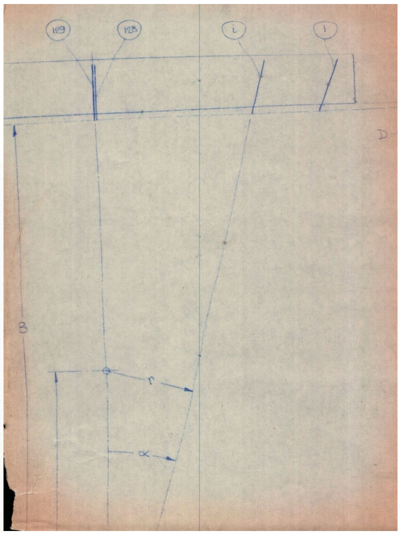

Drawing of table-top system

Measurement geometry with xenon detector on top showing detector plates and center of rotation projection 1/4 of the way from 129 and 128

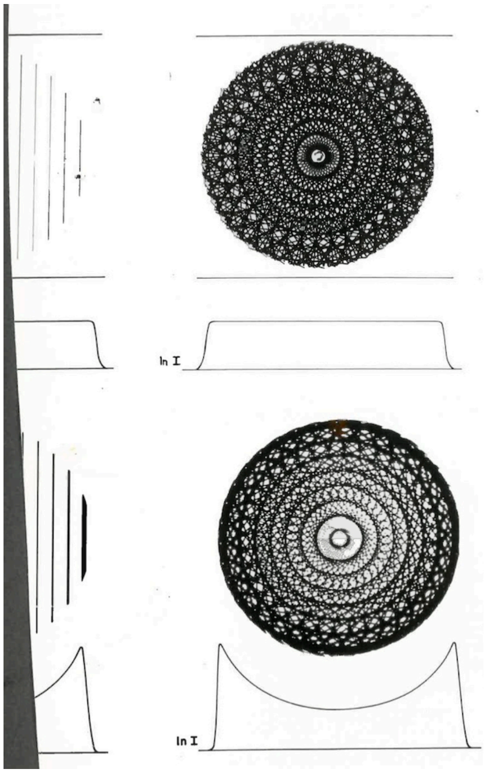

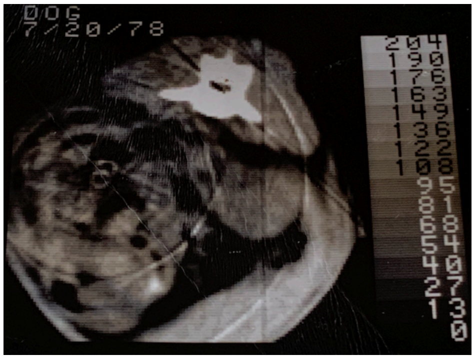

Upper left: measured log intensity through a round object with a beam shaping filter (bowtie) that makes the transmission uniform. Lower left: without the filter. Rosette patterns are from backprojection of measured intensity showing fewer photons contributing to the center of the image if the filter is not used. Above: image of a dog.

New EMI machine for diagnosing brain disease

Photographs available

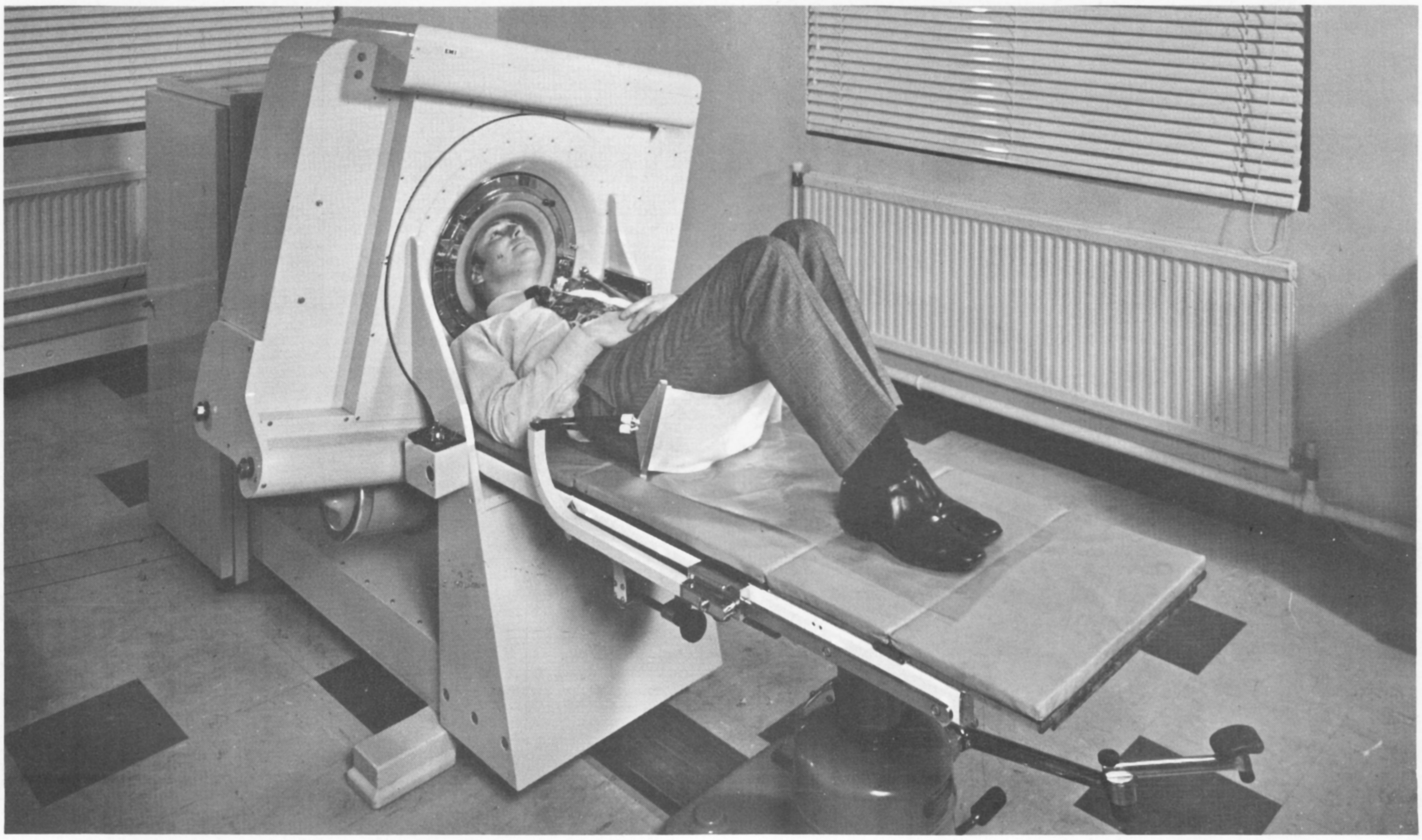

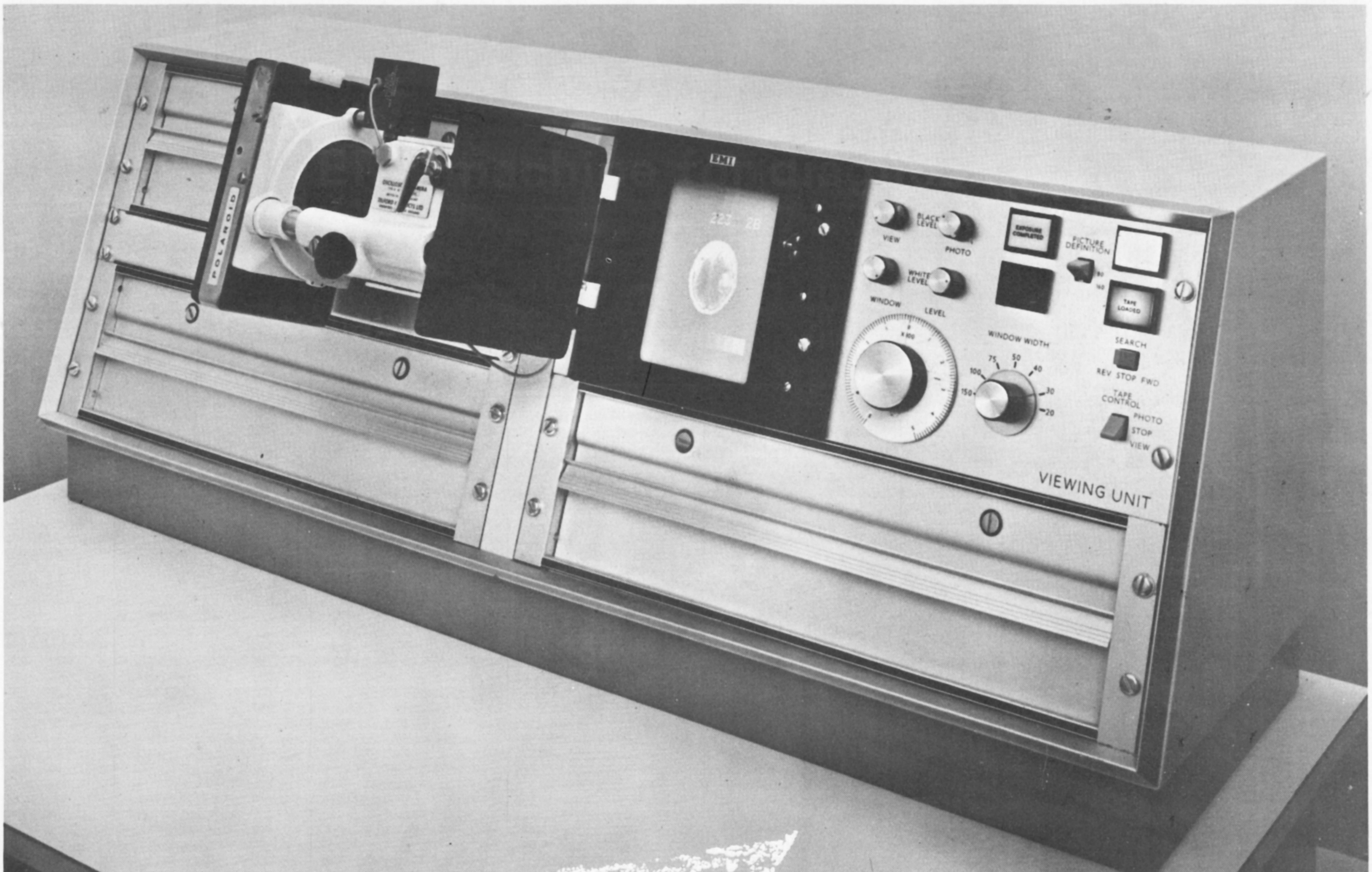

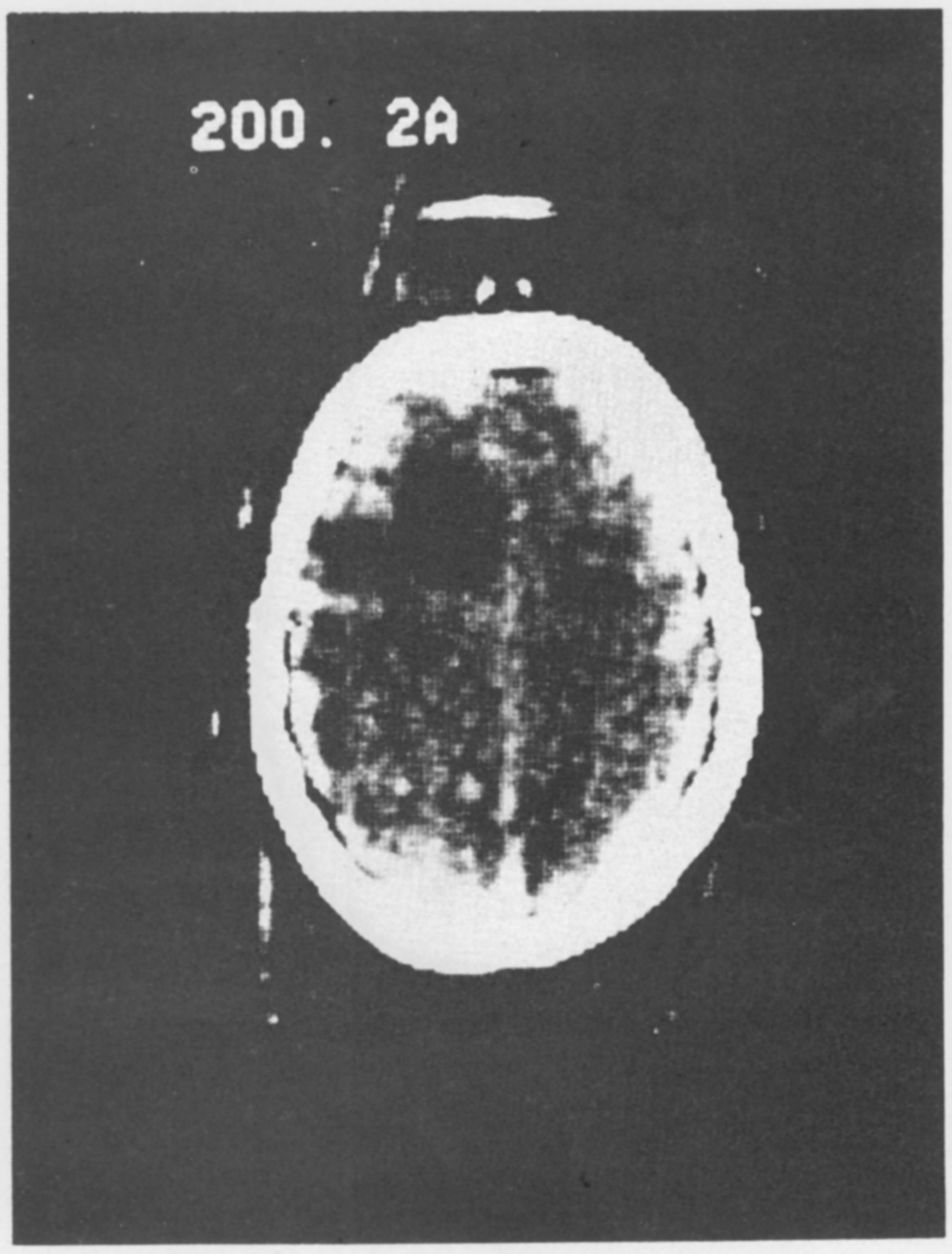

Astrocytoma III cystic left front lobe (160 x 160 matrix high-definition picture - note detection of grey and white matter patterns)

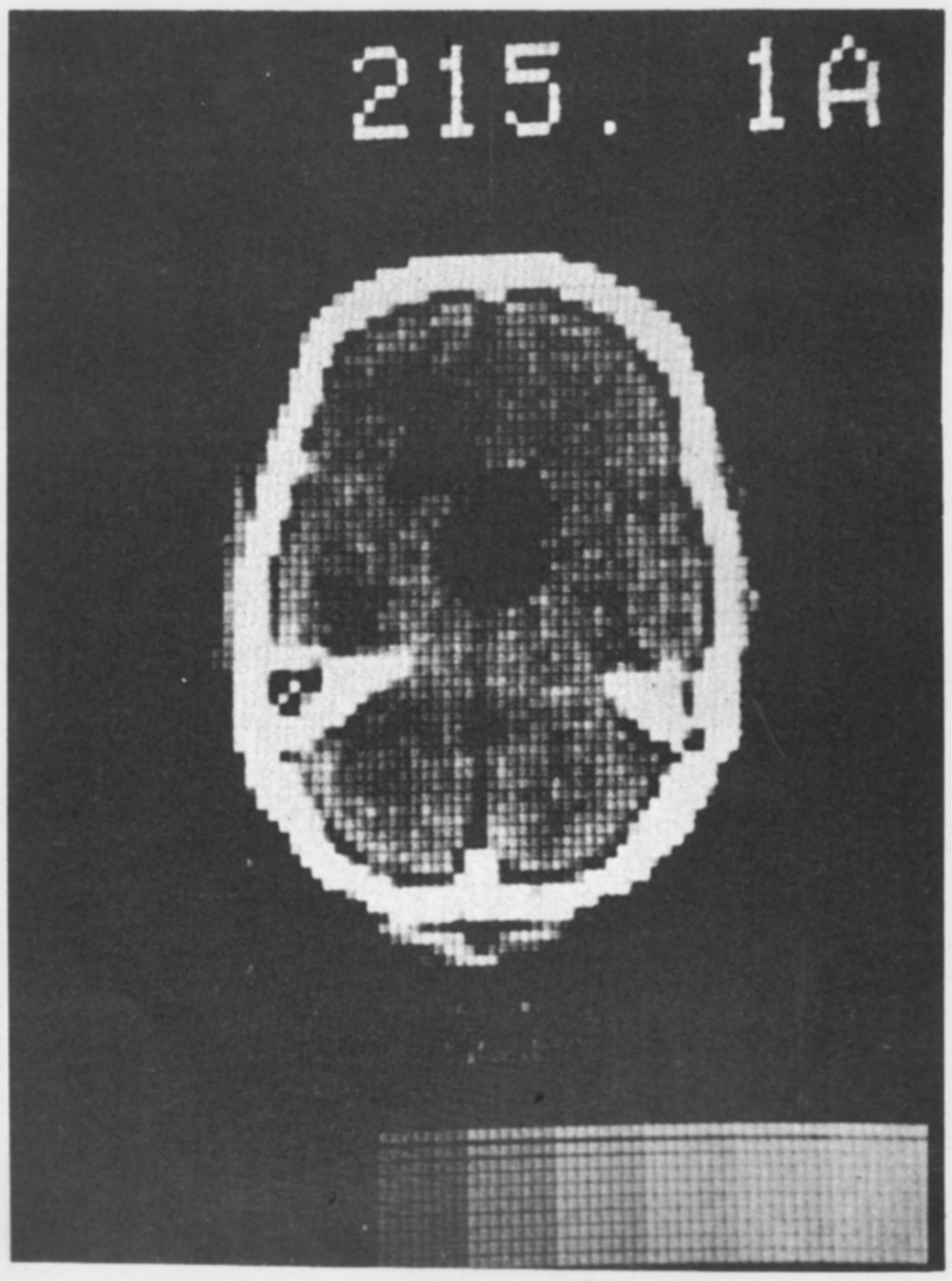

Cranio pharyngioma

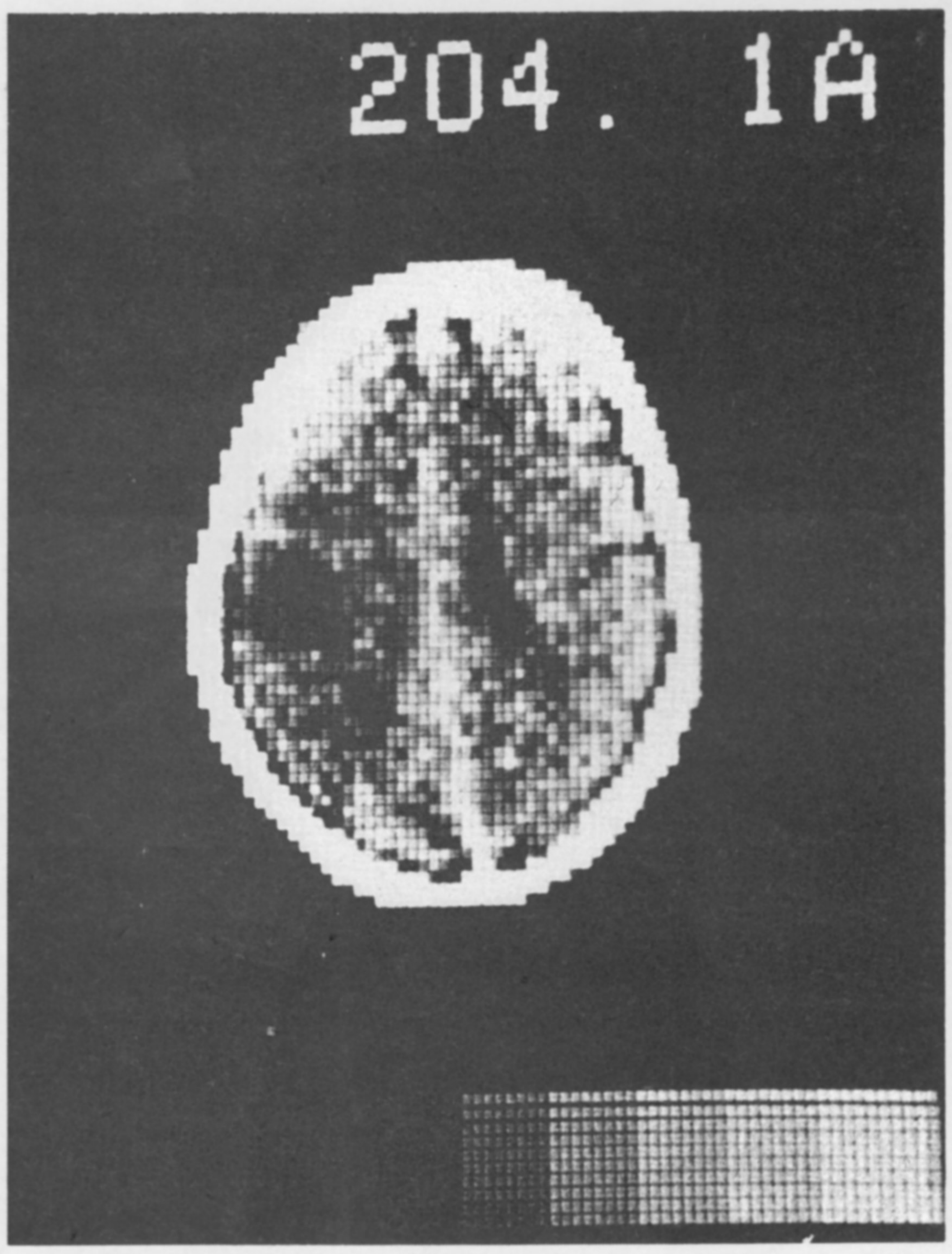

L. parietal astrocytoma grade III.

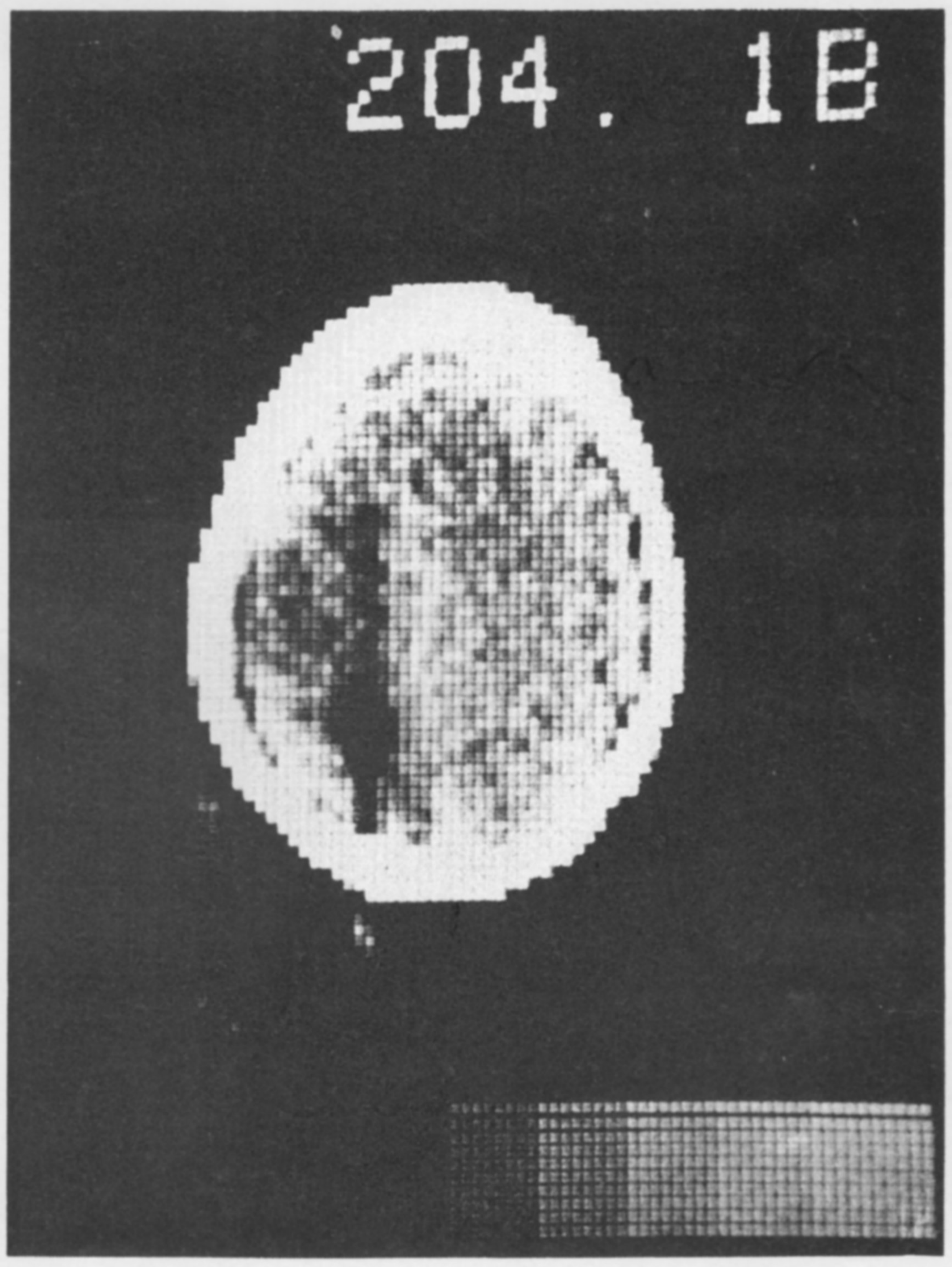

(As left) Taken 1 cm. higher on same patient.

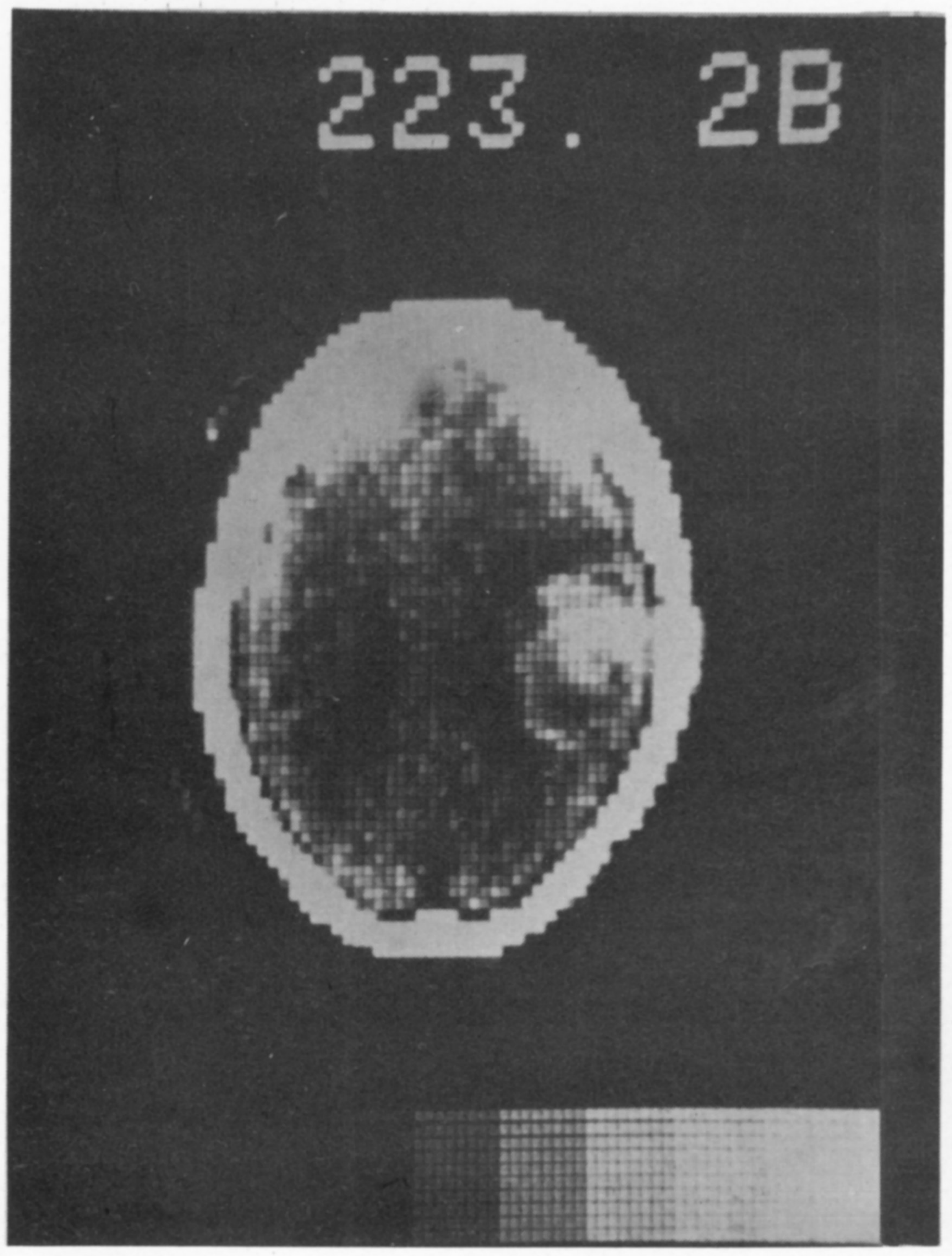

Haematoma associated with tumour

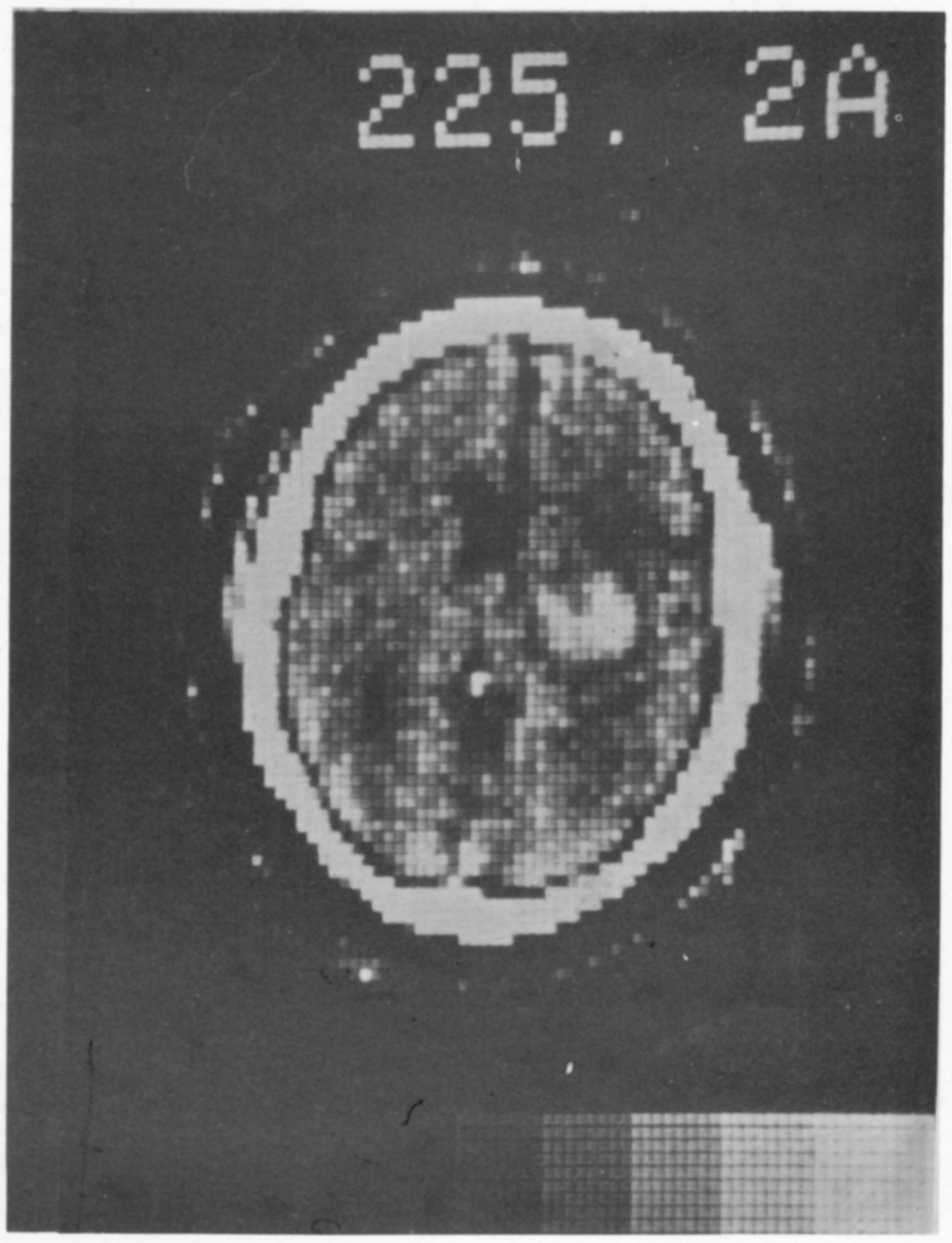

Haematoma

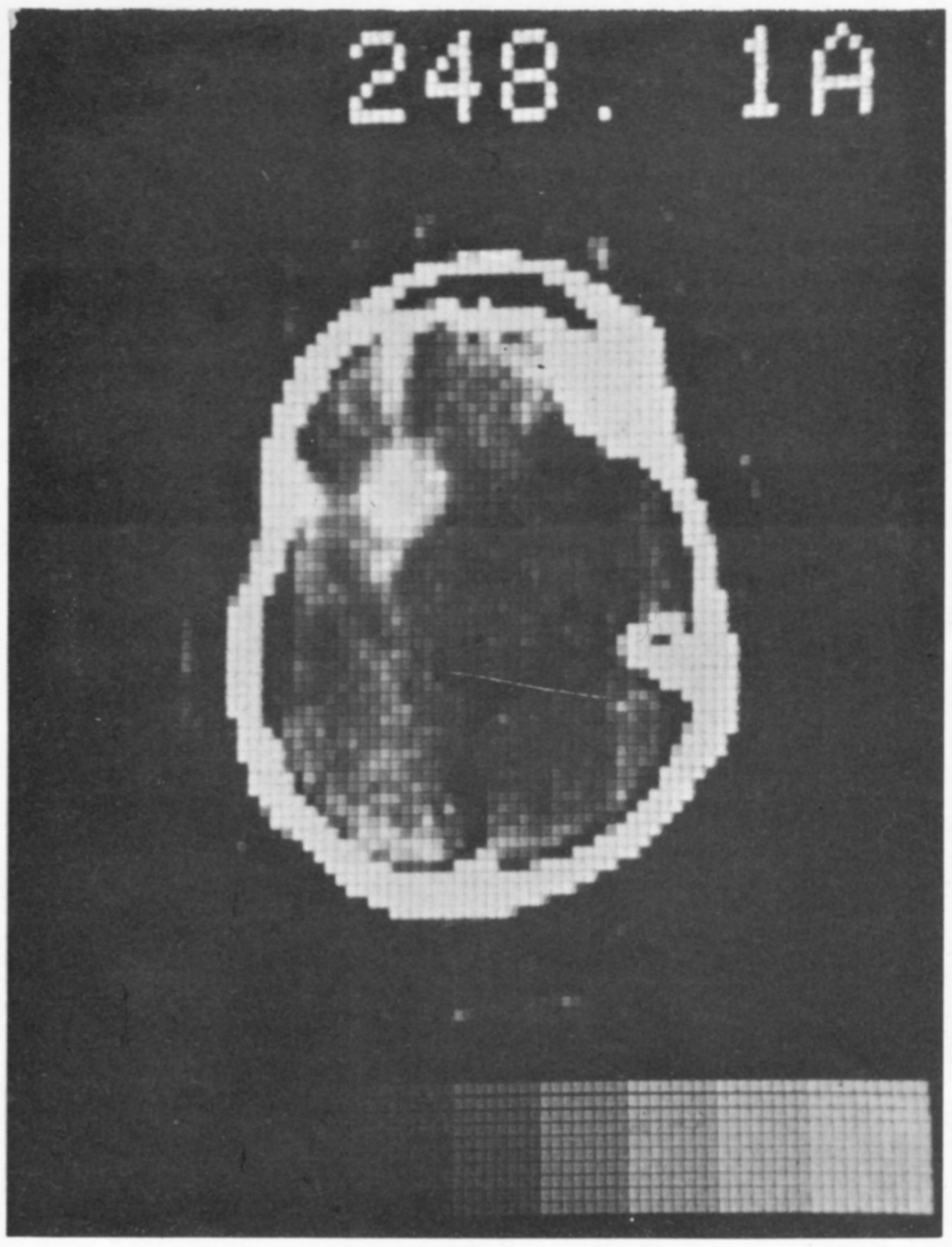

Primary intracerebral haemorrhage

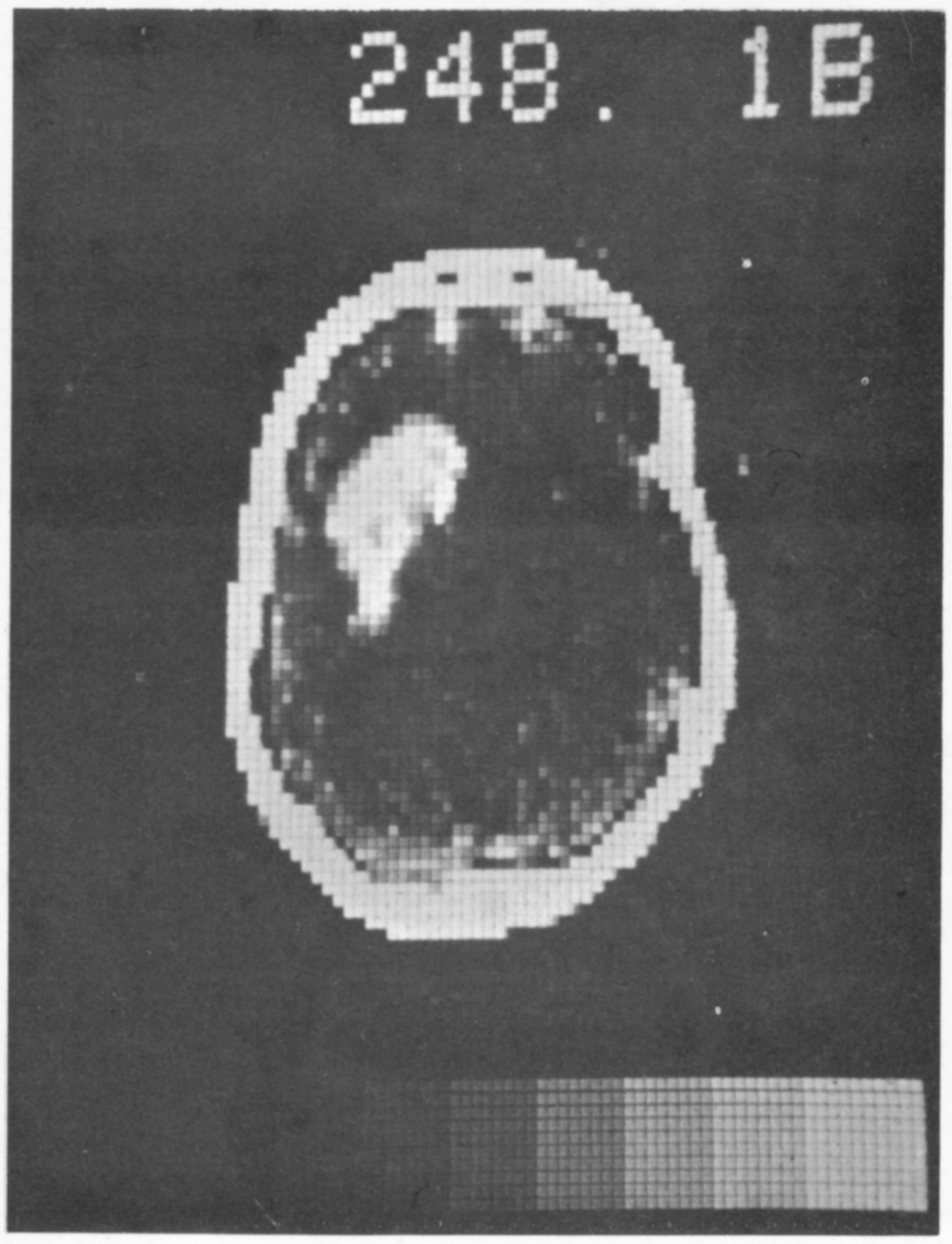

(As left) Taken 1 cm. higher on same patient.

Results by alternative techniques

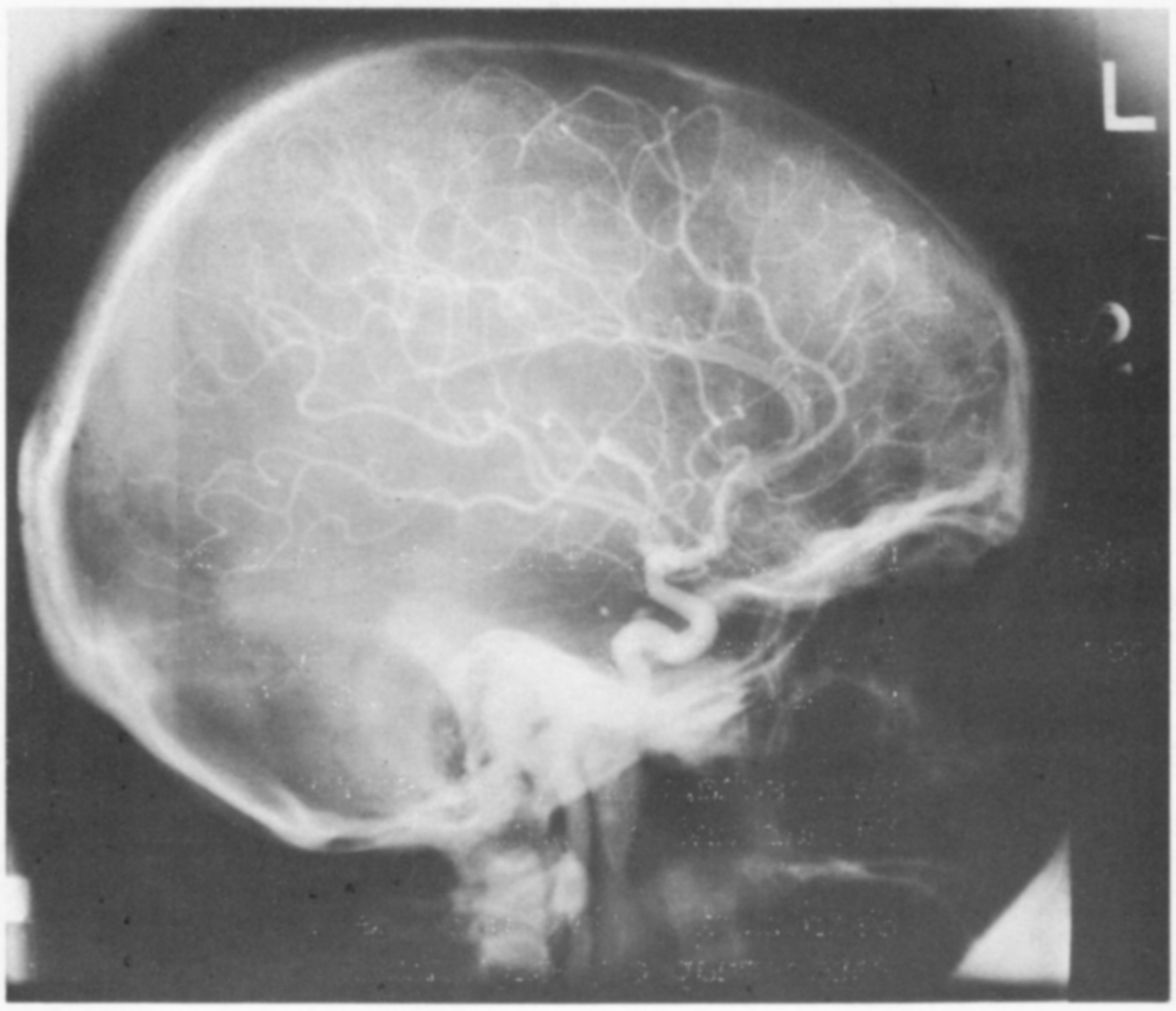

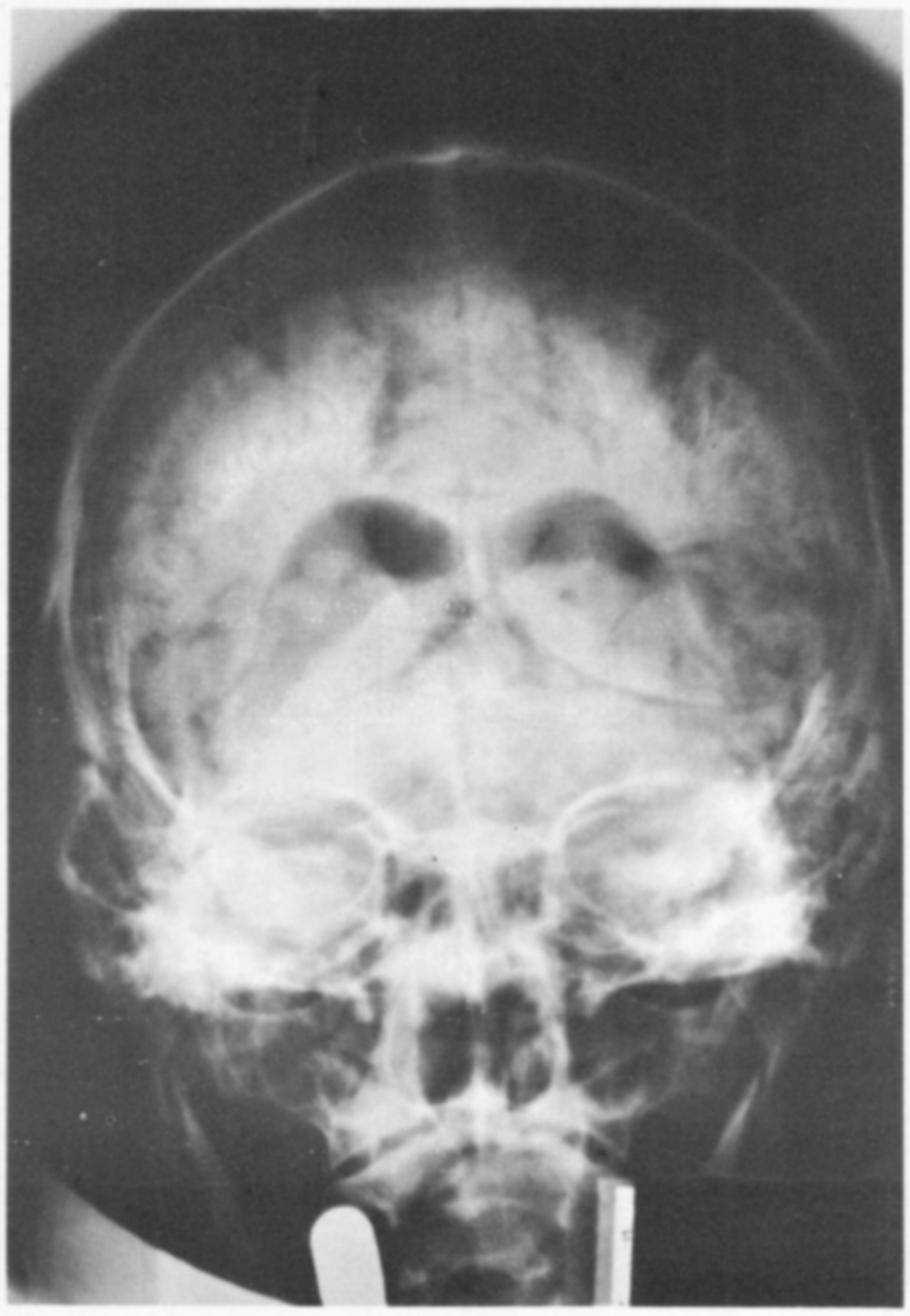

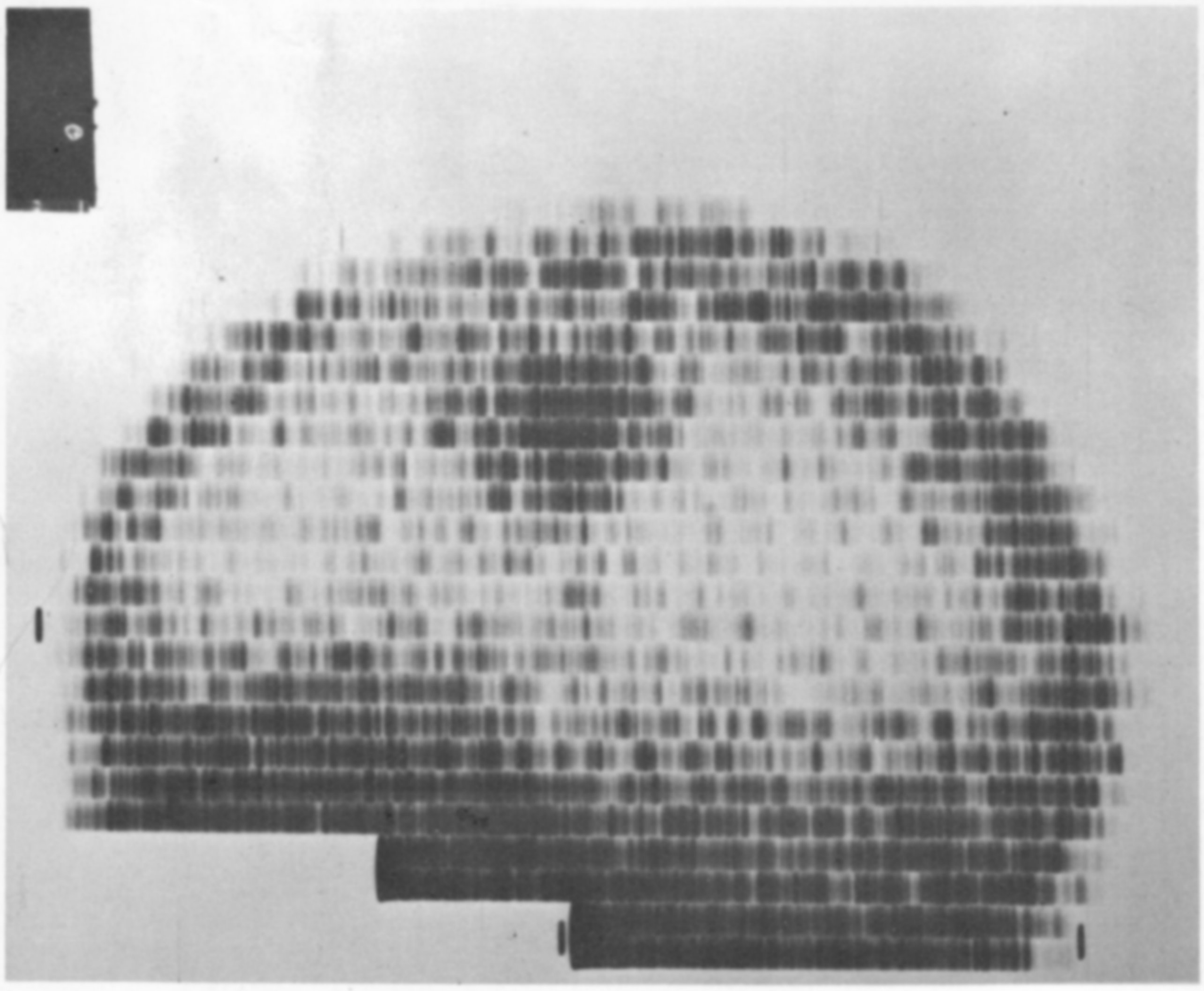

EMI-SCANNER

A new perspective on brain disease

EMI

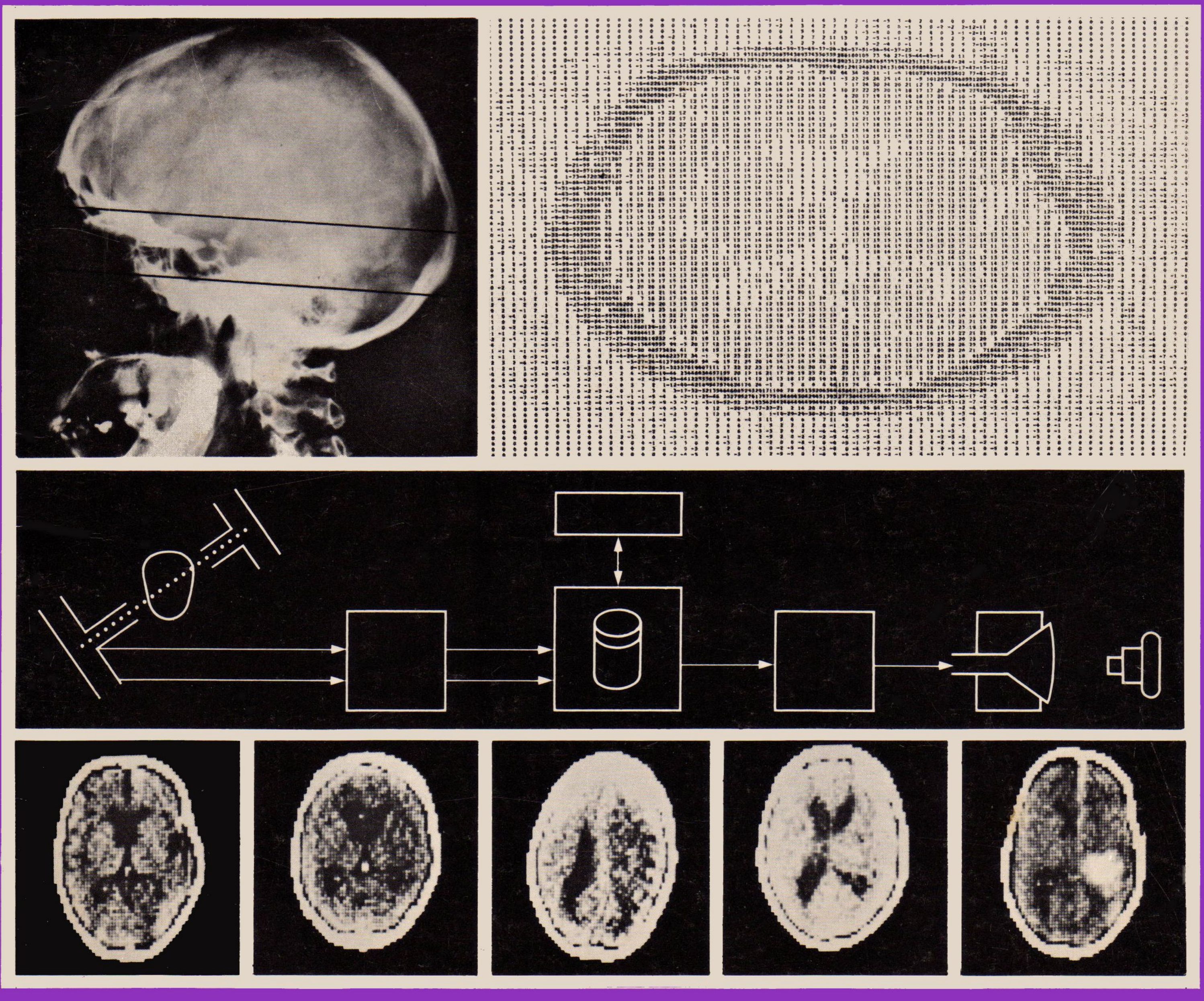

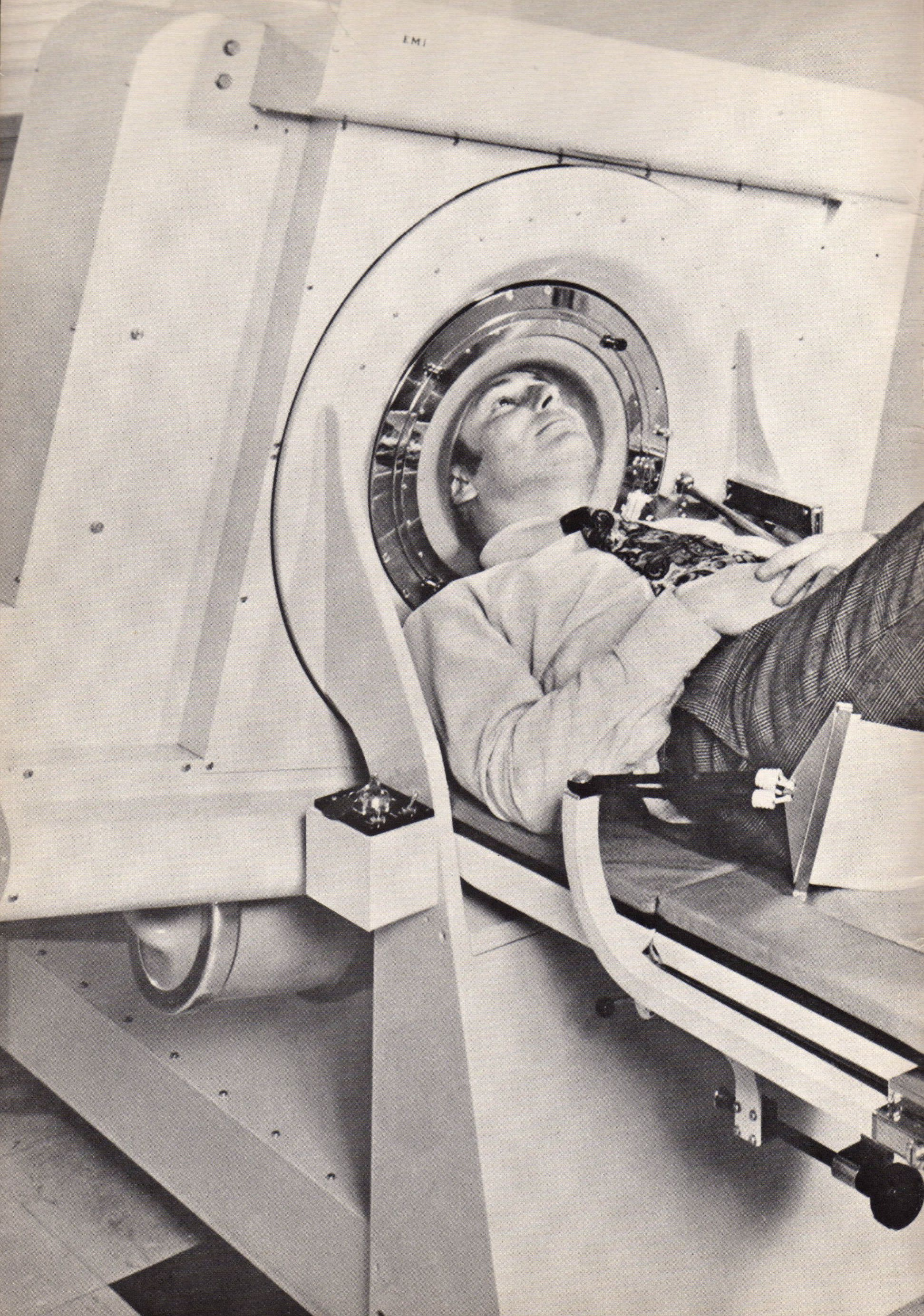
EMI

EMI-SCANNER

A new perspective on brain disease

Left
The EMI-SCANNER installed at the Department of Neuroradiology at the Atkinson Morley's Hospital—which contains the Neurological and Neurosurgical Units of St. George's Hospital, Wimbledon, England.

EMI-SCANNER

The key features of the EMI-SCANNER system in the investigation of brain tissues are:-

1. 100 times more information is obtained than from conventional X-ray systems
2. Straightforward operation by one unaided radiographer
3. Examinations may be performed up to a rate of four patients per hour (single scan investigation giving results for two contiguous tomographic slices for each patient)
4. Discomfort to the patient during investigation is avoided
5. Residual morbidity is eliminated
6. Results of investigations are available within a few minutes of the scan
7. Measures X-ray absorption of brain tissue to ½% accuracy

A new perspective on brain disease

The photographs of brain tissue abnormalities shown below were produced using a new system of transverse axial tomography developed by EMI Limited in conjunction with Britain's Department of Health & Social Security.

It has been developed to permit considerably greater information to be extracted from the transmission of X-ray photons through the head, and to present the information obtained in the most useful form for evaluation by neuroradiologists, neurologists and neurosurgeons.

In clinical terms, the most important contribution resulting from this development is the presentation of detailed information on brain tissue abnormalities in a new perspective so that not only is accurate indication given of the nature of the lesion, but also its location within the head is defined with considerable precision in three dimensions.

The EMI-SCANNER system, which has undergone prolonged clinical evaluation, has demonstrated the facility for discriminating between tissues of minutely varying density and for eliminating many of the causes of patient discomfort and morbidity, normally associated with brain investigations using pneumography, angiography and radio-active isotope scanning.

It has also shown that, by avoiding the need for anaesthesia or the injection of radio-opaque liquids, or gases, significant economies are possible. Typically an investigation consisting of three scans can be carried out by a radiographer, unaided, in 30 minutes. These economies are achieved particularly by avoiding the need to involve medical specialists in the initial examination and eliminating the subsequent recovery period in hospital.

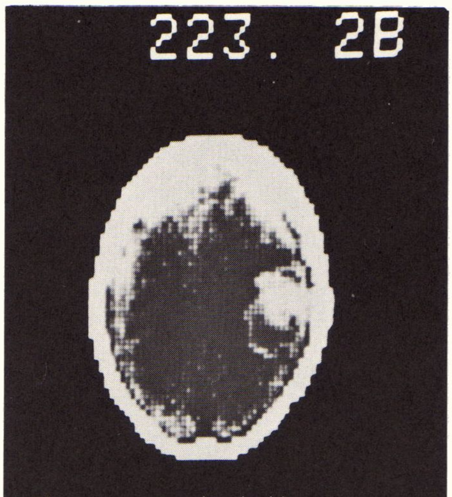

Haematoma associated with tumour.

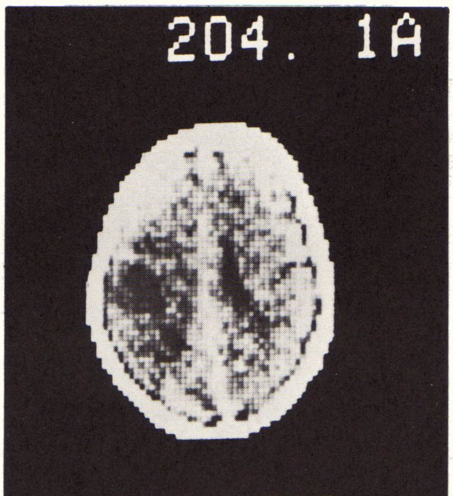

L. Parietal Astrocytoma Grade III.

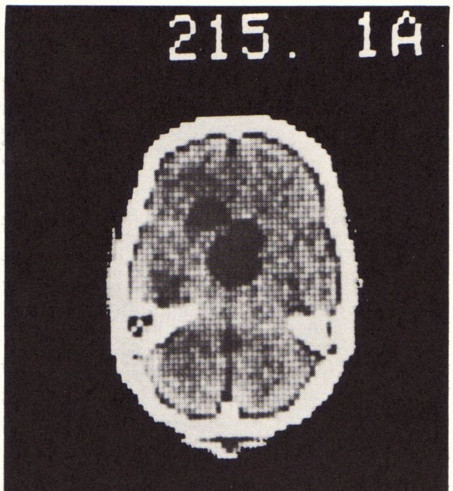

Cranio Pharyngioma. Slice taken 3.5 cm. above the orbito-meatal line. (Just above the pituitary fossa).

Primary Intracerebral Haemorrhage:

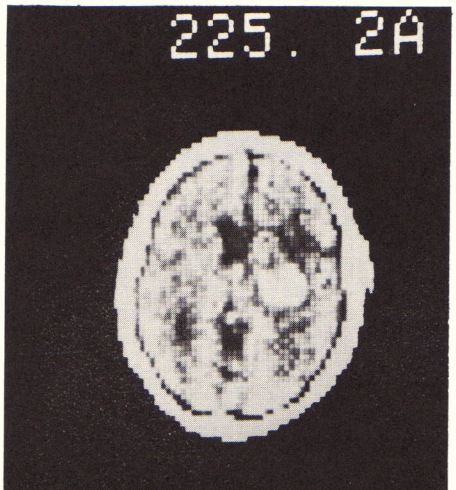

Slice taken between 3.7 and 5.0 cm. above the orbito-meatal line.

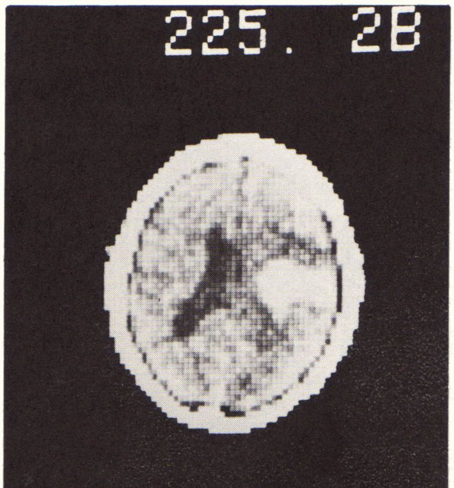

Slice taken between 5.0 and 6.3 cm. above the orbito-meatal line.

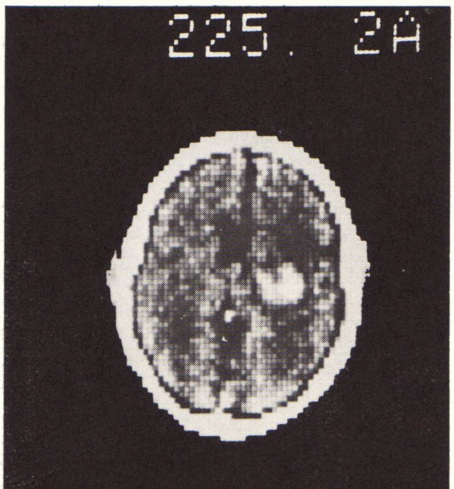

As picture at left (225.2A) but with different setting of "window level" control.

Comparative results

These illustrations compare the results by existing procedures with EMI-SCANNER investigation of a patient with a Primary Intracerebral Haemorrhage.

A Lateral Arteriogram
B Radioisotope Scan
C EMI-SCANNER record of a slice between 5.0cm and 6.3cm above the orbito-meatal line
D EMI-SCANNER Print Out (optional extra)

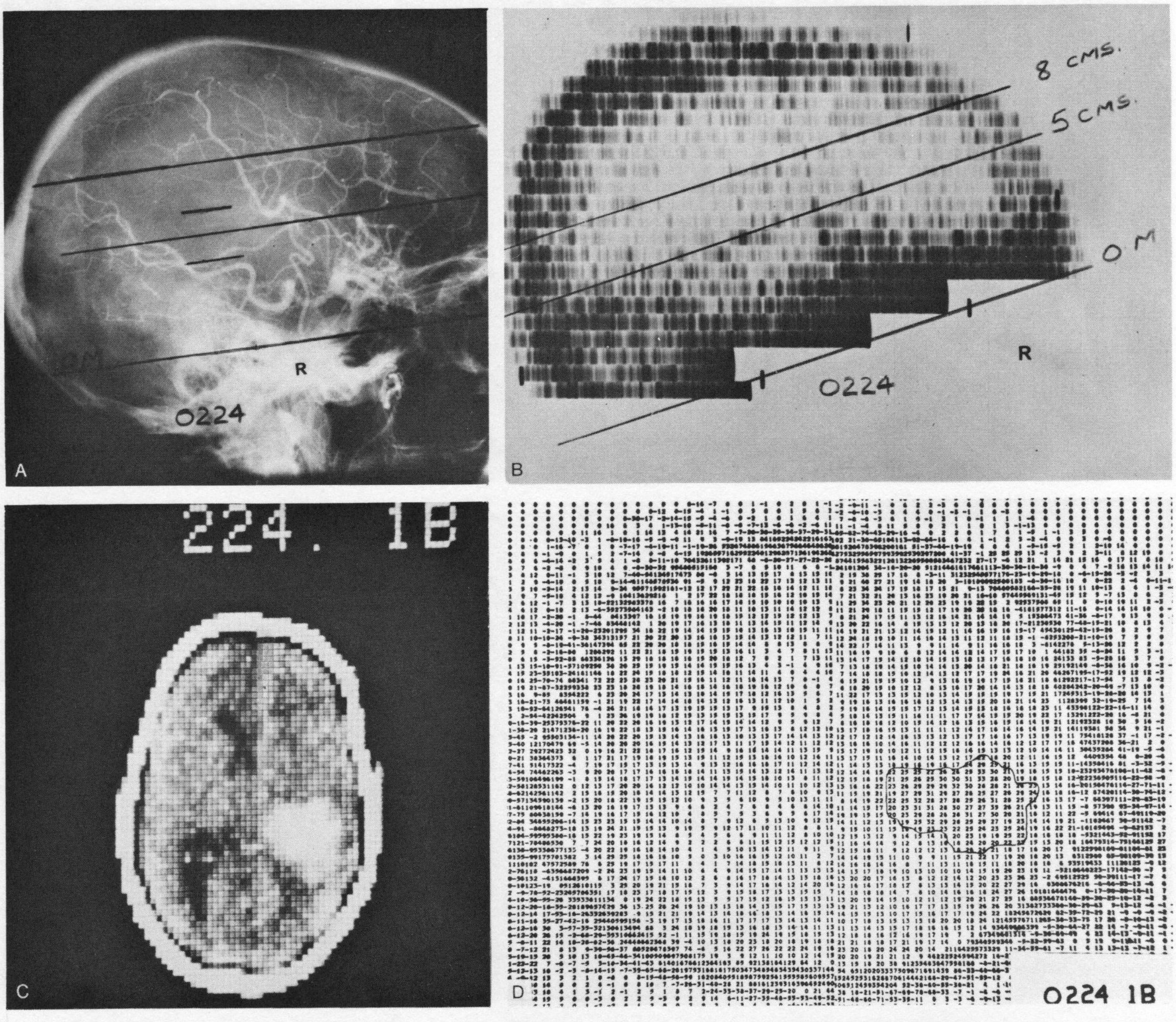

Theory of the EMI-SCANNER system

Investigations have indicated that with conventional X-ray techniques, approximately 99% of the information released by the transmission of X-ray photons through the head is not realised in a useful form on the photographic plate. This information loss is, of course, due to the difficulty of discriminating between tissues of closely similar photon absorption coefficients, together with confusion resulting from the super-imposition of three-dimensional information on a two-dimensional record.

Since all soft tissues fall within the narrow band of only approximately 4% overall variation in absorption coefficient (Figure 1), discriminating between different tissue densities within this band demands the use of extremely sensitive detectors.

Further, to avoid superimposition, and hence confusion of the details in the resulting record, it is necessary to employ tomographic principles, to allow the three-dimensional object to be examined as a series of two-dimensional slices (Figure 2).

The EMI-SCANNER combines the use of sensitive photon detectors with tomographic examination techniques. These are used in conjunction with a computer to handle the vastly increased amount of information which is retrieved. Although the X-ray dose is comparable with a conventional skull radiograph, the system uses the X-ray photons more efficiently to yield approximately 100 times more information than alternative methods and is consequently able to present information on marginally varying densities of tissue across the full area of each tomographic slice.

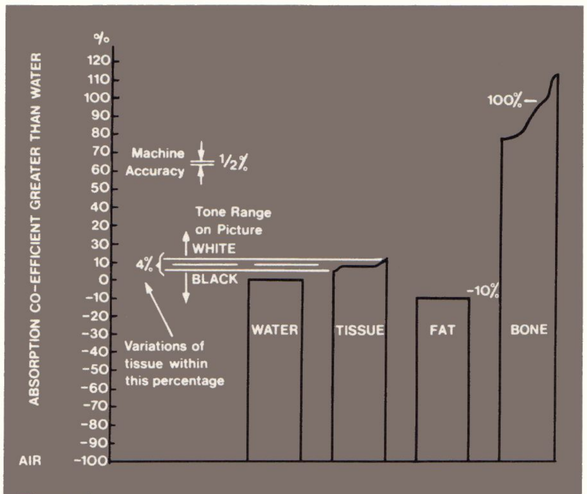

Figure 1

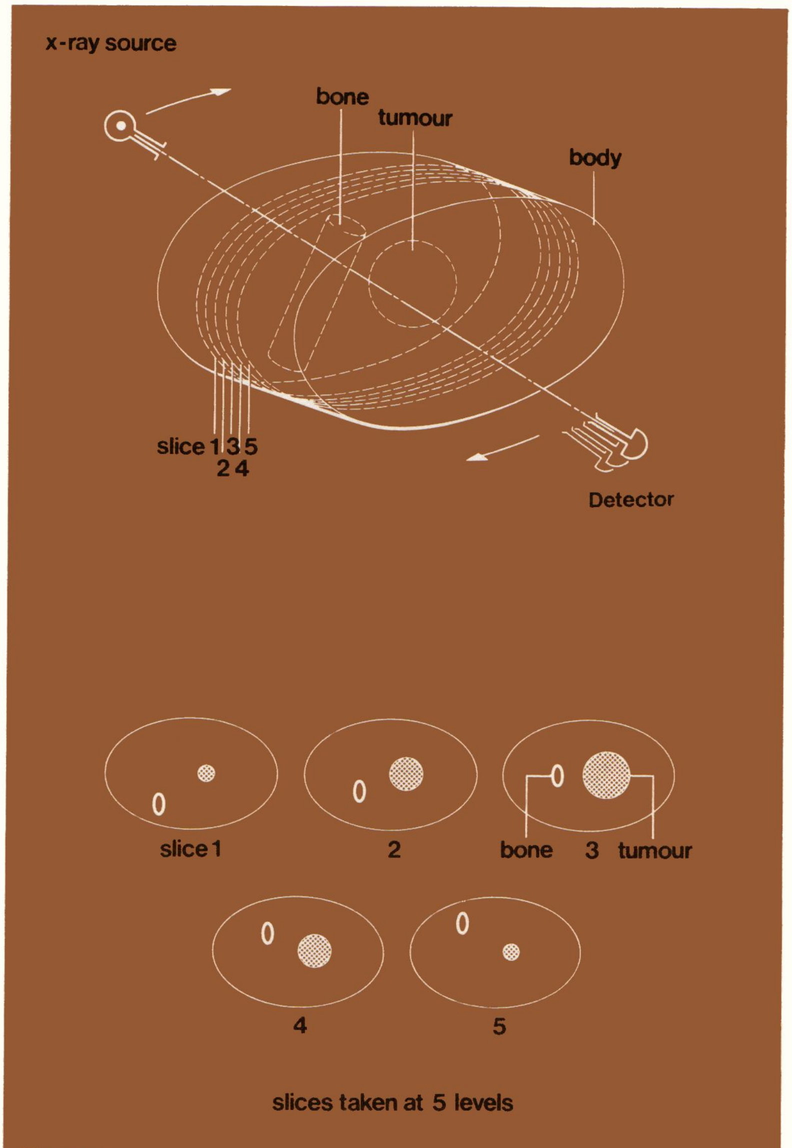

Figure 2

Operation of the system

The EMI-SCANNER system consists of a scanner unit (housing an X-ray source and accurately aligned detector units), a control unit, a computer unit and a viewing unit (Figure 3).

The equipment uses a narrow beam of X-rays to scan the patient's head in a series of 1 cm. wide slices; two adjacent slices may be taken simultaneously in the span of about five minutes. The rays are passed through the head and are detected by two sensing devices which always remain in alignment with the X-ray source. Both the X-ray tube and the detectors scan across the patient's head linearly taking 160 readings of transmissions through the head as shown on the diagram (Figure 4).

The X-ray scanning unit is then rotated 1° around the head and the process is repeated (Figure 4). This continues for 180 scans, when 28,000 readings will have been taken by each detector. These readings are then processed by a mini-computer which calculates 6,400 absorption values of the material within each slice from the 28,000 simultaneous equations generated.

The system yields about one hundred times more information than conventional X-ray systems and enables small variations in tissue density to be differentiated. The skin area irradiated is confined to a narrow band around the edge of the slice and the dosage is approximately equivalent to a conventional X-ray picture.

From the calculations performed by the computer, a picture may be built up in the form of a matrix (80x80) of 6,400 picture points, each indicating the value of the absorption coefficient of the corresponding material at each point in the slice. Alternatively, an optional additional facility can be supplied to reproduce the results as a numerical print-out of the absorption coefficients of the material at each point in the slice. This provides detailed information about the nature of the tissue and is a function of the density and atomic number of the material.

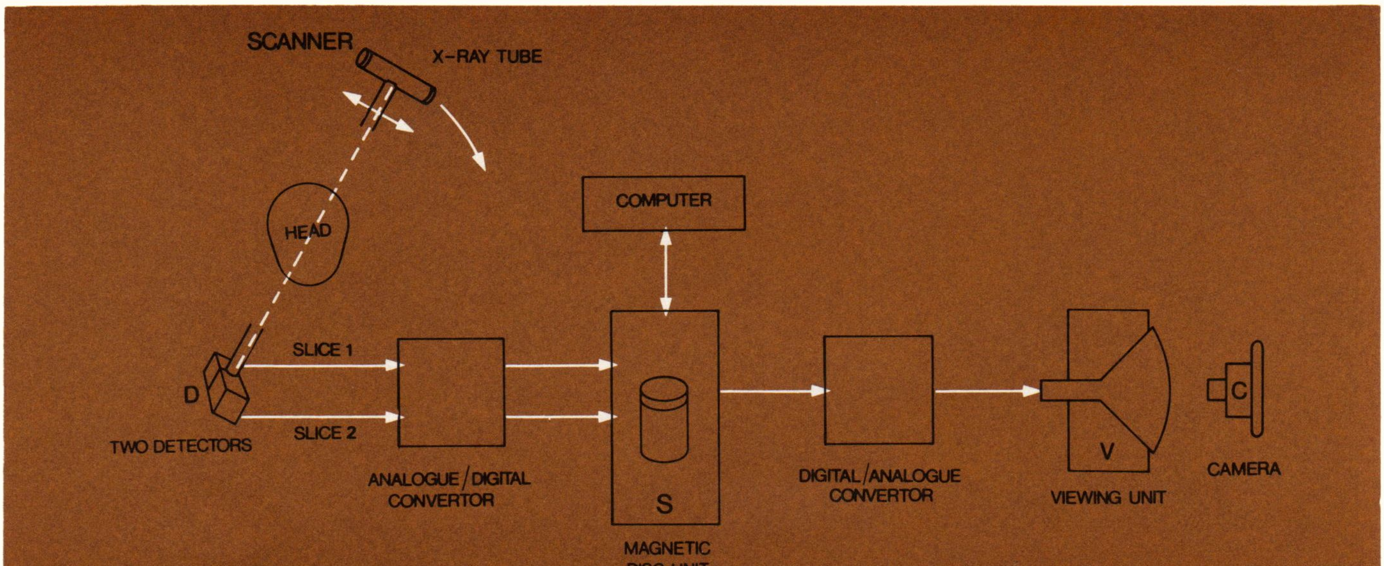

Figure 3

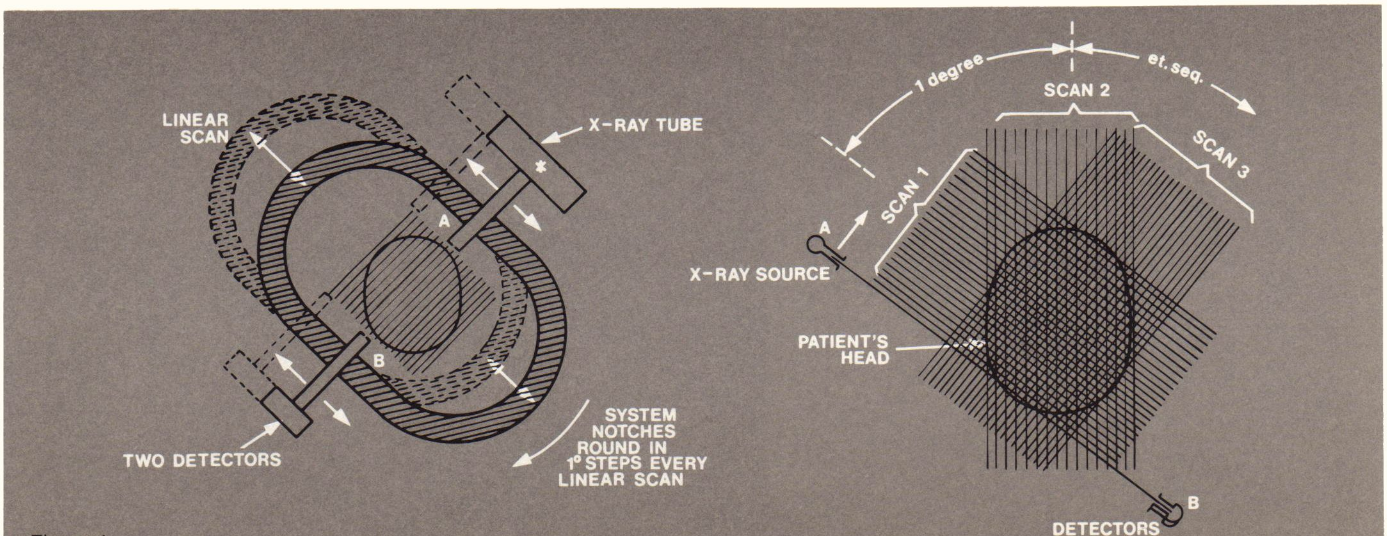

Figure 4

Some idea of the sensitivity and accuracy of the system can be demonstrated by the chart (Figure 5) which shows the percentage absorption coefficient relative to that of water for a number of materials commonly encountered in clinical radiology. It can be seen that on this scale, the absorption coefficient of fat is 10% less than that of water whereas the absorption coefficient of tissue found in the head varies between 2% and 6% greater than that of water.

For the examination of differing tissue types the machine sensitivity can be set by means of the "window width" switch so that this 4% range in absorption coefficient represents black to peak white on the cathode ray tube display. Other range values can be selected if required.

In addition to this, a further calibrated control, 'window level', enables the centre of the range selected by the 'window width' switch to be set at any desired point on the scale of absorption coefficient. Thus for tissue examination the window level control would be set at about the 8% level, whereas for examination of fat the control would be set at about – 10% in order to obtain the best display of absorption differences.

The chart also shows the relationship between the percentage absorption coefficients described above and the scale actually used on the controls and in the computer print-out.

In operation the equipment is straight-forward and an unaided radiographer can perform examinations on up to four patients per hour. The patient lies on the examination table with the head in a rubber head-cap in the scanner unit. It will be seen (Figure 7) that the patient is able to wear normal clothing and the position of examination is

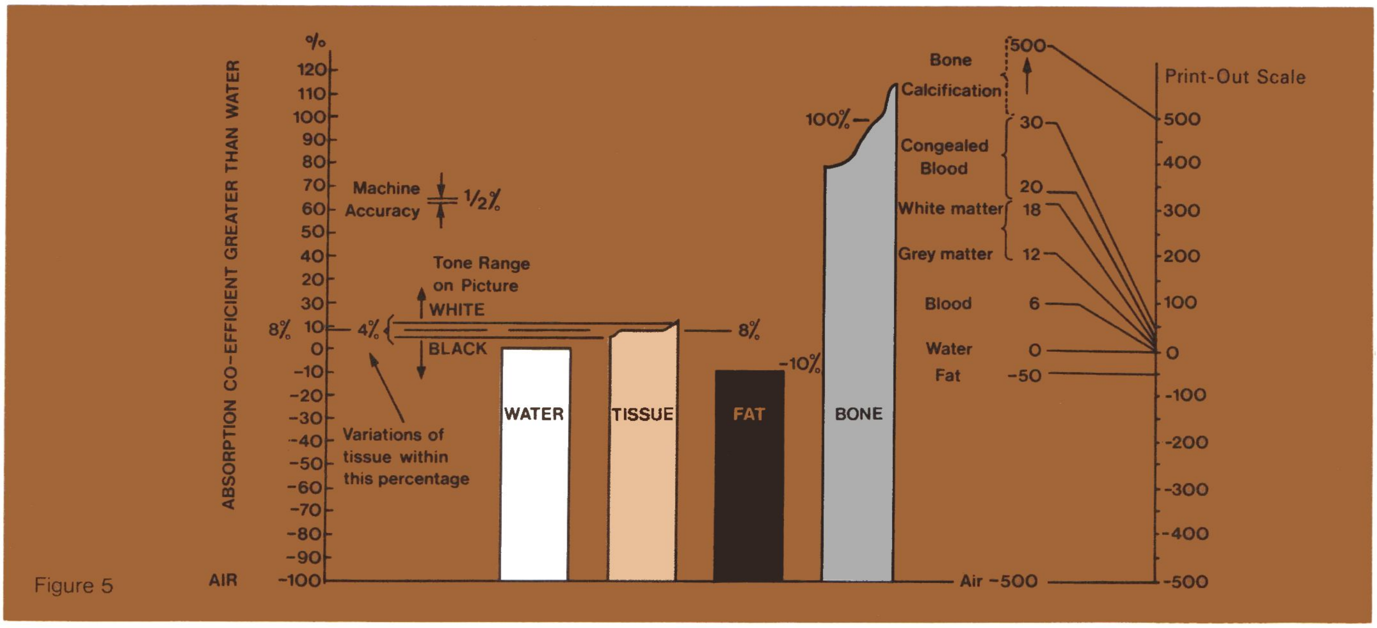

Figure 5

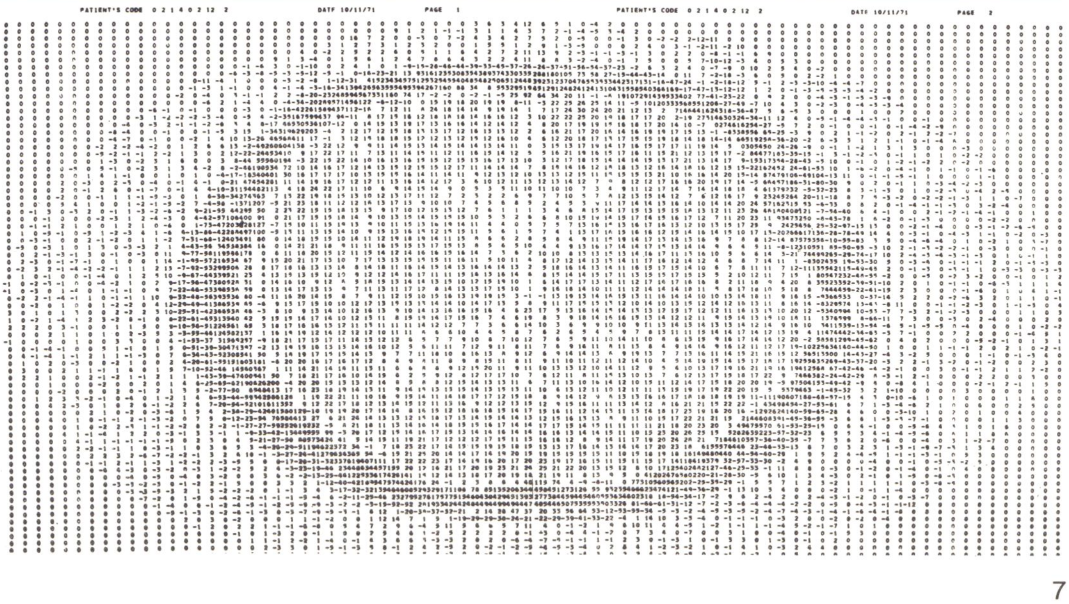

Figure 6

such that the patient is comfortable and relaxed. This permits considerable benefits, since the distress and possible hospitalisation resulting from conventional methods are completely eliminated.

The radiographer operates the scanner from the control unit in the protective cubicle as shown in the photograph (Figure 9). Simple controls are provided and a number of interlock arrangements ensure safe operation.

The results from scans are stored in a removable magnetic disc store. The readings from the previous X-ray investigation may be processed by the computer while the subsequent patient is being scanned. The computer takes approximately 7 minutes to perform the calculations required for each picture and the processed results are then stored on the magnetic disc for subsequent viewing, photography and analysis. The viewing unit is shown in the illustration (Figure 8). The results of a number of scans are illustrated in the photographs (Figure 10). All these results are displayed on the 80x80 matrix.

The smallest volume that can be detected would have an area viewed perpendicular to the slice of approximately 3mmx3mm the other dimension being the width of the slice which can be varied between 1.3 cm to 0.8 cm. As a general guide to diagnosis the computer print-out and the chart (Figures 5 and 6) should be studied. A single high number at least ten units above or below the rating of the surrounding tissue could indicate an abnormality but numbers with lower variation must be considered in groups covering larger areas.

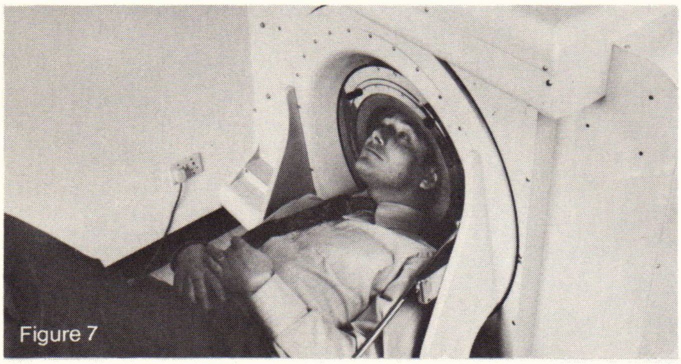
Figure 7

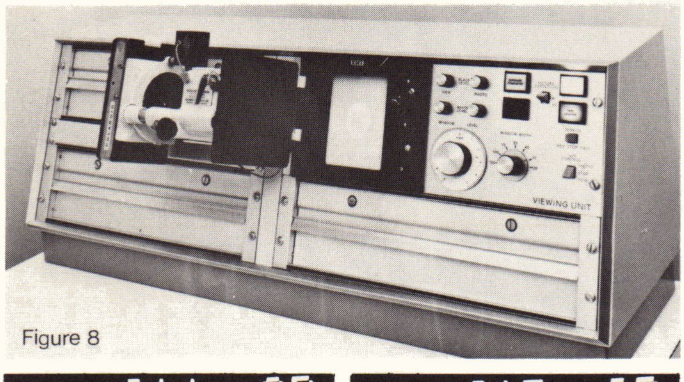
Figure 8

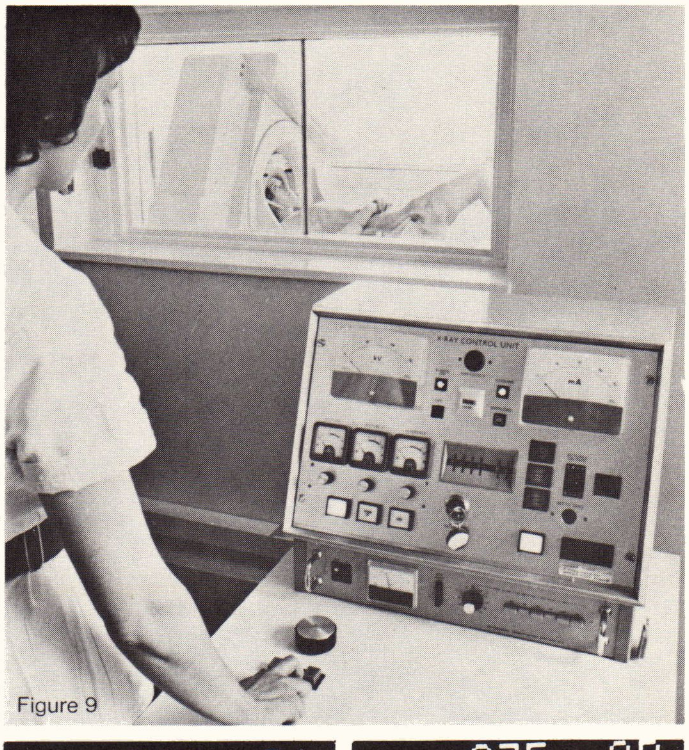
Figure 9

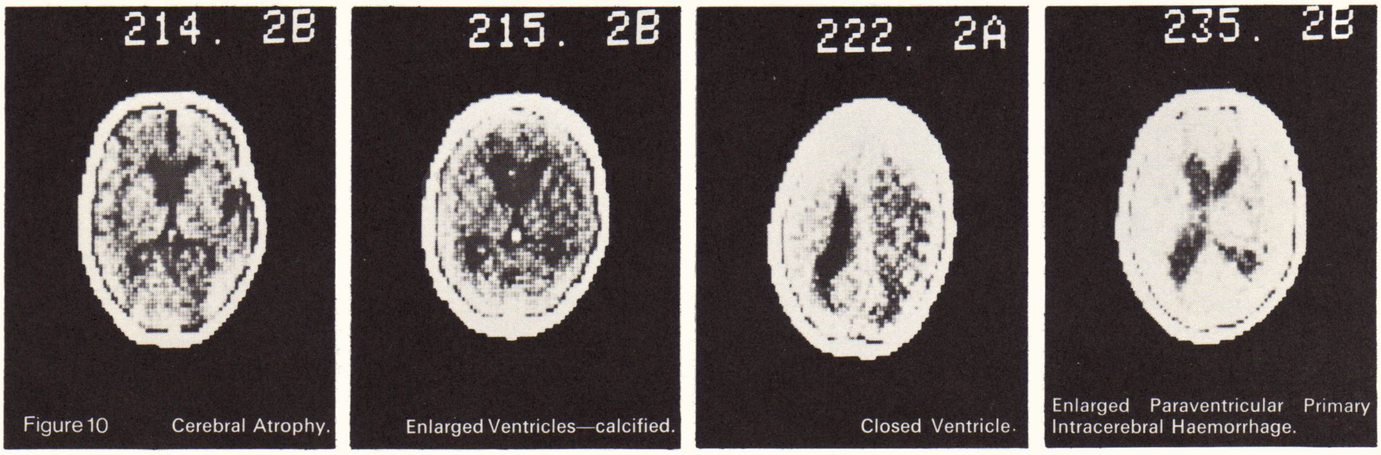

Figure 10 Cerebral Atrophy. Enlarged Ventricles—calcified. Closed Ventricle. Enlarged Paraventricular Primary Intracerebral Haemorrhage.

Control panels

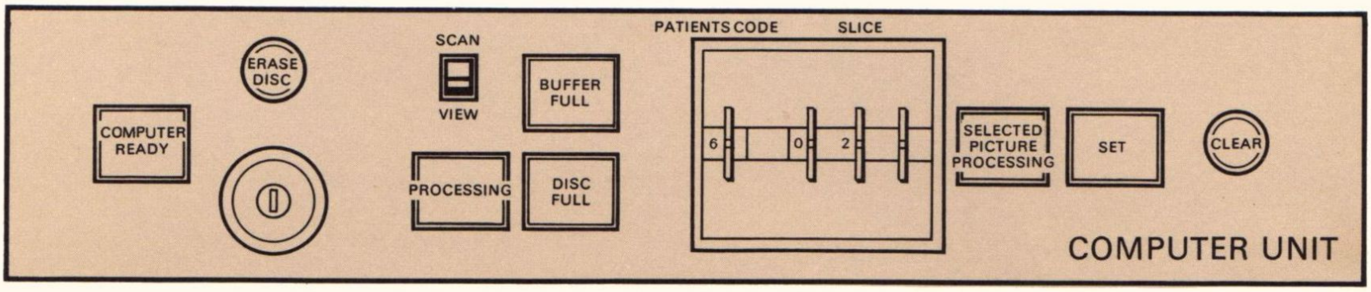

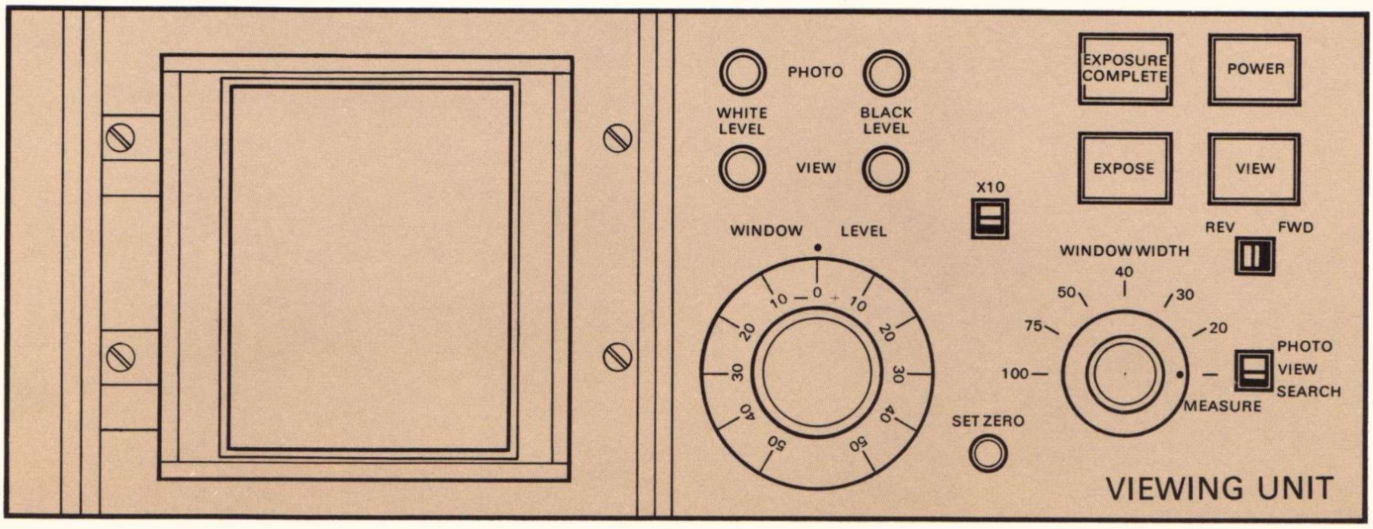

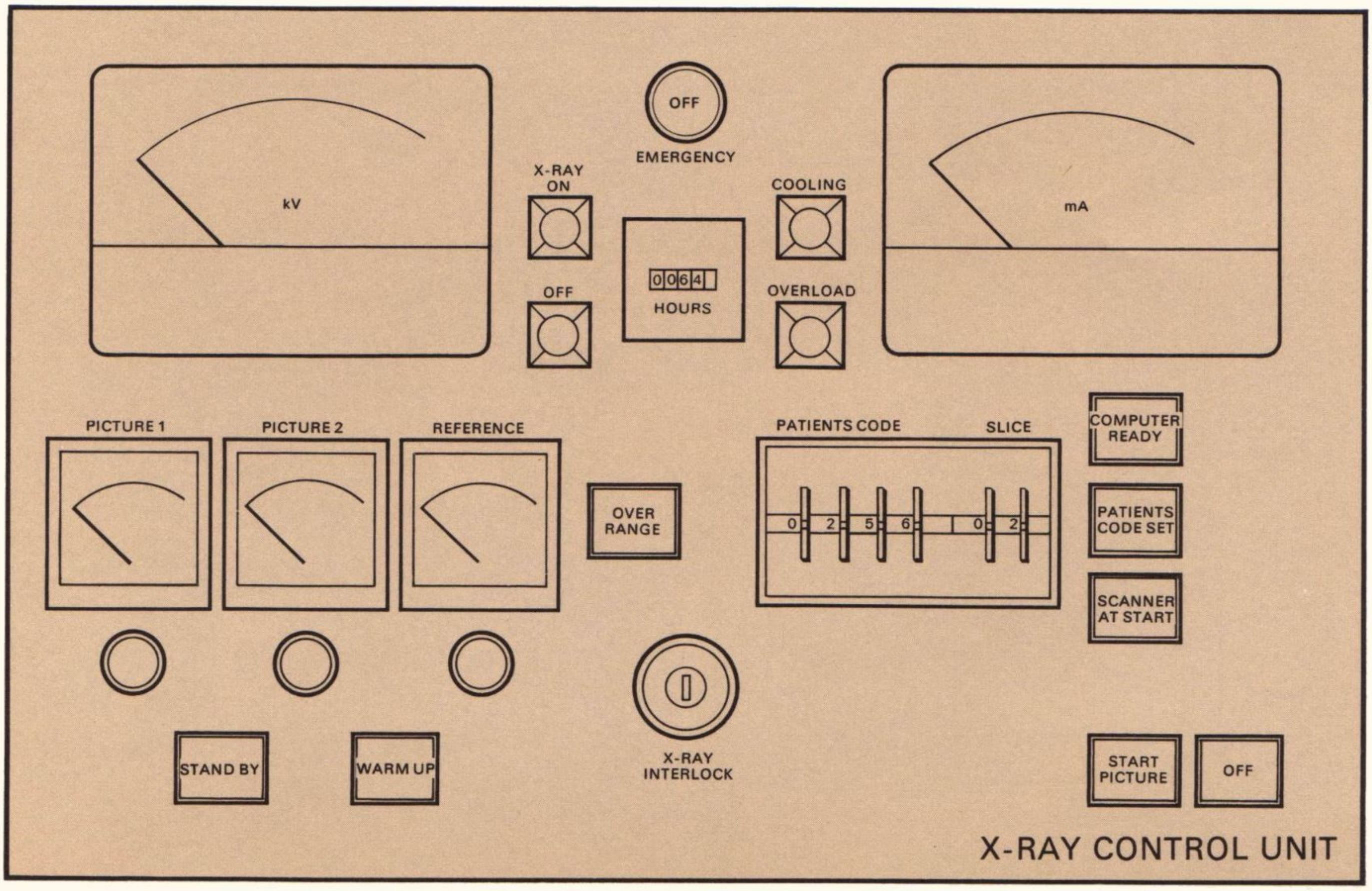

A typical layout

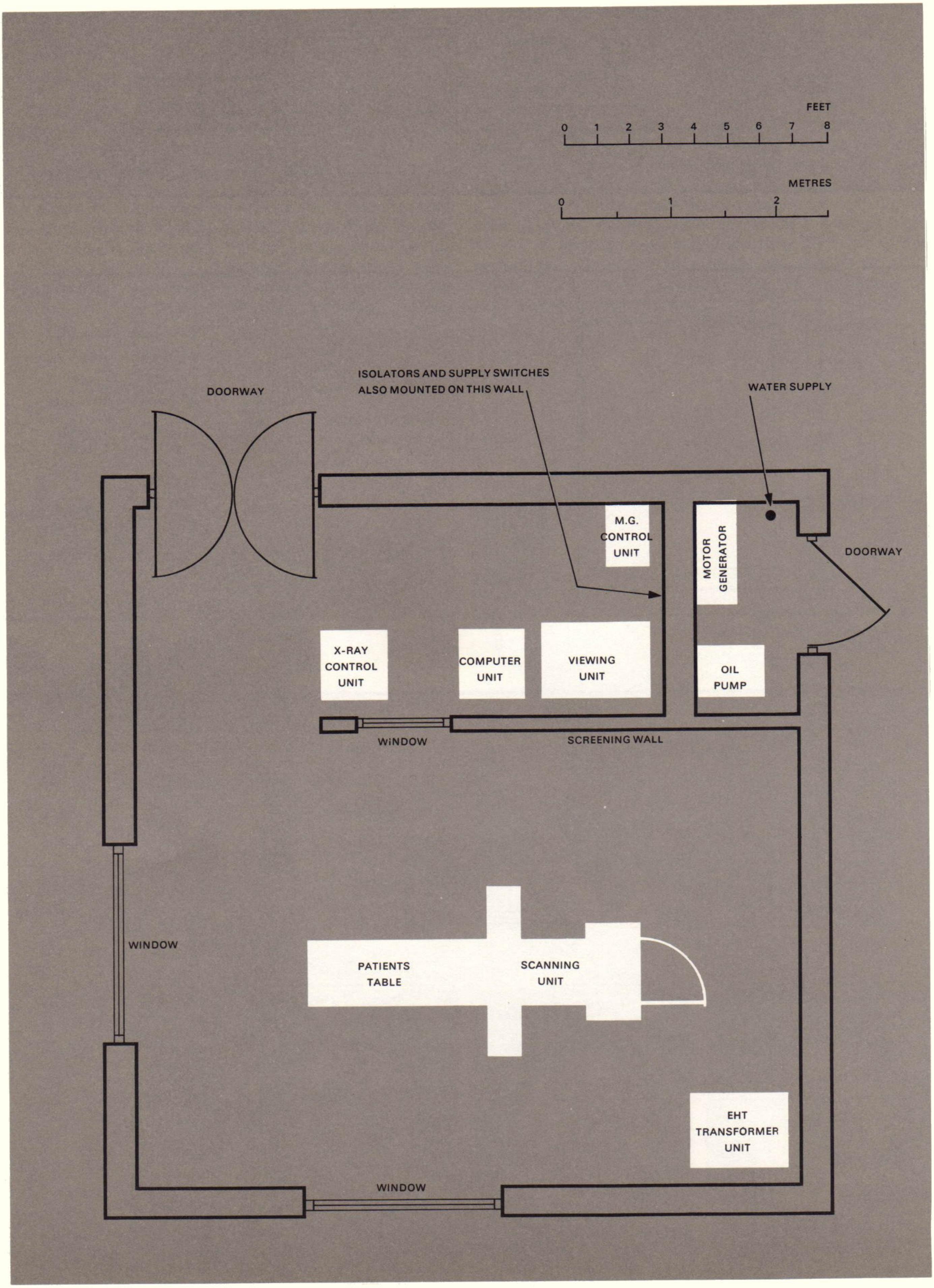

Specifications

Capability:
To be capable of simultaneously scanning two contiguous sections through a live human head and processing the readings taken to give a picture display on a viewing unit.

Range of X-ray absorption coefficient:
+500 to −500 (see the scale used on Figure 5)

Resolution of X-ray absorption coefficient:
$\pm\frac{1}{2}\%$

Time of single scan (2 contiguous sections):
Variable between approx. $4\frac{1}{2}$-20 minutes.

Radiation Dosage to the skin for a complete head examination of 3 or 4 scans each of 5.5 minutes duration:
120kV – Mean dose 1.25R Maximum dose 1.91R
140kV – Mean dose 1.55R Maximum dose 2.26R

Radiation Dosage to the skin for a complete head examination of 3 or 4 scans each of 4.0 minutes duration:
120kV – Mean dose 0.91R Maximum dose 1.39R
140kV – Mean dose 1.13R Maximum dose 1.64R

Gonad Dose for a complete scan:
Less than 0.1 mR.

X-ray tube Voltage/Current:
100kV – 40mA max.
120kV – 32mA max.
140kV – 27 mA max.

X-Ray tube focal spot size:
12 mm × 1.3 mm

Mean X-ray beam width:
0.8 cm or 1.3 cm

Detectors:
Sodium iodide crystals + photomultipliers

Rubber head cap life:
Up to three months depending upon use

Power requirements:
400 volts ± 10% 50HZ 3 phase supply mains, 7KVA.
230 volts ± 10% 50HZ single phase 4 kw.
or
190 volts ± 10% 60HZ 3 phase supply mains, 7KVA
110 volts ± 10% 60HZ single phase 4 kw.

Water supply to oil cooler:
To be capable of supplying a maximum flow of 4 imp/. pints/minute (2.3 litres/minute)

Initial warm-up time of X-ray tube:
Less than 10 minutes

Operating temperature range:
10°C – 35°C

Configuration of head receptacle:
23.5 cm diameter hole. The edge of the widest X-ray beam will scan the head approximately 2.4 cm from the edge of this hole.

X-ray tube leakage:
10 mR per hour at 1 metre from the tube (excluding scatter from patient)

Computation Time:
Approximately 7 minutes per picture

Storage disc capacity:
About 60 pictures

Picture Matrix:
80 × 80

Display Size:
Rectangular 140 mm x 101 mm ($5\frac{1}{2}$ inches x 4 inches)

Refresh rate:
Approximately 10 per second

Reference number display:
The picture reference number appears above the X-ray picture on the C.R.T. and shows the patient's number and slice number.
A and B represent lower and upper slice respectively.

Camera:
Polaroid Print size 76 mm x 95 mm (3 inches x $3\frac{3}{4}$ inches)

Sizes

Scanning Unit

Minimum Length required including space for table movement and equipment door opening:
393.4 cm (155 in)

Minimum Height required for rotation:
175.2 cm (69 in)

Minimum Width required for rotation:
152.4 cm (60 in)

Weight:
Approx. 635 kg (1400 lb.)

Maximum Floor Loading:
1.13 kg/sq cm (16 lb/sq in)

Viewing Unit

Maximum Width:
108.8 cm (42.87 in)

Maximum Height:
110.4 cm (43.5 in)

Maximum Depth including Desk:
68.6 cm (27 in)

Weight:
Approx. 159 kg (350 lb)

X-Ray Control Unit

Width:
61.2 cm (24.13 in)

Height:
123.1 cm (48.5 in)

Depth:
68.6 cm (27 in)

Weight:
Approx. 204 kg (450 lb)

Computer Unit

Width:
58.4 cm (23 in)

Height:
190.4 cm (75 in)

Depth (Cabinet):
64.8 cm (25.5 in)

Depth (including plinth):
74.9 cm (29.5 in)

Weight:
Approx. 272 kg (600 lb)

E.H.T. Transformer Unit

Width:
86.3 cm (34 in)

Height:
76.2 cm (30 in)

Depth:
68.6 cm (27 in)

Weight:
331 kg (730 lb)

Motor Generator Control Unit

Width:
53.3 cm (21 in)

Height:
45.6 cm (18 in)

Depth:
45.6 cm (18 in)

Weight:
Approx. 45.4 kg (100 lb)

Motor Generator Unit

Width:
91.4 cm (36 in)

Height:
41.9 cm (16.5 in)

Depth:
41.9 cm (16.5 in)

Weight:
254 kg (560 lb)

Cooling Oil Pump Unit

Width:
60.9 cm (24 in)

Height (including stand):
183.0 cm (72 in)

Depth:
60.9 cm (24 in)

Weight:
Approx. 227 kg (500 lb)

Optional Extras

Computer printing unit

Replacement rubber head caps

Additional magnetic discs (one disc is supplied with the standard system)

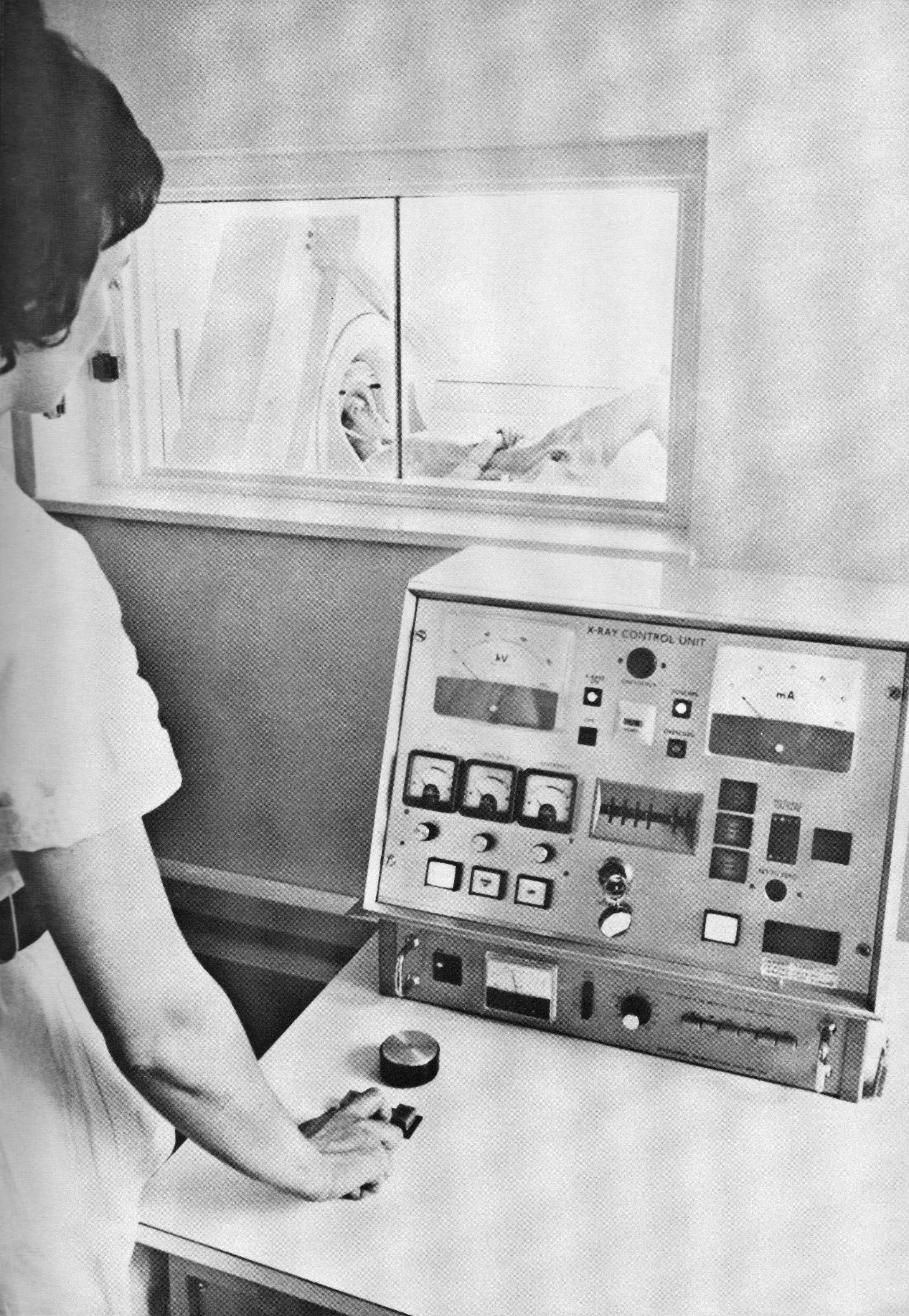
X-RAY CONTROL UNIT
kV
mA
EMERGENCY
COOLING
OFF
OVERLOAD
SET TO ZERO

EMI Limited,
Central Research Laboratories,
Shoenberg House, Trevor Road,
Hayes, Middlesex, England
Telephone : 01-573 3888 Extension 2182

A member of the EMI group of companies.
International leaders in Electronics, Records and Entertainment

The Company reserves the right to modify designs and specifications without notice.

Printed in England Lithofast 6m/10/72

CT/T total body scanner

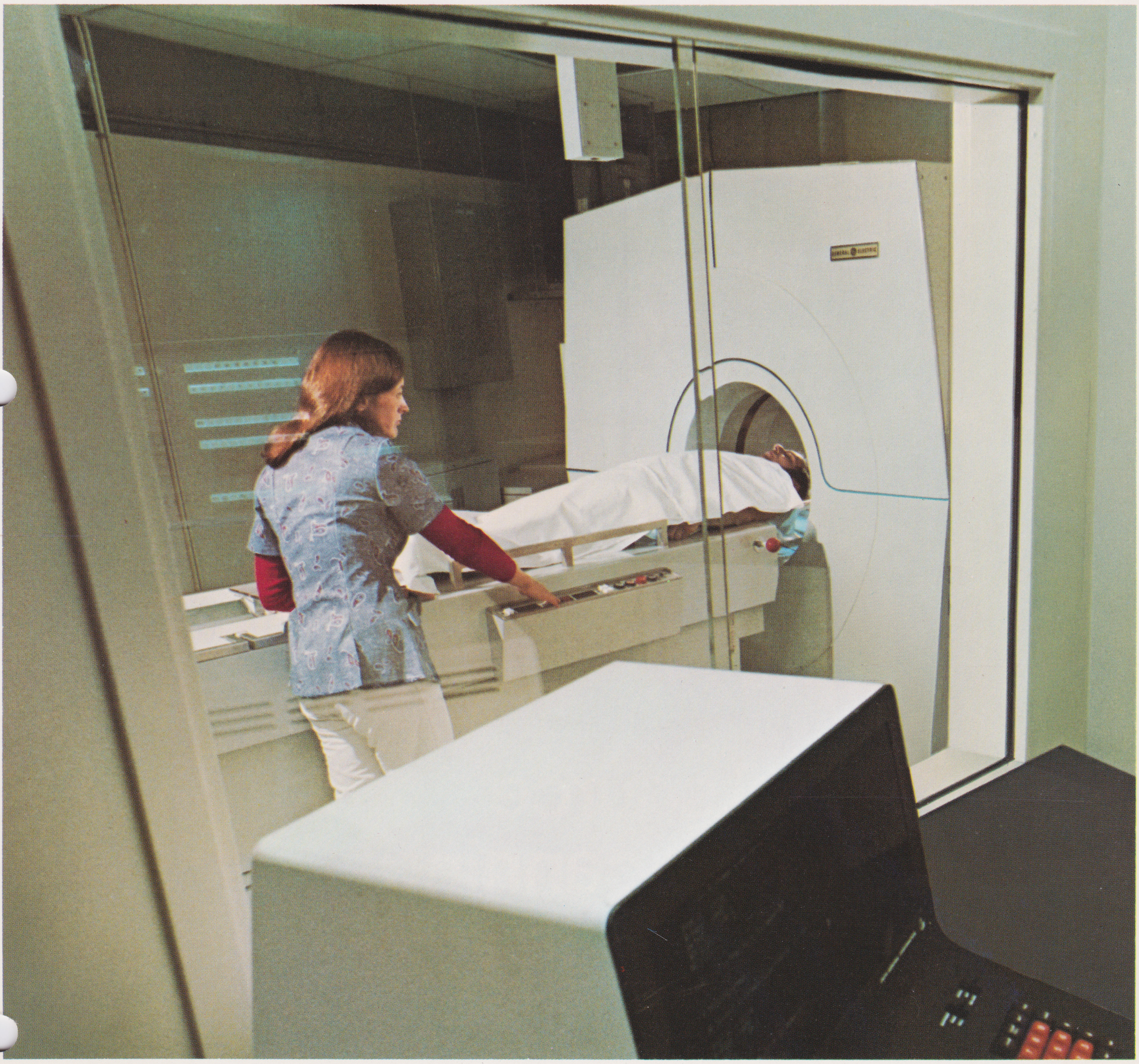

GENERAL ELECTRIC

AS&E®
CT Scanner

©1976 American Science and Engineering, Inc.

AS&E®
CT Scanner

The AS&E® CT Scanner utilizes a unique Stationary Circular Array of detectors with a single rotary motion of an intense X-ray source to acquire high quality images in scan times as short as five seconds. Its advanced geometry and stable mechanical design make possible the generation of artifact-free images with intrinsically high spatial and contrast resolution. These features result in CT images of unequalled quality for both head and body examinations.

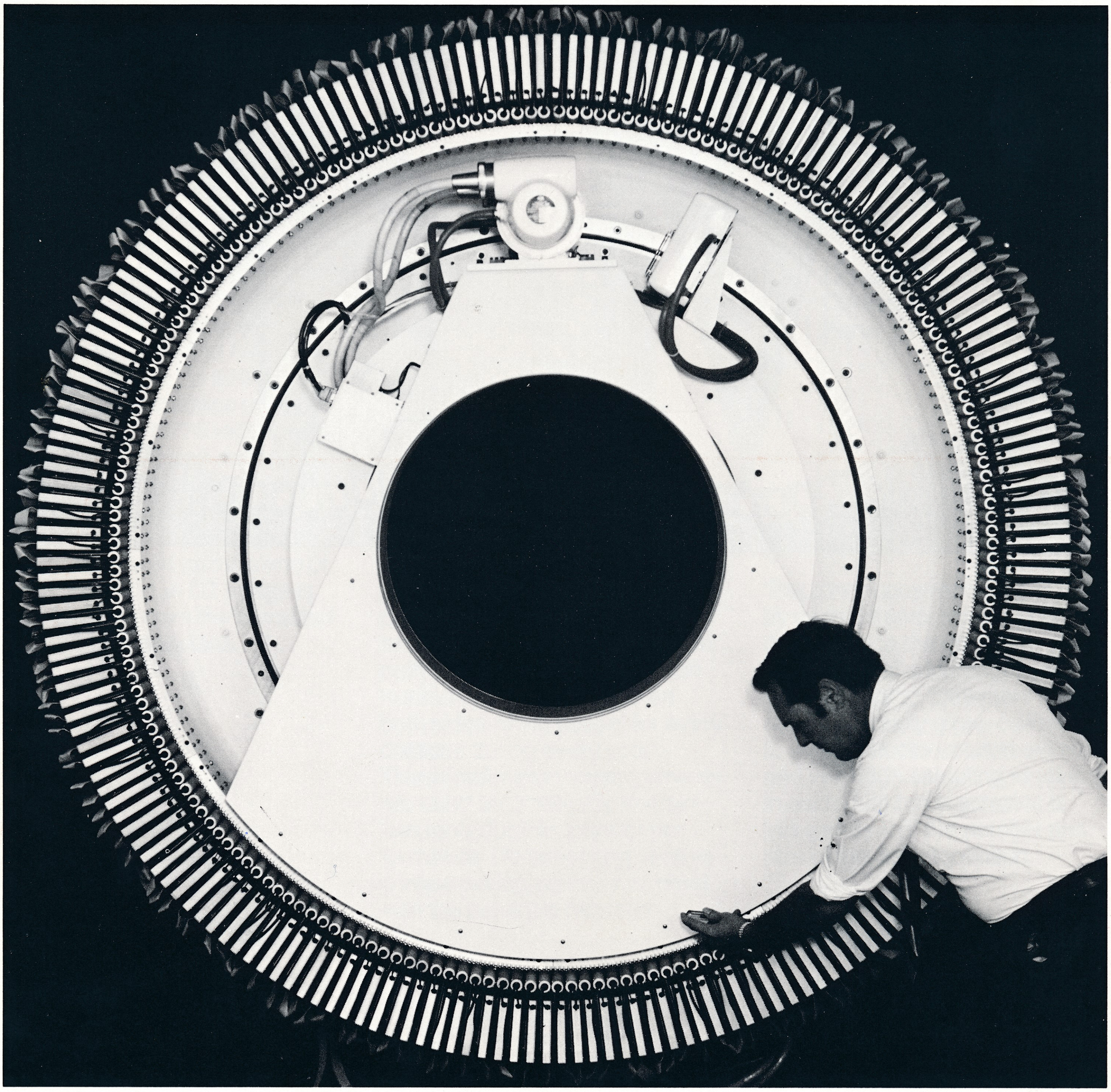

How the System Works

The principle of operation of the AS&E® CT Scanner is illustrated here. A Stationary Circular Array of 600 scintillation detectors encircles the patient in the plane of the slice. A high-power rotating anode X-ray source, operated at up to 150 KV and 100 MA in a continuous (non-pulsed) mode, rotates rapidly around the patient inside the detector array. The angular extent of the X-ray fan beam is 50° for body scans and 25° for head scans.

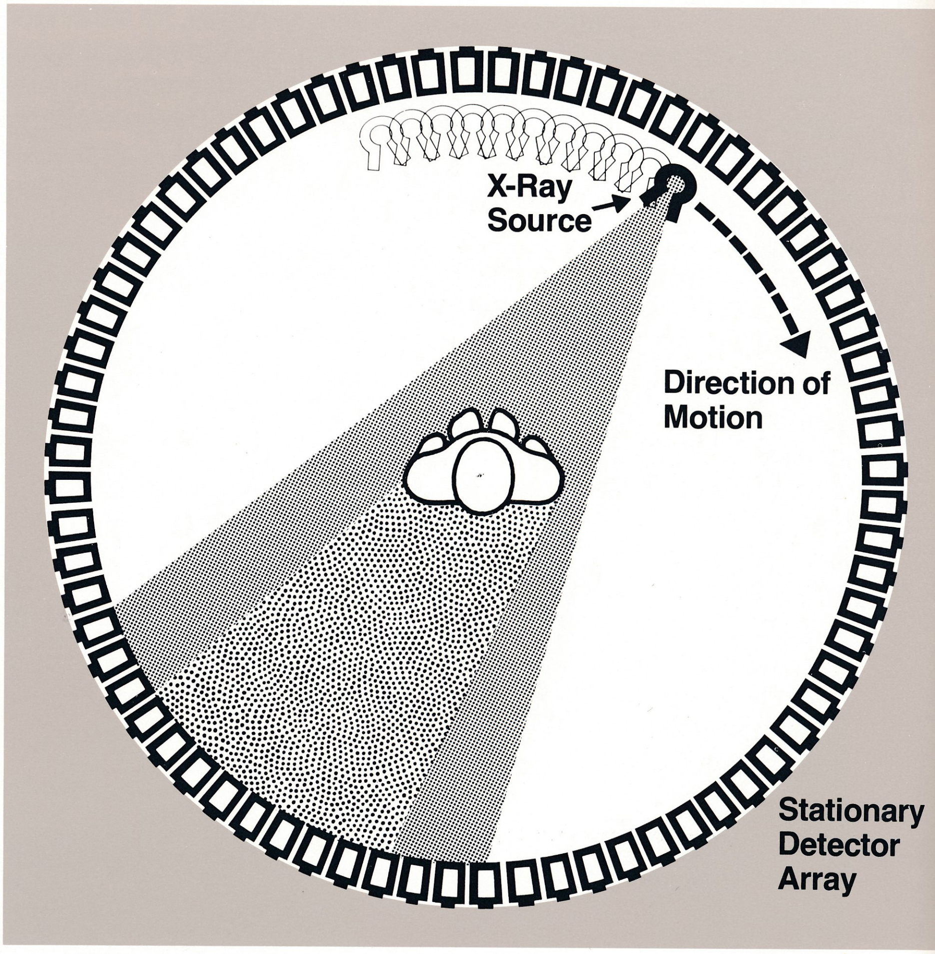

The many discrete X-ray absorption measurements recorded by each one of the 600 detectors during a scan are represented by the lines in the illustration. Each detector records measurements in the plane of the slice across the entire patient. The set of discrete measurements recorded by each detector is referred to as a data fan.

Rotation of the source through an angle well in excess of 360° is affected on all scans to provide overscan data which is used to nearly eliminate the effects of patient motion, already small in the AS&E system. A complete scan results in the acquisition of 675 data fans, the first 600 of which correspond to 600 different views of the slice under examination. The 75 redundant data fans, measured during the overscan, are combined with the first 75 measured data fans to compensate for patient motion between the start and the end of the scan period. A CT image is reconstructed from the corrected set of 600 full data fans.

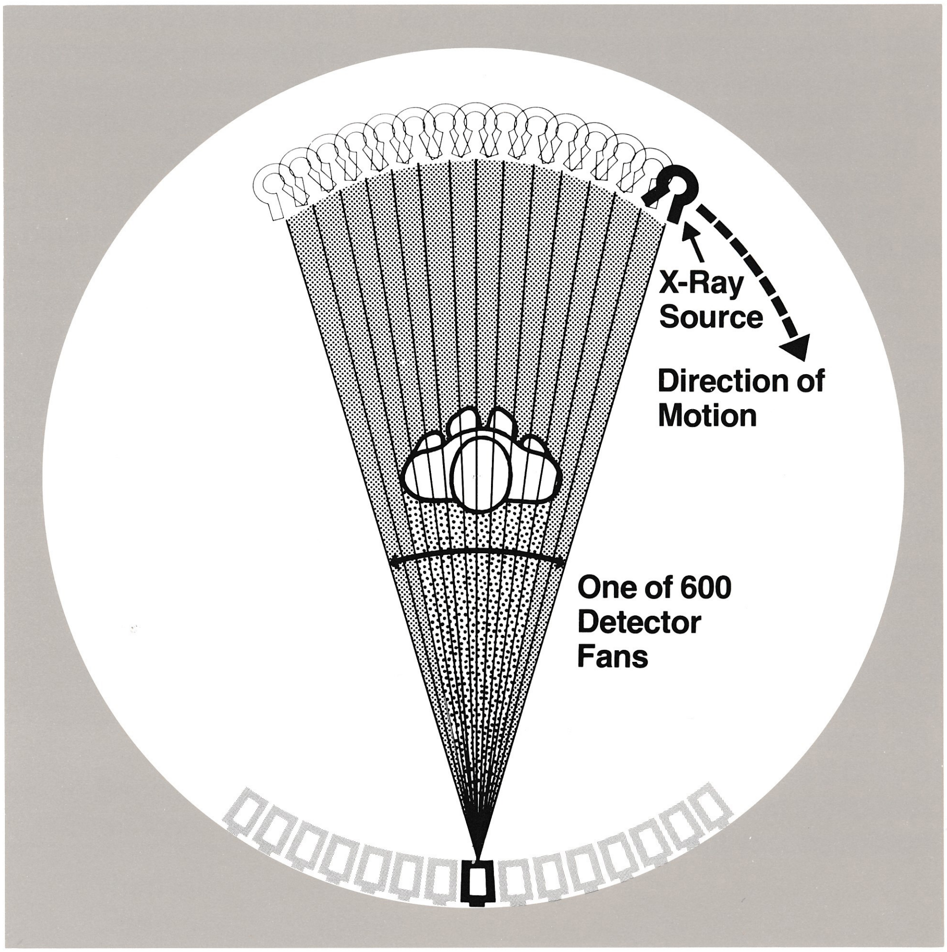

As shown in the illustration, each detector records measurements extending beyond the patient on both sides. Detector calibration measurements are made on both sides of the patient for every detector during every scan. These calibration measurements are used automatically to maintain reproducible scanner performance. No water bag or other compensating device in contact with the patient is required.

The continuous recording of data by each detector from one side of the patient to the other provides two essential advantages for high quality imaging. Detectors need not be precisely cross-calibrated to avoid detector-related artifacts, and very high sampling resolution in each data fan is achievable independent of the number and size of the detectors used.

Scanning Modes

The AS&E® CT Scanner provides three nominal scan times for both head and body scanning: 5, 10, and 20 seconds. The number of measurements recorded in each data fan increases with increasing scan time. To the extent that patient motion is not a factor, longer scan times yield higher sampling resolution and better images.

For body scans, a 48cm (19in) diameter circle is imaged and the normal scan time is 10 seconds. The 5-second body mode is intended for patients for whom breath holding or other motion problems exist. The 20-second body mode can be used to achieve very high sampling resolution, provided extra care in patient management is exercised to avoid motion.

For head scans, a 24cm (9.5in) diameter circle is imaged. The normal scan time for head examinations is also 10 seconds. The 5-second mode may be used for patients who are difficult to immobilize. The 20-second head mode affords extremely high sampling resolution for special examinations.

In the normal 10-second scan mode, 511 separate data measurements are recorded by each detector. For body scans, these measurements cover the 48cm (19in) diameter patient circle and are taken at intervals of 1mm; for head scans, these 511 data measurements cover the 24cm (9.5in) diameter patient circle and are taken at intervals of .5mm. In the 5-second mode the number of data measurements is half the number for the 10-second mode. In the 20-second mode, the number of data measurements is twice that for the 10-second mode.

Image Reconstruction

In normal clinical operation, images are reconstructed in one minute as an array of 512 by 512 computed picture elements. The image is displayed on a 512 by 512 matrix corresponding to a 48cm (19in) diameter field for body scans and to a 24cm (9.5in) diameter field for head scans. Each pixel in the normal body image represents an area of 1mm by 1mm and in the normal head image an area of .5mm by .5mm.

A capability is also provided to reconstruct a square of arbitrary size on the full 512 by 512 matrix. This regional reconstruction feature may be used, for example, in the very high resolution, 20-second scan modes to reconstruct a selected small region of the field on the full 512 by 512 matrix. All picture elements in the 512 by 512 image are computed independently for regional reconstruction.

Number of Measurements Recorded by Each Detector

	5-Second* Scan time	10-Second* Scan time	20-Second* Scan time
Data Measurements for Each Fan	255	511	1023
Calibration Measurements for Each Fan	44	88	176

Total Measurements Made for Each Slice Including Overscan (Body and Head Scans)

	5-Second* Scan time	10-Second* Scan time	20-Second* Scan time
Data Measurements	172,125	344,925	690,525
Calibration Measurements	29,700	59,400	118,800
TOTALS	201,825	404,325	809,325

Distance Between Measurements

	5-Second* Scan time	10-Second* Scan time	20-Second* Scan time
Body Scan	2mm	1mm	.5mm
Head Scan	1mm	.5mm	.25mm

*Nominal

AS&E®
CT Scanner System

The basic system configuration for the AS&E® CT Scanner is shown in the block diagram below. Two separate minicomputers provide independent scanning and viewing capability in order to maximize patient throughput. The scanner computer controls patient scanning and image reconstruction and is operated from a single Operator Console. The Operator Console includes a keyboard terminal for entry of patient identification information. The display computer controls image viewing functions, hard copy generation, image manipulation, and data storage and retrieval and is operated from an independent Display Console.

In normal clinical operation, the patient is positioned in the scanner at the location for the first slice with the aid of a scan-centered light beam. All subsequent scanning operations, including slice-to-slice indexing, are controlled from the Operator Console. Each scan is reconstructed on a 512 by 512 matrix one minute after the scan, using special arithmetic processors. Shorter reconstruction times may be obtained by selecting a smaller, 256 by 256, matrix.

Immediately after reconstruction, the image is displayed for verification by the operator on a Quick Look Display with a 256 by 256 matrix. This feature allows the operator to confirm all aspects of the system, including patient positioning, before proceeding to the subsequent slice. The patient couch is indexed automatically and the second scan slice can be initiated immediately after reconstruction of the first.

Special procedures are controlled from The Operator Console keyboard. These include provisions for storing raw data from a scan, entering user-oriented reconstruction algorithms, reconstructing images on other than standard field and matrix formats, and running routine maintenance programs. In addition, the keyboard allows storage of raw data from a scan before or after reconstruction. Storage prior to reconstruction allows scans to be taken at less than one-minute intervals.

In normal clinical operation, the reconstructed 512 by 512 image is held on a large disk system with a capability of storing up to 125 images for instant recall. Viewing of these images and hard copy generation may take place simultaneously with scanning.

A separate system controls all display functions. It includes a high resolution television display for viewing reconstructed 512 by 512 images, a multi-image format hard copy transparency camera, a nine-track magnetic tape drive, a line printer, and a Display Console. A Polaroid hard copy camera and a floppy disk unit for patient oriented image storage capability are available as options.

Viewing and image manipulation are accomplished at the Display Console. Window level and width, cursor control, and hard copy generation are achieved by using simple controls.

The Display Console also includes a keyboard terminal for image recall from the disk. Specialized functions are also accessed using the keyboard. These include magnetic tape operation for long-term storage and retrieval of images, statistical image analysis program operation, diagnostic program operation, and line printer operation for print-out of numerical densities.

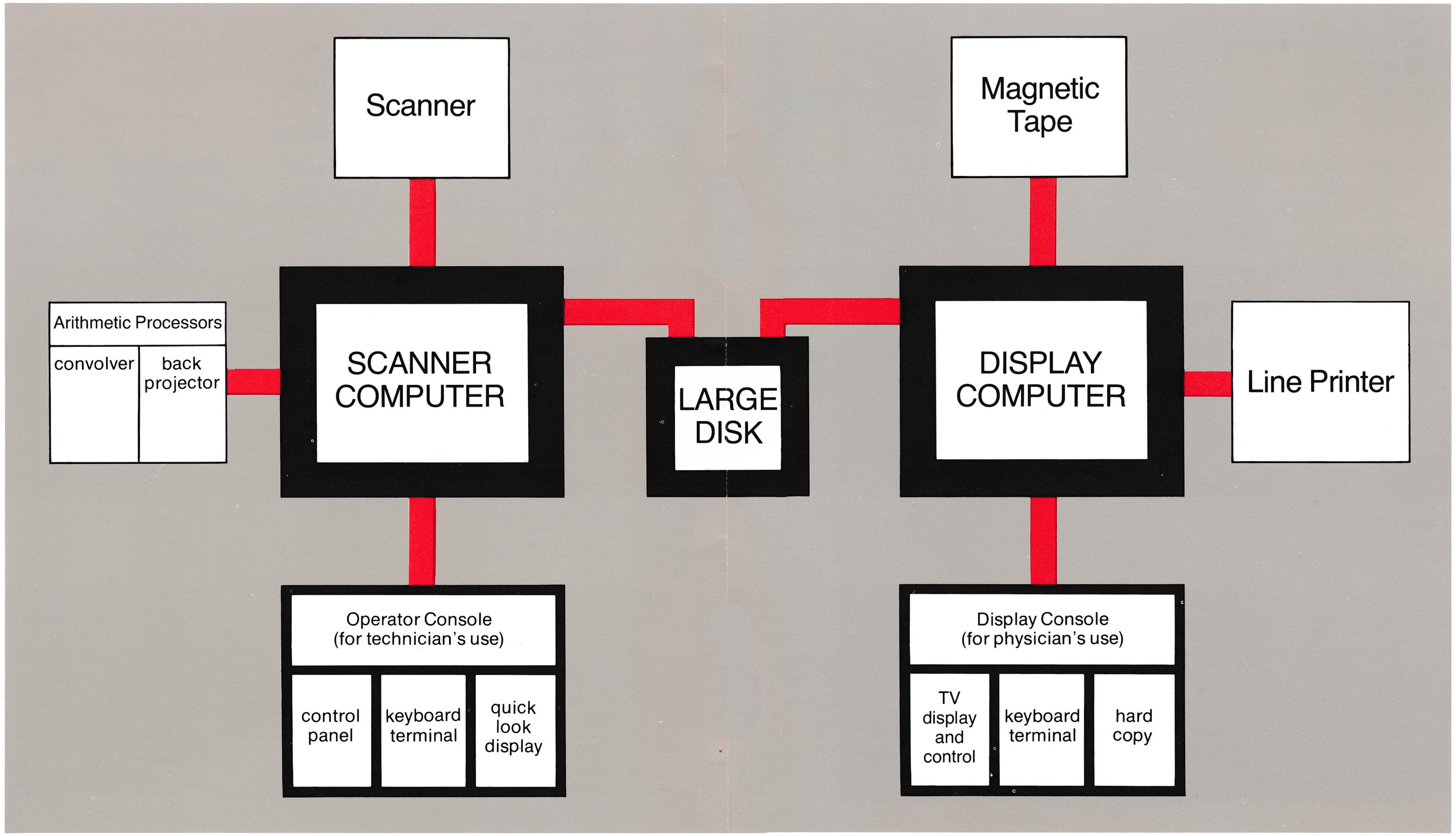

Two independent Computer Systems provide simultaneous scanning and viewing

Operator Console Control Panel

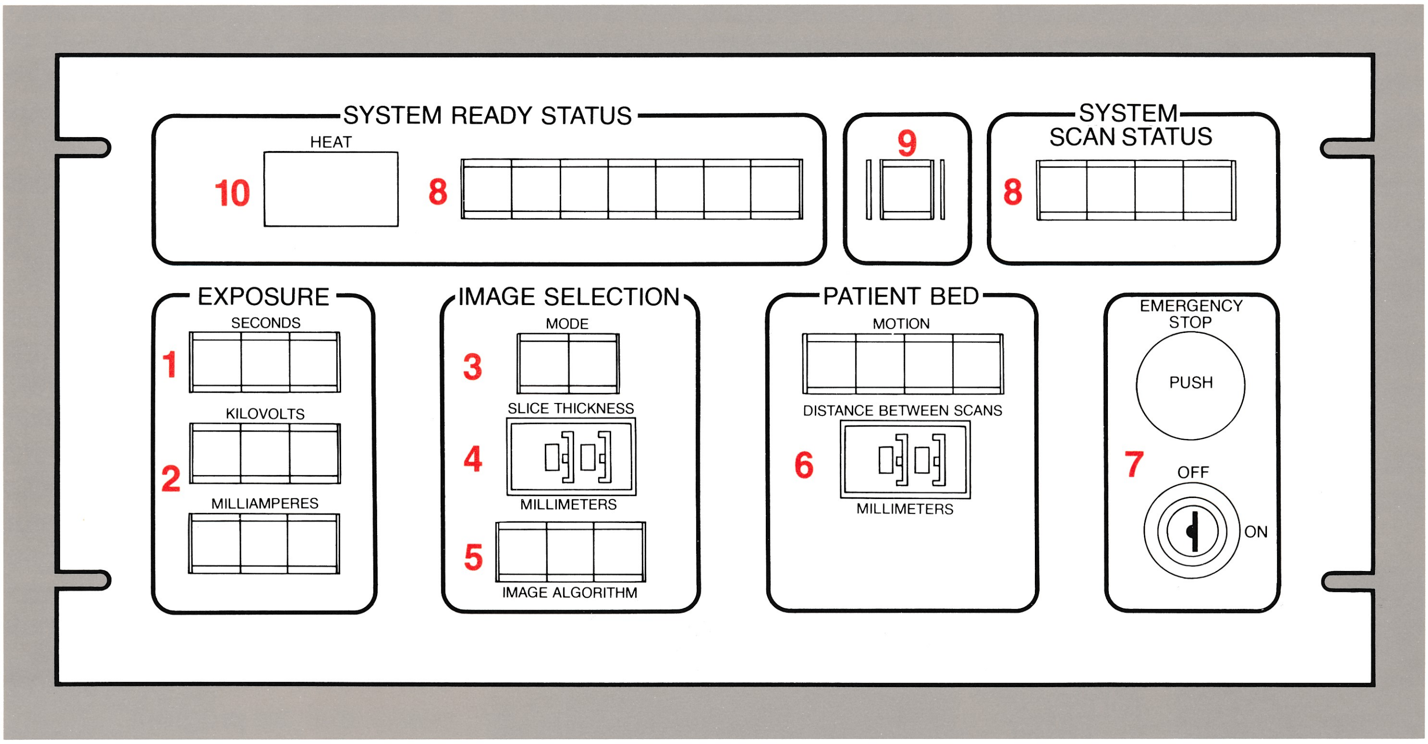

1 Seconds—Scan time is operator selectable.

2 Kilovolts and Milliamperes—KV and MA are operator selectable to optimize image quality and radiation dose. Three settings of both KV and MA are available. Values other than the normal settings shown are obtainable.

3 Mode—Head or body operation is operator selectable.

4 Slice Thickness—Slice thickness is operator selectable from 2 to 10mm.

5 Image Algorithm—A choice of reconstruction algorithms is provided to give flexibility for obtaining optimum image quality. Provision is made for selecting reconstruction algorithms according to user preference. Reconstruction is achieved by the technique commonly referred to as "convolution and backprojection." Algorithm selection is achieved by changing the convolution filter coefficients.

6 Patient Bed—The distance between slices is operator selectable. The patient couch normally indexes automatically between scans. Forward/reverse, repeat slice, and manual indexing controls are also provided.

7 Emergency Stop—Emergency stop and key lock are provided.

8 System Ready Status and System Scan Status—Full Scanner status is continuously indicated.

9 Start Scan—Scan initiation is accomplished with one push-button.

10 Heat—X-ray tube heat loading is displayed continuously.

Display Console

The Display Console contains all display functions in one location for maximum flexibility.

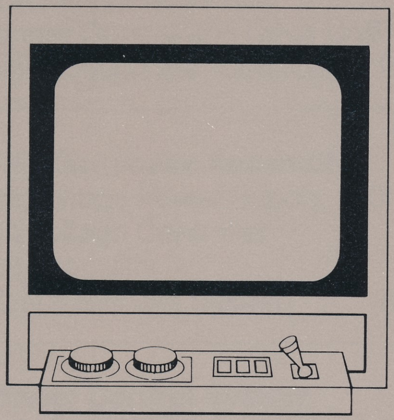

Television Monitor

Continuously variable window level and window width controls are provided. Level and width settings are displayed continuously on the television monitor.

Joystick control of a cursor is provided. The cursor may be used to identify a region, the average density of which is desired. It also may be used to identify the center of a region about which a zoom magnification is desired.

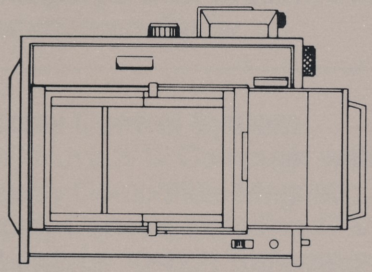

Hard Copy

Hard copy may be obtained by push-button control.

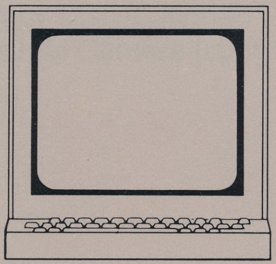

CRT

All image retrieval functions are controlled from the CRT display keyboard.

Routine Maintenance

In normal operation, the AS&E® CT Scanner does not require a routine calibration procedure since all detectors are calibrated automatically on every scan. However, special maintenance programs are supplied to assure that the scanner provides optimum performance.

1 X-ray tube alignment verification is accomplished automatically by scanning an Alignment Phantom supplied with the scanner.

2 A Detector Diagnostic Program is supplied to isolate faulty detector units. Detectors that are not operating properly are automatically eliminated from the image reconstruction process. The AS&E® CT Scanner has been designed to operate without detectable image degradation in the event of failure of up to 20 detector units.

Deliverable Hardware

Scanner Assembly
600-Detector Stationary Circular Array
Rotor Assembly including Laser Alignment Assembly
Cable Handling Assembly including High Voltage Cables
Eimac Rotating Anode X-Ray Tube/Housing or equivalent with Heat Exchanger Assembly
Scanner Control and Drive Unit
Patient Couch with Controls

X-Ray Generator Assembly
High Voltage Power Supply, 150KV, 100MA
High Voltage Controller
Heat Integrator
Safety Interlocks System

Scanner Control System
DGC Nova 3/12 Computer with 32K Word Memory or equivalent
Convolver
Back Projector
Operator Console including Alphanumeric Display with Keyboard plus 9-inch TV Monitor for Quick Look Displays
Paper Tape Reader
80 Megabyte Disk System, Dual Port Control
Interprocessor Buffer

Display Control System
DGC Nova 3/12 Computer with 16K Word Memory or equivalent
Industry Compatible Magnetic Tape System
Display Console including 17-inch TV Monitor, Multi-image Format 8-inch by 10-inch Transparency Camera, and Alphanumeric Display with Keyboard
Display Processor
Line Printer
Modem
Floppy Disc (optional)
Polaroid Camera (optional)

This list is subject to change without notice.

AS&E®
CT Scanner
Standard Specifications

Scanner geometry: Stationary Circular Array of 600 scintillation detectors concentric with rotating X-ray source.

Modes: Body and Head, selected by operator.

Fan angle: 50° for whole body scans, 25° for head scans; automatically set by mode selection.

Field size: Body scans: 18.8-inch diameter circle; Head scans: 9.6-inch diameter circle.

Scan time: Body and Head Scan: 5, 10, or 20 seconds, selected by operator. (Actual scan times may differ from nominal values by 10%.)

Detectors: 600 Stationary Bismuth Germanate scintillation detectors.

Data acquisition: 675 data fans corresponding to 600 views (in 360°) plus 75 overscan views. Up to 1023 measurements per view in the 20 second mode, producing 690,525 measurements per slice, including overscan. For the 5 and 10 second modes, the measurements made are 172,125 and 344,925 respectively.

Reconstruction matrix: 512 by 512 standard (256 by 256 selectable).

Data reconstruction time: One minute for full 512 by 512 matrix.

Slice thickness: 2mm to 10mm at center of patient circle, selected by operator.

Diameter for patient clearance: 26 inches.

Ray displacement between measurements:

Scan Mode	5 Seconds	10 Seconds	20 Seconds
Head	1.0mm	0.5mm	0.25mm
Body	2.0mm	1.0mm	0.5mm

X-Ray source:

KV:	100KV, 125KV, or 150KV (alternate values are available), selected by operator.
MA:	20MA, 50MA, or 100MA (alternate values are available), selected by operator.
Focal Spot Size:	1mm x 1mm nominal.
Loading/Cooling:	400,000 heat unit target capacity; 75,000 heat unit/minute cooling rate; automatic heat integrator to protect against thermal overload.

These specifications are subject to change without notice.

Scale of X-Ray absorption values: −1000 to +2000 where −1000 is equivalent to air, 0 is equivalent to water.

Patient couch tilt: ±20°, selected by operator.

Patient couch indexing: Automatic or manual, from 1 to 10mm per slice, selected by operator.

Control console: A desk-type console containing an alphanumeric 9-inch CRT display with keyboard, system control switches and indicators, and a separate 9-inch TV monitor for quick-look display.

Display console: A desk-type console containing an alphanumeric 9-inch CRT display with keyboard plus the following: (a) 17-inch black-and-white TV monitor for main display; (b) 9-inch black-and-white TV monitor for hard copy display; multi-image format on 8-inch by 10-inch transparency (4-inch by 5-inch Polaroid optional); (c) continuously variable window level and window width control; (d) joystick cursor control for location of density read-out and zoom center.

Computer system: Data acquisition and image reconstruction simultaneous with picture display capabilities. One CPU with high-speed arithmetic processor for picture reconstruction plus one CPU with constant refresh display processor for image display.

Disk storage: 125 images (512 by 512).

Magnetic tape storage: 35 images (512 by 512) per 2400-foot reel.

Floppy disk storage (optional): One image (512 by 512).

Modem: Employed for remote control and diagnostics.

Power requirements: See AS&E® CT Scanner Technical Bulletin DS 801-030.

Environmental requirements: See AS&E® CT Scanner Technical Bulletin DS 801-030.

Space requirements: See AS&E® CT Scanner Technical Bulletin DS 801-030.

These specifications are subject to change without notice.

American Science & Engineering, Inc.
955 Massachusetts Avenue
Cambridge, MA 02139
617-868-1600

AS&E®

Printed in U.S.A.

Whole-Body CT Scanners

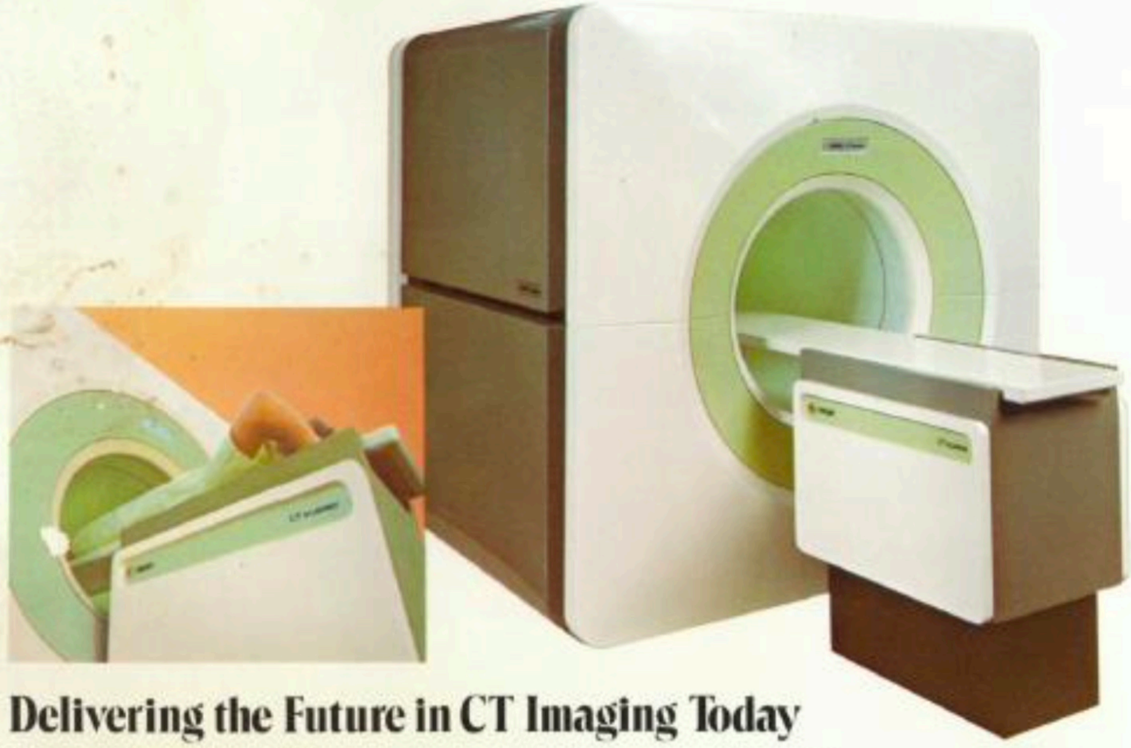

Delivering the Future in CT Imaging Today

Varian's Whole-Body CT scanner, now in production, is engineered for tomorrow's state of the art in CT imaging. Anticipating the future, Varian has incorporated three important design features: continuous, non-stop gantry rotation; an extremely powerful Varian V-76 computer system; and an extra large patient aperture. These features plus the superior head and body images already achieved on the Varian CT Scanner, project it far beyond the dimensions of current CT technology.

Design Features of the Future

6-Second Scan. The Varian CT Scanner collects over 108,000 data points through 360° in six seconds. It uses a precisely collimated fan beam detected by a Varian-designed linear array of 301 optimized Xenon-Krypton detectors.

Continuous Rotation via Slip Ring. The slip ring construction allows simple, trouble-free continuous gantry rotation for single or multiple rotation examinations or, as a standard feature of the Varian unit, triggered physiological gating of data collection.

Customized Scanning. By virtue of the power and speed of the Varian V-76 computer, the operator can easily adjust pixel size, field size, algorithm parameters or other major system parameters at the control console to achieve optimum displays for a variety of clinical situations. Optimization can be carried out virtually simultaneously with data collection and reconstruction of images.

90 cm (36") Diameter Patient Aperture. The extra large patient aperture gives unique flexibility in patient positioning, and use of clinical accessories during scanning.

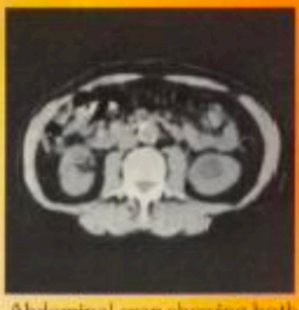

Abdominal scan showing both kidneys and extensive gas in the bowels. Calcification has occurred around the aorta and in a small region of the intestines.

varian

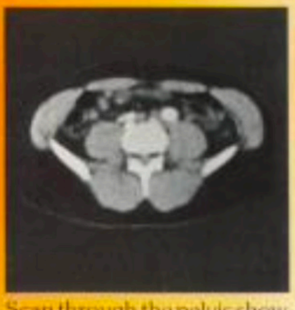

Scan through the pelvis showing bowel gas (black) and contrast agent (white) from a lymphangiography.

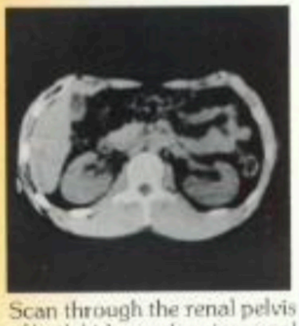

Scan through the renal pelvis of both kidneys showing renal vasculature.

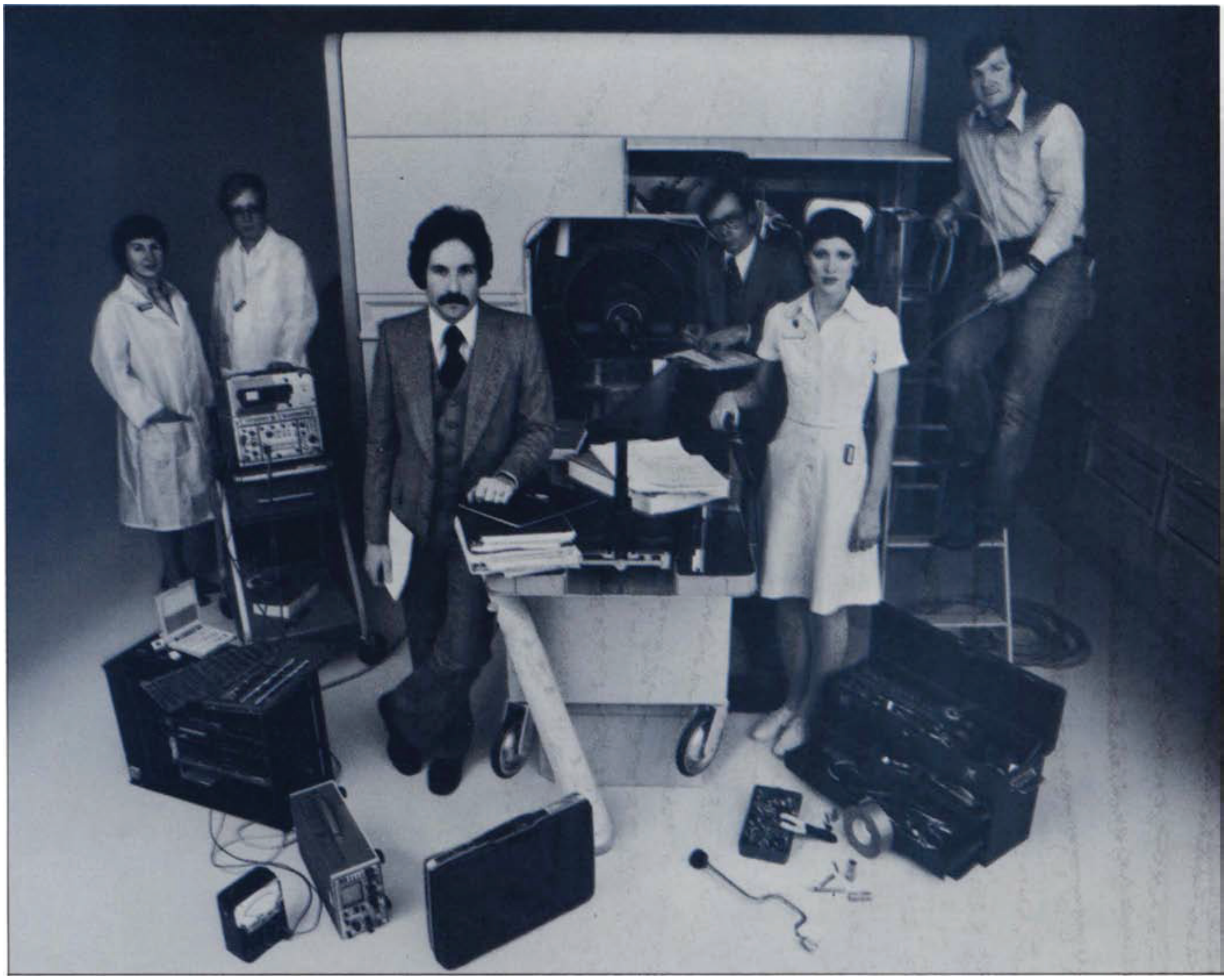

When you're making this kind of commitment, you should know what kind of commitment the manufacturer is making.

Is making...not *will* make. That's the difference between a commitment and a claim. And that's why Pfizer encourages questions about our CT scanner. We think that when it's time to compare answers, you'll see the difference for yourself.

Prestigious installations.
Pfizer has over 150 working CT sites in the United States and 13 foreign countries. Our list includes installations at University of Colorado Medical Center, Columbia-Presbyterian Medical Center Hospital, Johns Hopkins Hospital, Michigan State University Hospital, University of Minnesota Hospital, and Yale-New Haven Hospital.

Near-perfect service.
A worldwide network of dedicated, experienced service engineers, each capable of total system maintenance. They've provided a documented average uptime record of 96% for installations in the U.S. And that's a remarkable service record in the CT industry.

Complete product line.
A full family of scanning equipment. From the industry's first total-body scanner to our benchmark 0200FS whole-body unit, up to the recently announced Pfizer/AS&E CT scanner — the first high resolution fourth-generation scanner. Pfizer now offers one of the most complete lines of CT scanning equipment in the world. Featuring CT images of outstanding clarity, mobile patient handling systems, diagnostic flexibility and advanced software capability every step of the way.

Pfizer and AS&E.® Together.
Early this year, Pfizer Medical Systems, Inc. signed an agreement with American Science and Engineering, Inc. Pfizer and AS&E, both pioneers in the industry, will continue to develop new radiological products, including a revolutionary cardiac scanner. It was Pfizer who developed the first whole-body scanning system. It was AS&E who first introduced fourth-generation geometry, acknowledged as the most advanced CT technology in the world. And together we will continue to practice our commitment. Not just promise it.

For more information, write
Pfizer Medical
Systems, Inc.,
Dept. RA09, 9052
Old Annapolis
Road, Columbia,
Maryland 21045.

Pfizer
PFIZER MEDICAL SYSTEMS, INC.

We're leaders. And we can show you why.

One-second scanning that's second to none.

The Synerview® 300/600 is the only CT system presently available that can routinely perform high-quality scans in 1 second. It's our response to the timely need of the medical community for fast, accurate diagnostic information.

And that's perfect timing.

Synerview combines fast scan and image-processing speeds with rapid patient-data entry. Scanning selection protocol and large access disk storage also facilitate maximum patient throughput.

Patient handling. With Synerview, patient handling originates with the mobile patient transport which allows transfer of patients from anywhere in the hospital. This means you can prepare your patients for scanning in a location remote from the scanning room to prevent interference with scanning in progress. The unique table features a floating top that enables rapid patient positioning.

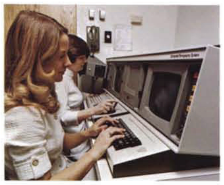

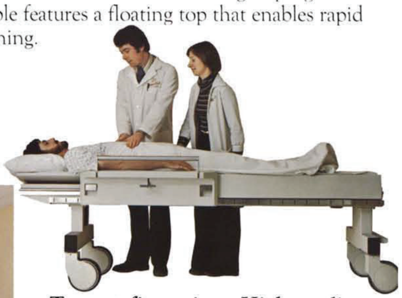

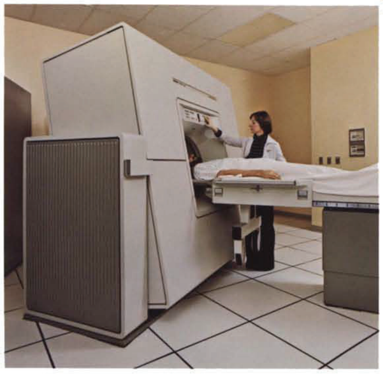

Two configurations. High-quality components. All components are designed to assure years of dependable performance. Detectors use time-proven solid-state devices requiring no special warranty. Modular options can be added to the Synerview 300/600 to meet your individual operating and financial needs. These options include remote diagnostic station, multiformat camera and additional patient-transport tables.

Courtesy of Carlisle Hospital, Carlisle, PA; and Wishard Memorial Hospital, Indiana University Medical Center, Indianapolis, IN.

Synerview exclusives. We offer features that are not found in any other CT system, including three different scanning-mode selections. A choice of Automatic-Rapid, Automatic-Normal, and Manual mode selections means versatility of performance ranging from CT angiography to routine head and body scanning. And, all major CT functions are readily performed, including sagittal and coronal reconstruction, and calcium correction.

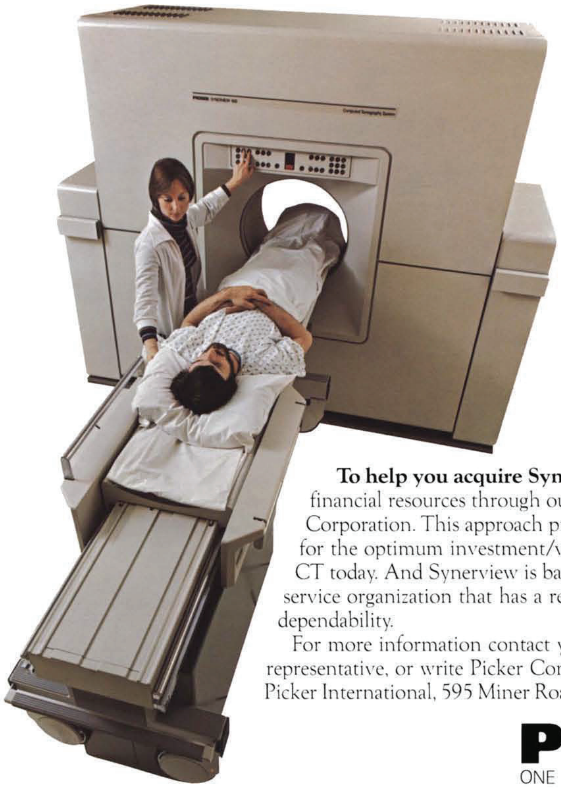

To help you acquire Synerview, we offer our financial resources through our parent CIT Financial Corporation. This approach provides the opportunity for the optimum investment/value ratio available in CT today. And Synerview is backed by our worldwide service organization that has a reputation for proven dependability.

For more information contact your local Picker representative, or write Picker Corporation or Picker International, 595 Miner Road, Cleveland, OH 44143.

PICKER®
ONE OF THE CIT COMPANIES

SIEMENS

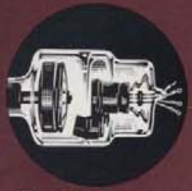

The custom application OPTI-150 CT X-ray tube permits continuous patient scanning at a rate of up to 125 scans/hour.

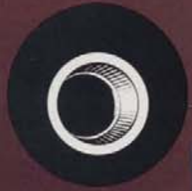

Wide gantry aperture and scan field (both 54 cm. in diameter) facilitate patient handling and positioning.

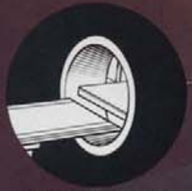

Exclusive dual-section, flat-top table system with motorized patient conveyor.

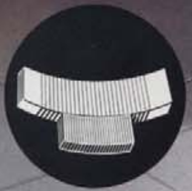

SCINTILLARC-DUAL detector system provides maximized detector utilization with optimized clinical results.

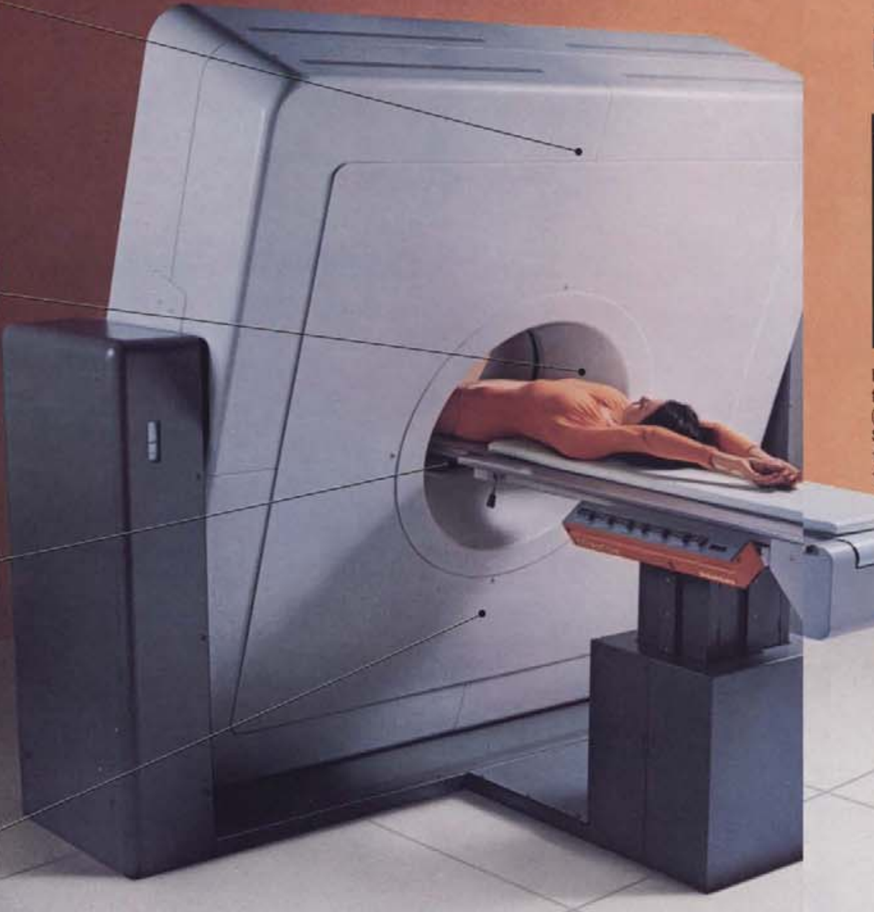

The only CT scanners with instantaneous image reconstruction

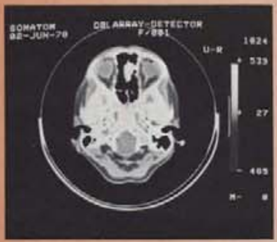

Instantaneous image reconstruction makes the SOMATOM (whole body scanner) and the SIRETOM (head/neck scanner) the most sophisticated computed tomographic systems available today. Upon completion of each scan, the image is immediately available for clinical evaluation. The SOMATOM offers selectable scan times of four and eight seconds while the SIRETOM scan time options are 60, 120, and 180 seconds.

The EVALUSKOP, an independent evaluation console, extends the capability for CT image manipulation and analysis. Programmable and available at the push of a button are window variation, multiple region of interest selection, distance and angle measurement, histogram, magnification, saggital/coronal reconstruction, multiple tomogram displays, tomogram addition and subtraction, and filter manipulation.

Siemens' full line of computed tomographic equipment is designed to fulfill the exacting requirements of CT application. Behind each system definition—X-ray tubes, tables, generators, radiation measurement, and signal processing—is more than 100 years of engineering excellence.

Siemens' world-wide reputation for product support and service is unsurpassed. In the United States alone, Siemens has a technical staff of over 500 trained professionals ready to serve you—should the need arise.

For further information

Please contact your local Siemens Representative or Siemens Corporation, Medical Systems Division, 186 Wood Avenue South, Iselin, New Jersey (201) 494-1000.
In Canada: Siemens Canada Limited, P.O. Box 7300, Pointe Claire 730-P.Q.

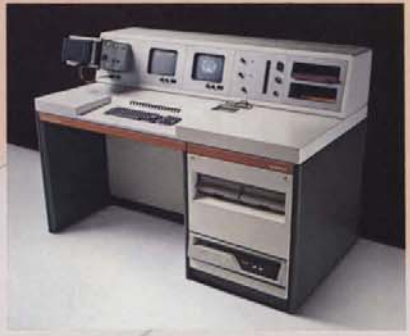

Concepts... engineered to radiologic excellence

EMI scanner 7070

The CT Scanner

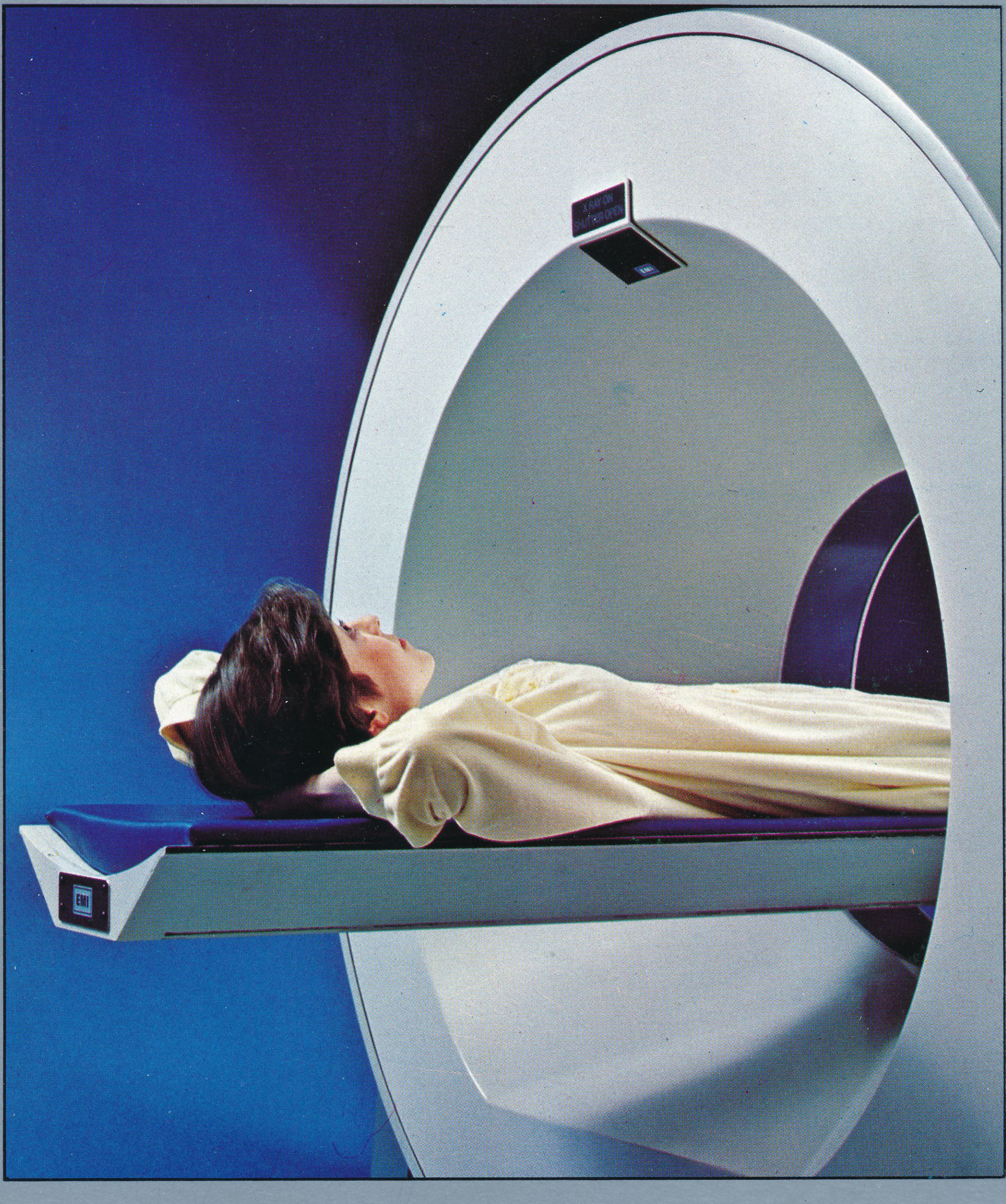

EMI Medical

EMIscanner 7070

The CT Scanner

The scanning system that opens new horizons in CT diagnosis

- Diagnostically excellent scans in three seconds.
- Uncompromised picture quality.
- Nutating Sensor Geometry gives 1½ million readings per scan.
- High dose efficiency.
- Unparalleled diagnostic scope.
- Total diagnostic parameter control.
- Compact, discreet gantry design with generous aperture.
- Simple and accurate patient positioning.
- Elegantly simple console.

The key to the 7070 diagnostic break-through is EMI Medical's revolutionary Nutating Sensor Geometry — an elegantly simple engineering solution to a problem as old as CT technology. The EMI-Scanner 7070 overcomes the traditional incompatability between high scan speed and high picture quality.

The EMI-Scanner 7070 represents the epitome of 7000 Series development.

The 'Master Scanner' of a new generation of CT systems offering a total spectrum of choice; the culmination of eleven years in CT development, proven in a thousand systems. This continuing development leads to the phased introduction of further advanced technology. For example direct use of CT information in therapy planning with the new EMIPLAN 7000 RTP Radiotherapy Planning System.

Fast scans with uncompromised resolution

Reduced scan time has traditionally resulted in a reduction of spatial resolution. The problem, however, was basically the engineering limitations of existing scanner geometry. Our engineering ingenuity has solved it.

Nutating Sensor Geometry, an EMI-Scanner 7000 Series 'first', produces low noise images with excellent spatial and density resolution from the 1½ million readings collected in a three second scan. Even with this fast scan speed, EMI Medical's reputation for high picture quality is fully maintained.

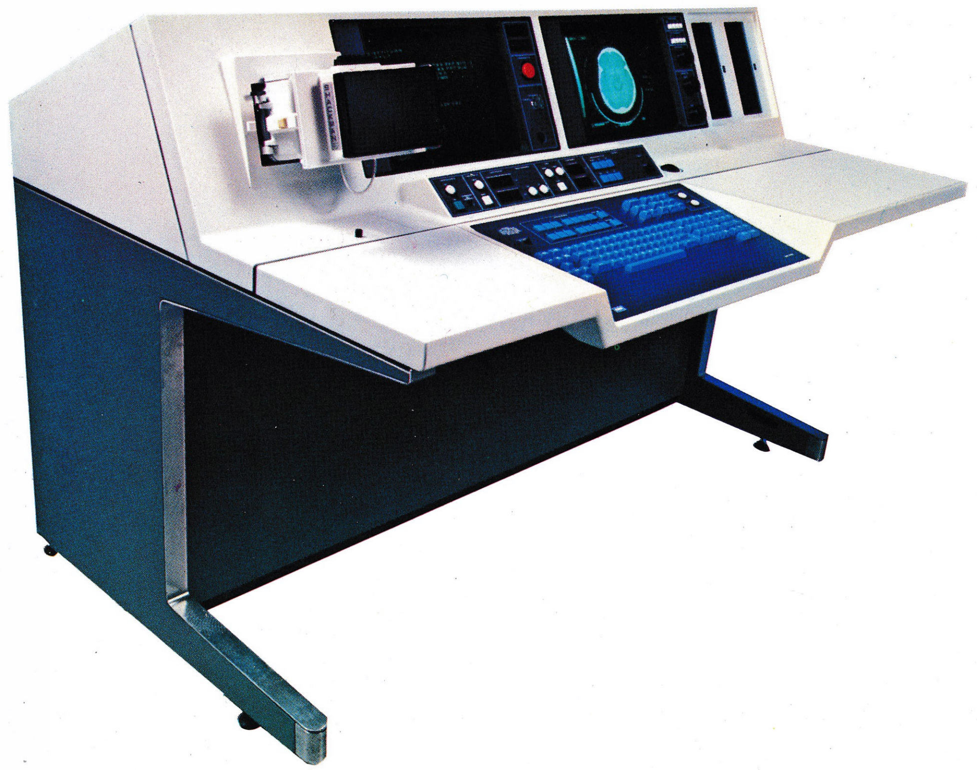

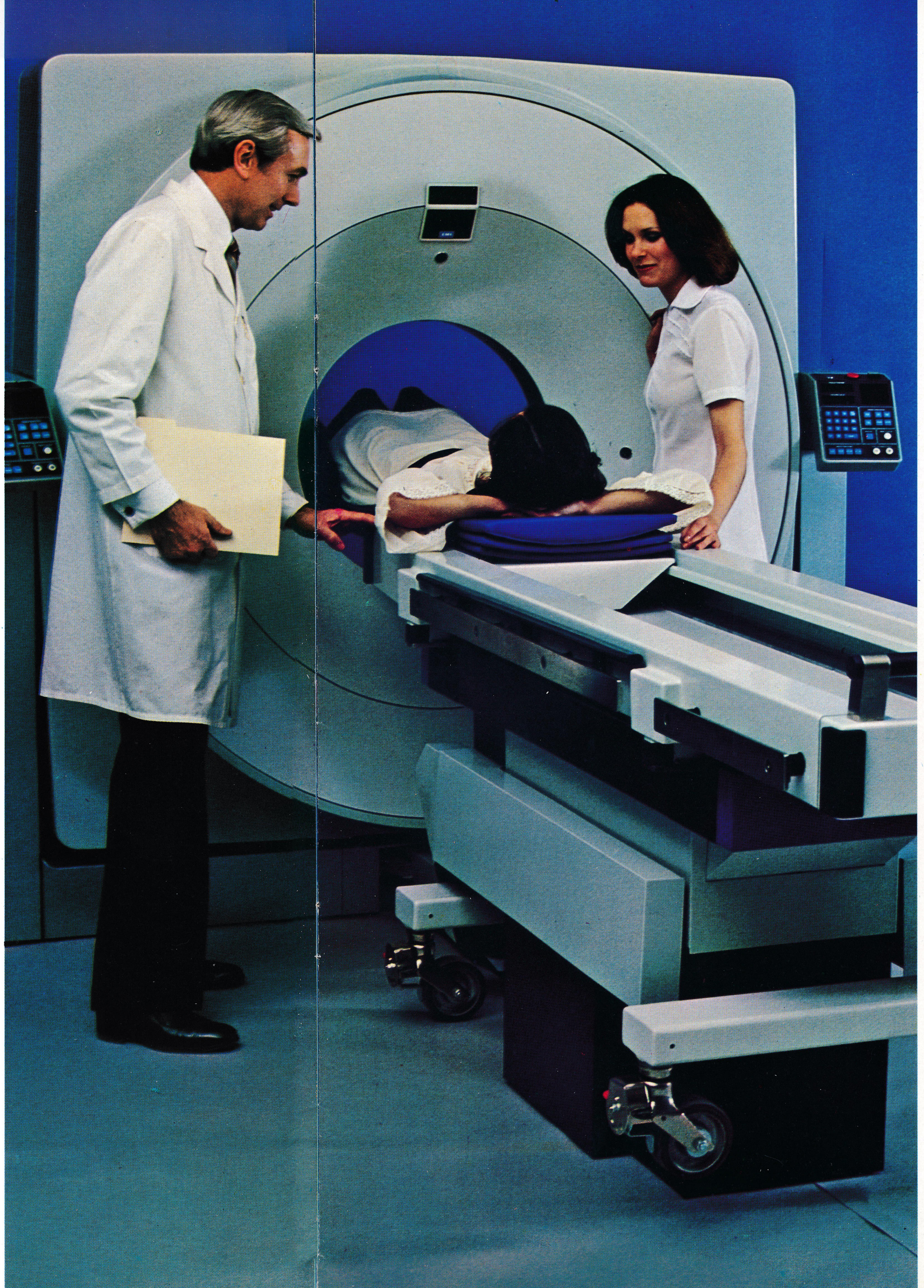

Optimum dose levels; highest diagnostic power

Maximisation of photon detection is the reward of Nutating Sensing Geometry and of a new breed of integrated solid-state sensors. Clearly, the greater the photon detection efficiency, the lower the dose needed to achieve a diagnostically effective image. EMI-Scanner 7070 produces advances towards ideal dosage efficiency.

Elegant technology; thoughtful design

It is significant that a crucial breakthrough in scanning principles should spearhead a total CT systems design reform. It is reflected in the appearance, size and operational logic of the 7070.

With its compact unobtrusive design and low lines, its generous 60cm aperture and its quiet operation, the 7070 gantry encourages patient co-operation and promotes confidence.

In fact, the whole procedure of 7070 operation becomes one of calm, precise routine. Gantry controls are duplicated left and right for operator convenience. The scanning table is ingeniously designed as a mobile, lock-on module which can also be the patient preparation trolley. Flexibility of table adjustment including slew and gantry tilt, permits simple and precise, patient positioning and minimum patient handling.

Elegantly simple operator controls

Maximum patient comfort is matched by total operational ergonomics. The console controls are deployed for simplicity of use and incorporate preset function buttons which implement complete predetermined sequences. In fact up to eight entire scan procedures can be pre-programmed to enhance patient throughput.

The two large high-definition alpha-numeric and image displays are augmented by the dedicated photographic monitor.

Comprehensive archive facilities are provided on disc or dual floppy disc and optional magnetic tape, enabling easy storage, transfer and retrieval of scans.

New horizons in diagnostic application – and control

The unprecedented combination of scan speed and superlative image quality opens up new areas of CT application. On the 7070 this new diagnostic scope may be fully exploited by hitherto unknown freedom of diagnostic parameter control.

Angled scans which could once not have been contemplated for certain patients, may now be simply undertaken by a combination of gantry tilt (+10° to −30°) and patient table slew (±20°).

The operator is free to select any one of **five scan times** from 3 to 30 seconds and **five scan fields** from 120mm to 500mm.

Slice thickness is controllable too, between 2mm and 15mm, while other facilities include **statistical calculation** over a **region of interest** with **magnification** and comprehensive **picture manipulation.**

More control, finer control, simpler control, faster control – of the most advanced CT system yet devised.

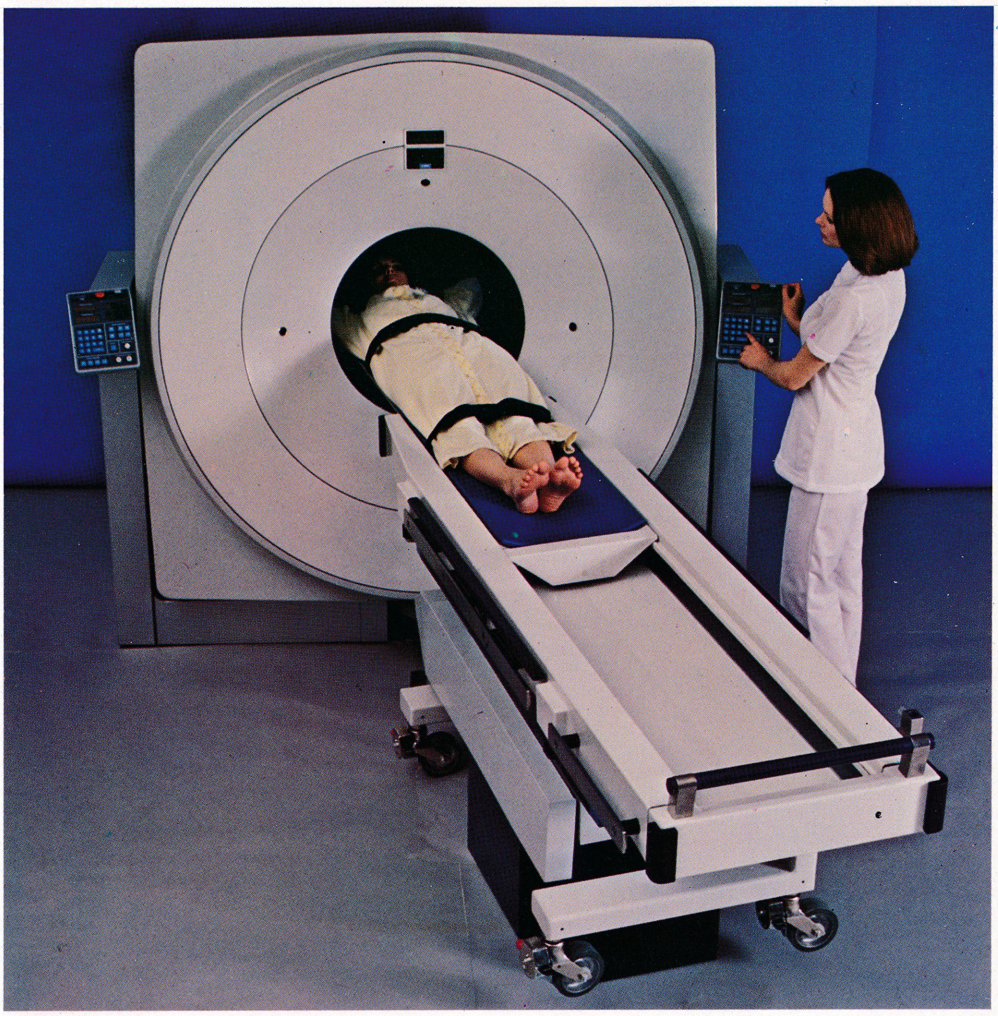

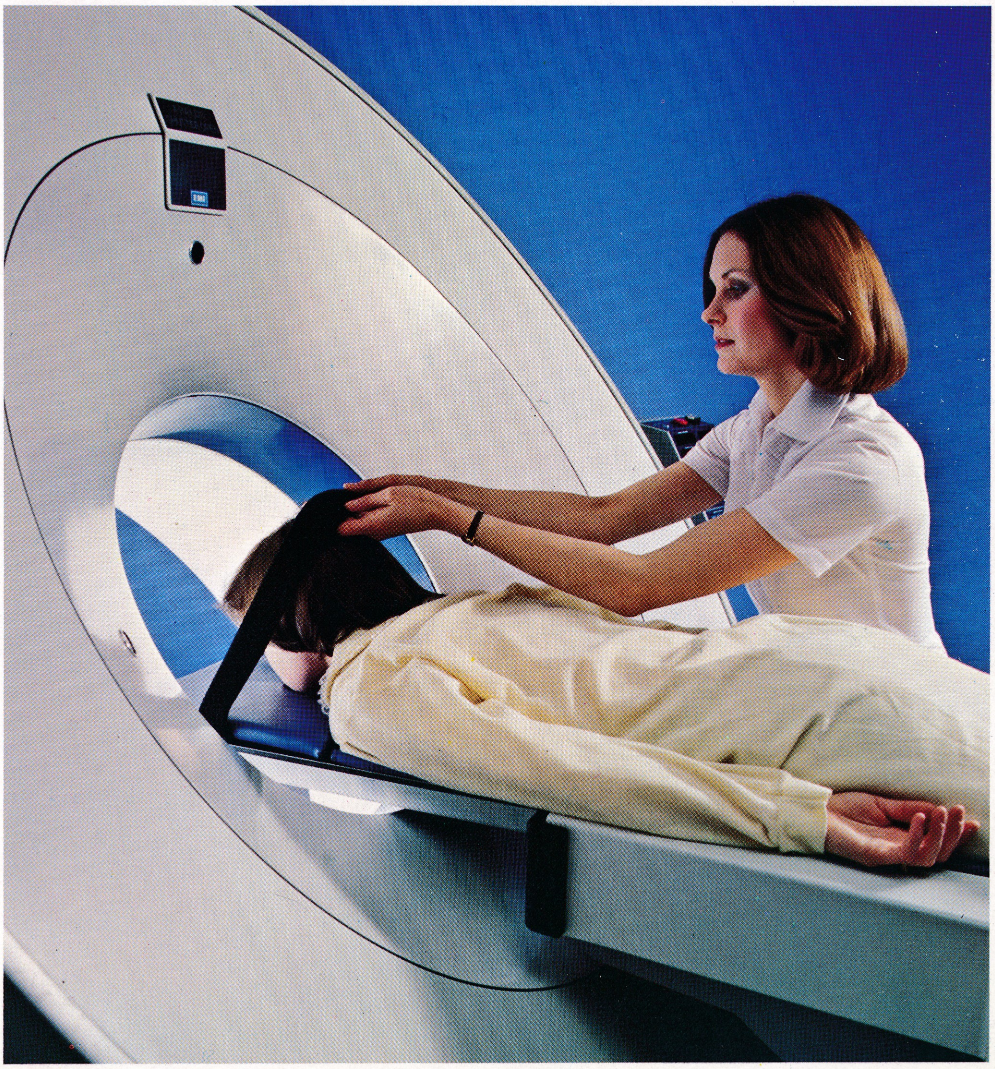

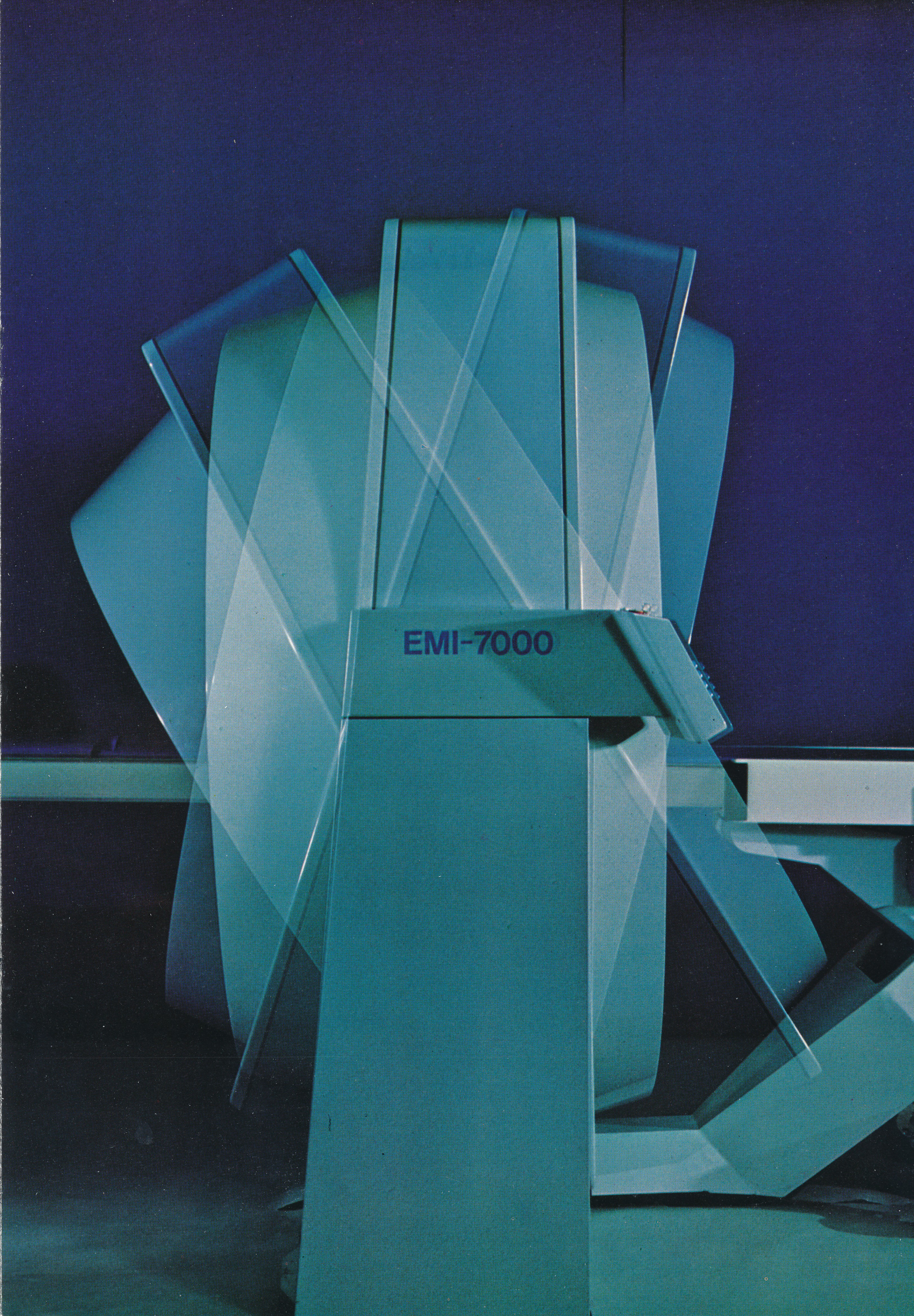
EMI-7000

The Key to the EMI-Scanner 7070 concept

The philosophy, like the design concept of **Nutating Sensor Geometry,** is simple but has no parallel.

It is this: for every combination of scan, speed and dose parameters you select, the EMI-Scanner 7070 will produce the maximum diagnostic information.

Two advances make this possible.

The first is an electronic one. The availability of Caesium Iodide solid state sensors used in conjunction with photodiodes provides photon conversion efficiency markedly better than conventional photomultiplier tubes. Every sensor converts more photons into diagnostic data. Which brings us to the second advance – an engineering one.

Nutating Sensor Geometry

The patient is placed within an unbroken, non-rotating ring of detectors, while the X-ray source rotates around the outside of this ring. As the source rotates, that section of the detector array nearest to it is displaced slightly to allow the beam to pass directly through the patient to the detectors diametrically opposite.

This simple solution means the patient is considerably closer to the detector array than to the source – a relationship which minimises penumbra. Because penumbra is now limited the detectors can obviously be more closely spaced – so closely that 'dead space' between detectors is virtually eliminated in the tightly packed ring of over 1000 detectors.

Major EMI-Scanner 7070 options

Magnetic Tape Unit for long term archival storage of data.

Line Printer for print-out of scan data and directories.

Diagnostic Console provides the same image display facilities as the Operator Console and allows on-line viewing of current scans with simultaneous diagnostic evaluation of previous scans, and provides control of window parameters, ROI and sagittal and coronal reconstruction.

Additional patient trolley. The transportable scanning table can serve as patient preparation trolley. The addition of a second trolley allows simultaneous scanning and preparation, invaluable in fully loaded systems for maximum patient throughput.

Multi-format Imager. A free-standing, multiple image camera enabling scans to be recorded onto imaging film for processing and viewing as normal X-ray film.

Other options include the **bromide paper camera** and **colour monitor.**

EMI-Scanner 7070 – The CT scanner

Nutating Sensor Geometry
The Essential Benefit

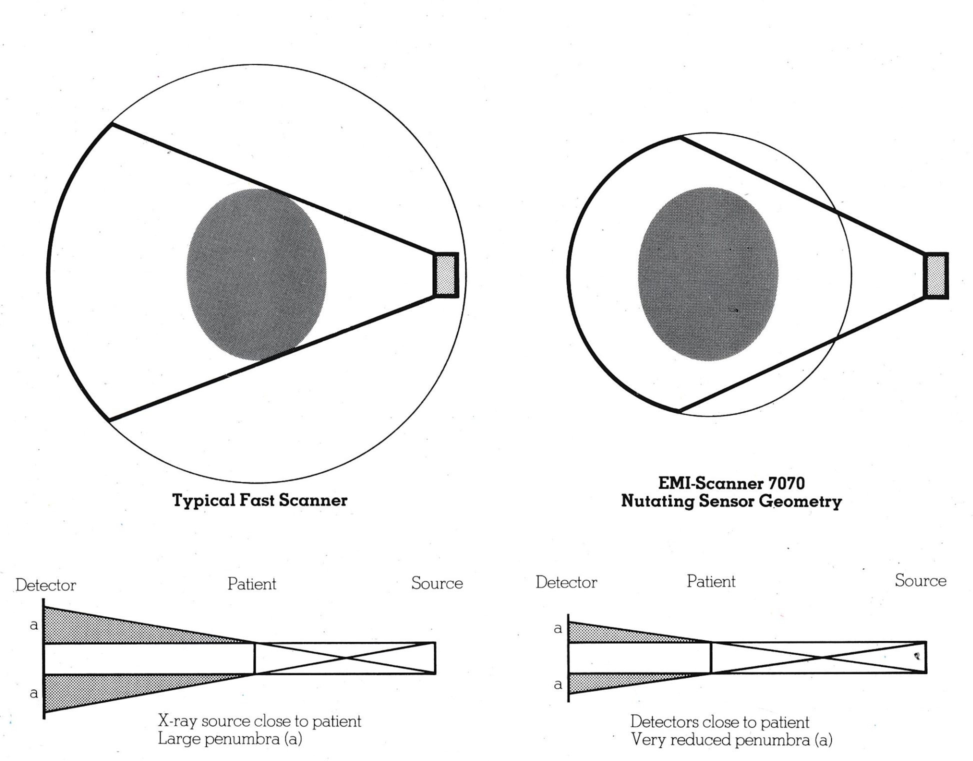

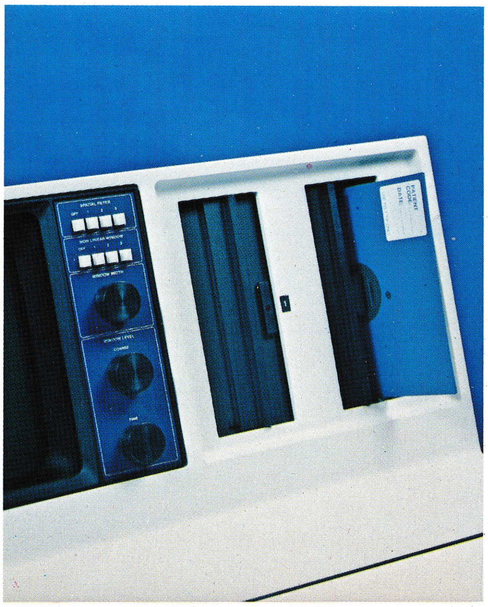

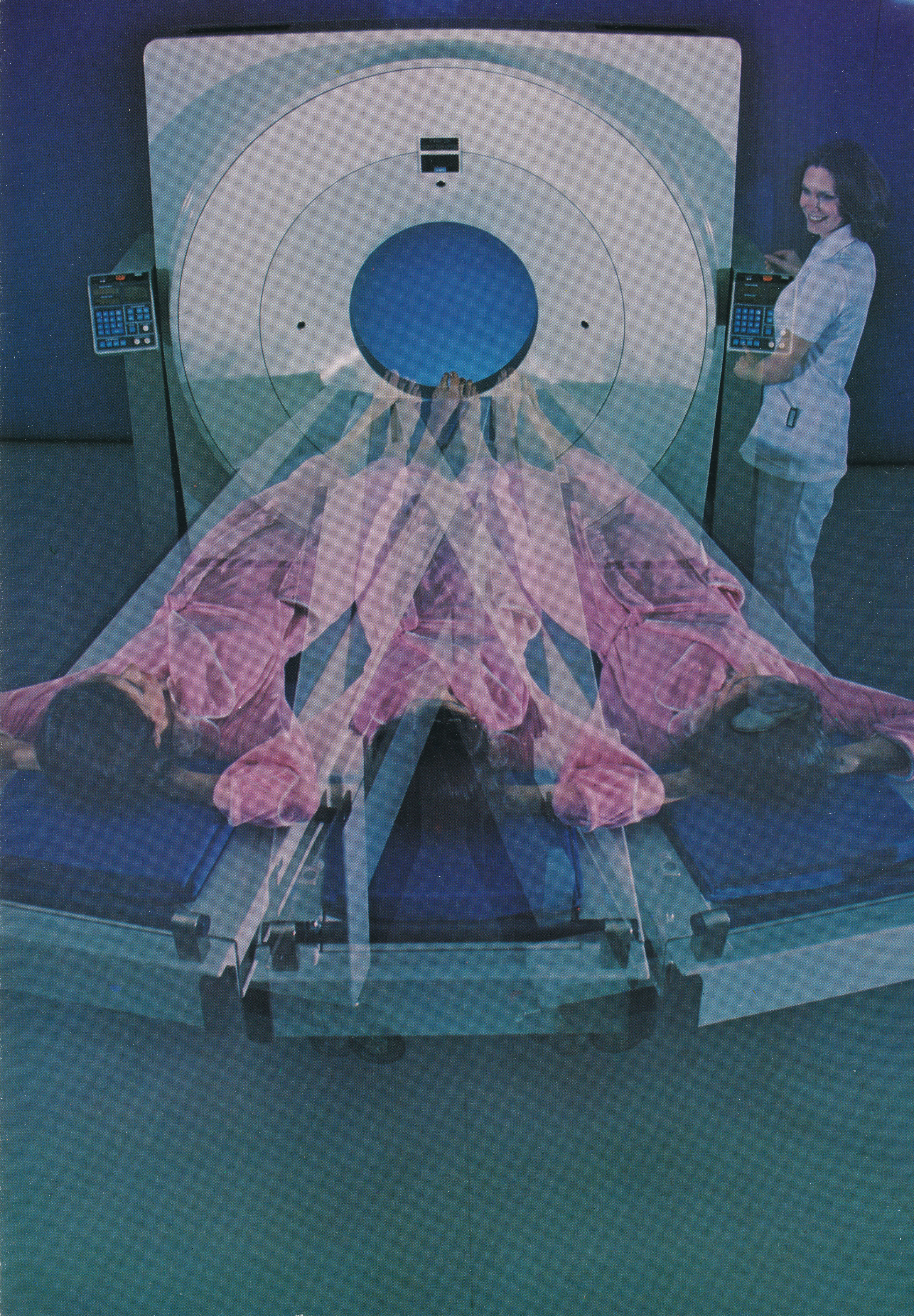

EMI Medical

Head Office
EMI Medical Limited
Jubilee House
120 Blyth Road
Hayes
Middlesex UB3 1DL
England
Telephone: 01-573 7766
Telex: 23215

A member of the EMI Group.
International leaders in music,
electronics and leisure.

EMI Medical reserves the right to
modify or revise the specifications
shown in this brochure.

EMI-Scanner is a
Trade Mark of EMI Limited

©EMI Medical Limited 1978

Printed in England

7070

UM120091/0978

DELTASCAN™ 2020

Two Second Total Body CT System

TECHNICARE

DELTASCAN DIVISION

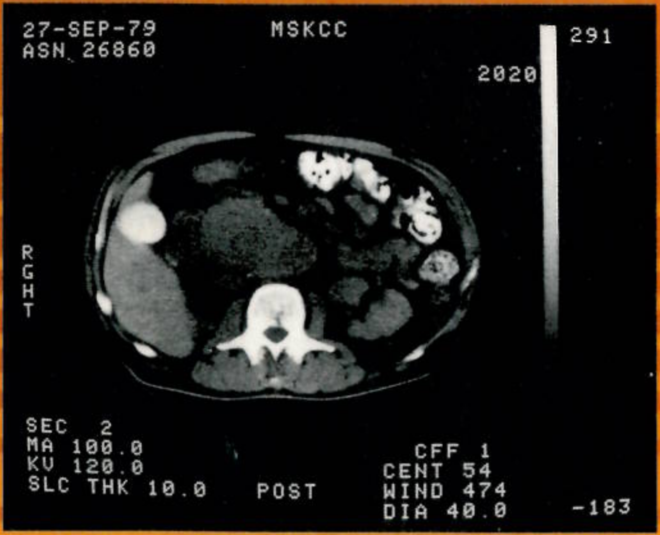

Aneurysm of the abdominal aorta

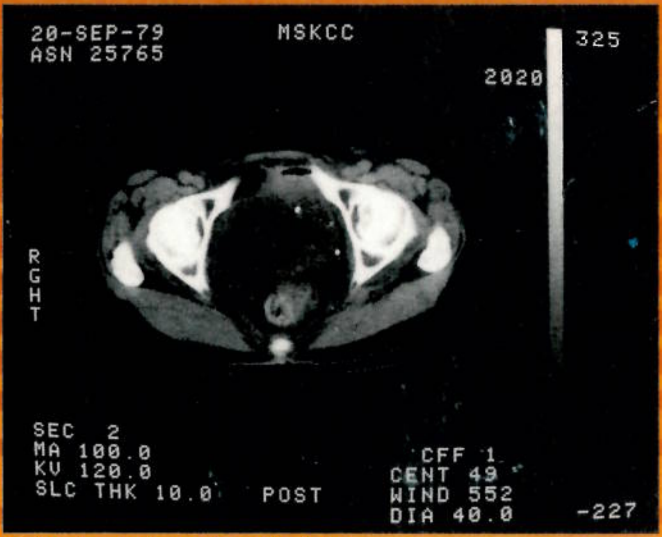

Pelvic tumor invading bladder

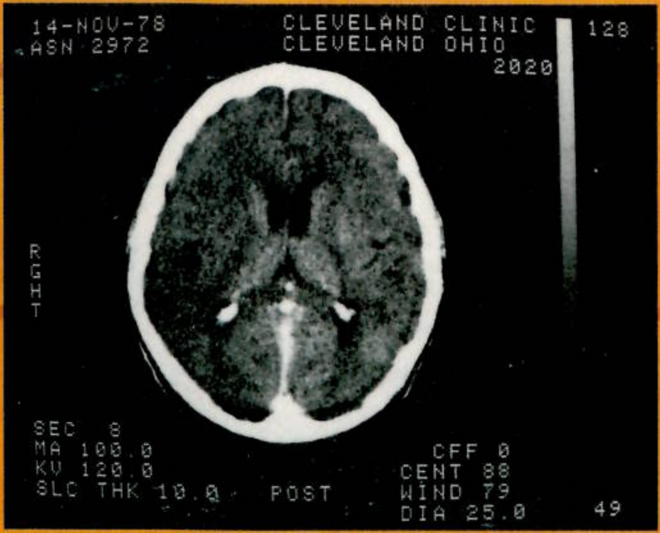

Normal brain showing choroid plexus (with shaped filter collimator)

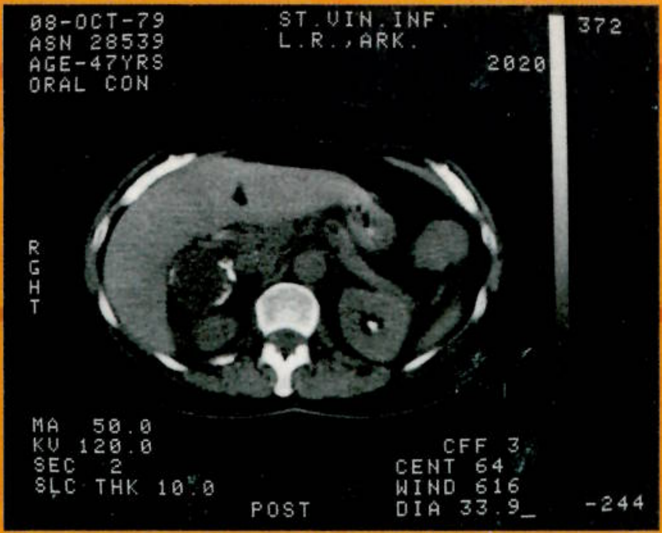

Mass anterior to kidney with rim calcification

DELTASCAN 2020

The Ultimate in CT Flexibility

Meet the Future The Deltascan™ 2020 system is the first CT scanner to go beyond expected capability, setting fourth generation standards. Uncompromised in performance, it is one of the most flexible CT systems available today.

Speed Motion artifacts, a constant problem in slower systems, are virtually eliminated with a scan speed of two seconds. And with a special "Quick Look" feature, reconstruction occurs in less than six seconds. The Deltascan 2020 system is capable of outstanding patient throughput for an advanced CT system—up to 20 patients per eight hour day under optimum conditions.

Flexibility The greatest possible range of operator selectable scan parameters available facilitate total flexibility in dose/resolution decisions. Superb image quality with a 512 matrix, the largest pixel grid of any CT system, plus a shaped filter collimater make the Deltascan 2020 system unmatched in flexibility, uncompromised in image quality.

Control Software manipulations can be performed on both the operator and physician's consoles. While the operator is viewing a "Quick Look" image on his console, the physician can monitor the scan in progress, or interpret a previous image, on his terminal using the 512 matrix. Only with the Deltascan 2020 do you get this kind of designed in control.

More Computer Power The 2020 system shuns obsolescence. With the most sophisticated computer technology in CT, the Deltascan 2020 system has future capability built into the system today. The 2020 system's built-in reserve of computing power is unmatched.

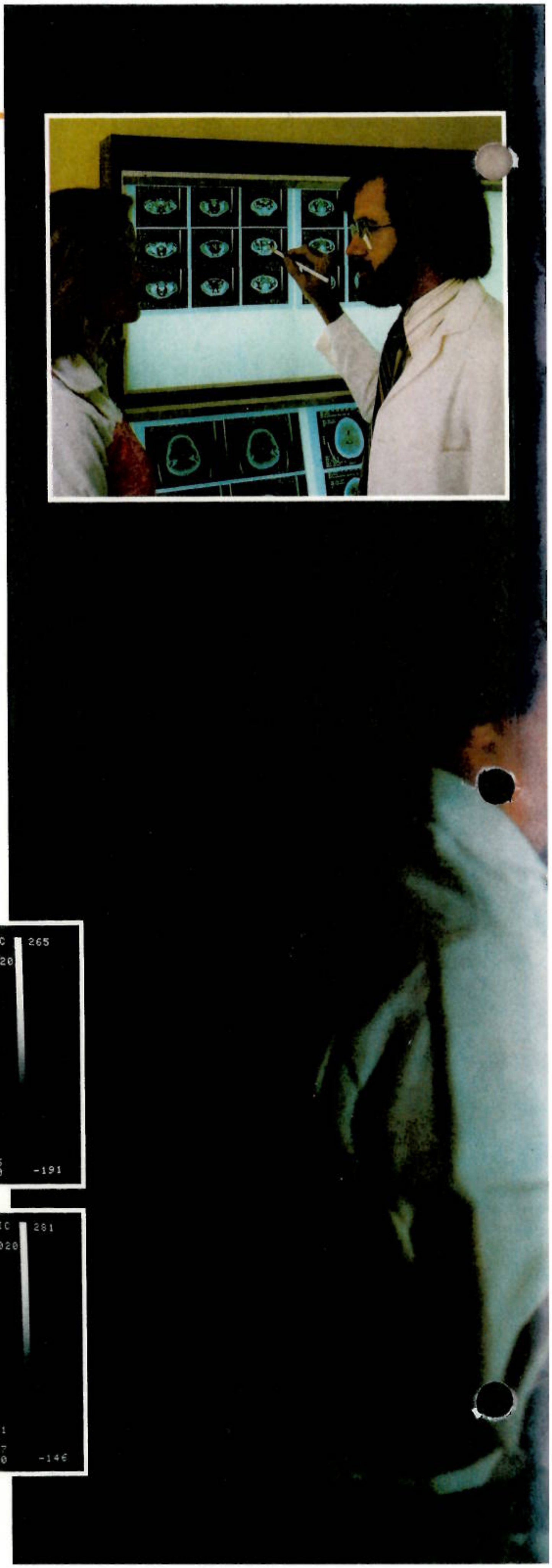

Normal orbits

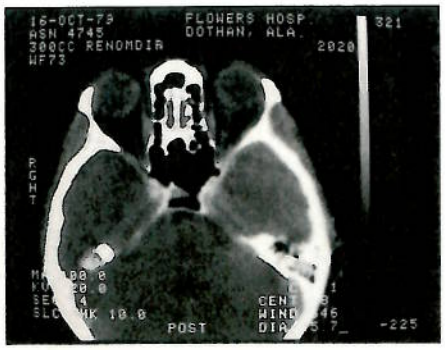

Right renal cyst; mass at tail of pancreas

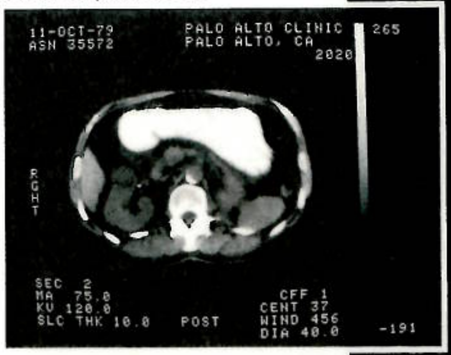

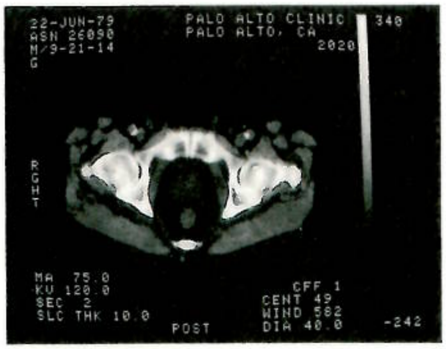

Normal prostate

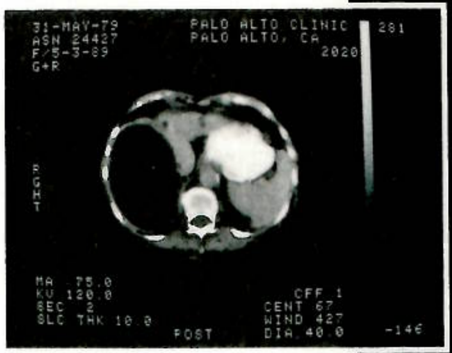

Multiple hepatic cysts

Deltascan™ is a trademark of TECHNICARE Deltascan Division.

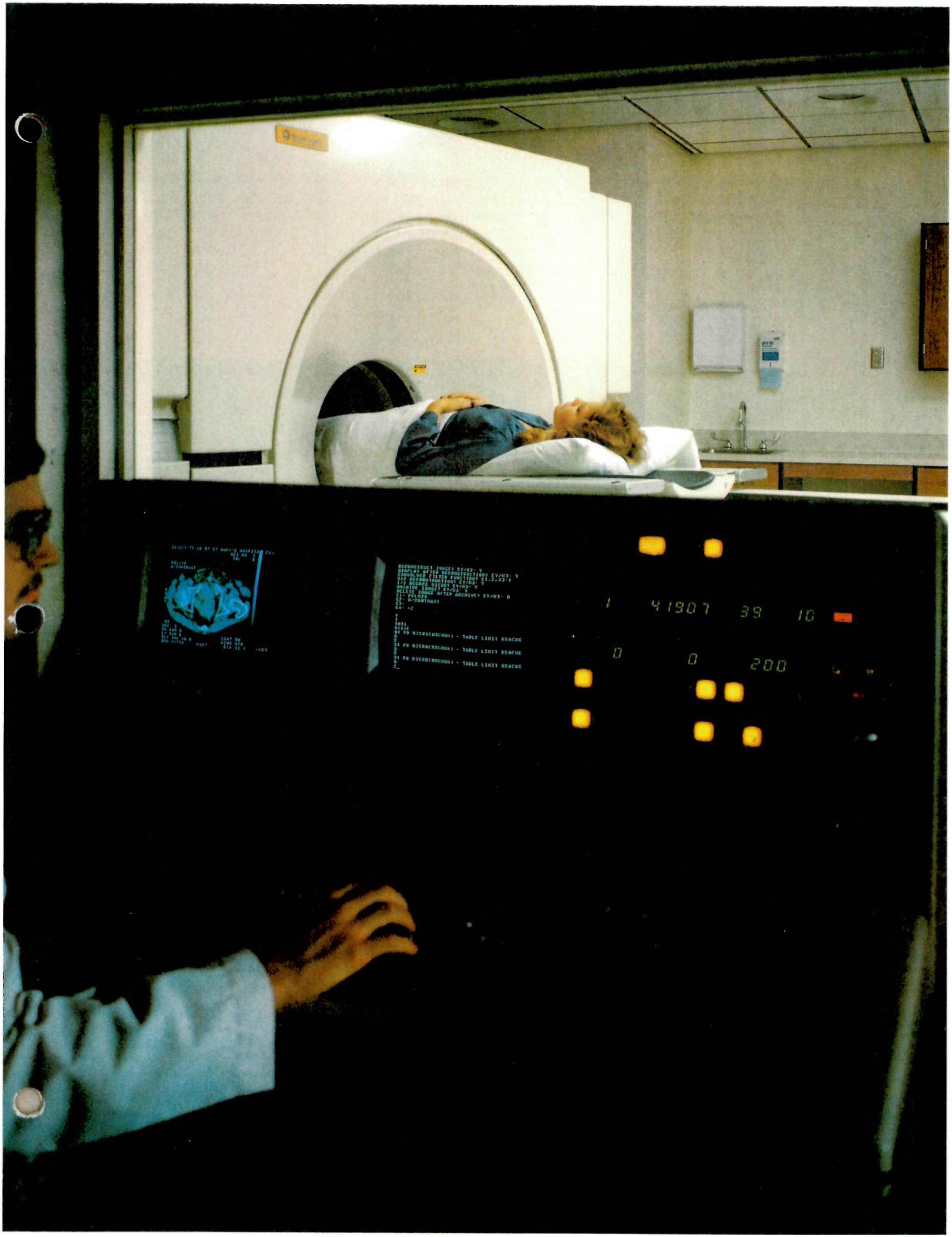

DELTASCAN 2020

A Complete Scan Control and Image Review Center

Operator Control is Optimized
The operator's console on the Deltascan 2020 system is designed for optimum control, ease of operation and technical efficiency. Patient scanning, reconstruction and "Quick Look" image verification can occur at the operator's console while previous images are being evaluated at the physician's terminal using the 512 matrix. Both the operator and physician's consoles deliver complete control of all CT viewing and software manipulation functions.

For hard copy, CT transparencies can be made automatically with the use of the Deltamat™ Multiformat camera.

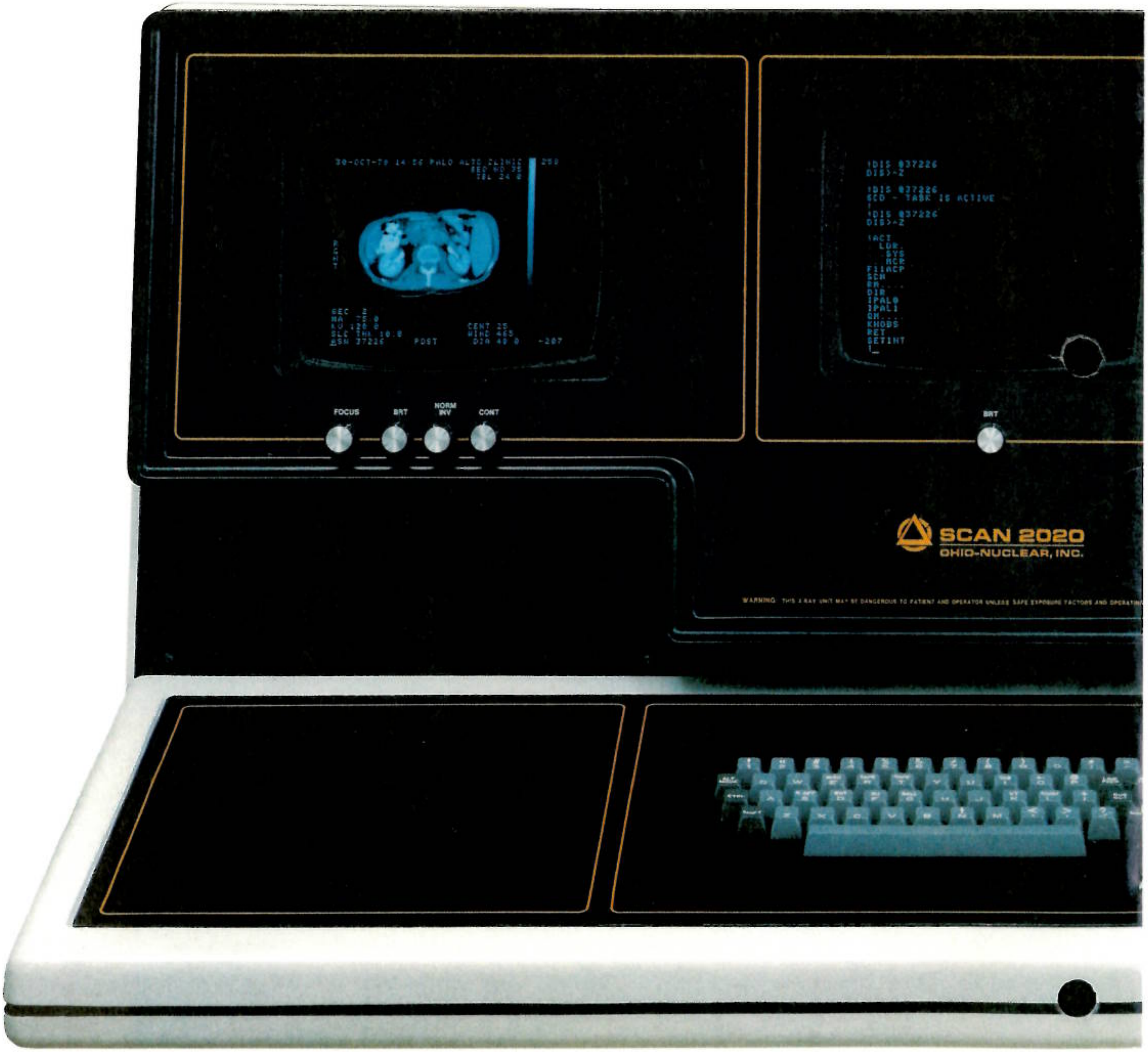

Deltamat™ is a trademark of TECHNICARE Deltascan Division.

Flexibility in Dose/Resolution Control

All technique factors in the Deltascan 2020 system are software controlled. Consequently, dose and image quality are optimized.

In addition, using the Shaped Filter Collimator, at any given mAs, surface dose is reduced by a factor of three; or, for any given skin dose, noise is reduced by a factor of 2.4. Ultra low dose scans, as low as 0.3 rads, can also be performed if required for specialized techniques.

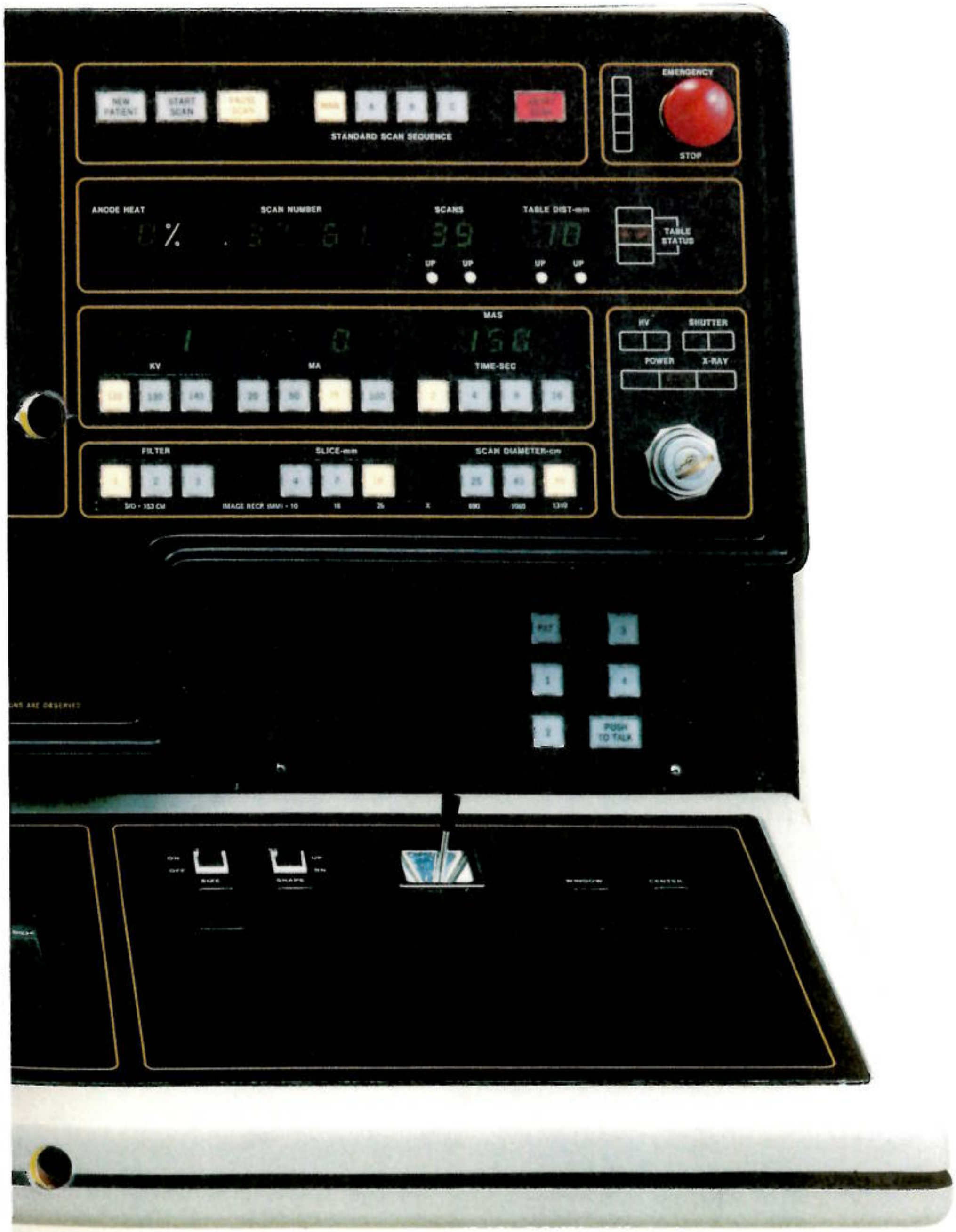

Increased Patient Throughput

Another design feature of the 2020 system allows for either immediate high resolution reconstruction of the slice or deferred batch reconstruction after all the scans have been performed. Using the batch reconstruction mode with "Quick Look," patient throughput can be dramatically increased, as patient transfer can occur during batch reconstruction.

Largest Selection of Scan Parameters

Backlit scanning technique buttons provide the operator with the largest possible selection of scan parameters; 80, 100, 120kVp; 25-100mA; 2, 4, 8, 16 second scan times; 3 filters for source filtration; 2, 4, 7, 10mm slice thicknesses; 12*, 25, 40, 50cm scan circle diameters; and 256 or 512 matrices plus a 128 matrix for "Quick Look" image verification.

Three standard scan sequence buttons which can be pre-programmed to any of the scan parameters provide automatic selection of any combination of the scanning technique buttons. This is flexibility in CT diagnostics; this is complete operator control with optimum patient throughput; this is the Deltascan 2020 system.

*Available in 1980.

DELTASCAN 2020

Precision Scanning

The Deltascan 2020 System— Flexibility built for the future:

- Selectable scan speeds of 2, 4, 8 or 16 seconds with "Quick Look" reconstruction in less than six seconds
- Flexibility in image quality decisions with 20 software convolution filter functions built in
- 512 matrix—the largest pixel grid available for the highest possible resolution
- Shaped filter collimator to reduce surface dose and noise
- The most sophisticated computer technology in CT today
- Exceptional patient throughput capability

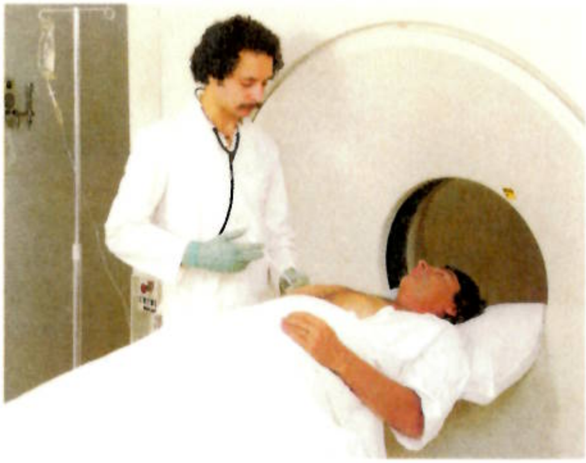

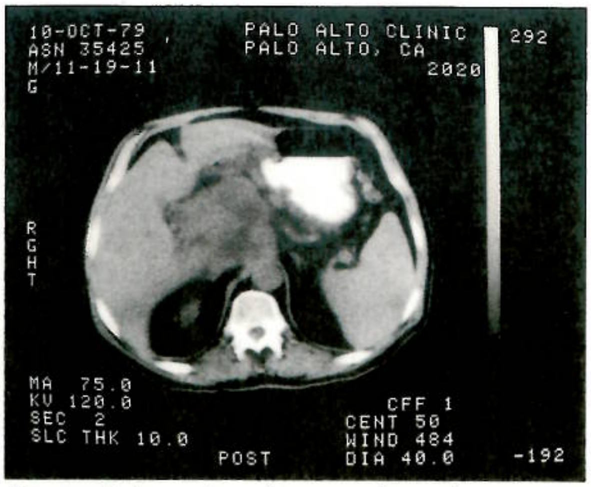

Pancreatic tumor invading porta hepatis

Optimum Patient Handling

A 60cm gantry opening allows virtually any size patient to be scanned. In addition to having the largest available gantry opening, the Deltascan 2020 system also offers the widest selection of fields of view presently available (12* to 50cm scan diameters) and permits effective scanning of 98% of the adult patient population. The gantry tilt of ±20° facilitates direct coronal head scans and scans parallel to spinal column structures. The beveled gantry opening and thin gantry size at the patient, permit easy patient access when administering contrast or during emergency situtations.

Delta Prep™

The Delta Prep patient handling system provides maximum patient comfort and flexibility of use for both in-patients and out-patients. For critically ill or injured patients a mobile unit is used to transfer the patient to the scan room. With the Delta Prep system the technologist's time in the scan room is optimized.

Delta Prep features:

- Mobile patient pallet (contoured to fit the body)
- Automated pedestal
- Flat insert for therapy planning

*Available mid 1980.

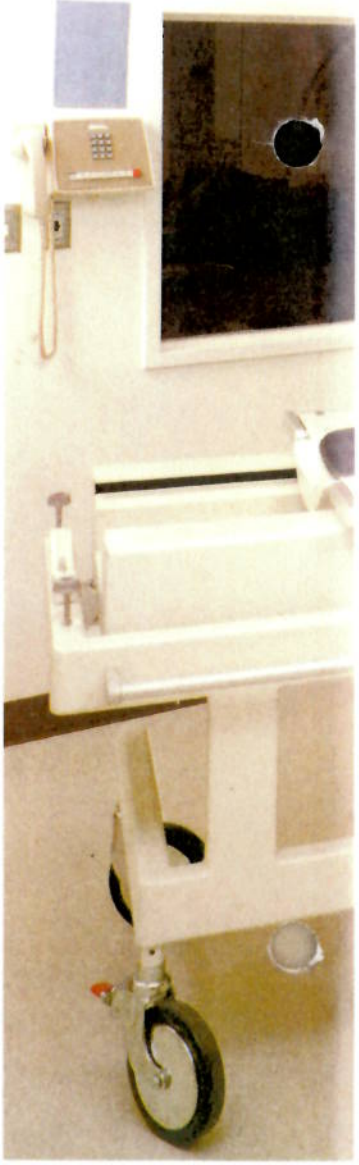

Delta Prep™ is a trademark of TECHNICARE Deltascan Division.

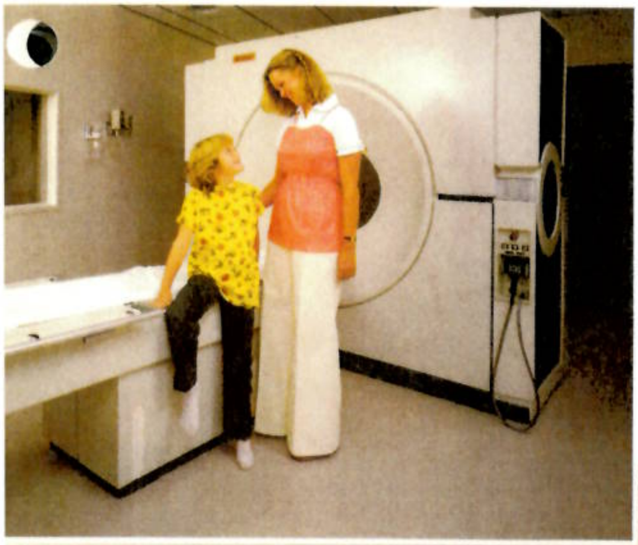

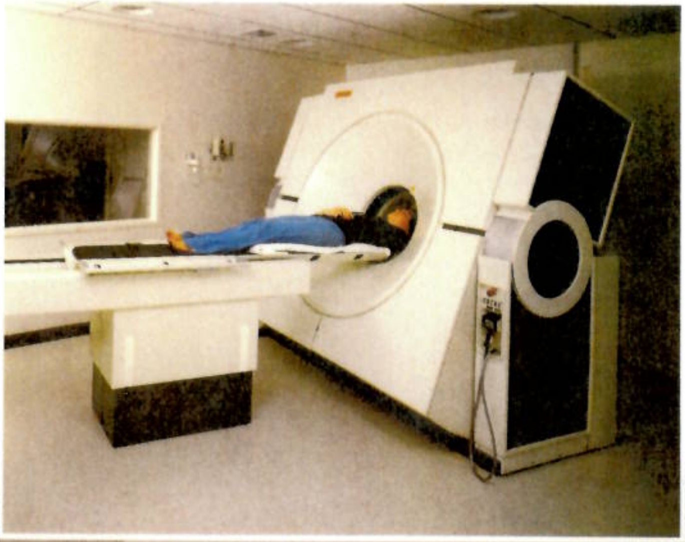

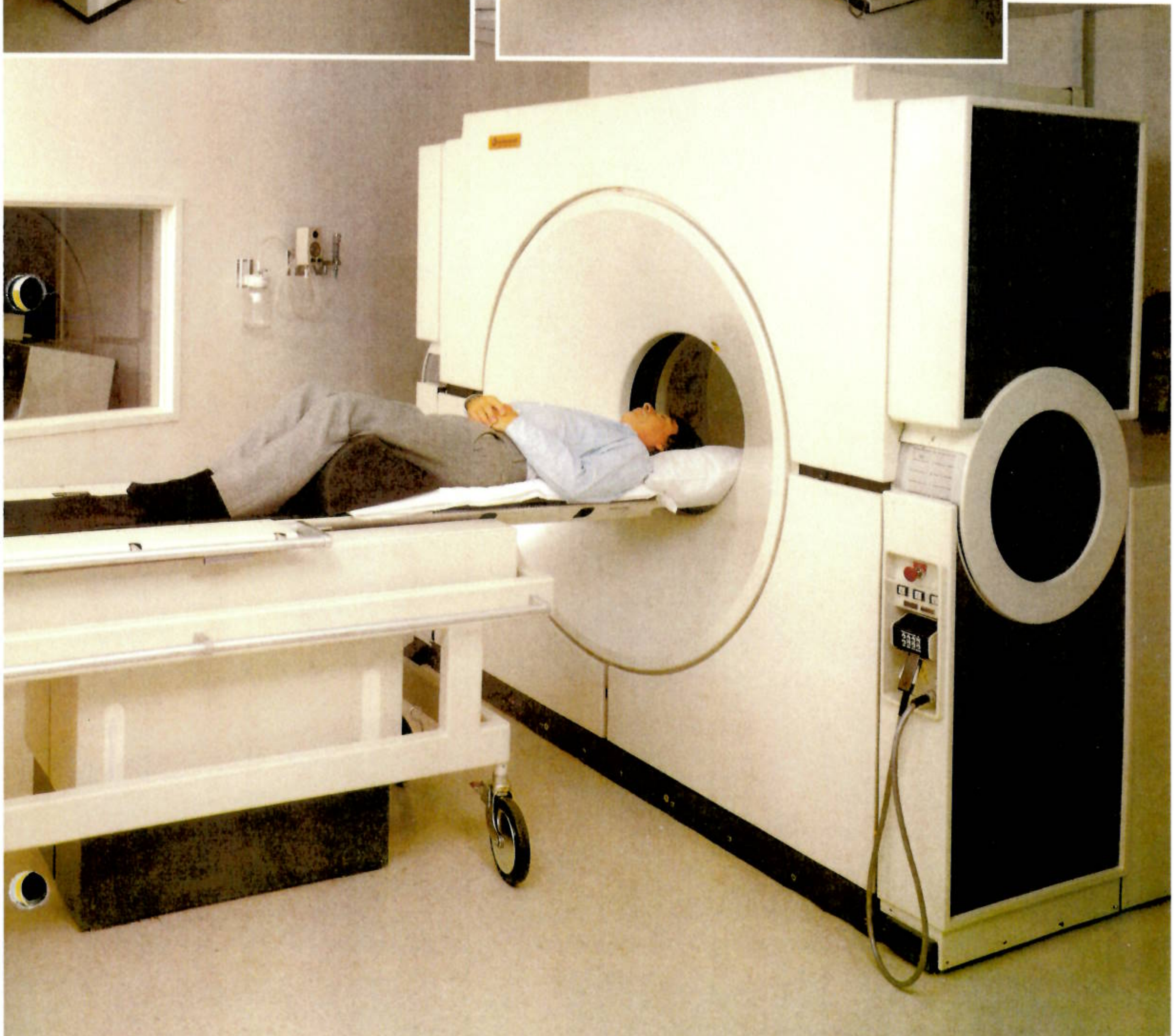

DELTASCAN 2020

A New Dimension in Control— The Physician's Console

No other CT scanner affords the physician the flexibility or the control offered by the Deltascan 2020 system. Using the physician's display terminal, the physician can review previous scans while checking on the scan in progress. When necessary, he can request changes in scan parameters by simply conveying his wishes to the technician at the operator's console—a unique Deltascan feature.

The Deltascan 2020 system offers a large and growing selection of interactive software programs to extend the frontiers of diagnostic capability.

Software Capabilities

- Image Display—128x128, 256x256, or 512x512 matrices
- Flexible Alphanumeric Format Control
- Selectable Gray Scale
- Region Histogram
- Dual Elliptical Region of Interest*
- Image File Selection
- Spatial Reference
- Display Orientation
- Gray Scale Inversion
- Image Expansion
- Density Highlight
- Image Enhancement
- Regular Region of Interest
- Irregular Region of Interest
- Image Review Mode*
- Static Reference*
- Saggital/Coronal Display*
- Multiple Image Format*
- Deltapoint Annotate*

*Available in 1980

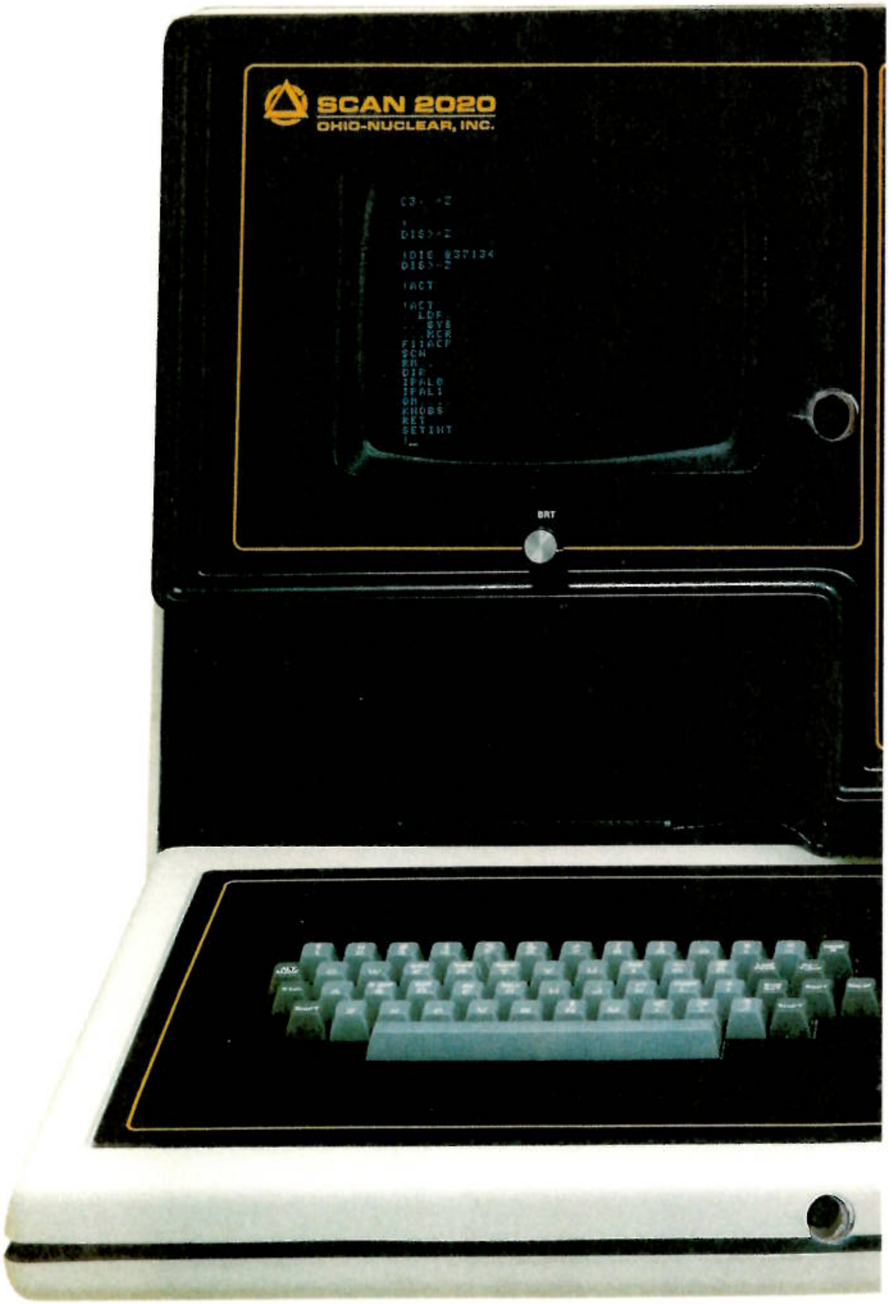

Ease of data entry is enhanced by a modern alphanumeric keyboard and sixteen programmable command push-buttons allow for interactive software function selections. Individual image window and center controls plus region of interest size and shape controls accommodate image display adjustment and analytic program interaction.

Three CRT monitors are located at the physician's display terminal. A 14″ diagonal image display, a 9″ diagonal text display, and a 5″ diagonal image display for photographic use with either the bezel mounted Polaroid® camera or alternative film types.

For greater flexibility in site design and diagnostic privacy, the physician's display terminal can be located up to 500 cabled feet from the control room. A voice communication intercom connects the operator's console, physician's display terminal and the patient in the gantry for complete and constant audio control.

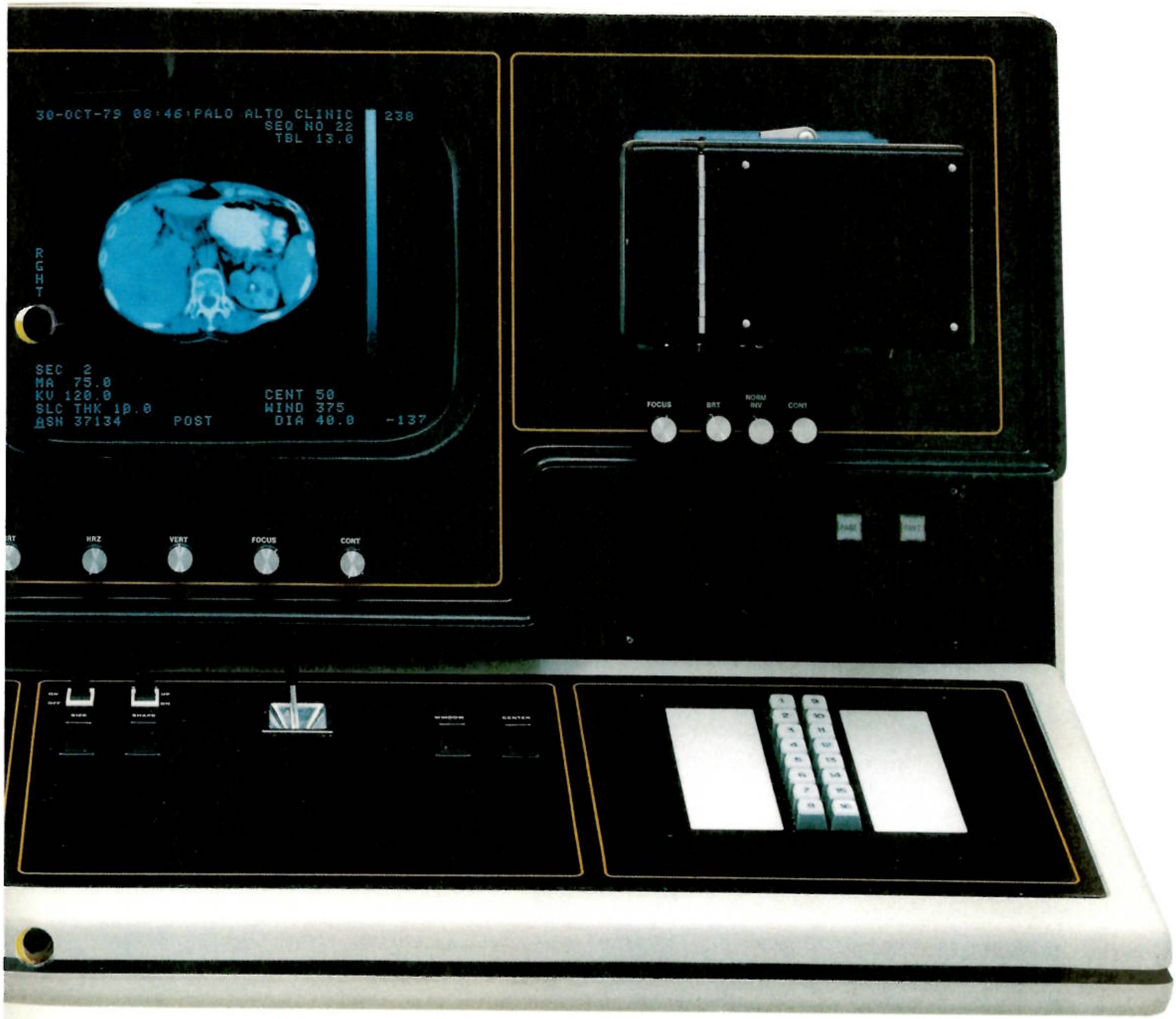

Polaroid® is a registered trademark of Polaroid Corporation.

DELTASCAN 2020

Fourth Generation Technology —A Bridge To The Future

The Deltascan 2020 system is the finest two second total body CT system available today—evolutionary in design and revolutionary in performance and capability. Software developments can be implemented efficiently making the 2020 system an investment for the future. This designed in computer power for future applications precludes obsolescence and provides a diagnostic bridge to the future. Because of its fourth generation technology, your Deltascan 2020 system will allow you to participate in Technicare/Deltascan's ongoing developments, both hardware and software, which will expand the possibilities of CT diagnostics.

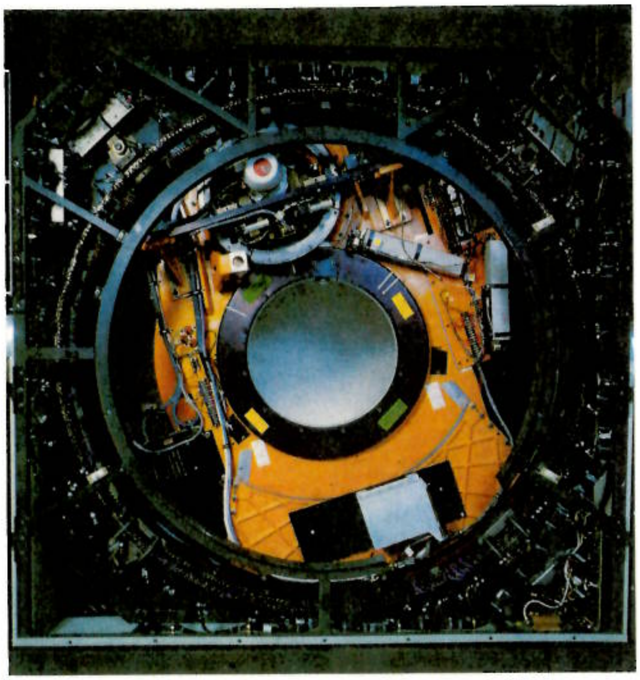

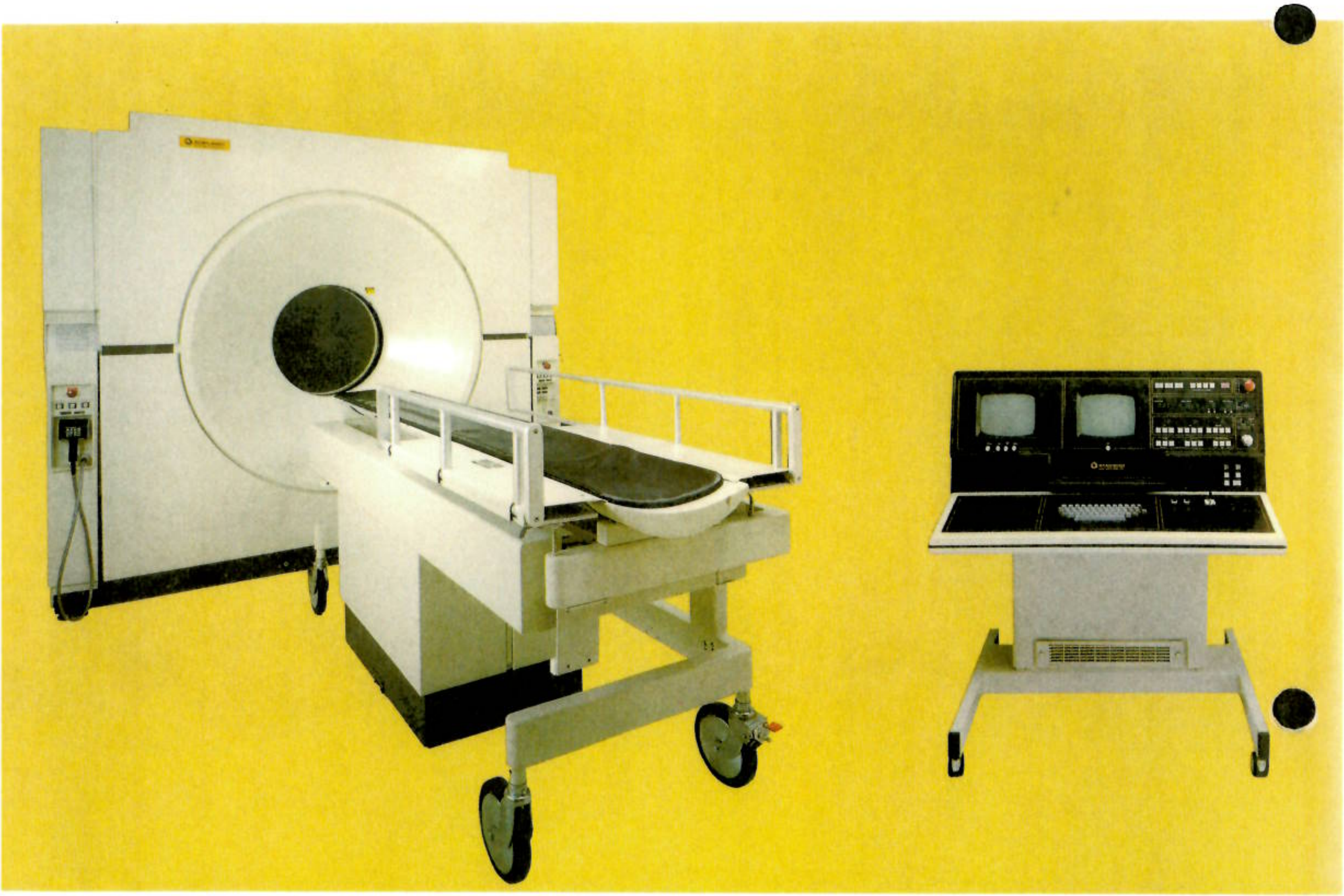

A Dedication to Uptime Reliability

The worldwide Deltascan service force, over 700 strong, undergo rigorous, comprehenisive training at our International Training Center. This training ensures Deltascan 2020 service engineers complete familiarity with every component of the modular system. With plug-in circuit boards and integrated circuit chips, servicing is fast and efficient, thus avoiding extended periods of down-time. Parts for servicing are maintained in Regional Service Centers worldwide. Plus a trained group of Technical Specialists is available for consultation and specialized support, on site when necessary. Each of Deltascan's highly trained service personnel is dedicated to maintaining the operational reliability of your CT system.

Site Readiness

Appropriate site preparation is another service feature provided to Deltascan customers. A Site Planning Specialist, experienced in a wide range of CT installations, will make a detailed study of space availability, patient flow, and individual operational requirements. Based on this information, site drawings and specifications are prepared. The completed drawings, including detailed information on electrical, shielding and air conditioning requirements are forwarded to the customer for approval. Prior to delivery a follow-up survey is made to ensure site readiness and accuracy. Site planning is a joint effort between the customer and Deltascan. Disruption of normal hospital routine is minimized to ensure a fast, smooth installation.

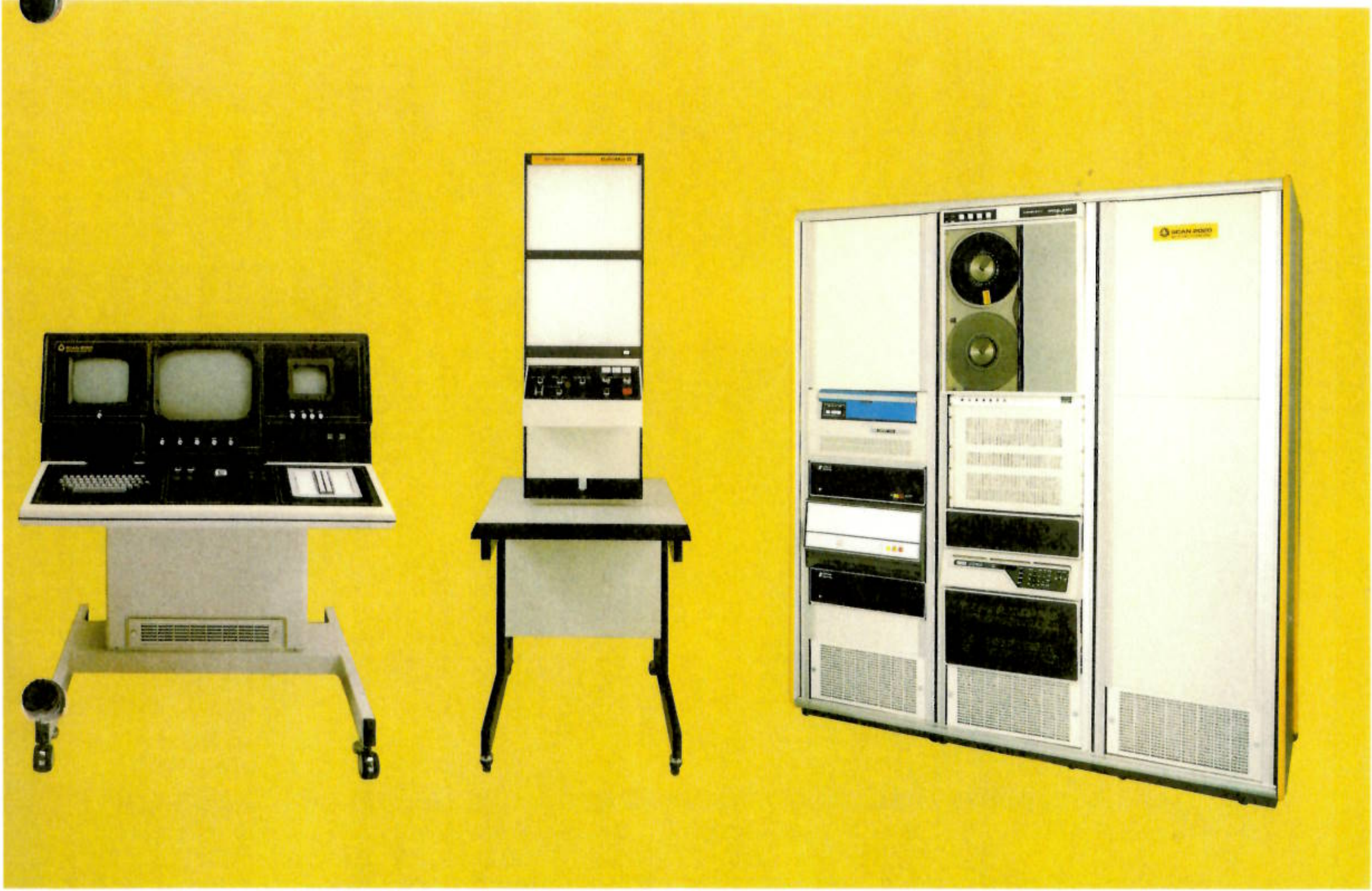

DELTASCAN 2020

Full Range of CT Systems

Deltascan offers you the largest selection of head and body CT systems available today, with performance ranges such as scanning speeds from 120 to 2 seconds, reconstruction times from 1 to 55 seconds and slice thickness capabilities from 4 to 13mm. Investigate the full line of Deltascan CT systems, the Delta 50 Fast Scan, 2005, 2010, or the incomparable 2020—plus a full line of dedicated head scanners. Whatever your requirement, Deltascan can offer a system configured to meet your budget and your needs. Contact your Deltascan representative for more information on any of our products.

TECHNICARE: Since 1974, an industry leader in providing physicians with sophisticated software programs for computed tomography. TECHNICARE Deltascan Division is an international organization serving 40 countries with more than 500 CT installations worldwide, and an enviable record of over seven million CT studies completed. TECHNICARE: a proven record of expertise, installation and service and the most complete line of CT Head and Body scanners available today. Contact your TECHNICARE Deltascan Division representative for complete information regarding the system that best meets your budget and needs.

Specifications:

Scan Speed	2, 4, 8, 16 seconds selectable
Reconstruction Matrix	512x512 or 256x256—displayed and stored<br>128x128—displayed only
Reconstruction Speed	6 seconds—128x128 "quick-look"<br>30-50 seconds—256x256 matrix<br>40-65 seconds—512x512 matrix
Scan Field Diameter	Head Scans—12*, 25cm<br>Body Scans—40, 50cm
Slice Thickness	2mm*, 4mm, 7mm, 10mm
Spatial Resolution	5 line pairs at 25cm scan diameter
Contrast Resolution	3mm at 0.5% contrast (800mAs with shaped head filter)
Noise	0.5% at 800mAs
Dose	Flat Filter 20mR/mAs<br>Shaped Filter 6mR/mAs
Technique Factors	Current 25, 50, 75, 100mA<br>Voltage 80, 100, 120kVp
Gantry	Aperture—60cm<br>Tilt ±20°
Image Storage (80mb)	Disk—400 images 256x256<br>125 images 512x512<br>Tape—200 images 256x256<br>50 images 512x512
Computer Hardware	PDP 11/34 196 K-bytes main memory<br>AP 120B Array Processor<br>1 Megabyte mass memory<br>5 M6800 Microprocessors
Hard Copy	Deltamat Multiformat Camera<br>2"x2", 3"x3", or 4"x4" on 9"x14" film<br>2"x2" on 8"x10" film<br>Polaroid Bezel mounted camera

*Available in 1980.

Class II He-Ne Laser—light localizer for patient positioning.

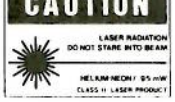

TECHNICARE
DELTASCAN DIVISION

29100 Aurora Road
Solon, OH 44139
Telephone: (216) 248-1800

International

OHIO-NUCLEAR/UNIRAD GMBH
Gustav-Stresemann-Ring 12-16
D-6200 Wiesbaden
West Germany
Telephone: 6121/362-1

ALOKA CO., LTD.
6-22-1, Mure
Mitaka-shi
Tokyo 181 Japan
Telephone: (0422) 45-5111

A Johnson & Johnson Company

© Copyright 1979 TECHNICARE Deltascan Division
All Rights Reserved
119/10K Printed in USA

Elscint

Dual-Slice™ Spiral CT

CT Twin™

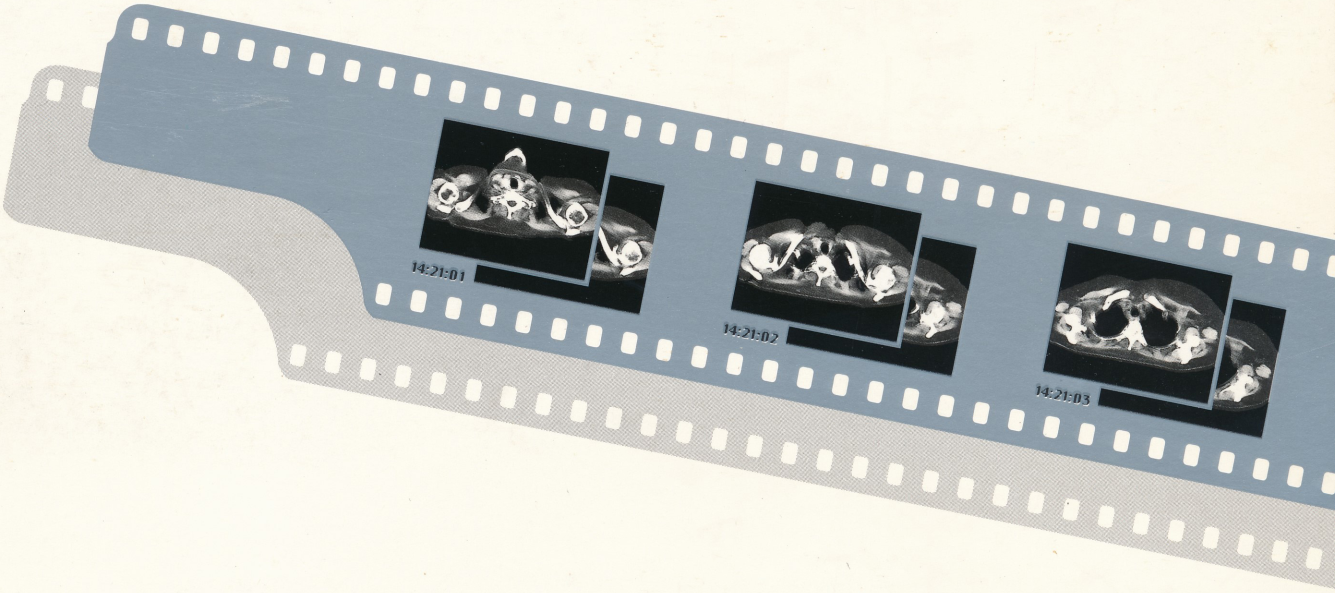

Revolutionary Twin-Beam™ technology...

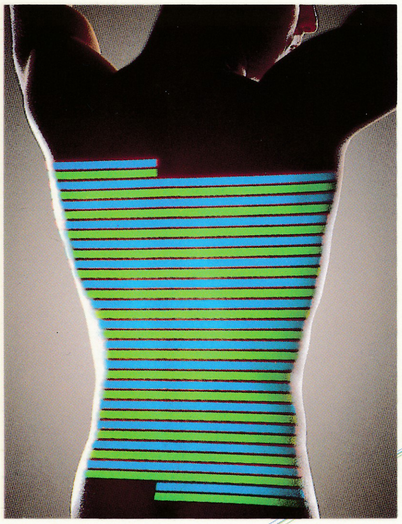

Double-Helix scanning: full-resolution double-volume spiral examinations of 60 cm length in only 30 seconds.

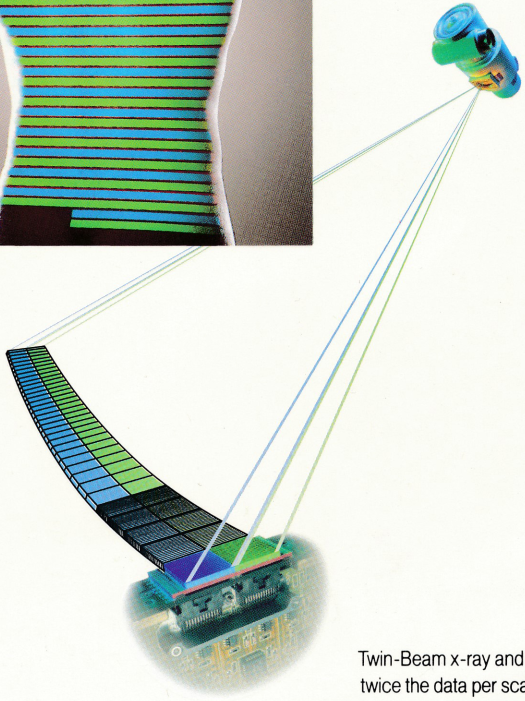

Twin-Beam x-ray and detection system acquires twice the data per scanner revolution. Every scan produces two contiguous slices.

For nearly two decades, clinicians and scientists have shared a vision of a CT "dream machine", capable of scanning two slices simultaneously. After years of intensive research and development Elscint has advanced the Dual-Slice concept and based upon it a spiral CT system with unrivaled Double-Helix™ performance.

The Twin-Beam system, a unique combination of two closely-packed solid-state detector arrays with a patented Dynamic Focus (DFS™) x-ray design, mounted on the most advanced Slip-Ring CT yet developed, make the dream machine a reality... the CT-Twin.

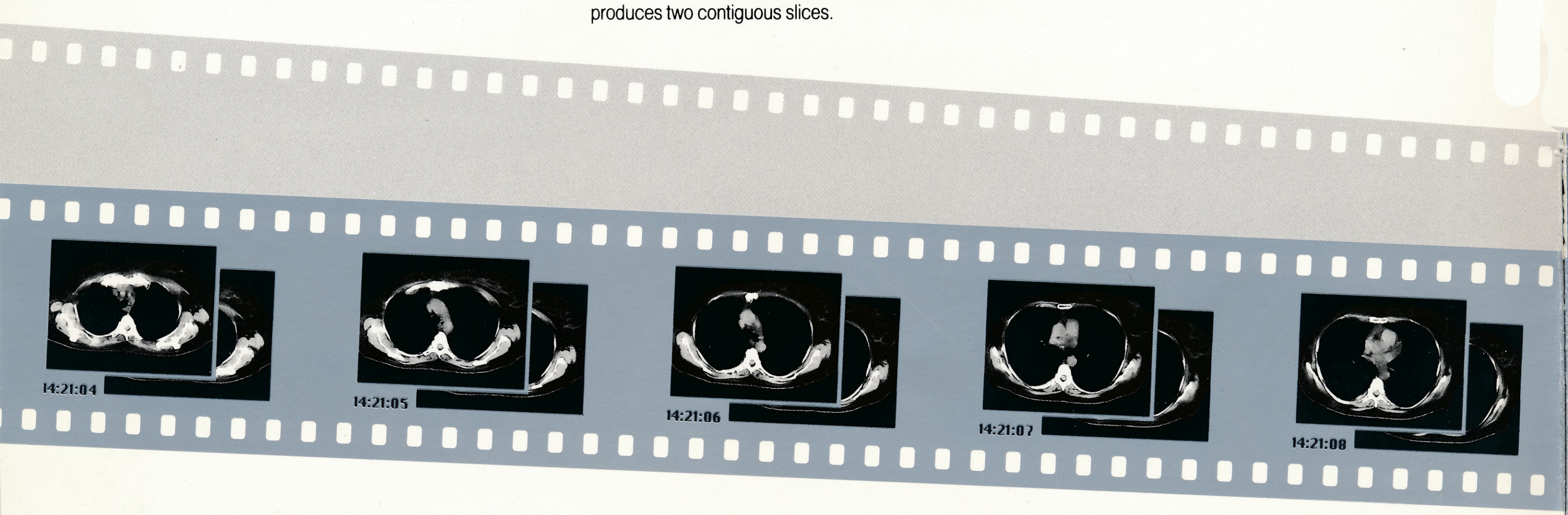

...CT as it was meant to be.

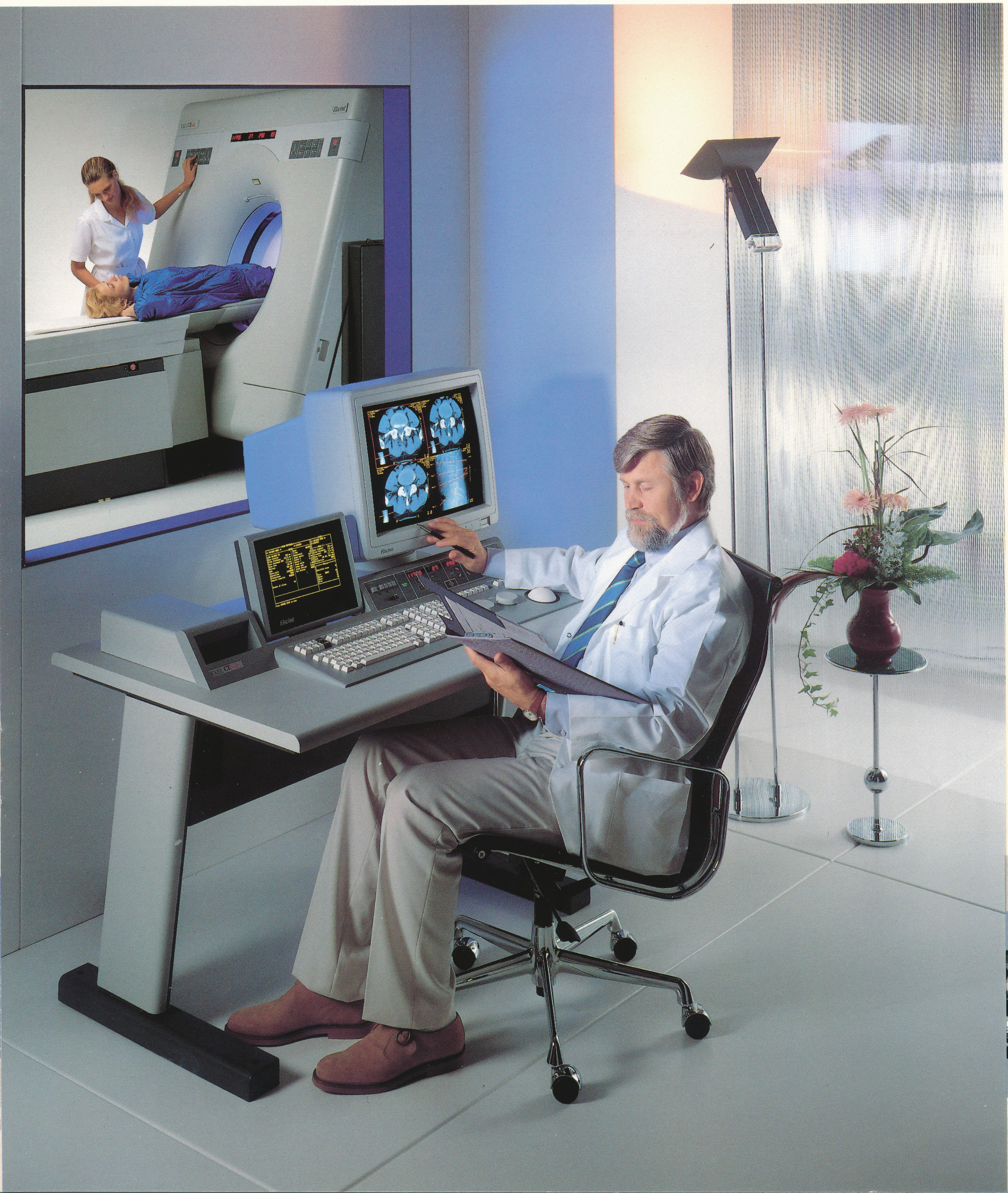

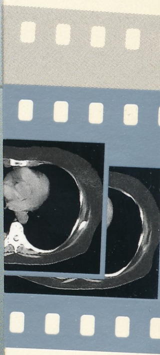

Unique imaging procedures...

Double-Helix Spiral Imaging:

achieves double the SPI*.

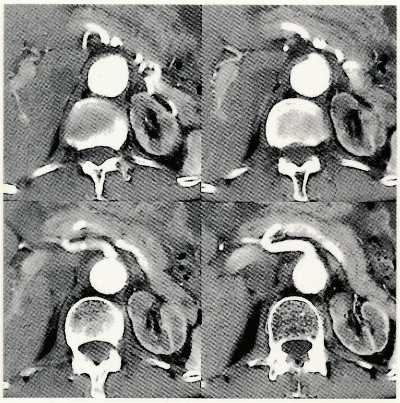

6.5 mm slices reconstructed with 4 mm inter-slice spacing, taken from a 30 second 30 cm Double-Helix volume acquisition.

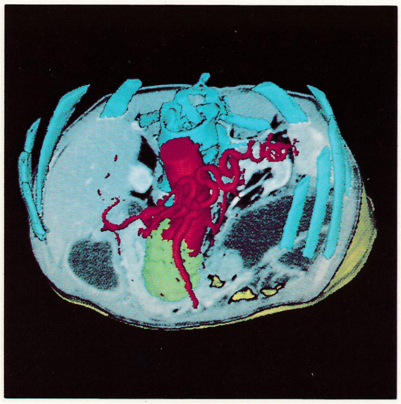

Double-Helix CT angiography: twice the volume at twice the speed with no loss of resolution.

Speed, scan volume and image quality—all crucial factors in the evaluation of spiral scanning. CT-Twin represents a breakthrough in spiral performance, a doubling of SPI*. With twice the efficiency, Double-Helix spiral scanning doubles your clinical capabilities. Twice the length at twice the speed, with no loss in image quality. Any spiral protocol—from detailed lung and CT angiography to full torso, abdominal and GI studies—simply twice as good.

**** Spiral Performance Index (SPI):***

$$SPI = \frac{volume}{time \times width}$$

Three factors affect spiral performance: scan volume, acquisition time and effective image slice-width. CT-Twin sets the standard in spiral imaging.

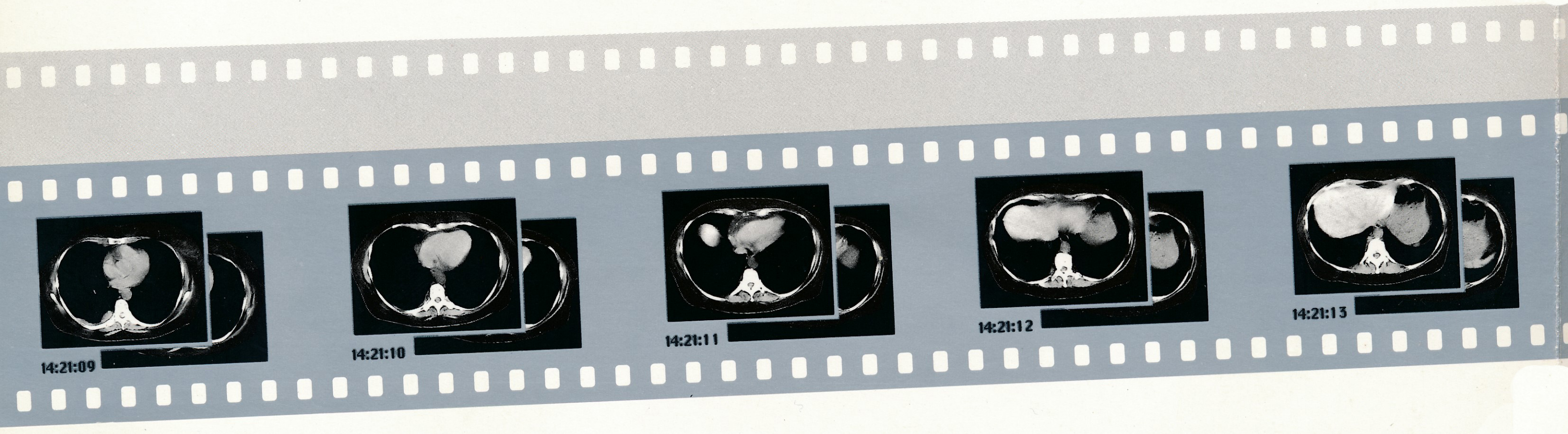

Through thick and thin, any CT-Twin examination can be examined with both thick-slice quality and thin-slice resolution. Consider completing an abdominal study and then retrospectively splitting each image into two detailed thin slices. No need to retake.

Simultaneously scan two contiguous thin-slices and fuse them into a single thick-slice image. Apply fused imaging to posterior-fossa studies, and the result is unprecedented clarity, with significantly reduced partial-volume artifacts.

Split™ Imaging:

both thin and thick slices without rescanning.

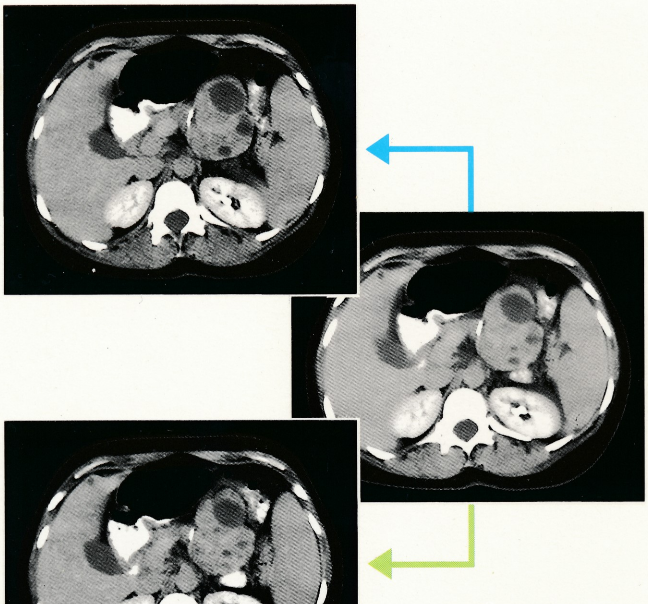

Liver lesions apparent in 10 mm slice width, fully detailed when split retrospectively into two 5 mm slices.

Fused™ Imaging:

thick-slice detectability, thin-slice quality.

Significantly improved posterior-fossa visualization when two simultaneous 2.5 mm slices are fused into a single 5 mm low-noise image.

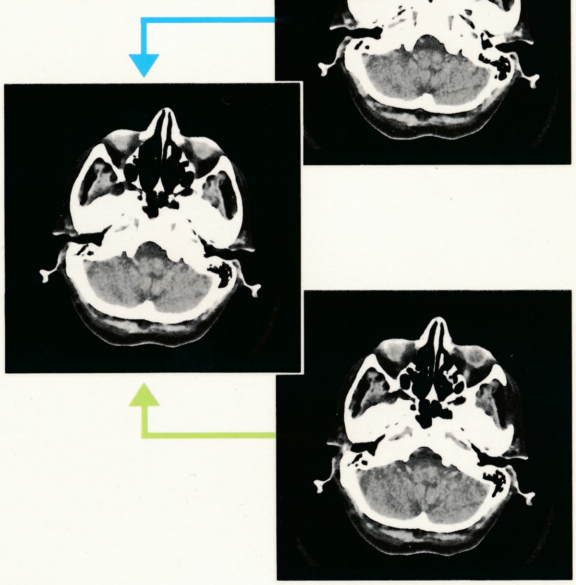

...Exclusive clinical benefits.

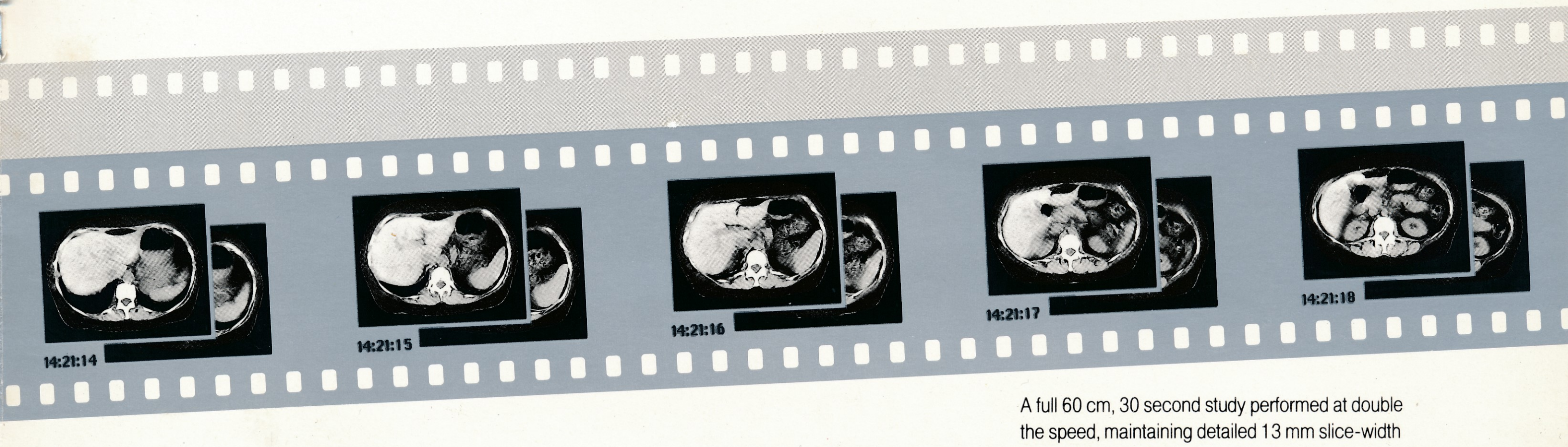

A full 60 cm, 30 second study performed at double the speed, maintaining detailed 13 mm slice-width resolution.

A new approach to system control...

...Unprecedented diagnostic efficiency.

The double performance of the CT-Twin demands a completely new concept in workstation design and configuration: the OmniView™. Picture it! CT-Twin routinely acquiring, reconstructing, archiving and displaying two simultaneous images every 6 seconds. Each image within its own frame on the OmniView display, each frame under independent control.

Uninterrupted patient throughput at an unprecedented pace supported by a 5 MHU x-ray tube, effectively doubled to 10 million heat units by the Twin-Beam system, and a 60 kW rotor-mounted high-frequency generator for reliable 1 second scanning.

OmniView; a unique multimedia concept with multiple image areas, each under independent control - all packed onto one 1280 x 1024 high-resolution color display. Single keystroke functions initiate realtime processing: multi-planar reformatting, 3D image manipulation, multi-format display, zoom and pan, and more. All gantry controls and displays are conveniently located above the keyboard.

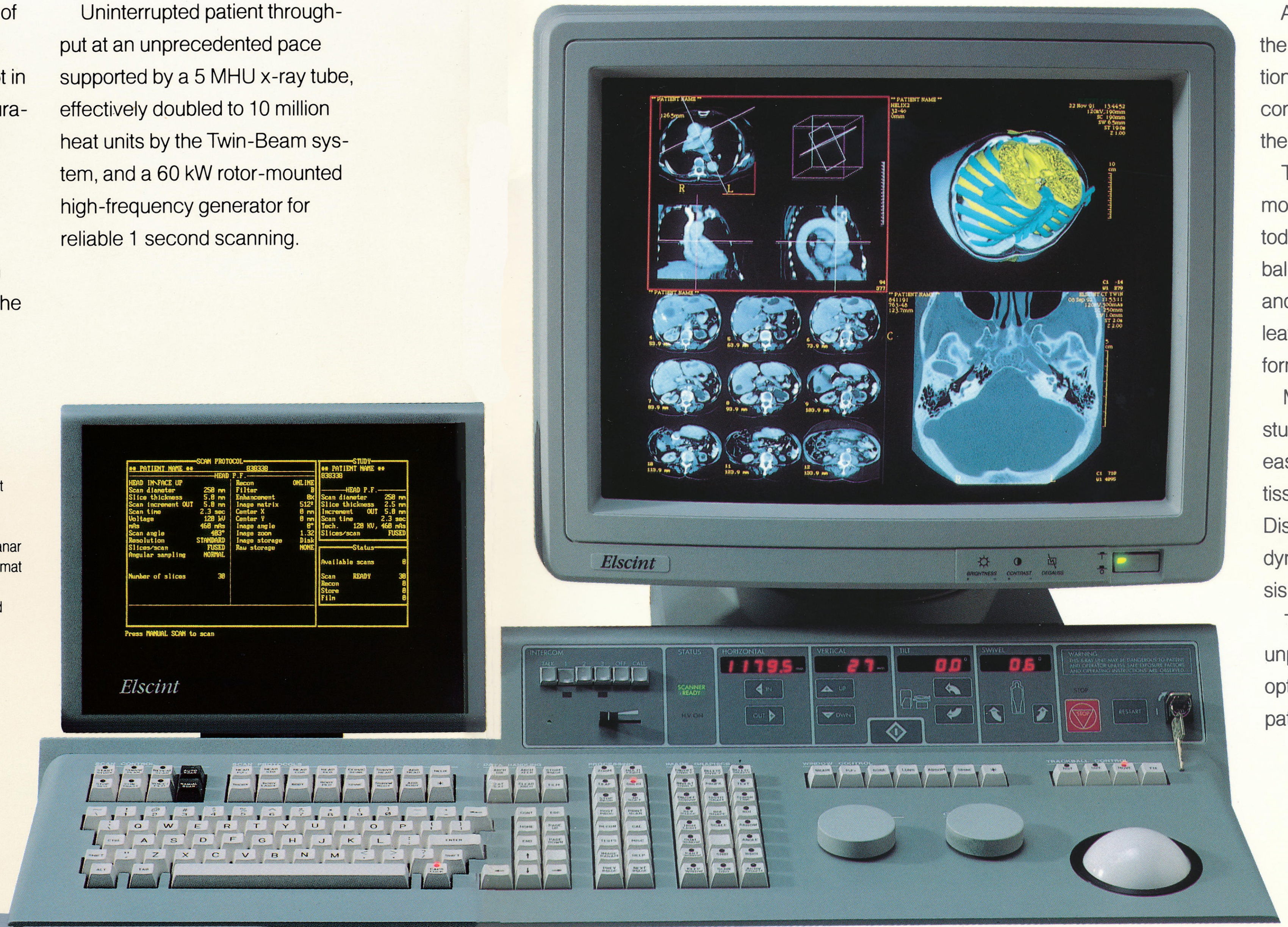

As the study proceeds, swivel the patient to the precise angulation, with all table and gantry controls conveniently clustered at the OmniView workstation.

Take realtime control of the most powerful processor in CT today. Zoom and pan with trackball speed, instantaneous planar and curved reformatting, cine leafing, and full-screen multi-format display.

Manipulate volumetric spiral studies with breathtaking realtime ease. 3D visualization with tissue cutting, Shaded Surface Display (SSD) angiography, dynamic contrast perfusion analysis and more.

The OmniView approach: unprecedented user flexibility, optimal operator ease, premium patient throughput.

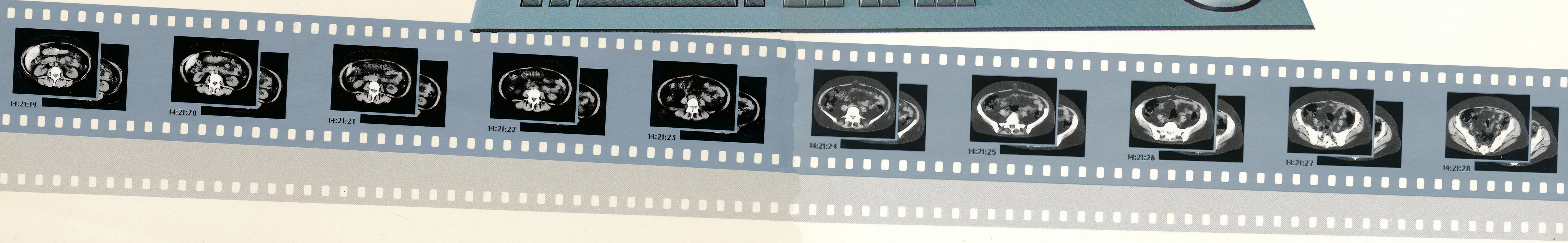

CT-Twin: *Technical specifications*

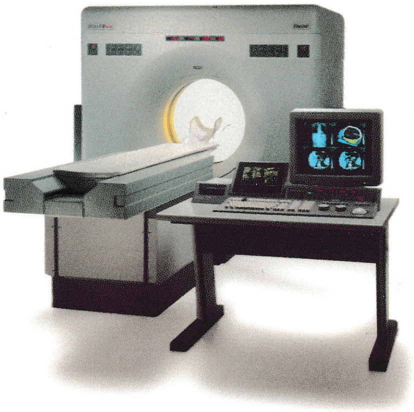

Scan performance

Scanner type: Dual-Slice continuous rotate/rotate

Detectors: 1052 solid-state

X-ray tube: 5 MHU dynamic focus system

Exposure techniques: 90 to 140 kV
20 to 400 mA continuous

Fields of view: 18, 25, 43, 50 cm

Axial scan

Scan times: 0.6**, 1, 2, 4 sec

Slice widths: 1.0, 2.5, 5.0, 10.0 mm

Dynamic cycle time: 20 scans/min

*Double-Helix spiral scan**

Scan times: up to 32 sec

Scan length: up to 60 cm

Slice widths: 1.1 to 13 mm

Scan rate: 1.5 to 20 mm/sec

SPI (Spiral Performance Index): 1.0 to 1.54

Patient handling

Gantry aperture: 70 cm

Gantry angulation: -30° to +20°

Table swivel angulation: ±12°

Table load capacity: 215 Kg / 475 lbs

Imaging performance

Reconstruction time: 2 to 5 sec

Image cycle time: 3 to 6 sec

Spatial resolution: 20 lp/cm in 25 cm fov

Contrast detectability: 0.25% for 3.0 mm pin at 120 kV, 300 mAs, on 16 cm ATS phantom, 30 mGy peak dose

Reconstruction matrices: 340^2, 512^2, 768^2, 1024^2

Display: 1280 x 1024 color multi-display

Image storage: typically 2100 512^2, images on 760 MB disk; 4500 512^2 images on 1.0 GB, erasable optical disk*

*Application features****

AutoVoice patient instruction system

AutoFilm laser imager interface*

Realtime image zoom and pan

Realtime planar and curved oblique reformatting

Realtime 3D imaging

DentaCT™ oral prosthetic implantation planning*

QCT trabecular bone mineral analysis*

Stereotactic surgery planning

* Optional ** 223° scan angle *** Partial list

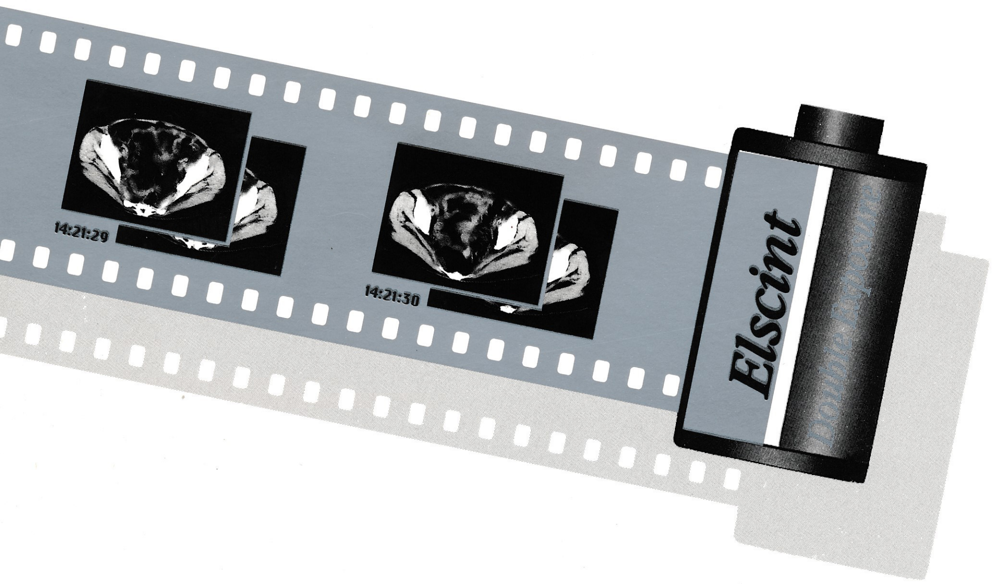

CT-Twin, Dual-Slice, Twin-Beam, DFS, Double-Helix, Split and Fused imaging, OmniView and DentaCT are trademarks of Elscint Ltd.

Elscint CT
The Intelligent Image

Belgium: Zaventem. Tel: (2) 720.92.46. **Brazil:** Sao Paulo-S.P. Tel: (11) 869-4644. **Canada:** Markham, Ontario. Tel: (416) 474-1229. **France**: Bagnolet. Tel: (1) 48-57-08-18. **Germany**: Wiesbaden. Tel: (61) 22/7070. **Hong Kong**: Tel: (5) 292231. **Israel**: Haifa. Tel: (04) 310310. **Italy**: Milano. Tel: (2) 39320603. **Mexico:** Mexico D.F. Tel: (5) 254-5939. **South Africa:** Auckland Park. Tel: (11) 482-3000. **Spain:** Barcelona. Tel: (3) 209.21.99. **U.K.:** Watford, Herts. Tel: (923) 239511. **U.S.A.:** Hackensack, New Jersey. Tel: (201) 342-2020; 1-800-228-7226.

Data subject to change without notification. © Elscint, 1993

CT/P/13/500/0193

original invitation and paperwork not passed through this office.

THORN EMI Central Research Laboratories

Trevor Road, Hayes, Middlesex, UB3 1HH England
Telephone 01-573 3888 Telex 22417 Cables Emisearch London

Your ref
Our ref: GNH/AL
Date: 24.2.83

Mr. J. Harris
Thomas Magnus School
Newark & Trent
Notts.
NG24 4AB

Dear Mr. Harris,

May I thank you and your staff so much for the cordial welcome you gave to me and my family at the opening of the School Library extension. It was a most pleasant occasion on which to renew old acquaintances.

My family and I were greatly impressed by the friendly atmosphere and by the very well organised programme you presented. Would you be good enough to convey our thanks to the Board of Directors and, in particular, to Mr. Beaumont and Lt. Col. Derry for the kind remarks they directed to us. I should like to thank the school once more for the beautiful Plate they presented to me which, I can assure them, will have a proud place in my home and will be a valued momento of a most enjoyable afternoon.

It was also very kind of you to show me around the school and allow me to visit the upper forms. I really appreciated the opportunity of meeting and talking to some of the students.

Yours sincerely,

Godfrey Hounsfield

Godfrey Hounsfield

A Member of the THORN EMI Group
A Division of EMI Limited
Registered in England No. 255852
Reg. Office: Blyth Road, Hayes, Middlesex

I was very pleased to receive you

It was with great pleasure that I accepted the kind invitation to open this new Library, and I do appreciate the great honour of having it named after me. There are not many things I'd rather be associated with than a building designed to help young people with their studies.

DAYS AT MAGNUS

As an old Magnusian, now more than "Forty Years On", I am naturally delighted to visit my old school again and to see how it has grown. How different it looks now! I can still remember my first years at Magnus when I was taught for a while in a temporary classroom that was an ex-Army hut with a coke stove in the middle - it was quite a cosy place.

Mr Carswell and Mr Vernon, two of the staff who patiently tried to teach this rather reluctant student, are happily with us today, and so some of my thanks must also go to them and to all the other teachers who tried hard to educate me. I'm particularly grateful for the grounding in physics and maths that I received at Magnus, and sincerely wish the school as much success in the future as it has had in the past.

THE IDEA OF THE SCANNER

I have been asked to say a few brief words, particularly to the students present here, about the work I did in developing the system of the medical scanner which I had the fortune to invent. It now has the sophisticated name of Computed Tomography. I don't want to burden you with detail, but I'd like to remind you that it is a machine using X-rays that examines the body as a series of slices, much as you would get using a bacon slicer. It has the advantage of being very much more sensitive than conventional X-ray techniques. This helps medical diagnosis, as soft tissue organs can be clearly seen.

Many people have asked me how I got the idea of this invention. They are very disappointed, I assure you, when I tell them that I didn't jump out of a bath, rather like Archimedes, shouting, "Eureka! I have found it!" No, the idea came much more slowly than that.

PATTERN RECOGNITION - THE BLACK BOX

At that time I was busily engaged in what was then called "Pattern Recognition". This meant, in my case, trying to teach a rather stupid computer, with a camera coupled to it, how to recognize objects placed in front of the camera. ~~This~~ required a certain amount of complicated information processing.

~~It was one day, probably in my spare time at home, that~~ the idea occurred to me that, if there were a box with an unknown object in it, and if an X-ray beam and ~~a~~ detector were to take accurate absorption measurements through the box at a multitude of different angles, then a computer should be able to work out in detail exactly what was inside the box and present it in picture form. You see, each measurement would be interrelated with other measurements in the form of hundreds of thousands of simultaneous equasions. ~~This~~ could be handled by a computer, which would be good at solving ~~this~~ vast amount of data. All of this information could be transposed into pictures in three dimensions.

It was a very exciting moment when I realized, ~~after further study, that this system~~ was indeed capable of producing pictures which were very much more sensitive than the conventional X-ray pictures that we know at present!

APPLICATION TO THE HUMAN BODY

I wondered ~~whether~~ this method could be ~~used~~ to look inside the human body, as I had theoretical evidence ~~that~~ it ~~should~~ be able to see soft organs such as liver and pancreas. ~~This~~ challenge was far from easy for me because I had no particular medical experience and the firm I was working for had only little medical interest.

~~I first tested the theory~~ by simulating ~~objects~~ within the computer and attempting to reconstruct them, ~~using the~~ mathematical procedure that ~~I hoped to~~ use in practice.

~~This worked so well, that I knocked~~ on the door of the Department of Health and Social Security ~~to show them that it~~

I set out to convince them that it would work

~~would work~~, clutching my only evidence -- a piece of paper with rows and rows of numbers on ~~it to~~ which represented ~~the~~ a reconstructed picture. ~~This~~ which must have impressed them to some extent, as they gave the firm some money to help ~~with the building of~~ to build a laboratory machine which I set out, ~~without delay, to build~~ to do without delay.

SOME BIZARRE EVENTS - LEADING TO SUCCESS

~~This was~~ This turned out to be a rather Heath Robinson ~~connection~~ contraption, made up of old equipment that happened to be lying around, but it triggered off a very bizarre series of events: my crossing London carrying a paper bag full of bullocks' brains; or fetching pigs' carcases, bought from a local slaughterhouse; or grisly specimens of human remains from the local hospital. These were all tried out on the machine. Because this was only an experimental machine, it took as long as nine hours to produce a picture, and on a hot day there was a constant battle to compose a picture before the specimen decomposed.

Looking back now on this period, I know I found it all tremendous fun. It was a great moment when the machine began to display very startling pictures of the interiors of the specimens, and we realised ~~knew~~ then that we had to design and build a proper clinical scanner. This task had its moments of hard work ~~and frustration~~ -- it was a field completely new to me. but it did bring me into close contact with medicine ~~[illegible]~~. The machine was eventually installed in a London hospital, and you can imagine what a great day it was for me when the first patient was scanned. A tumour in the head was clearly seen, in detail sufficient for a successful operation to be performed. It was gratifying to know that this could not have been seen in any other way at the time.

GROWING MOMENTUM

From then on, events moved very rapidly. and we ~~[illegible]~~ ~~We~~ built hundreds ~~more~~ of machines, which were sent all over the world. ~~We were seeing many abnormalities in the body which could not be seen by any other means.~~

Meanwhile, I was rushing around the world, introducing the system to hospitals and studying the findings of the many doctors who were using the scanner. in order to perfect its design. This is still continuing to this day. ~~[illegible]~~ magnetic fields ~~[illegible]~~ instead of x-rays ~~[illegible]~~ being used ~~with great success~~. nowadays. This is called Nuclear Magnetic Resonance.

Other methods of scanning the patient using

In addition methods using magnetic fields instead of x rays are being used. This is called Nuclear Magnetic Resonance.

APPEAL TO YOUTH

Well, I hope I haven't spoken too long about what is, after all, history, past and done with. ~~Now we have to look to the future and towards the youth of the country.~~ My purpose in telling ~~you~~ ~~my~~ this story is to impress those of you who are about to leave school, especially those who are going into science and technology, that ~~it~~ a career in this field can be an extremely rewarding experience, particularly if you can carry through an idea of your own making, as I did.

Remember, too, that Each new discovery that is made brings with it the seeds of other, future, inventions. There are many discoveries, probably just around the corner, waiting for someone to bring them to life. Could this possibly be you? ~~[illegible]~~

It is possible that ~~I am sure that~~ many of these ideas will come not in the straight forward way by one saying "I have a problem: I am going to find a solution" ~~but~~ but by saying I have a potential solution; let us find a problem that fits it"

~~In the same way [illegible] this certainly happened with me~~

~~It is apparent that this happened to me.~~

~~That [illegible]~~

~~Cinematography has only come about because of Celluloide not of~~

~~Photography not because of then [illegible] ~~a well known art.~~ as it was by then a well known art~~

~~In the same way as Celluloid was the solution to cinematography ~~and photography as this was by then a well known art~~~~

~~In the same way as the laser is the solution to many problems of the past & will be to many unknown problems of the future.~~

Some drivers of CT market changes

THE RUSH TO ACQUIRE CT TECHNOLOGY | CHALLENGES AND SOLUTIONS: The US was clearly the fasted growing market. After the first presentations in 1972 at Montefiore in the Bronx in May and at RSNA in Chicago in November, there was a huge pent up demand for CT. By the end of 1973 there were 8? EMI Mark-I scanners installed in the US. Each scanner cost about $350,000 and a typical installation-construction cost was $150,000 in a large metropolitan area teaching hospital.

Freestanding Centers: The first non-hospital-based system was xxx in 197x, which was approximately the 40th EMI Mark-I installed. This led to many other entrepreneurs and private Radiology groups setting up their freestanding center, which avoided the large costs that teaching hospitals had to incur because of hospital infrastructure and red tape.

Mobile CT Scanning: Yet another advance was placing CT scanners in portable trucks which resided in hospital parking lots. In 1977, the first mobile CT system, an EMI 1010 was launched in the LA area as a joint venture between American Medical Inc., a large hospital company and the predecessor company to RadNet Inc., today the largest freestanding imaging center company in the country. At the time, many hospitals were uncertain about purchasing their own system and/or were caught up in the long process of trying to qualify for a Certificate of Need (CON). In the 70’s CON regulations were prevalent throughout the country and sharing a CT system solved many concerns and allowed the hospital to see if the demand would justify their own purchase and build a use case for later CON approval.

The engineering required to build a reliable mobile CT system with it massive and sensitive computers that required a temperature, moisture and vibration control was considerable but the engineers at EMI, AMI and Calumet Coach were up to the task. At the peak, there were over 500 truly mobile CT systems serving about 1500 hospitals and many were operating around the clock 7 days a week because of the intense demand and emergency cases requiring an immediate CT exam.

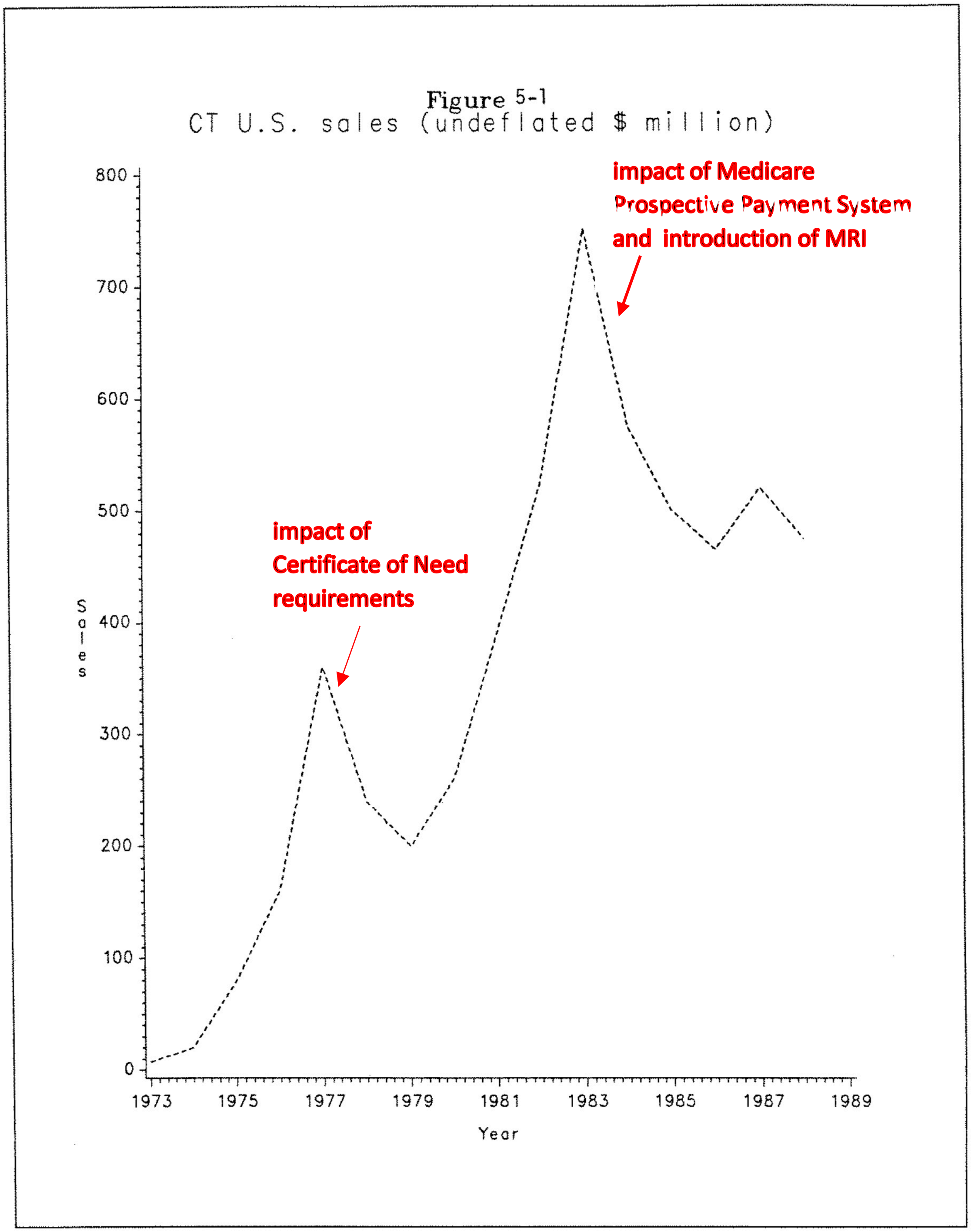

From: "TheDiagnosticImagingIndustry5CT.pdf", downloaded from
http://www-2.rotman.utoronto.ca/william.mitchell/Bio/Dissertation/TheDiagnosticImagingIndustry5CT.pdf
Red annotations added by authors of this paper
